# Assessing similarities and disparities in the skin microbiota between wild and laboratory populations of house mice

**DOI:** 10.1038/s41396-020-0690-7

**Published:** 2020-06-09

**Authors:** Meriem Belheouane, Marie Vallier, Aleksa Čepić, Cecilia J. Chung, Saleh Ibrahim, John F. Baines

**Affiliations:** 1 Max Planck Institute for Evolutionary Biology, August-Thienemann-Str. 2, 24306 Plön, Germany; 2 Institute for Experimental Medicine, Kiel University, Arnold-Heller-Str. 3, 24105 Kiel, Germany; 3 Lübeck Institute of Experimental Dermatology, University of Lübeck, Lübeck, Germany

**Keywords:** Microbial ecology, Microbial communities

## Abstract

The house mouse is a key model organism in skin research including host–microbiota interactions, yet little is known about the skin microbiota of free-living mice. It is similarly unclear how closely laboratory mice, which typically live under exceptionally hygienic conditions, resemble the ancestral state of microbial variation in the wild. In this study, we sampled an area spanning 270 km^2^ in south-west France and collected 203 wild *Mus musculus domesticus*. We profiled the ear skin microbiota on standing and active communities (DNA-based and RNA-based 16 rRNA gene sequencing, respectively), and compared multiple community aspects between wild-caught and laboratory-reared mice kept in distinct facilities. Compared to lab mice, we reveal the skin microbiota of wild mice on the one hand to be unique in their composition within the *Staphylococcus* genus, with a majority of sequences most closely matching known novobiocin-resistant species, and display evidence of a rare biosphere. On the other hand, despite drastic disparities between natural and laboratory environments, we find that shared taxa nonetheless make up the majority of the core skin microbiota of both wild- and laboratory skin communities, suggesting that mammalian skin is a highly specialized habitat capable of strong selection from available species pools. Finally, the influence of environmental factors suggests RNA-based profiling as a preferred method to reduce environmental noise.

## Introduction

The skin serves critical functions as a physical and immunological barrier, but is also a dynamic ecosystem inhabited by diverse microbial symbionts. This ecosystem is influenced by fundamental processes of community assembly including dispersal, local diversification, environmental selection, and ecological drift [1–3]. Understanding the relative contribution of these forces remains challenging and largely unsolved [4, 5].

Natural populations are valuable resources for investigating principles of community assembly and potential selective pressures on the host. Free-living individuals are confronted with heterogeneous environments comprised of diverse species pools and are regularly exposed to a wide range of pathogens, underscoring the need for efficient immune strategies to maintain skin barrier function and inflammatory homeostasis. Several recent studies [6–8], revealed that wild mice reflect the immune responses of adult humans far better than laboratory mice, suggesting that wild mice may be valuable to inspect aspects of the hygiene hypothesis, immune functioning and potential treatment of autoimmune and inflammatory disorders.

Previous efforts explored the composition of skin-associated microbiota and surrounding environments across different species living in the same habitat, and addressed the role of resident bacteria on host survival in natural populations [9–14]. These pioneer studies revealed two key findings: (i) host species identity is the strongest predictor of community composition, (ii) the impact of the surrounding environments on the microbiota composition are limited. Importantly, signatures of phylosymbiosis in the mammalian skin microbiota serve as first evidence of coevolutionary processes [14], which potentially carry fitness consequences such as those observed for the gut microbiota in deer mice [15]. Indeed, several studies in amphibians demonstrated resident skin bacteria to play a critical role in resistance to fungal infection, and thus directly contribute to host fitness in nature [16–19].

The house mouse is a key model organism for microbiome research and is intensively studied [20–23]. Although several studies examined the gut microbiota of free-living mice [24–28] or the skin microbiota of other mammals [14, 29–31] still nothing is known about the normal range of variation of skin microbiota in wild house mice, nor how closely the microbial communities of laboratory mice, which are housed under controlled conditions and experience little to no external stimuli, reflect the natural state of microbial composition in the wild.

In this study we conducted fieldwork and sampled 203 wild house mice over an area of approximately 270 km^2^. We profiled the ear skin microbiota using 16S rRNA gene sequencing based on both extracted DNA-material and RNA-material (standing and active communities, respectively), and thoroughly compared multiple community aspects between wild-caught and three populations of laboratory-reared mice kept in different facilities. We reveal for the first time that the skin microbiota of wild mice is dominated by *Staphylococcus* genus, whose identity most closely matches known novobiocin-resistant species and displays an excess of rare taxa, but that community membership otherwise substantially overlaps between wild and laboratory populations. Moreover, we identify important structural disparities across mouse populations, and detect a pattern of similarity decay in community composition with geographic distance in the wild.

## Materials and methods

### Wild mouse collection and tissue sampling

In September and October of 2013, 203 wild-house mice were collected from 34 distinct farms and stables that were randomly chosen around the southwestern French commune of Espelette. Mean pairwise distance between sampling sites was 10 km, and standard deviation (SD) 6 km. For each mouse, the following parameters were recorded: sex, bodyweight, tail length, body length, female pregnancy status, and farm/stable animal breeding type. Trapped mice were first transported within 2 h to a common location for euthanization with CO_2_ followed by dissection. Dissections were performed with care to avoid cross contamination, whereby ear biopsies [32] only came in contact with instruments that were freshly cleaned (70% ethanol, followed by RNase AWAY) and heat-sterilized prior to each dissection. Two distinct pieces of the left ear were sampled from each mouse for population structure analysis and 16S rRNA gene profiling, respectively. Tissues were stored immediately at −20 °C and then shipped on dry ice to the laboratory, where they were stored at −80 °C until processing.

### Nucleic acid extraction

For population genetic analysis (D-loop and microsatellites), DNA was extracted using the DNeasy Blood and Tissue Kit (Qiagen) following the manufacturer’s instructions. DNA and RNA for 16S rRNA gene sequencing were extracted from the tip of the left ear using the AllPrep DNA/RNA 96 Kit (Qiagen) along with blank negative extraction controls. Samples were homogenized through bead beating using the FastPrep®−96 (MP Biomedicals) 2 × 45 s at speed 4.0, then held for 2 h at room temperature in buffer RLT to increase nucleic acids yield. RNA was treated with DNase (Qiagen) for 15 min, twice. Complementary DNA (cDNA) synthesis was performed using a High-Capacity cDNA Reverse Transcription Kit (AB Applied Biosystems). RNA purity was checked by a negative Reverse Transcriptase control (without transcriptase) PCR reaction and agarose gel electrophoresis.

### Mitochondrial D-loop sequencing and haplogroup analysis

An 885 bp portion of the mitochondrial D-loop was sequenced as described by Prager et al. [33] Sequences edited in Geneious (v.7.0) [34] were aligned in MEGA 5 [35] to *Mus musculus domesticus*, *Mus spretus*, and *Mus spicilegus* reference sequences (GenBank Accessions: AM182648, U47539, U47536, respectively) and sequences from Linnenbrink et al. [24]. A NeighbourNet network was constructed with Huson and Bryant’s hypothesis-poor algorithm using the SplitsTree package (v.4.10) [36]. Individual wild mice were clustered into haplogroups previously defined by Bonhomme et al. [37]. p-distance, which represents the proportion (*p*) of different nucleotide sites between two compared sequences, was calculated in MEGA 5 using default parameters.

### Microsatellite typing and population structure analysis

We typed 18 unlinked neutral autosomal microsatellites as described by Thomas et al. [38] and Hardouin et al. [39] in Geneious (v.7.0). Allele tables were then transferred, and analyzed in STRUCTURE (2.3.4) [40–42] to infer the population structure (See Supplementary Methods). Mice with ≥80% ancestry assigned to a given cluster were considered to be reliably assigned to that cluster, and referred to as “non-admixed”, whereas the remainder were classified as “admixed”, but assigned to the cluster making up the largest portion of their ancestry. Cavalli-Sforza distance (CAS), an absolute measure of genetic distance [43], was calculated using GenoDive (v.2.0) [44].

### 16S rRNA gene sequencing and processing in wild mice

The hypervariable V1–V2 region of the bacterial 16S rRNA gene was amplified using 27F and 338R primers following a dual indexing approach on the Miseq Illumina platform as described in Supplementary Methods. For sequence processing, no mismatch in the barcode was allowed while demultiplexing (Casava, Illumina). Forward and reverse reads were filtered in R (v.3.6.1) using the “DADA2” package (v.1.14) [45] as follows: truncated at the first base for which the quality score dropped below *Q* = 2, no ambiguous nucleotides were allowed, maximum expected errors maxEE were 2 and 5, and minimum length of truncated reads were 250 and 200 bp for forward and reverse reads, respectively. Sequence reads were then subjected to the de-noising algorithm with the “pool=pseudo” option to increase sensitivity to low frequency sequence variants. De-noised forward and reverse reads were merged with a minimum identical overlap of 20 bp, amplicon sequence variants (ASVs) were inferred, and chimeric ASV sequences were removed using the de novo consensus method. Afterwards, ASVs whose length exceeded 350 bp were excluded, and ASV taxonomy was assigned from the phylum to genus level using the Silva reference database (release 132) [46] with bootstrap confidence minBoot=80. ASVs assigned to Eukaryota, unclassified kingdom or mitochondria were excluded.

### Identification of contaminant ASVs

To identify potential contaminant ASVs, we applied the statistical classification procedure implemented in the “Decontam” (v.1.6.0) R package [47]. First, the DNA and RNA concentration of each extracted skin sample was measured, including all negative extraction controls, using the fluorescent Qubit dsDNA and the fluorescent Qubit RNA broad range assay kits (life technologies) for DNA and RNA, respectively. The concentrations are provided in Supplementary Table 1. Specifically, the frequency method was used, which assumes that sequences from contaminating taxa are likely to inversely correlate with sample DNA and RNA concentration. Those negative extraction controls that displayed any PCR amplification (4/4 and 3/4 controls for DNA and RNA, respectively) and whose total processed reads equaled or exceeded 40 were included (4/4 and 2/4 controls for DNA and RNA, respectively). With a probability threshold of 0.1 we identified contaminant ASVs distinctly in DNA and RNA datasets, and excluded the combined defined contaminants (204 ASVs) for subsequent analyses.

### Normalization of sequencing depth across samples

We normalized the read distribution to an equal sequencing depth across samples to (i) reduce biases in subsequent ecological analyses [48], and (ii) further detect inaccurate/suspicious ASVs that may result from sequencing and/or processing artifacts that could alter diversity measures based on taxon presence/absence. Accordingly, we randomly drew 4000 reads (close to the minimum depth in the dataset of 4321 reads) 1000 times independently for each sample. Afterwards the distribution of ASV frequency across the 1000 independent draws was inspected, and ASVs whose frequency across the 1000 draws equaled or exceeded the 10% quantile of frequency distribution of all ASVs were selected, and a final sample of 4000 reads was drawn exclusively from the selected ASVs. Excluded ASVs represent between 0.06 and 2.7% of the initial non-normalized sequences across samples. After normalization, we included 30,361 ASVs jointly in the DNA and RNA datasets (average 250 ASVs, SD = 179)

### Comparison of skin microbiota between wild and laboratory-reared mice

In order to compare wild and laboratory mice, we included three additional groups of laboratory mice reared in distinct facilities: (i) an advanced intercross between *M. m. domesticus* and *M. m. castaneus* strains described in Belheouane et al. [49] (*n* = 225), reared at the University of Luebeck, Germany, hereafter referred to as HL-Lab, (ii) a mixed collection of wild-derived *M. m. domesticus* (*n* = 29) that includes WSB/EiJ (*n* = 9), wild-derived individuals from Cologne/Bonn, Germany (CB) (*n* = 11), and the Massif Central, France (MC) (*n* = 9) that were bred under laboratory conditions following an outbreeding scheme for nearly a decade, hereafter referred to as MPI-Lab, and (iii) the C57BL/6J strain (*n* = 13). MPI-Lab and C57BL/6J mice were reared at the Max Planck Institute for Evolutionary Biology, Ploen, Germany. The handling and sacrifice of wild and lab (see below) mice was conducted according to the German animal welfare law (Tierschutzgesetz) and Federation of European Laboratory Animal Science Associations guidelines. All mice were sacrificed with CO_2_ followed by cervical dislocation. Tissue sampling for scientific purposes was performed according to the German animal welfare law (Permit V 312-72241.123-34). Ear skin was sampled, processed, and 16S rRNA sequences were generated as described above.

Accordingly, we merged the 16S rRNA gene sequence datasets of the wild-caught (*n* = 203) and laboratory populations HL-Lab (*n* = 225), MPI-Lab (*n* = 29), and C57BL/6J (*n* = 13). Sequence processing and the identification of contaminant ASVs was performed as described above. In total, we included negative extraction controls for every individual extraction round (*n* = 21 each for DNA and RNA, i.e., *n* = 42 total). Of these, 18 for DNA and 19 for RNA yielded detectable amplification on an agarose gel using Image Lab Software (Bio-Rad) and were included in sequencing libraries. Of those negative extraction controls that were sequenced, 17 for DNA-based and 10 for RNA-based libraries retained a sufficient number of sequencing reads after processing (≥40 reads; median of 3721 and 3158 DNA and RNA, respectively for negative extraction controls compared to 12,215 and 19,716 DNA and RNA, respectively for real samples) to be included in Decontam analysis. This resulted in the removal of 314 ASVs. We normalized sequencing depth across samples to 2000 reads per sample as described for the wild mice, resulting in the exclusion of ASVs that represent from 3.69 to 0.004% of non-normalized reads. We detected 36,353 ASVs in the entire DNA and RNA datasets (average 165 ASVs, SD: 104).

All statistical analyses were performed in R (v.3.6.1) (R Core Team, 2015). Comparison of main taxa across groups was performed using Wilcoxon tests, and *p* values were corrected for multiple testing according to Benjamini and Hochberg [50]. Indicator species analysis was applied in the “indicspecies” R package (v.1.7.9) [51] using the “r.g” function [52] and 10^5^ permutations. Random Forest classification and regression analyses were carried out using the “randomForest” package [53] (4.16–14) with 10^6^ trees and “mtry=13” for core DNA and RNA genera and “mtry=5” for core *Staphylococcus* ASVs. Alpha diversity indices and principal coordinates analyses of Bray-Curtis and Jaccard indices were carried out with the “vegan” package using functions “diversity”, “estimateR”, and “cmdscale”.

### Identification of *Staphylococcus* and *Streptomyces* species

To determine species-level taxonomy of the *Staphylococcus and Streptomyces* ASVs, we selected samples (DNA) that harbored the highest absolute diversity of traits and amplified a longer portion of the 16S rRNA gene in wild and HL-Lab individuals using genus-specific primers (see Supplementary Methods). PCR products were cloned using the CloneJet PCR kit from ThermoScientific and One Shot TOP10 Chemically Competent *E. coli* from Invitrogen, followed by Sanger sequencing. Taxonomy of trimmed clone sequences (approx. 800 and 500 bp for *Staphylococcus* and *Streptomyces*, respectively) was defined in RDP match (v.16) [54] based on the highest seqmatch score (S_ab). When delta S_ab values were lower than 0.05, multiple matches were reported. Subsequently, sequences of clones, ASV representatives, and outgroups (*Bacillus luciferensis* “AJ419629.1” for *Staphylococcus* and *Kitasatospora kifunensis* “AJ781341.1” for *Streptomyces*) were aligned using the Geneious algorithm, and ASVs were matched to clones based on the highest alignment identity score (percentage of identical bases).

### High-throughput sequencing of the *Staphylococcus tuf* gene

To improve the taxonomic classification of *Staphylococcus* species, we generated partial sequences of the *tuf* gene in a subset of 53 wild (from all 34 farms, 1–4 mice per farm), 41 from HL-Lab, 18 MPI-Lab, and 6 C57BL/6J DNA samples. Additionally, we included two replicates of a microbial community standard (ZymoBIOMICS) containing *S. aureus* and the negative extraction controls (*n* = 17). We followed a two-step PCR strategy using *Staphylococcus*-specific primers and sequenced amplicons on the Miseq Illumina platform. Sequence processing and the definition of ASVs were performed as describe above. Representative ASV sequences were aligned to a *Staphylococcus tuf* gene database, which includes 36 sequences and 1 sub-species used in McMurray et al. [55], and with a sequence from the closest genus to *Staphylococcus* [56] i.e., *Macrococcus canis* (accession number: KM45013) as an outgroup (See Supplementary Methods and Supplementary Table 10). Negative extraction controls were included and treated in the same manner as for the 16S rRNA gene sequencing. A single negative extraction control out of 21 displayed any amplification, but after sequencing processing contained only a single sequence that did not belong to *Staphylococcus*.

### Analysis of sources of variation in skin microbiota composition in wild mice

To further characterize skin microbiota community of the wild-caught mice, we calculated alpha and beta diversity indices based on ASV distribution in the “vegan” package (v.2.5.6) as described above [57]. Representative ASV sequences were aligned in DECIPHER (v.2.14) [58], a distance matrix was calculated in “phangorn” (2.5.5) [59] and a Neighbor-Joining tree was inferred. Phylogenetic diversity (PD) was calculated according to Faith et al. [60] using the “picante” package (v.1.8) [61]. Unweighted and weighted UniFrac metrics [62, 63] based on ASVs were calculated using the “Fast UniFrac” algorithm in the “Phyloseq” package (v.1.30) [64]. The influence of sampling location and host features (sex, weight, proportion of body to tail length, pregnancy, and body mass index) on the composition of standing and active bacterial communities was assessed through a linear mixed effects approach using restricted maximum likelihood (REML) in the “lme4” R package (v1.1–21) [65]. Furthermore, the impact of distance between sampling locations on community structure and diversity was determined (See Supplementary Methods).

## Results

To gain critical knowledge on the microbial communities inhabiting the ear skin of wild house mouse, we analyzed 203 *M. m. domesticus* individuals coming from 34 distinct locations around the southwestern French commune of Espelette. Further, we recently determined that community profiling based on 16S rRNA gene transcripts may better reflect true residents and underlying interactions with the host [49] due to the skin low microbial biomass and potential for environmental noise. Thus, we performed 16S rRNA gene sequencing using both bacterial genomic DNA and RNA reverse transcribed into cDNA as templates, which we refer to as the “standing” and “active” communities, respectively. To compare these data to skin microbial composition typically observed in a laboratory environment, we included three different groups of laboratory mice reared in distinct facilities: HL-Lab [49], MPI-Lab, and C57BL/6J (Table 1, Supplementary Table 1).

**Table 1:** Description of the mouse populations used in this study.

Mouse population	Description	Sex (females, males)	Average age (days)	Breeding environment
Wild	Wild *M. m. domesticus* mice (*n* = 203) caught in the region of Espelette, south-west France	83, 120	Undetermined	Nature
HL-Lab	The 15th generation of an advanced intercross line (*n* = 225) from parental *M. m. domesticus* strains MRL/MpJ, NZM2410/J, BXD2/TyJ, and wild-derived *M. m. castaneus* strain CAST/EiJ purchased from the Jackson Laboratory (Maine, USA)	153, 72	176	Animal facility, University of Luebeck, Germany
MPI-Lab	Mixed collection of *M. m. domesticus* composed of (i) WSB/EiJ (*n* = 9); (ii) two groups that originated from wild-caught mice, subsequently kept under laboratory environment for a decade: Cologne/Bonn, Germany referred to as CB (*n* = 11) and Massif Central, France referred to as MC (*n* = 9)	14, 15	304	Animal facility, Max Planck Institute for Evolutionary Biology, Ploen, Germany
C57BL/6J	C57BL/6J mice (*n* = 13)	5, 8	68	Animal facility, Max Planck Institute for Evolutionary Biology, Ploen, Germany

### Skin microbiota composition in wild and laboratory mice

First, we assessed the relative abundances of the five most abundant phyla (defined as one of the five most abundant taxa in at least two of the four groups, comprising 90.57%, SD = 14.09%, and 82%, SD = 12.32%, of the total abundance for DNA and RNA datasets, respectively), and five most abundant genera (comprising 38%, SD = 18.47%, and 30%, SD = 14.31% of the total abundance for DNA and RNA datasets, respectively). This reveals Firmicutes to be the most abundant phylum in wild, MPI-Lab, and C57BL/6J mice on the DNA level, which is significantly lower in HL-Lab mice, whereas Proteobacteria is more abundant in C57BL/6J.

**Fig. 1:**
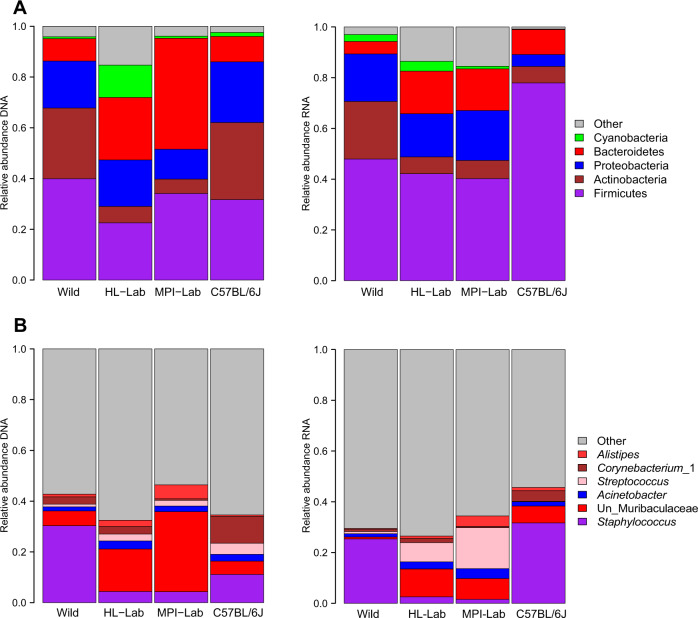
Comparison of major phyla and genera. Mean relative abundances of phyla (**a**) and genera (**b**) across mouse populations in standing (DNA-based) and active (RNA-based) communities. Detailed description of “Other” is reported in Supplementary Table 2. Un Unclassified.

Of note, a clear and significantly higher Actinobacteria abundance is also a shared aspect of the wild and C57BL/6J mice at the DNA level, whereas on the RNA level Actinobacteria remains highly abundant in the wild and Firmicutes dominates the C57BL/6J community (Fig. 1a, Supplementary Table 2). Among the genera detected at the DNA level, a substantially higher abundance of *Staphylococcus, Streptomyces*, unclassified Actinobacteria and *Saccharopolyspora* are a unique aspect of the wild mice, while C57BL/6J harbor different members of Actinobacteria, namely *Cutibacterium* (formerly *Propionibacterium*) and *Corynebacterium*. Interestingly, at the RNA level *Staphylococcus* is equally and highly abundant in wild and C57BL/6J mice (Fig. 1b and Supplementary Table 2).

Although the relative pattern of phylum and genus-level abundances among the four groups of mice is largely correlated between the standing and active datasets, notable discrepancies are the phylum Bacteroidetes and the genus unclassified Muribaculaceae, which are particularly high in the communities of MPI-Lab based on DNA, and C57BL/6J, which harbors different mean abundances of *Staphylococcus* (11 and 31% in DNA and RNA, respectively). Further, unclassified Muribaculaceae overall appears abundant in the standing communities, but is very low in the active communities, suggesting that its detection at the DNA level may represent carry-over from fecal material (see “Discussion” section).

### Diversity of skin microbiota within and between wild and laboratory mice

**Fig. 2:**
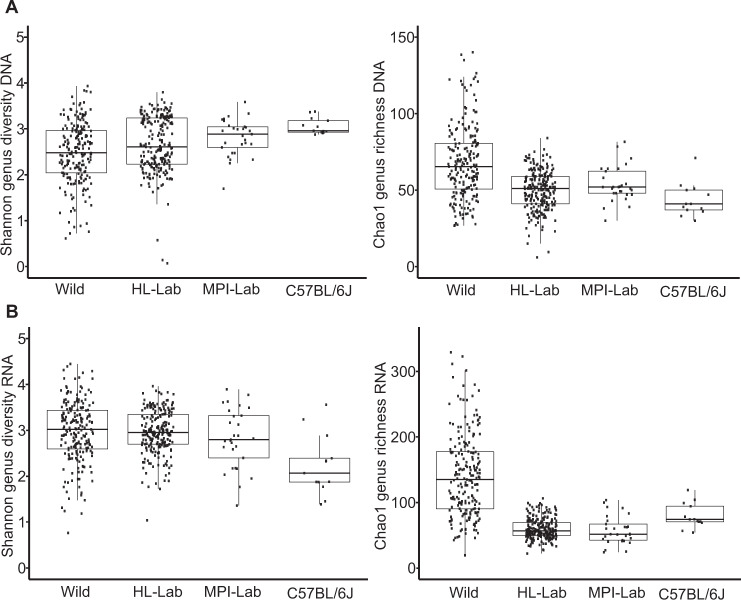
Alpha diversity indices. Shannon and Chao1 indices across mouse populations in standing (DNA-based) (**a**) and active (RNA-based) (**b**) communities. Pairwise Wilcoxon tests are reported in Supplementary Table 2.

To evaluate community structure and diversity in more detail, we performed alpha and beta diversity analyses. Analysis of taxon evenness and richness at the genus level expressed by Shannon and Chao1 indices, respectively, reveals higher richness among wild mice, both in the standing and active communities; whereas evenness is greater in laboratory populations based on DNA, while evenness is lowest in C57BL/6J based on RNA, which is consistent with a strong dominance of *Staphylococcus* (Fig. 2a, b).

**Fig. 3:**
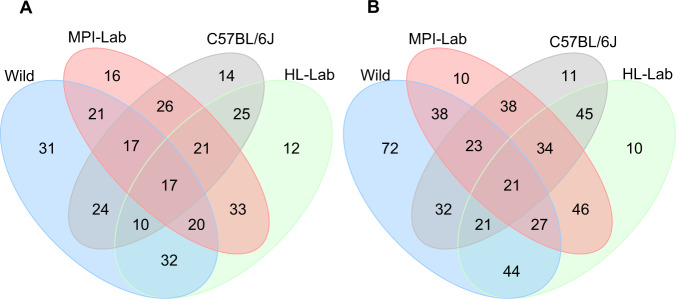
Unique and shared core genera in mouse populations. Standing (DNA-based) (**a**) and active (RNA-based) (**b**) communities. Summary statistics of core and unique genera are reported in Supplementary Table 2.

Furthermore, we performed a simple analysis of shared versus unique taxa at the phylum (Supplementary Fig. 1) and genus levels (Fig. 3). To generate a reliable and accurate picture of taxa inhabiting each mouse population, we limited the analyses to core phyla and genera that we defined within each population’s DNA and RNA datasets as present in at least 25% of the individuals. Core taxa represent approximately 99 and 90% of all phyla and genera, respectively, detected within mouse populations (Supplementary Table 2). In all cases, wild mice display by far the largest number of unique genera, which is consistent with the observations of genus-level richness.

Despite the higher number of taxa unique to wild mice, the smaller number of taxa shared among all four groups (5 and 6 phyla and 17 and 21 genera in standing and active communities, respectively) comprises the highest proportion of the core abundance within communities. Specifically, shared genera at the DNA level represent on average from 58% in wild to 78% in MPI-Lab. Unique genera make up the second largest proportions in wild (27%) and C57BL/6J (19%), whereas unique genera represent comparatively minor fractions in HL-LAB (5%) and MPI-LAB (9%). Shared core genera based on RNA represent between 48% in wild to 65% in C57BL/6J. Notably, unique RNA genera represent minor fractions in all laboratory groups (from 1% in C57BL/6J to 9% in HL-Lab), while in the wild the unique fraction remains the second largest (32%).

**Fig. 4:**
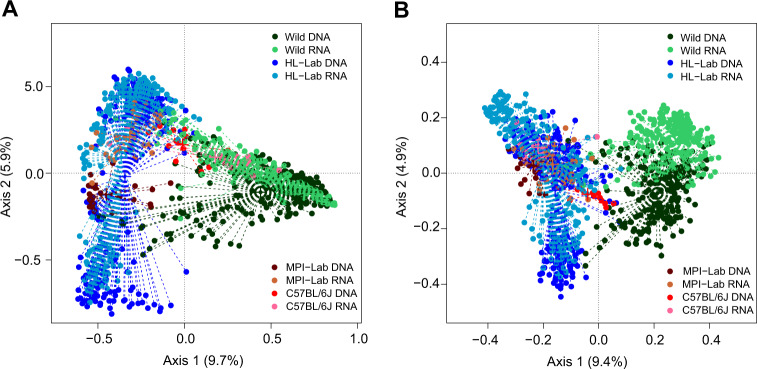
Beta diversity indices. Unconstrained principal coordinates analysis (PCoA) of Bray–Curtis (**a**) and Jaccard (**b**) indices (genus-level) in mouse populations in standing (DNA-based) and active (RNA-based) communities. Goodness of fit of mouse origin: Bray–Curtis axes, *R*^2^ = 0.49, *p* = 10^−5^, Jaccard: *R*^2^ = 0.62, *p* = 10^−5^, based on 10^5^ permutations. “+” centroid of the cluster.

To thoroughly assess beta diversity, we analyzed the Bray–Curtis and Jaccard indices, which are based on weighted taxon abundances and taxon presence/absence, respectively (Fig. 4 and Supplementary Fig. 2). Principal coordinates analysis of Bray–Curtis index reveals a large effect of sample origin, with the first, second, and third axes being significant (Fig. 4a). This effect is larger than that of profiling type (i.e., standing versus active communities). Interestingly, C57BL/6J mice display an intermediate pattern of community structure between the wild, HL-Lab, and MPI-Lab mice. Analysis based on the Jaccard index, on the other hand, reveals a more substantial distinction between the wild and three laboratory mouse groups, with an additionally more pronounced difference between the standing and active communities of the wild mice (Fig. 4b). Moreover, we inspected the influence of additional features including location (farm, family|cage) and sex, and find a significant influence of mouse location and sex (permanova adonis, Bray–Curtis: mouse origin *R*^2^ = 0.21, *p* = 10^−5^, location *R*^2^ = 0.15, *p* = 10^−5^, sex *R*^2^ = 0.004, *p* = 10^−5^; Jaccard: mouse origin *R*^2^ = 0.13, *p* = 10^−5^, location *R*^2^ = 0.14, *p* = 10^−5^, sex *R*^2^ = 0.002, *p* = 10^−5^, based on 10^5^ permutations).

### Indicator species and random forest classification analyses

To identify individual taxa contributing to patterns of beta diversity, we first performed indicator species analysis based on the joint core genera (i.e., the sum of all those present in ≥25% of the mice in any given group) relative abundance and presence/absence in the standing and active communities (Supplementary Table 3). Analysis based on relative abundance in the standing communities identifies *Staphylococcus*, *Streptomyces*, and *Brevibacterium* as strong indicators for wild-caught individuals. Indicators of laboratory mice include *Cutibacterium* and *Corynebacterium_1*, which are strongly associated to C57BL/6J, and several Clostridiales and Bacteroidetes genera associated to MPI-Lab.

In the active communities, *Staphylococcus* is a strong common indicator of wild and C57BL/6J, whereas, *Burkholderia* and *Streptococcus* become significant indicators of MPI-Lab mice. These results are clearly in line with the compositional contrasts described above. Interestingly, presence/absence analysis reveals numerous genera belonging to Actinobacteria, with *Streptomyces* showing the strongest association to wild mice.

To further assess the discriminating power of the core genera, we performed random forest classification analyses. We find that core DNA (*n* = 133) and RNA (*n* = 191) genera accurately classify all individuals to their origin (OOB estimate of error rate: 1.7%, mean classification accuracy across groups 100%) (Supplementary Fig. 3). Moreover, we inspected the mean decrease accuracy component across genera and find that several Actinobacteria taxa including *Streptomyces*, *Brevibacterium*, and *Nocardiopsis* along with *Staphylococcus* are crucial to the accuracy of the classification (Supplementary Table 3).

Given the interesting patterns of *Staphylococcus* abundance across mouse groups, we additionally performed the analyses based on core *Staphylococcus* ASVs (*n* = 33). First, indicator analyses find most ASVs to be strongly associated to wild mice, and fewer with laboratory mice. Specifically, ASV_2, ASV_3, and ASV_4 are associated to wild mice, while ASV_1 and ASV_17 indicate C57BL/6J, and ASV_19 and ASV_77 jointly indicate HL-Lab and MPI-Lab (Supplementary Table 3). Random Forest analyses correctly classifies wild individuals, whereas 15/29 to 21/29 MPI-Lab, and 2/13 C57BL/6J are assigned to HL-Lab (OOB estimate of error rate: 9.79 and 7.23%, mean classification accuracy across groups 96.38 and 94.25% in DNA and RNA, respectively). Importance components find ASV_2, ASV_3, ASV_4, ASV_19, and ASV_11 as most crucial to classification (Supplementary Table 3). These results importantly show that *Staphylococcus* core ASVs are sufficient to accurately discriminate between wild and laboratory mice (Supplementary Fig. 4)

### Diversity of *Staphylococcus and Streptomyces* in wild and laboratory mice

Given the notably higher abundance of *Staphylococcus* as a distinguishing feature of the wild and active C57BL/6J mouse skin microbiota, we further attempted to reveal the identity of individual taxa using a nested approach including genus-specific 16S rRNA gene primers for subsequent cloning and Sanger sequencing, followed by matching ASV sequences. We recovered 223 sequences from ten wild samples (18–26 per sample) that were selected to maximize the recovery of *Staphylococcus* ASVs. Due to the overall low skin biomass and comparatively lower *Staphylococcus* abundance in most laboratory mice, amplification with this primer pair yielded only 25 sequences from 2 laboratory individuals (2 to 23 per sample). In wild individuals, *S. equorum* and *S. xylosus* are preeminent among clone sequences, with comparatively fewer belonging to *S. cohnii* and *S. succinus*. In HL-Lab samples, most clones are *S. epidermidis*, with fewer sequences classifying as *S. hominis* and *S. pasteuri* (Supplementary Table 4).

**Fig. 5:**
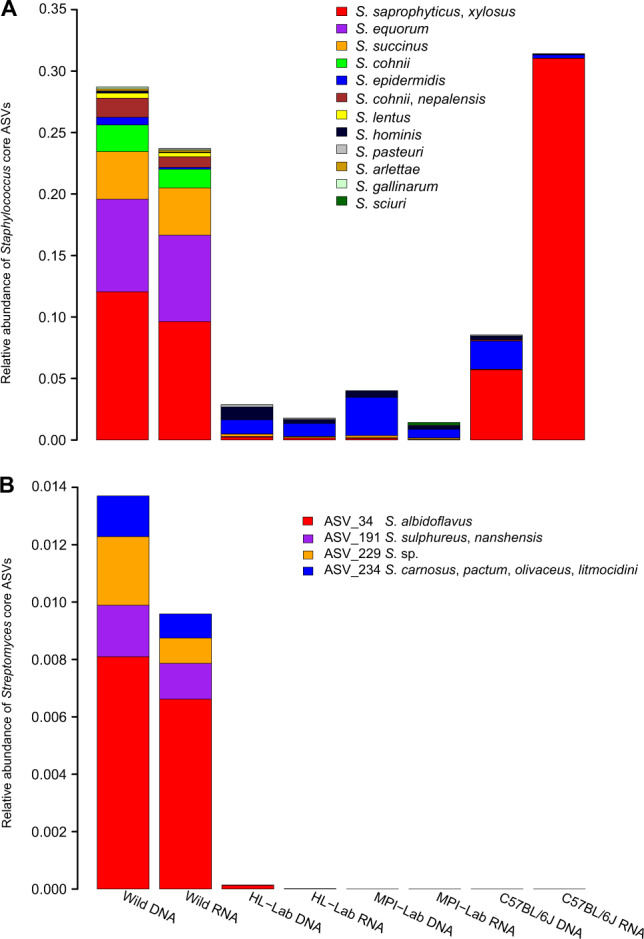
Relative abundance of *Staphylococcus* and *Streptomyces* taxa. *Staphylococcus* (**a**) and *Streptomyces* (**b**) in standing (DNA-based) and active (RNA-based) communities of wild and laboratory mice. Taxonomy is based on Sanger sequencing of genus-specific 16S rRNA gene amplicons.

Next, to assign species-level taxonomy to the core *Staphylococcus* ASVs (n = 33), we aligned representative ASV sequences to the *Staphylococcus* clone sequences obtained above, which yielded matches for all ASVs (Supplementary Table 4). This reveals ASV_1, ASV_3, ASV_4, and ASV_17 to most closely match *S. xylosus* / *saprophyticus*, ASV_2 and ASV_11 to most closely match *S. equorum*, and ASV_19 and ASV_77 to most closely match *S. epidermidis* and *S. hominis*, respectively. Upon inspection of the distribution of *Staphylococcus* ASVs across mouse groups (Fig. 5a), we observe that wild mice harbor high species diversity, in contrast to C57BL/6J, which contains almost exclusively *S. xylosus*/*saprophyticus*. Of note, *S. xylosus*/*saprophyticus* is mostly driven by distinct ASVs in wild and C57BL/6J mice whereby wild individuals harbor ASV_3 and ASV_4 and C57BL/6J show ASV_1 and ASV_17 (Supplementary Fig. 5A). In contrast, same *S. epidermidis* and *S. hominis* ASVs are detected across mouse groups, but remain extremely low in the wild (Supplementary Fig. 5B, C).

To further validate *Staphylococcus* species patterns, we obtained sequences from a portion of the *tuf* gene, which provides better resolution for *Staphylococcus* [66], in a subset of 73 samples including 49 wild, 11 HL-Lab, 12 MPI-Lab, and 1 C57BL/6J. Overall, we detect 779 *Staphylococcus* ASVs, of which 442 could be assigned species-level identity. We find 665 ASVs unique to wild mice, 128 of which belong to *S. xylosus* and *S. equorum*. In contrast, only 50 ASVs are unique to laboratory groups, of which most belong to *S. hominis* and *S. epidermidis*. Interestingly, 64 ASVs are common to wild and laboratory mice, and mostly match *S. xylosus* (9 ASVs), *S. equorum* (8 ASVs), and *S. succinus* (6 ASVs) (Supplementary Table 5). Overall, analysis of *Staphylococcus* based on the *tuf* gene reveals substantial diversity in the wild, with most ASVs belonging to *S. equorum* and *S. xylosus*. While numerous species are shared between wild and laboratory animals, they are characterized by distinct ASVs, as the fraction of shared ASVs remains minor. Finally, another noteworthy observation is that wild mice display a preponderance of taxa whose closest matches are to the phylogenetically closely related group of novobiocin-resistant species [67], which is important in view of the dearth of sequences matching taxa classified as novobiocin-susceptible in wild mice.

Interestingly, novobiocin is also known to be secreted by *Streptomyces niveus* (*S. spheroides*) [68], and *Streptomyces* is similarly a powerful indicator of the wild mice (association statistics 0.91 and 0.86, *p* ≤ 0.05 in standing and active datasets, respectively). Accordingly, we explored *Streptomyces* species in wild mice using genus-specific 16S rRNA gene primers as described above and recovered 135 clones from the eight samples with the highest diversity of *Streptomyces* traits (12–20 sequences per sample). This reveals *S. albidoflavus* (ASV_34) and *Sulphureus*/*nanshensis* (ASV_191) to be widespread in the standing and active datasets. *S. niveus*, the described secretor of novibiocin, is however not detected among clone sequences (Fig. 5b and Supplementary Table 4).

### Sources of variation in skin microbiota composition in wild house mice

To examine potential sources of variation in the composition of wild skin microbiota that may contribute to the community characteristics revealed above, we analyzed environmental and genetic parameters unique to the wild mouse dataset, including geographic sampling location, neutral microsatellite markers, and mitochondrial D-loop sequences. These serve as proxies of the local environment, population structure and maternal transmission, respectively. Analysis of D-loop sequences reveals five distinct haplogroups, whereas analysis of microsatellites reveals *K* = 13 as the optimal number of genetic clusters (Supplementary Table 1). Of the 203 wild-caught individuals, 124 are non-admixed (≥80% of their ancestry is assigned to a given cluster) and 79 are admixed (<80% of their ancestry is assigned to a given cluster) (Supplementary Fig. 6). Using a mixed effect modeling and partial correlation framework to control for the influence of population structure and maternal transmission, we subsequently evaluated the influence of farm characteristics, geographic distance, and host features on community composition in 115 selected individuals.

Among the factors examined, farm or location of sampling is associated with variation in wide-reaching aspects of community composition and structure as expressed by *R*^2^m. In the standing communities (Supplementary Table 6), farm influences the abundance of Bacteroidetes (77.33% of total explained variance) and all four beta diversity measures (Bray–Curtis, Jaccard, unweighted, and weighted unifrac), and explains substantial fractions of variance for the Jaccard index (PC1 90.8% and PC2 88.35%).

In the active communities, farm and weight influence *Staphylococcus* and jointly explain 39.38% of the total variance. Additionally, farm explains important portions of variance in alpha-diversity and beta-diversity measures, and explains up to 74.66 and 63.29% in the Jaccard index (PC1, and PC2, respectively) (Supplementary Table 7).

In addition to farm and host features, we investigated the influence of geographic distance between sampling locations. Based on the geographic coordinates of sampling sites, we calculated Euclidian pairwise distances for the same 115 individuals mentioned above (geographic coordinates of sampling locations are presented in Supplementary Table 8 and Supplementary Fig. 7). Partial Mantel tests included distances between sampling locations as the main variable, and Cavalli-Soforza and p-distance matrices as conditions to account for correlations between genetic (Cavalli-Soforza, *p*-distance) and geographic distances (See Supplementary Methods) (Mantel test: Sampling locations distances-Cavalli-Soforza, *r* = 0.23, *p* = 0.0009; Sampling locations distances-p-distance *r* = 0.19, *p* = 0.0009; Cavalli-Soforza-p-distance, *r* = 0.17, *p* = 0.0009, based on Spearman’s correlation and 1000 permutations). Response variables included all four beta diversity measures in both the standing and active communities. In the standing communities, geographic distance significantly and positively correlates with all four beta diversity measures (Bray–Curtis: *r* = 0.20, *p* = 0.001, Jaccard: *r* = 0.23, *p* = 0.001, unweighted UniFrac: *r* = 0.23, *p* = 0.001, weighted UniFrac: *r* = 0.13, *p* = 0.001). In the active communities, we find positive significant correlations between sampling locations and all diversity measures (Bray–Curtis: *r* = 0.068, *p* = 0.008, Jaccard: *r* = 0.062, *p* = 0.008, unweighted Unifrac: *r* = 0.088, *p* = 0.001, weighted UniFrac: *r* = 0.08, *p* = 0.003). Of note, correlation coefficients are substantially lower in the active compared to the standing communities.

In summary, we find that farm characteristics influence a large number of taxa and diversity measures in the standing and active communities, and despite a relatively restricted sampling area, we detect a “distance-decay” similarity pattern in the skin microbiota among wild mice.

## Discussion

The house mouse is an important model for understanding the skin microbiota in health and disease. Moreover, given the marked differences in the immune system of free-living mice versus those living under the “abnormally hygienic” conditions of specific pathogen-free (SPF) barrier facilities [7, 8], characterizing skin microbiota of natural mouse populations is of critical importance, as it offers a more accurate window into the microbial communities with which mice evolved and provides possible explanations for why wild mice more closely resemble human immune traits. As such, our study reports the first description of the native skin communities of free-living mice, and contrasts standing and active communities between wild-caught and laboratory mice reared in different facilities, including the C57BL/6J strain typically used in skin biomedical research. We reveal a number of salient features of the wild mouse skin microbiota that may bear relevance on future experimental models. In addition, the contrast in patterns observed between standing and active communities among groups of mice provides valuable insight into the general interpretation of diversity patterns among low biomass skin samples.

### Compositional similarities and differences between laboratory and wild habitats

Despite drastic differences in environmental conditions, and thus in potential colonizing species pools between the laboratory and wild habitats, wild-caught and laboratory-reared mice harbor overall similar communities at the genus level, whereby shared taxa comprise the largest part of the core genera abundance within communities (between 58–78% and 48–65% in standing and active communities, respectively). This suggests strong host selection upon the available species pool, whereby the skin is a specialized habitat (e.g., structurally, biochemically) that results in bacterial associations largely independent of the surrounding environment. These observations are akin to those in amphibians, which harbor skin microbiota largely distinct from their aquatic environment [13, 69, 70]. On the other hand, wild mice display significantly higher taxon richness and a higher number of unique taxa than laboratory mice, which is observed in both the standing and active communities. This suggests that these taxa may be true residents and reflect a rare biosphere of unknown functional importance. Compositional differences are also clearly reflected by beta diversity, indicator taxa and random forest analyses, the latter of which identify a number of important candidates such as members of *Staphylococcus* and *Streptomyces*.

### *Staphylococcus* taxa closely matching novobiocin-resistant species dominate the skin microbiota of wild house mice

The observation that *Staphylococcus* species and ASVs markedly differ between the wild and laboratory environments is potentially of great importance. This genus is one of the most dominant taxa of the human skin microbiota, whose individual members can have a profound impact on health and disease, and we reveal that *Staphylococcus* abundance is considerably higher in wild mice, with the exception of high *Staphylococcus* activity (RNA-based abundance) in C57BL/6J. Furthermore, its within-genus composition- defined with a combination of 16S rRNA and *tuf* gene analyses also differs substantially between laboratory and natural populations. Although our study is limited to only metagenomic analysis as samples processed in the field were not prepared for future culture-based analysis and phenotypic screening, we find sequences matching novobiocin-resistant species (*S. xylosus*, *S. equorum*, together with *S. cohnii*, *S. succinus*) to drive *Staphylococcus* abundance in the wild, while laboratory HL-Lab and MPI-Lab mice harbor sequences matching novobiocin-susceptible *S. hominis* and *S. epidermidis*. Notably, *S. hominis*, and *S. epidermidis* are detected in the standing wild microbiota, albeit at marginal abundances. Interestingly, however, we reveal that *Staphylococcus* within the active C57BL/6J skin microbiota is dominated by sequences matching *S. xylosus*, but with ASVs distinct from the wild.

Given the predominance of sequences matching novobiocin-resistant *Staphylococcus* species together with *Streptomyces* being an indicator of wild mice, we investigated whether these observations might be explained by the presence of *Streptomyces niveus*, a known secretor of novobiocin. However, we did not detect *S. niveus* in wild mice. One possible scenario is that novobiocin-resistant *Staphylococcus* species are resistant against other antibiotics present in the wild, to which *S. epidermis* and *S. hominis* are susceptible. Indeed, Resch et al. [71] and Jeong et al. [72] reported that some *S. equorum* and *S. xylosus* strains are resistant to several antibiotics secreted by members of Actinomycetales, such as chloramphenicol, lincomycin and erythromycin. Of note, *S. epidermidis* strains can be susceptible to erythromycin [73], an antibiotic secreted by *Saccharopolyspora erythraea*, and *Saccharopolyspora* is an indicator of wild mice. Thus, other antibiotics potentially present in the wild may lead to a competitive advantage for novobiocin-resistant *Staphylococcus* species.

To date, little is known about interactions between novobiocin-resistant *Staphylococcus* and the skin. *S. xylosus* is described as a skin commensal and potential opportunistic pathogen in human and mouse [74–76]. Interestingly*, S. xylosus* triggered a specific immune response in the mouse skin, similar to *S. epidermidis*, a crucial player in human skin health [77–79]. Furthermore, *S. equorum* strains have been isolated from the skin of animals such as horses and sheep [67], fermented food, and human skin wounds [80]. To date, *S. equorum* has not been described to inhabit the mouse skin. Whether *S. equorum and/or S. xylosus* are functionally equivalent to *S. epidermis* in shaping skin inflammatory and defensive homeostasis requires further exploration.

### Environmental influences differentially reflected by DNA- compared to RNA-based profiling

The mice included in our analyses differ in their environmental origin at a number of levels. First, the laboratory mouse groups were housed in distinct facilities that differ in aspects of animal husbandry, which is known to lead to different fecal microbiota composition between individuals of a lab strain common to different facilities [81]. Our analyses identified an unclassified genus belonging to Muribaculaceae, which is a prominent and specific member of the mammalian gut microbiota [82, 83], to be particularly abundant in the standing communities of MPI-Lab mice. This group is comprised of wild-derived individuals that are many generations removed from their wild predecessors. However, due to behavioral differences compared to inbred lab strains, the MPI-Lab mice are maintained with different husbandry routines, including a lower frequency of cage changing (every two compared to one week for the HL-Lab). Thus, the differential abundance of unclassified Muribacaculaceae (and its phylum Bacteroidetes) at the DNA level in MPI-Lab mice may represent inactive carry-over from fecal material. Second, the wild mice being sampled from 34 different farm locations provided a much broader range of environmental differences than within lab facilities, and multivariate analysis yielded a significant influence of farm on numerous aspects of skin community structure. Of note, farm is nearly the only factor found to influence microbial traits in the standing communities, whereas other factors such as weight and BMI are significant for many traits in the active communities. Further, similar to other studies reporting an influence of geography on the composition of skin microbiota of wild mammals [14, 29–31], we observe a pattern of similarity-distance decay, which is stronger in the standing communities. Taken together, the observation of potential non-skin-resident taxa and a more prominent influence of environmental variables or their proxies (similarity-distance decay) at the DNA level suggest that RNA-based profiling may reduce environmental noise in studies of the mouse skin microbiota.

In summary, we provide a first description of the skin microbiota of free-living wild mice, which yields important insights on the mouse as a model for biomedical research. Despite overall similarity between lab and wild mice suggesting strong host selection, key aspects of wild mice that appear to be missing in the laboratory environment include Actinobacteria genera, unique *Staphylococcus* ASVs and many rare, but active taxa. Given the contrasting immune phenotypes observed in wild- compared to laboratory mice [7, 8], some of which notably also differ in *Staphylococcus* composition [6], the differences observed in our study could represent candidates for differential outcome in skin disease models. Thus, future experimental evaluation of these taxa in the mouse skin, as well as the use of wild mice, would be warranted, and we further recommend the use of RNA-based bacterial profiling for the skin when possible.

## Supplementary information

Supplementary information

Supplementary Figure 1

Supplementary Figure 2

Supplementary Figure 3

Supplementary Figure 4

Supplementary Figure 5

Supplementary Figure 6

Supplementary Figure 7

Supplementray Table 1

Supplementary Table 2

Supplementary Table 3

Supplementary Table 4

Supplementary Table 5

Supplementary Table 6

Supplementary Table 7

Supplementary Table 8

Supplementary Table 9

Supplementary Table 10

## Data Availability

Profiles of neutral microsatellite loci are provided in Supplementary Table 9. Mitochondrial D-loop sequences are deposited in GenBank with accession numbers: MN027281-MN027496. *Staphylococcus* and *Streptomyces* clone sequences are deposited in GenBank with accession numbers: MN134086–MN134339, and MN161249–MN161392, respectively. 16S rRNA and *tuf* genes sequences are deposited in the Sequence Read Archive under BioProject PRJNA549583.
