# Supplementary material for: Assessing similarities and disparities in the skin microbiota between wild and laboratory populations of house mice
Source: ISME J. 2020 Jun 9;14(10):2367–80. doi: 10.1038/s41396-020-0690-7 (PMC7490391; doi:10.1038/s41396-020-0690-7)
Supplement: Supplementary file 1 — Supplementary information [file 41396_2020_690_MOESM1_ESM.docx]

**Supplementary Material**

**Supplementary methods**

**Microsatellite typing and population structure analysis**

The 18 unlinked autosomal microsatellites included: Chr01_25, Chr02_01, Chr03_21, Chr03_24, Chr04_31, Chr05_15, Chr05_45, Chr07_38, Chr08_11, Chr09_20, Chr11_64, Chr12_05, Chr13_22, Chr14_16, Chr16_21, Chr17_09, Chr18_08 and Chr19_08. STRUCTURE (2.3.4) analysis was applied using the following parameters: 500 000 burn-in period and 10^6^ Markov chain Monte Carlo (MCMC) simulations with 20 repeats for each K between 2 and 18. For the choice of K, we applied the criterion of Evanno *et* *al*., [1] and used CLUMPP [2] to compare the 20 repeats for each K and build an average cluster membership from the 20 runs using the “LargeKGready” algorithm with 10 000 repetitions and random input orders, with G’ as the pairwise matrix similarity statistics.

**16S rRNA gene sequencing and processing in wild mice**

The primer pair (5´-*AATGATACGGCGACCACCGAGATCTACAC*XXXXXXXXTATGGTAATTGT *AGAGTTTGATCCTGGCTCAG*-3´) and (5´*CAAGCAGAAGACGGCATACGAGAT*XXXXXXXXAGTCAGTCAGCC *TGCTGCCTCCCGTAGGAGT*-3´) contained the Illumina adapters P5 (forward) and P7 (reverse), denoted by *italics*; whereas the *underlined italic* sequences represent the broadly conserved bacterial primers 27F and 338R. A twelve-base linker sequence (underlined only) was added to the bacterial primer. Both primers contained a unique eight base multiplex identifier (Index; designated as XXXXXXXX) to tag PCR products. PCRs were conducted in a 12.5-μL volume containing DNA or cDNA template using the Phusion® Hot Start II DNA High-Fidelity DNA Polymerase (Thermo scientific). Cycling conditions were as follows: initial denaturation for 30 sec at 98°C; 30 to 35 cycles of 9 sec at 98°C, 30 sec at 55°C, and 30 sec at 72°C; final extension for 10 min at 72°C.

Each round of amplification included PCR negative (no template) controls, whose negative status was used as an inclusion criteria for any batch of samples to be further processed. If evidence of contamination in the PCR reagents and/or the introduction of contamination during the PCR setup was found, this round of PCR was abandoned and repeated until negative. Our dual index barcoding approach includes a set of 16 forward- and 24 reverse primers, and sufficient PCR negative controls were set up each time such that the PCR master mix and every different primer was screened for contamination (detection of amplification on an agarose gel using Image Lab Software (Bio-Rad).

For all samples, PCR product concentrations were first quantified on an agarose gel using Image Lab Software (Bio-Rad). After quantification, products were combined into equimolar subpools. Subpools were then extracted from an agarose gel with the Qiagen MinElute Gel Extraction Kit and quantified with the Quant-iT™ dsDNA BR Assay Kit on a Qubit fluorometer (Invitrogen). Finally, subpools were combined in one equimolar pool for each library. Pools were further purified using AMPure® Beads (Agencourt) and run on an Agilent Bioanalyzer prior to sequencing. Libraries were sequenced using the MiSeq Reagent Kit v3 (600 cycle) chemistry.

**Identification of *Staphylococcus* and *Streptomyces* species**

We amplified

*Staphylococcus* using F(107): CCTATAAGACTGGGATAACTTCGGG, R: (876) CTTTGAGTTTCAACCTTGCGGTCG [3]. *Streptomyces* using F(147) : ACAAGCCCTGGAAACGGGGT, R(647): CACCAGGAATTCCGATCT [4]. Cycling conditions were (i) 94°C for 3.0 min, (ii) 94°C for 1.5 min, (iii) 55°C for 1 min, (iv) 72°C for 1 min, (v) 35 cycles of steps 2 through 4 inclusive, and (vi) 72°C for 10 min.

**High-throughput sequencing of the *Staphylococcus* *tuf* gene**

Using genus specific *tuf* gene primers: forward: 5′-GCCAGTTGAGGACGTATTCT-3′, reverse: 5′-CCATTTCAGTACCTTCTGGTAA-3′ [5] (attached to Illumina sequencing primers and heterogeneity spacers, we amplified a 412 bp fragment using first 2 µl to 4 µl DNA, and the Phusion® Hot Start II DNA High-Fidelity DNA Polymerase (Thermo scientific) using a two-step PCR approach. Conditions of the first PCR were: 98℃ 30 sec, 98℃ 9 sec, 55℃ 1 min, 72℃ 90 sec, repeated 15 cycles, 72℃ for 10 min. For the second PCR, we used 5 µl of PCR product, and Illumina Nextseq Primers that allowed a dual barcode indexing of each sample (Supplementary Table 10). PCR conditions were: 98℃ 30 sec, 98℃ 30 sec, 50℃ 30 sec, 72℃, 90 sec, repeated 20 cycles, 72℃ for 10 min. Both PCR steps were carried in a total volume of 25 µl. Subsequently, PCR products were processed and sequenced as mentioned for the 16S rRNA gene. Next, Fastqs were generated, filtered, de-noised, merged and screened for chimeras as described above. Merged reads shorter than 412 bp and longer than 426 bp were removed. ASVs were defined (n=1559), and using Geneious (v.8.1.7), we aligned ASVs representative, the database, and the outgroup sequences using ClustalW algorithm with 1000 iterations, then trimmed the alignment to equal length (394 bp), and calculated a pairwise distance matrix expressed as percentages of identical residues between sequences.

We further trimmed the aligned sequences as follows: i) ASVs which pairwise distance to the outgroup and to the database did not reach 50% were excluded; ii) ASVs which pairwise distances to the outgroup exceed the highest pairwise distance between a database sequence and the outgroup (90.355%) were removed. Finally, ASVs which highest pairwise distances to the database sequences fell below the minimum pairwise distance within the database sequences (85.787%) were excluded. The remaining ASVs (n=794) were assigned to genus *Staphylococcus*. Subsequently, to assign species identity to the ASVs, we proceeded as follows: in the two replicates of the microbial community standard which contain solely *S. aureus*, we detected ASV_107 which pairwise distance to the *S. aureus* species of the database was 97.208%. Accordingly, we set this pairwise distance threshold to assign species identity, and thus ASVs which highest pairwise distance to a database species equaled or exceeded 97.208% were assigned the species identity of that database species, and ASVs which highest pairwise distance to a database species fell behind 97.208%, were assigned to *S. sp*., and the database species which correspond to the highest match was reported. Finally, we excluded samples which total reads assigned to *Staphylococcus* dropped below 100. We recovered between 133 to 139,964 sequences assigned to *Staphylococcus* per sample.

**Analysis of sources of variation in skin microbiota composition in wild mice** We analyzed environmental and genetic parameters, including geographic sampling location, microsatellite markers and mitochondrial D-loop sequences on the skin microbiota composition in wild-caught mice. These parameters represent proxies of the local environment, population structure and maternal transmission, respectively. To more accurately assess the effect of sampling location, we selected farms which individuals belong to distinct populations and/or haplogroups; in other words within a farm, mice that clustered into the same non-admixed population and into the same haplogroup were excluded from analysis. Additionally, the minimum sample size per farm was set at two; farms with smaller sample sizes were excluded. This curation resulted in 115 individuals spread over 23 farms and belonging to 16 populations (6 non-admixed and 10 admixed) and 5 haplogroups. In the mixed effects model, population and haplogroup (categorical variables) were included as random terms, while sampling location (as categorical variable) and host features (gender, weight, proportion of body to tail lengths, pregnancy, and body mass index) were incorporated as fixed terms. Response variables included relative abundances of major phyla and genera, alpha diversity indices, and the first three axes from principal coordinates analysis (PCoAs) of beta diversity indices. Square root- or log_10_-transformation was applied when the response variable distribution was moderately- or strongly skewed, respectively. Model selection was performed through backward elimination of non-significant (p≤0.07) fixed terms using the “step” function in “lmerTest” package (v.2.0-29) [6]. Model validation was performed by (i) checking for a normal distribution of the residuals, (ii) plotting the fitted- against the residual values, and (iii) plotting the residuals against all explanatory variables. Due to important differences in sample size between farms, and to check the sphericity assumption, the significance of the fixed terms from the best fitted models was calculated using the “Anova” function from the “car” package (v.3.0.-6) [7] using a type III Wald F test with a Kenward-Roger degrees of freedom approximation. Variance components were calculated using the “VarCorr” from the “lme4” package. Marginal R^2^, which represents the fraction of the total variance explained by the fixed terms [8], was calculated using the “r.squaredGLMM” function from the “MuMIn” package (v.1.43.13) [9].

Furthermore, we estimated the effect of geographic distance between sampling sites on community structure in the 115 individuals described above. We performed a partial Mantel test in “ecodist” package (v.1.2-9) [10] using Spearman’s correlation and 1000 permutations. We included pairwise Euclidian distances between sampling locations as the main variable, while Cavalli-Sforza distance (based on neutral microsatellite alleles) and p-distance (based on D-loop polymorphisms) were incorporated as conditional variables. Response variables included the four beta diversity measures (Bray-Curtis, Jaccard, unweighted and weighted UniFrac) in both standing (DNA) and active (RNA) communities.

**Supplementary references**

1. Evanno G, Regnaut S, Goudet J. Detecting the number of clusters of individuals using the software STRUCTURE: a simulation study. Mol Ecol. 2005;14:2611-20.

2. Jakobsson M, Rosenberg NA. CLUMPP: a cluster matching and permutation program for dealing with label switching and multimodality in analysis of population structure. Bioinformatics. 2007;23:1801-6.

3. Mason WJ, Blevins JS, Beenken K, Wibowo N, Ojha N, Smeltzer MS. Multiplex PCR protocol for the diagnosis of staphylococcal infection. J Clin Microbiol. 2001;39:3332-8.

4. Rintala H, Nevalainen A, Ronka E, Suutari M. PCR primers targeting the 16S rRNA gene for the specific detection of streptomycetes. Mol Cell Probe. 2001;15:337-47.

5. Heikens E, Fleer A, Paauw A, Florijn A, Fluit AC. Comparison of genotypic and phenotypic methods for species-level identification of clinical isolates of coagulase-negative staphylococci. J Clin Microbiol. 2005;43:2286-90.

6. Kuznetsova A, Brockhoff PB, Christensen RHB. lmerTest Package: Tests in Linear Mixed Effects Models. ‎J Stat Softw. 2017;82.

7. Fox J, Weisberg S. An {R} Companion to Applied Regression. Second Edition Thousand Oaks CA. 2011.

8. Nakagawa S, Schielzeth H, O'Hara RB. A general and simple method for obtaining R^2^ from generalized linear mixed-effects models. Methods Ecol Evol. 2013;4:133-42.

9. Barton K. MuMIn: Multi-Model Inference. R package version. 2015.

10. Goslee SC, Urban DL. The ecodist Package for Dissimilarity-based Analysis of Ecological Data. 2007 Contract No.: 7.

**Supplementary Figure legends**

**Supplementary Figure 1** Unique and shared core phyla across mouse populations, in standing- (DNA-based) (A), and active (RNA-based) (B) communities.

**Supplementary Figure 2** Unconstrained principal coordinates analysis (PCoA) of Bray-Curtis (A) and Jaccard (B) indices in mouse populations in standing (DNA-based) and active (RNA-based) communities. Goodness of fit of mouse population: Bray-Curtis axes, *R^2^*=0.61, *p*=10^-5^. Jaccard: *R^2^*=0.68, *p*=10^-5^, based on 10^5^ permutations.”+” centroid of the cluster.

**Supplementary Figure 3** Multi-dimensional scaling plot of the proximity matrix from RandomForest analyses based on core genera, in standing- (DNA-based) (A), and active (RNA-based) (B) communities.

**Supplementary Figure 4** Multi-dimensional scaling plot of the proximity matrix from RandomForest analyses based on core *Staphylococcus* ASVs, in standing- (DNA-based) (A), and active (RNA-based) (B) communities.

**Supplementary Figure 5** Distribution of *S. xylosus* / *saprophyticus* (A), *S. epidermidis* (B), and *S. hominis* (C) ASVs across mouse populations. Taxonomy is based on Sanger sequencing of genus-specific 16S rRNA gene amplicons.

**Supplementary Figure 6** Genetic clusters identified by STRUCTURE for K=13 in the wild-caught mice (n=203). Defined clusters are represented with different colors. Ancestry is denoted by individual bar plots. Individuals are sorted by sampling location, separated by black vertical lines. Neutral microsatellite loci are presented in Supplementary Table 9.

**Supplementary Figure 7** Distribution of sampling locations around Espelette in the southwest of France (n=34).

**Supplementary Table legends**

**Supplementary Table 1** Collected metadata of all mouse populations, and DNA and RNA fluorescence reading used for “Decontam”.

**Supplementary Table 2** Summary statistics of the abundance of phyla and genera, and pairwise comparison of relative abundances of major phyla and genera, and alpha diversity indices based on genera distribution Wild (n=203), HL-Lab (n=225), MPI-Lab (n=29), and C57BL/6J (n=13).

**Supplementary Table 3** Summary of indicator and Random Forest analyses based on core genera and core *Staphylococcus* ASVs.

**Supplementary Table 4** Taxonomy of *Staphylococcus* and *Streptomyces* clone- and ASVs representative sequences.

**Supplementary Table 5** Defined ASVs on the *tuf* gene sequences.

**Supplementary Table 6** Effect of host features and sampling location on major phyla and genera abundance, and diversity measures based on ASVs distribution in the standing communities (DNA) in wild mice (n=115). Significant p values (p ≤ 0.05) are indicated in bold. Un: unclassified.

**Supplementary Table 7** Effect of host features and sampling location on major phyla and genera abundance, and diversity measures based on ASVs distribution in the active communities (RNA) in wild mice (n=115). Significant p values (p ≤ 0.05) are indicated in bold. Un: unclassified.

**Supplementary Table 8** Geographic coordinates of the sampling locations (n=34).

**Supplementary Table 9** Neutral microsatellite loci in wild-caught mice (n=203).

**Supplementary Table 10** List of primers used for the *tuf* gene library.
