# Supplementary figures and images for: Assessing similarities and disparities in the skin microbiota between wild and laboratory populations of house mice
Source: ISME J. 2020 Jun 9;14(10):2367–80. doi: 10.1038/s41396-020-0690-7 (PMC7490391; doi:10.1038/s41396-020-0690-7)

**A**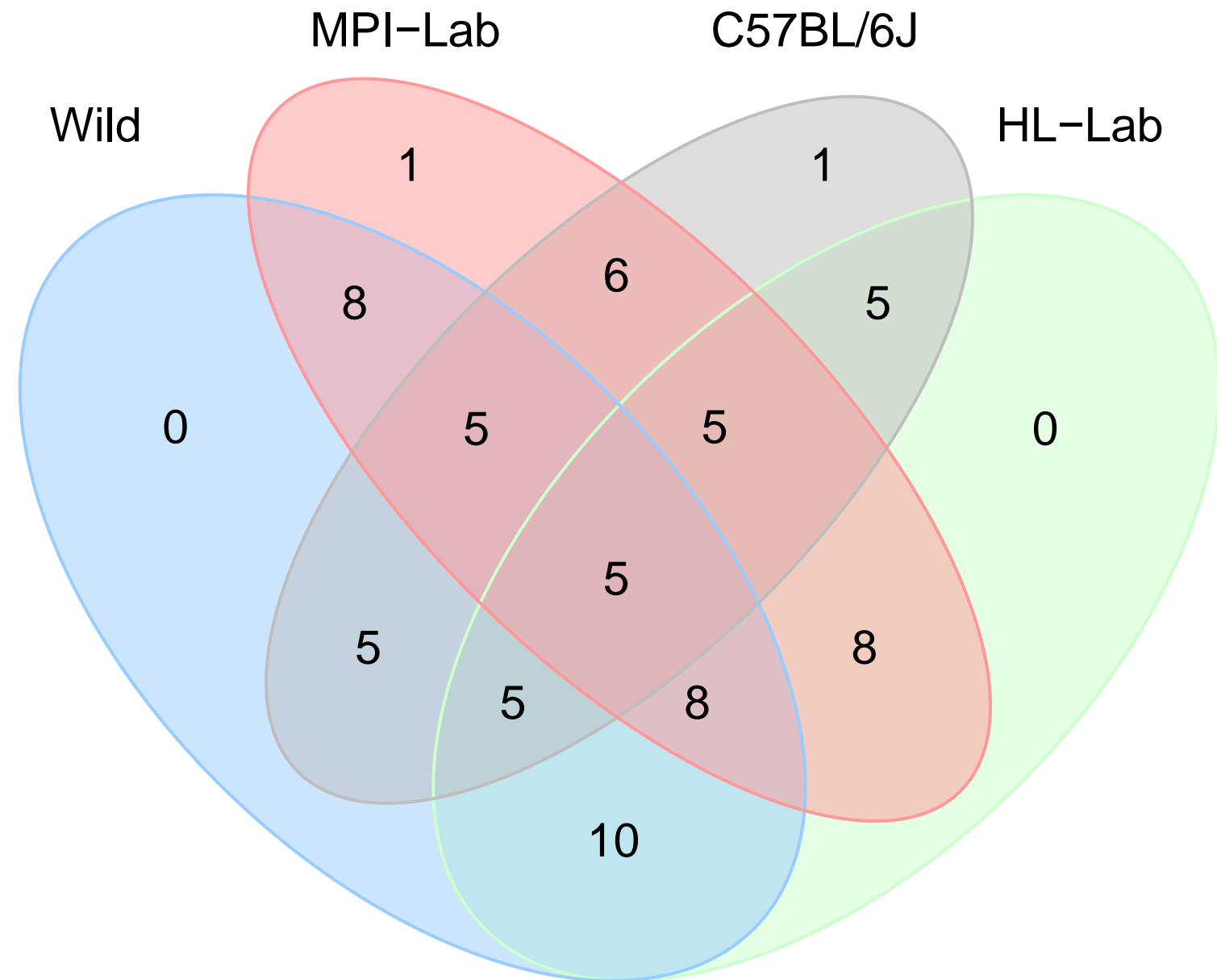**B**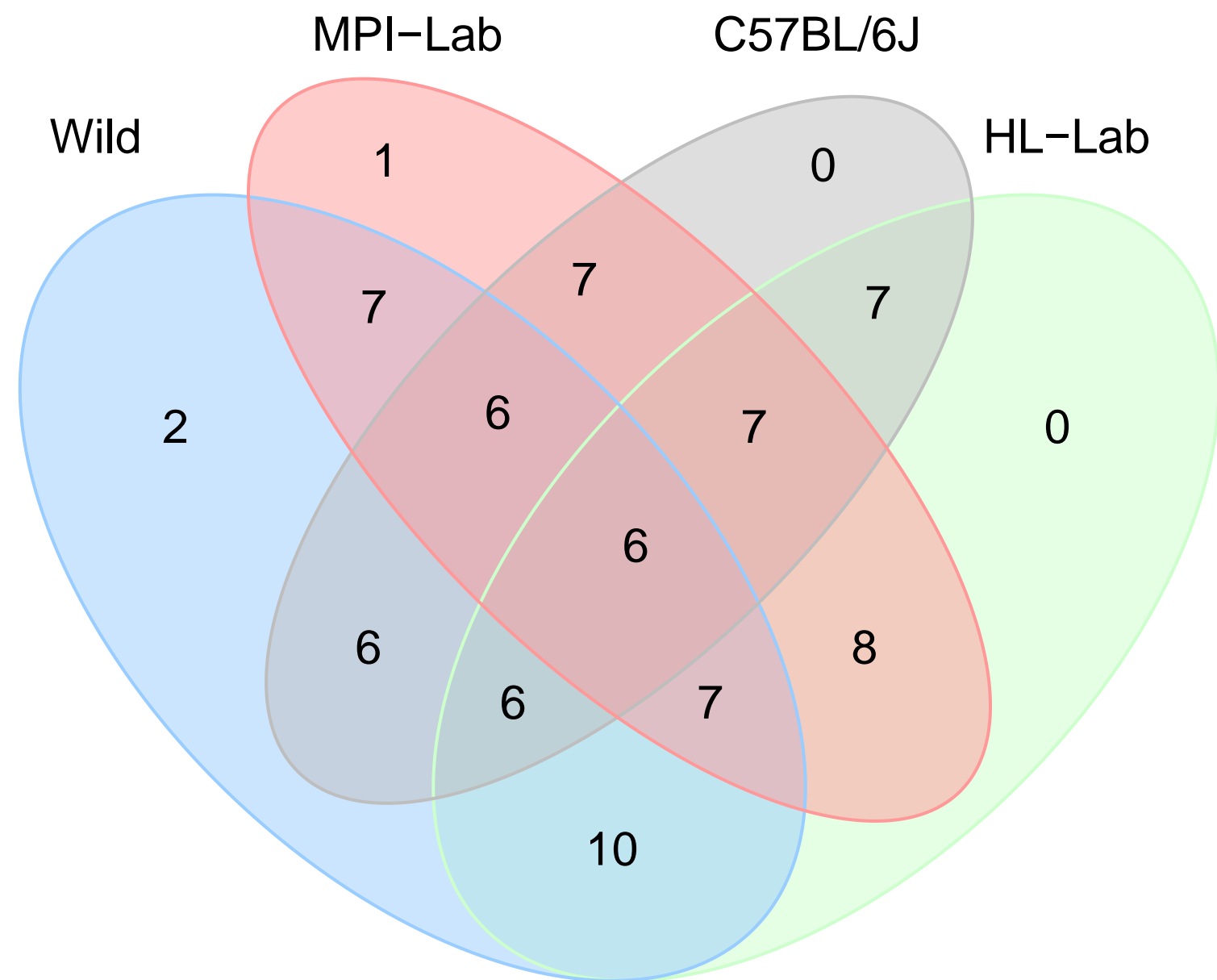

Supplement: Supplementary file 2 — Supplementary Figure 1 [file 41396_2020_690_MOESM2_ESM.pdf]

**A**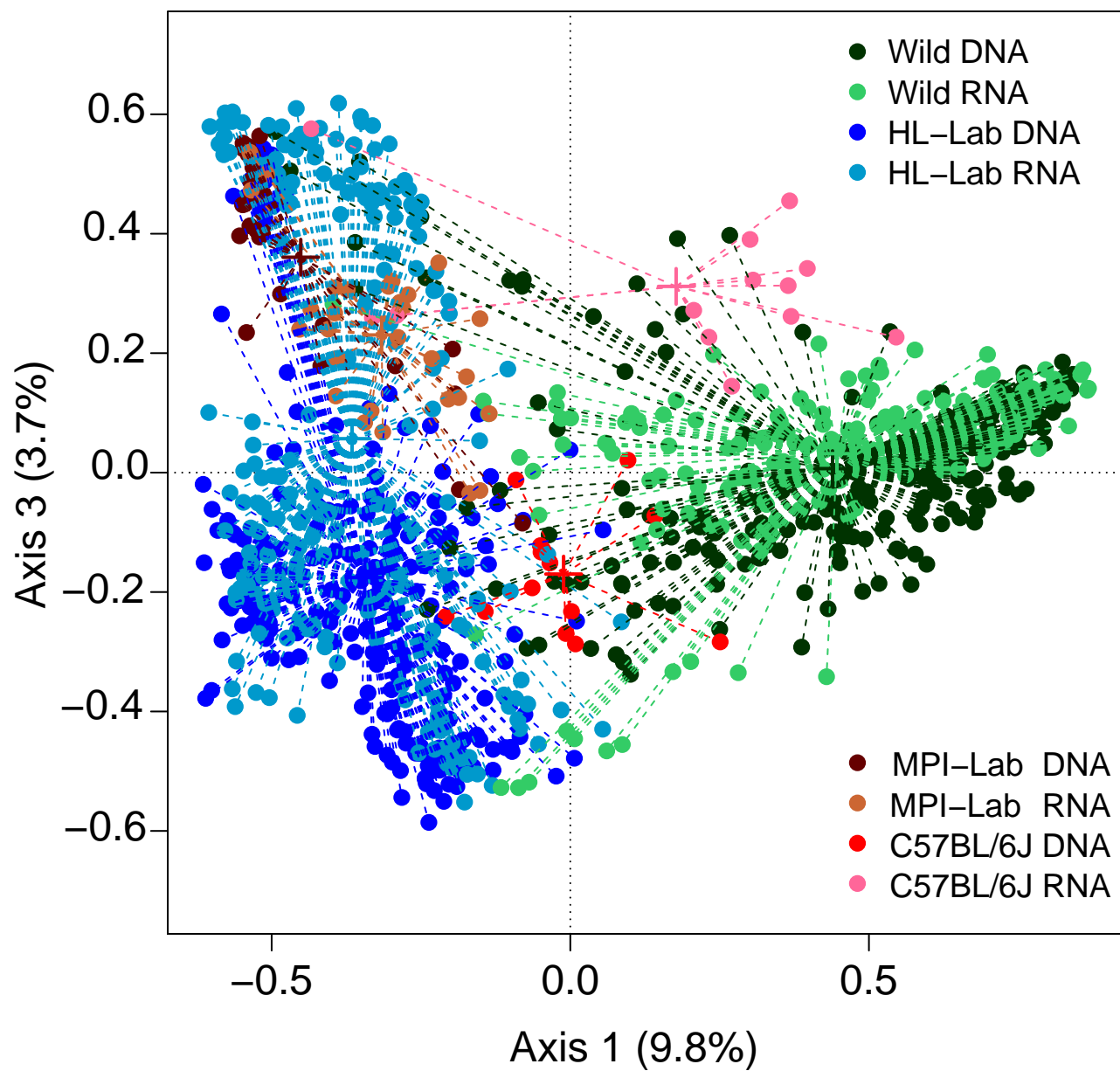**B**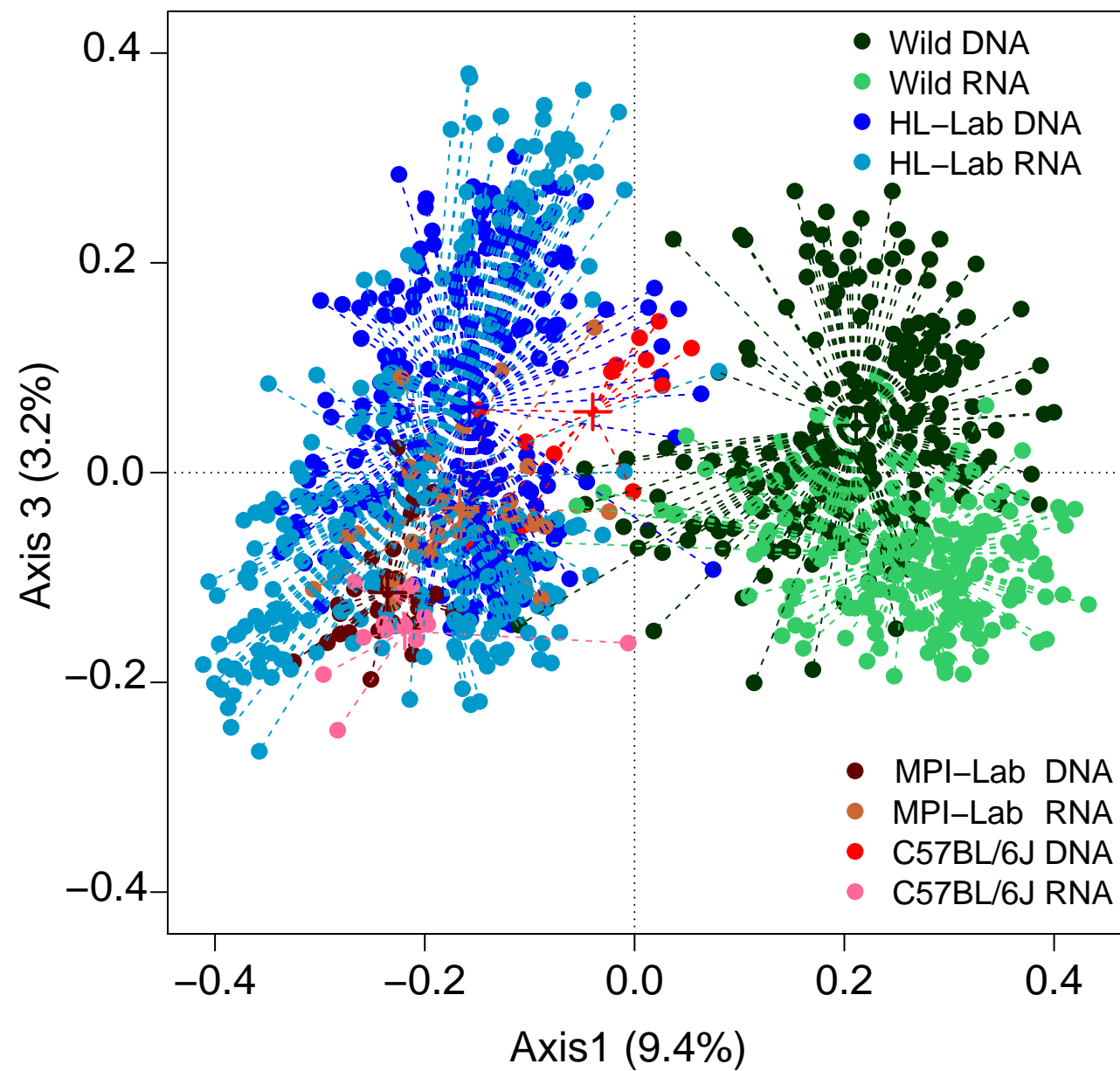

Supplement: Supplementary file 3 — Supplementary Figure 2 [file 41396_2020_690_MOESM3_ESM.pdf]

**A**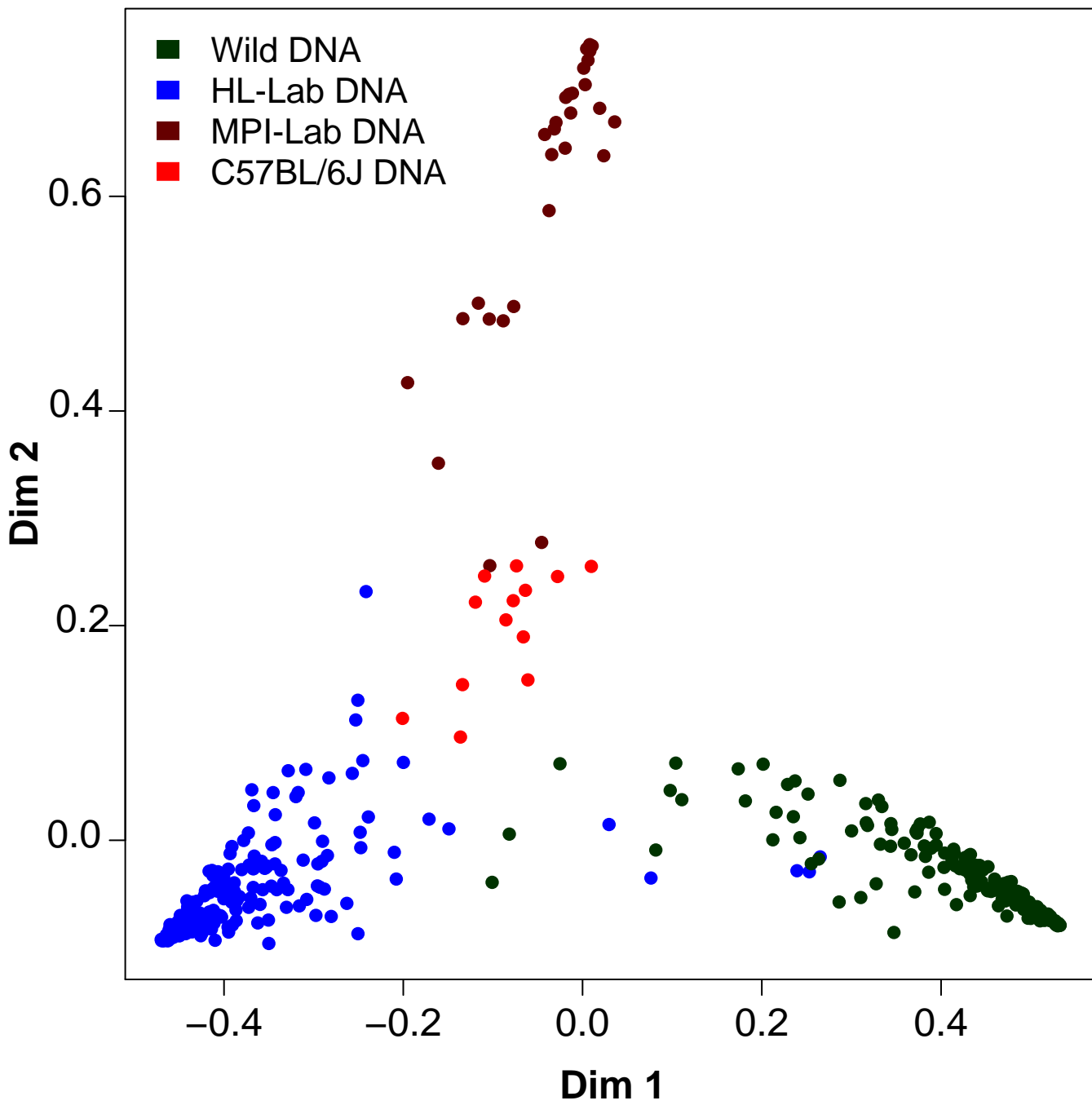**B**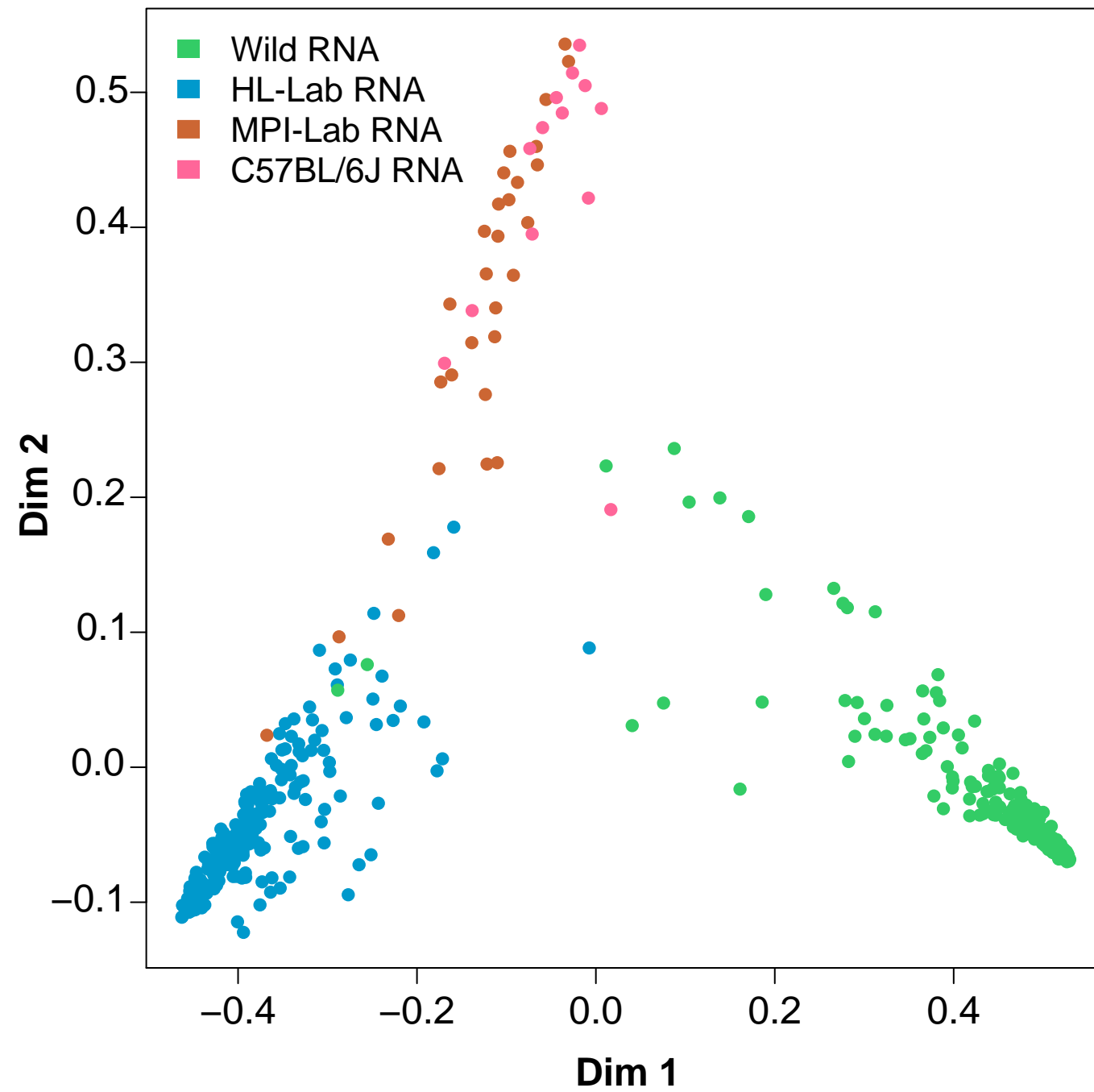

Supplement: Supplementary file 4 — Supplementary Figure 3 [file 41396_2020_690_MOESM4_ESM.pdf]

**A**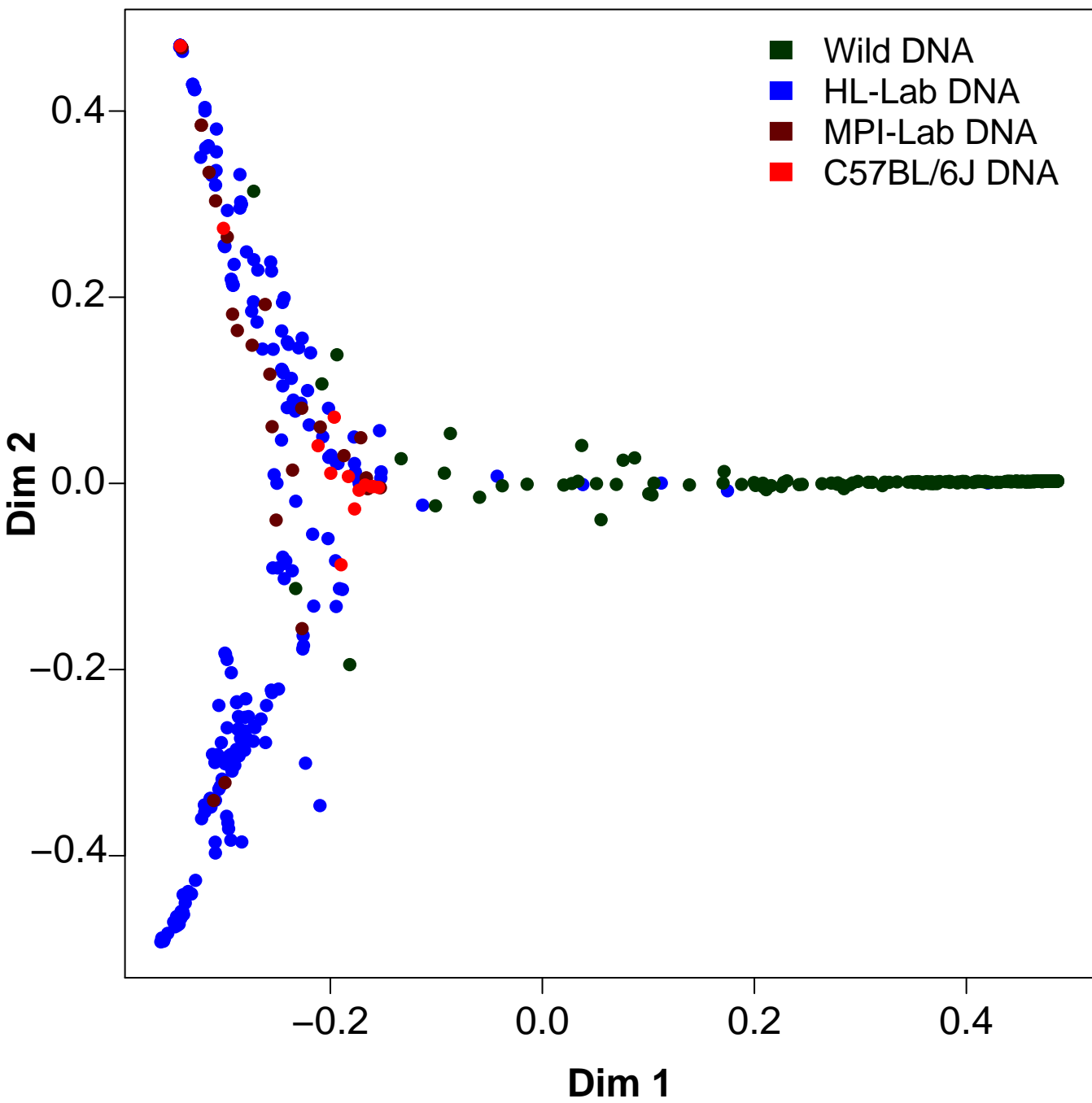**B**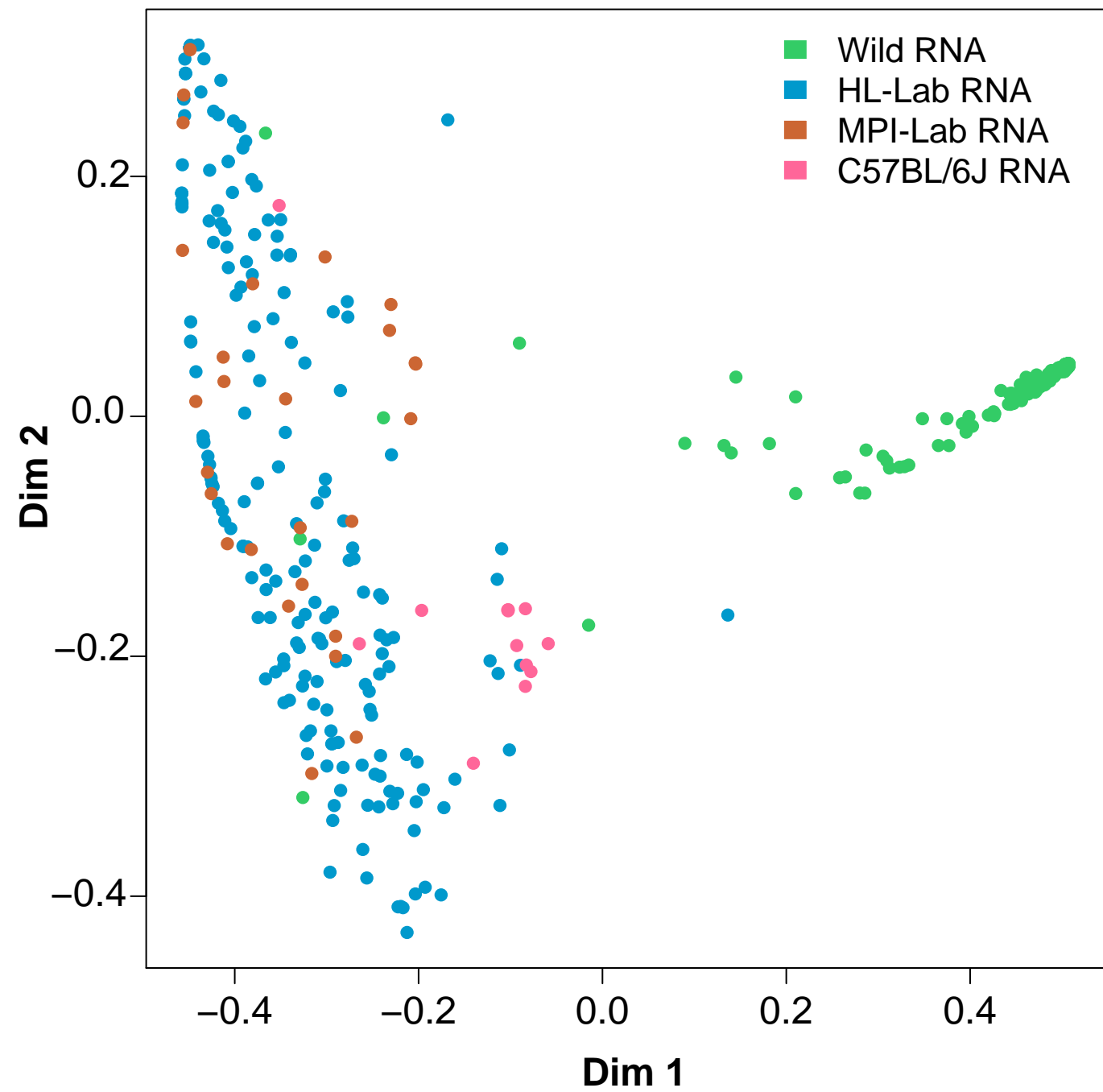

Supplement: Supplementary file 5 — Supplementary Figure 4 [file 41396_2020_690_MOESM5_ESM.pdf]

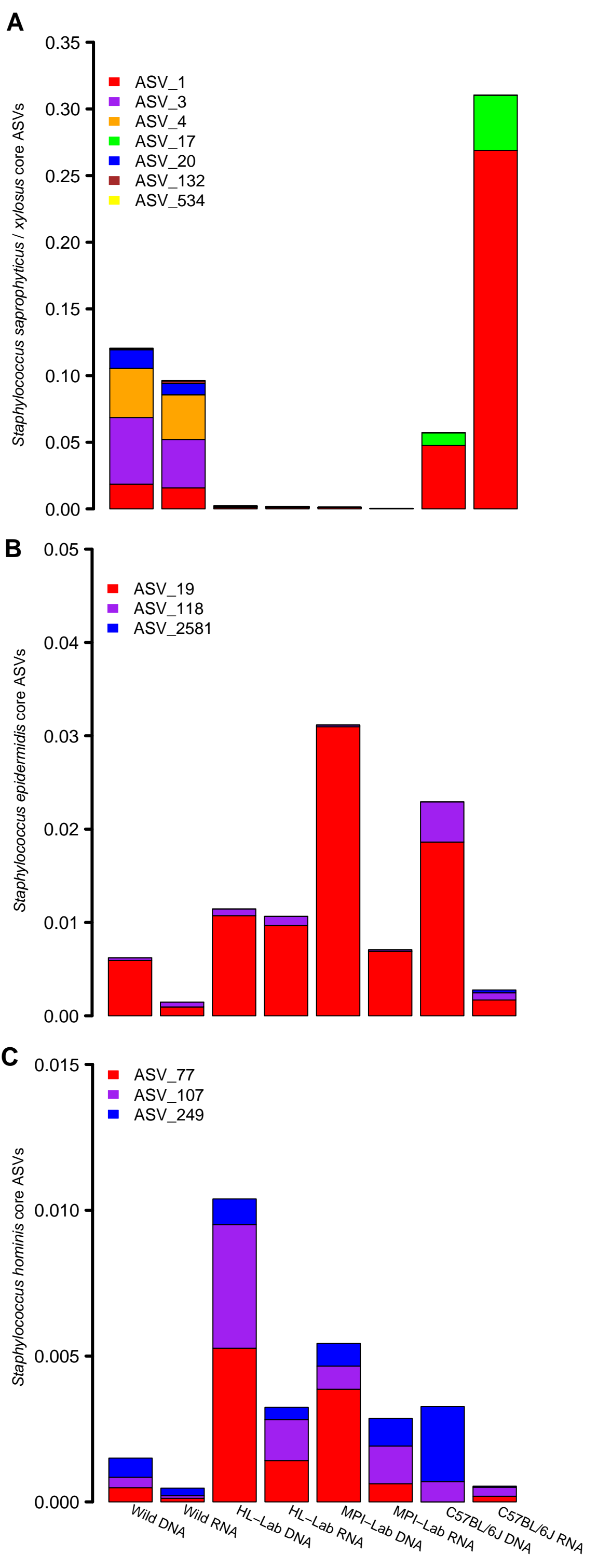

Supplement: Supplementary file 6 — Supplementary Figure 5 [file 41396_2020_690_MOESM6_ESM.pdf]

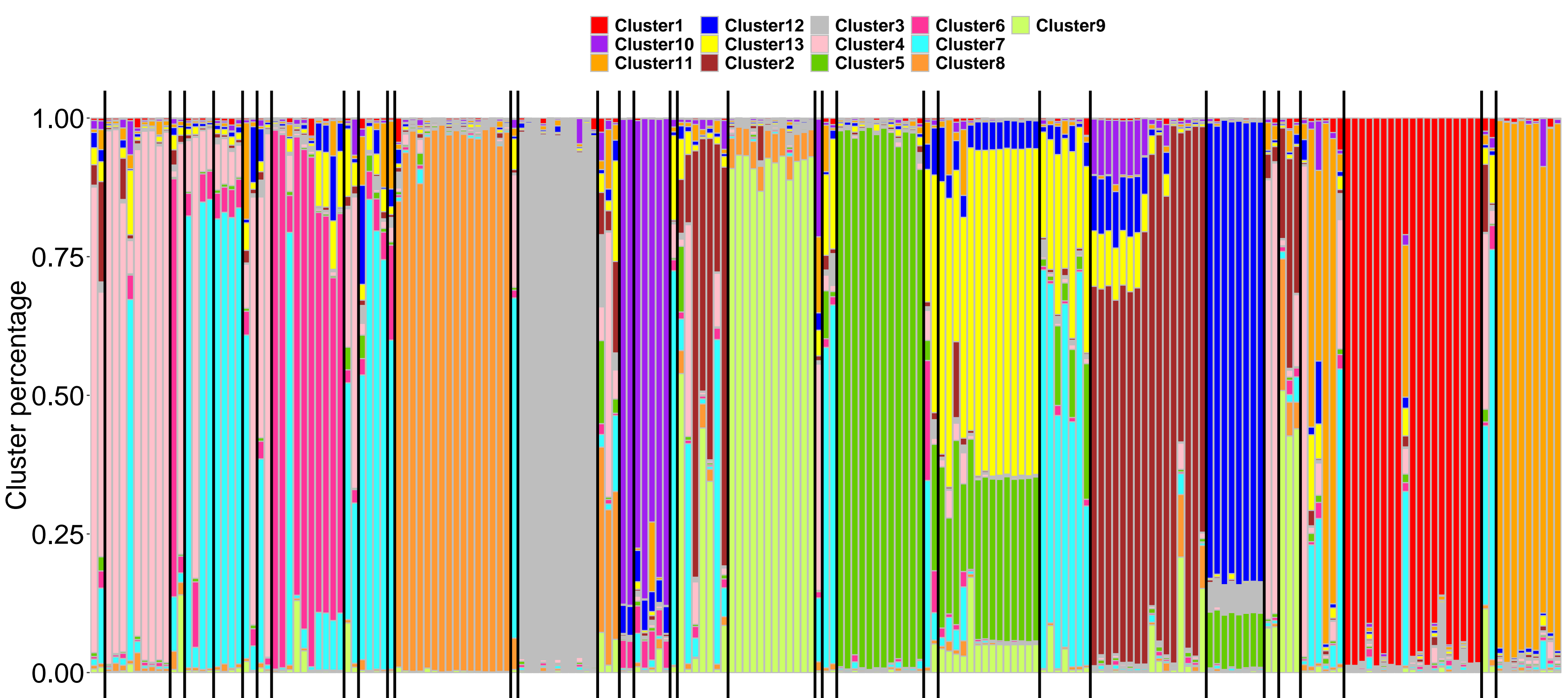

Supplement: Supplementary file 7 — Supplementary Figure 6 [file 41396_2020_690_MOESM7_ESM.pdf]

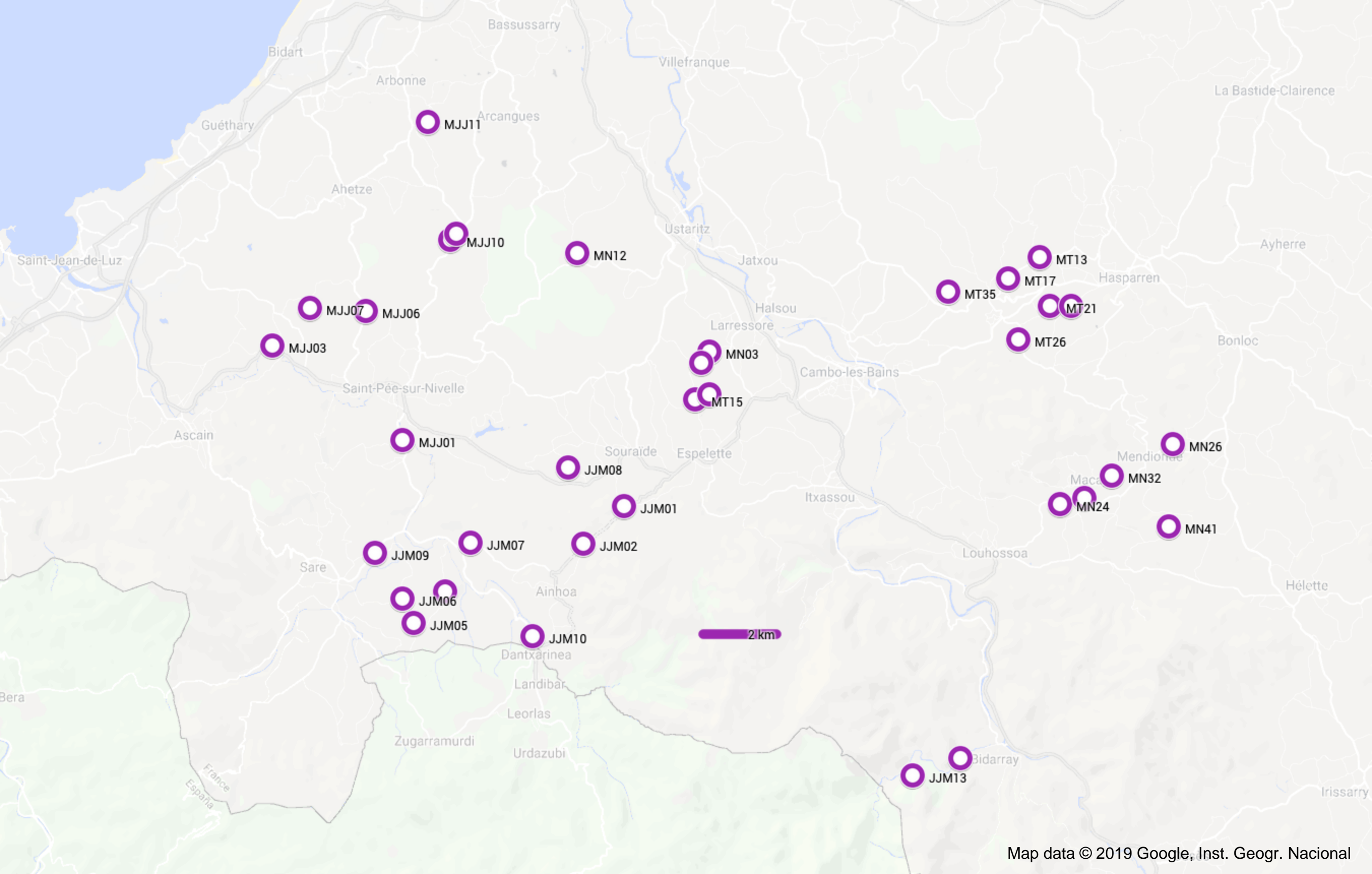

Supplement: Supplementary file 8 — Supplementary Figure 7 [file 41396_2020_690_MOESM8_ESM.pdf]
