# Supplementary material for: Assessing similarities and disparities in the skin microbiota between wild and laboratory populations of house mice
Source: ISME J. 2020 Jun 9;14(10):2367–80. doi: 10.1038/s41396-020-0690-7 (PMC7490391; doi:10.1038/s41396-020-0690-7)
Supplement: Supplementary file 9 — Supplementray Table 1 [file 41396_2020_690_MOESM9_ESM.pdf]

Supplementary Table 1.1 Collected metadata of the wild-caught mice (n=203), and ancestry analysis outputs. BMI: body mass index

Mouse_ID	Farm	Date_Of_Capture	Sex	Pregnancy status	Number of babies	Weight (g)	Total length (cm)	Tail length (cm)	Body length (cm)	Proportion of body to tail lengths	BMI	Haplotype (D-loop)	Haplogroup (D-loop)	Population (STRUCTURE)	Admixture status	Included in the statistical framework
JJM0101	JJM01	9_3_2013	female	no	0	14.5	14	7.5	6.5	0.87	3.43	ht28	hg1	A04	non-admixed	Yes
JJM0102	JJM01	9_3_2013	female	no	0	13	16	8.5	7.5	0.88	2.31	ht28	hg1	M04	admixed	Yes
JJM0202	JJM02	9_5_2013	male	not applicable	0	9	12	6	6	1	2.5	ht7	hg8	A04	non-admixed	Yes
JJM0203A	JJM02	9_5_2013	male	not applicable	0	10	14.5	7.5	7	0.93	2.04	ht21	hg4	A04	non-admixed	Yes
JJM0203B	JJM02	9_12_2013	male	not applicable	0	15.5	15	7.5	7.5	1	2.76	ht20	hg4	A04	non-admixed	Yes
JJM0204	JJM02	9_12_2013	male	not applicable	0	12	14	7	7	1	2.45	ht9	hg8	M07	admixed	Yes
JJM0206	JJM02	9_12_2013	female	no	0	10	13	7	6	0.86	2.78	ht16	hg4	A04	non-admixed	Yes
JJM0207	JJM02	9_12_2013	male	not applicable	0	11	13.5	7	6.5	0.93	2.6	ht20	hg4	A04	non-admixed	Yes
JJM0208	JJM02	9_12_2013	male	not applicable	0	12	13.5	7	6.5	0.93	2.84	ht20	hg4	A04	non-admixed	Yes
JJM0209	JJM02	9_12_2013	female	no	0	17	14	6.5	7.5	1.15	3.02	ht20	hg4	A04	non-admixed	Yes
JJM0210	JJM02	9_12_2013	male	not applicable	0	10.5	14	7.5	6.5	0.87	2.49	ht20	hg4	A04	non-admixed	Yes
JJM0401	JJM04	9_5_2013	male	not applicable	0	16	16.5	8.5	8	0.94	2.5	ht7	hg8	M06	admixed	Yes
JJM0402	JJM04	9_5_2013	female	no	0	16.5	17	9	8	0.89	2.58	ht3	hg8	M04	admixed	Yes
JJM0501	JJM05	9_5_2013	female	yes	0	15	15.5	8	7.5	0.94	2.67	ht27	hg2	A07	non-admixed	Yes
JJM0502	JJM05	9_5_2013	male	not applicable	0	13	15	8	7	0.88	2.65	ht27	hg2	A04	non-admixed	Yes
JJM0503	JJM05	9_5_2013	female	yes	5	15.5	15.5	8	7.5	0.94	2.76	ht27	hg2	A07	non-admixed	Yes
JJM0504	JJM05	9_5_2013	male	not applicable	0	7.5	12.5	6.5	6	0.92	2.08	ht27	hg2	A07	non-admixed	Yes
JJM0601	JJM06	9_7_2013	female	yes	10	29	17.5	8.5	9	1.06	3.58	ht10	hg8	A07	non-admixed	Yes
JJM0602	JJM06	9_7_2013	male	not applicable	0	13.5	15.5	8	7.5	0.94	2.4	ht17	hg4	A07	non-admixed	Yes
JJM0603	JJM06	9_7_2013	female	no	0	12.5	14	7.5	6.5	0.87	2.96	ht10	hg8	A07	non-admixed	Yes
JJM0604	JJM06	9_7_2013	male	not applicable	0	19	17	9	8	0.89	2.97	ht17	hg4	A07	non-admixed	Yes
JJM0701	JJM07	9_5_2013	male	not applicable	0	11.5	15	7.5	7.5	1	2.04	ht25	hg2	M07	admixed	Yes
JJM0702	JJM07	9_5_2013	male	not applicable	0	12	14.5	7.5	7	0.93	2.45	ht10	hg8	M04	admixed	Yes
JJM0801	JJM08	9_5_2013	female	no	0	11.5	15	8	7	0.88	2.35	ht8	hg8	M04	admixed	Yes
JJM0802	JJM08	9_5_2013	male	not applicable	0	14.5	15	7.5	7.5	1	2.58	ht15	hg4	A04	non-admixed	Yes
JJM0901	JJM09	9_7_2013	male	not applicable	0	16	16.5	8.5	8	0.94	2.5	ht7	hg8	A06	non-admixed	Yes
JJM0902	JJM09	9_7_2013	female	no	0	7.5	13	7	6	0.86	2.08	ht27	hg2	A06	non-admixed	Yes
JJM0903	JJM09	9_7_2013	male	not applicable	0	12	15	8	7	0.88	2.45	ht12	hg11	M07	admixed	Yes
JJM0905	JJM09	9_7_2013	female	no	0	10	15	8	7	0.88	2.04	ht27	hg2	A06	non-admixed	Yes
JJM0906	JJM09	9_7_2013	female	no	0	10	14.5	7.5	7	0.93	2.04	ht27	hg2	A06	non-admixed	Yes
JJM0908	JJM09	9_7_2013	male	not applicable	0	8.5	13.5	7.5	6	0.8	2.36	ht27	hg2	A06	non-admixed	Yes
JJM0909	JJM09	9_7_2013	female	no	0	6.5	12	6.5	5.5	0.85	2.15	ht26	hg2	M06	admixed	Yes
JJM0910	JJM09	9_7_2013	female	yes	8	22	17	8.5	8.5	1	3.04	ht26	hg2	M06	admixed	Yes
JJM0911	JJM09	9_7_2013	male	not applicable	0	17.5	17	8.5	8.5	1	2.42	ht13	hg11	M06	admixed	Yes
JJM0912	JJM09	9_12_2013	female	no	0	8	13	7	6	0.86	2.22	ht26	hg2	M06	admixed	Yes
JJM1001	JJM10	9_7_2013	male	not applicable	0	11	14	7	7	1	2.24	ht9	hg8	M07	admixed	Yes
JJM1002	JJM10	9_7_2013	male	not applicable	0	12.5	14.5	7.5	7	0.93	2.55	ht23	hg2	M04	admixed	Yes
JJM1201	JJM12	9_10_2013	female	no	0	6	13	7	6	0.86	1.67	ht12	hg11	M07	admixed	Yes
JJM1202	JJM12	9_10_2013	female	yes	3	12.5	14	7	7	1	2.55	ht20	hg4	A07	non-admixed	Yes
JJM1203	JJM12	9_10_2013	male	not applicable	0	16	16	8	8	1	2.5	ht15	hg4	M07	admixed	Yes
JJM1204	JJM12	9_10_2013	female	yes	0	13.5	14	7	7	1	2.76	ht11	hg8	M07	admixed	Yes
JJM1301	JJM13	9_10_2013	female	no	0	11.5	13.5	7.5	6	0.8	3.19	ht15	hg4	M07	admixed	No
MJJ0101	MJJ01	9_18_2013	male	not applicable	0	13.5	16	8	8	1	2.11	ht12	hg11	A08	non-admixed	No
MJJ0102	MJJ01	9_18_2013	male	not applicable	0	15.5	15.8	7.6	8.2	1.08	2.31	ht12	hg11	A08	non-admixed	No
MJJ0103	MJJ01	9_18_2013	male	not applicable	0	9	13.8	7	6.8	0.97	1.95	ht12	hg11	A08	non-admixed	No
MJJ0104	MJJ01	9_18_2013	male	not applicable	0	15.5	16.3	8.3	8	0.96	2.42	ht12	hg11	A08	non-admixed	No
MJJ0105	MJJ01	9_18_2013	male	not applicable	0	10	15.2	8.1	7.1	0.88	1.98	ht12	hg11	A08	non-admixed	No
MJJ0106	MJJ01	9_18_2013	male	not applicable	0	16	16.8	8.3	8.5	1.02	2.21	ht12	hg11	A08	non-admixed	No
MJJ0107	MJJ01	9_18_2013	male	not applicable	0	10	14.7	7.5	7.2	0.96	1.93	ht12	hg11	A08	non-admixed	No
MJJ0108	MJJ01	9_18_2013	male	not applicable	0	18	17.2	8.5	8.7	1.02	2.38	ht12	hg11	A08	non-admixed	No
MJJ0109	MJJ01	9_18_2013	male	not applicable	0	11	15.9	8.3	7.6	0.92	1.9	ht12	hg11	A08	non-admixed	No
MJJ0111	MJJ01	9_21_2013	male	not applicable	0	12	15.4	7.9	7.5	0.95	2.13	ht12	hg11	A08	non-admixed	No
MJJ0112	MJJ01	9_21_2013	female	no	0	10	14.8	7.6	7.2	0.95	1.93	ht12	hg11	A08	non-admixed	No
MJJ0113	MJJ01	9_21_2013	male	not applicable	0	13	15.1	7.6	7.5	0.99	2.31	ht12	hg11	A08	non-admixed	No
MJJ0114	MJJ01	9_21_2013	male	not applicable	0	12	15.1	7.6	7.5	0.99	2.13	ht12	hg11	A08	non-admixed	No
MJJ0115	MJJ01	9_21_2013	male	not applicable	0	16.5	16.2	7.8	8.4	1.08	2.34	ht12	hg11	A08	non-admixed	No
MJJ0116	MJJ01	9_21_2013	male	not applicable	0	8.5	13.2	6.8	6.4	0.94	2.08	ht12	hg11	A08	non-admixed	No
MJJ0117	MJJ01	9_21_2013	male	not applicable	0	16	16	7.8	8.2	1.05	2.38	ht12	hg11	A08	non-admixed	No
MJJ0301	MJJ03	9_18_2013	male	not applicable	0	11	14.5	7.5	7	0.93	2.24	ht13	hg11	M07	admixed	No
MJJ0601	MJJ06	9_20_2013	male	not applicable	0	8	13.3	7.3	6	0.82	2.22	ht16	hg4	A03	non-admixed	No
MJJ0602	MJJ06	9_20_2013	male	not applicable	0	13	16	8.5	7.5	0.88	2.31	ht16	hg4	A03	non-admixed	No
MJJ0603	MJJ06	9_20_2013	male	not applicable	0	13	15.3	7.9	7.4	0.94	2.37	ht16	hg4	A03	non-admixed	No
MJJ0604	MJJ06	9_20_2013	male	not applicable	0	13	16.3	8.3	8	0.96	2.03	ht16	hg4	A03	non-admixed	No
MJJ0605	MJJ06	9_20_2013	male	not applicable	0	12	15.7	8.3	7.4	0.89	2.19	ht16	hg4	A03	non-admixed	No
MJJ0606	MJJ06	9_21_2013	female	no	0	10	14.5	7.5	7	0.93	2.04	ht16	hg4	A03	non-admixed	No
MJJ0607	MJJ06	9_21_2013	male	not applicable	0	17	17	8.7	8.3	0.95	2.47	ht16	hg4	A03	non-admixed	No
MJJ0608	MJJ06	9_21_2013	male	not applicable	0	8.5	14.4	7.9	6.5	0.82	2.01	ht16	hg4	A03	non-admixed	No
MJJ0609	MJJ06	9_21_2013	male	not applicable	0	14	15.8	7.6	8.2	1.08	2.08	ht16	hg4	A03	non-admixed	No
MJJ0610	MJJ06	9_21_2013	male	not applicable	0	15.5	17.5	9	8.5	0.94	2.15	ht16	hg4	A03	non-admixed	No
MJJ0611	MJJ06	9_21_2013	male	not applicable	0	15	16.6	8.6	8	0.93	2.34	ht16	hg4	A03	non-admixed	No
MJJ0701	MJJ07	9_20_2013	male	not applicable	0	9.5	14	7.2	6.8	0.94	2.05	ht7	hg8	M08	admixed	No
MJJ0702	MJJ07	9_21_2013	male	not applicable	0	14	14.9	7.4	7.5	1.01	2.49	ht7	hg8	M04	admixed	Yes
MJJ0703	MJJ07	9_21_2013	male	not applicable	0	13.5	16	8	8	1	2.11	ht7	hg8	M08	admixed	Yes
MJJ0901	MJJ09	9_24_2013	female	no	0	11.5	16	8.5	7.5	0.88	2.04	ht20	hg4	A10	non-admixed	No
MJJ0902	MJJ09	9_24_2013	male	not applicable	0	12	15.1	7.8	7.3	0.94	2.25	ht20	hg4	A10	non-admixed	No
MJJ1001	MJJ10	9_25_2013	male	not applicable	0	13.5	15.4	7.4	8	1.08	2.11	ht19	hg4	M10	admixed	Yes
MJJ1002	MJJ10	9_25_2013	female	yes	7	19	17.2	8.3	8.9	1.07	2.4	ht20	hg4	A10	non-admixed	Yes
MJJ1003	MJJ10	9_25_2013	male	not applicable	0	10	14.7	7.5	7.2	0.96	1.93	ht20	hg4	M10	admixed	No
MJJ1004	MJJ10	9_25_2013	male	not applicable	0	14	16.1	8.3	7.8	0.94	2.3	ht20	hg4	A10	non-admixed	No
MJJ1005	MJJ10	9_26_2013	female	yes	0	14	16.3	8.4	7.9	0.94	2.24	ht20	hg4	A10	non-admixed	No
MJJ1101	MJJ11	9_25_2013	female	no	0	12	15.2	7.6	7.6	1	2.08	ht4	hg8	M07	admixed	No
MN0201	MN02	9_18_2013	male	not applicable	0	13.5	16.3	8	8.3	1.04	1.96	ht20	hg4	M09	admixed	yes
MN0202	MN02	9_18_2013	male	not applicable	0	14	16.4	8.4	8	0.95	2.19	ht5	hg8	M07	admixed	Yes
MN0203	MN02	9_18_2013	male	not applicable	0	15	16.7	8.3	8.4	1.01	2.13	ht6	hg8	M02	admixed	Yes
MN0204	MN02	9_18_2013	male	not applicable	0	7	13	7	6	0.86	1.94	ht5	hg8	M02	admixed	Yes
MN0205	MN02	9_21_2013	male	not applicable	0	13	15.1	7.6	7.5	0.99	2.31	ht6	hg8	M02	admixed	Yes
MN0206	MN02	9_21_2013	male	not applicable	0	13	15.3	7.8	7.5	0.96	2.31	ht5	hg8	M07	admixed	Yes
MN0207	MN02	9_21_2013	male	not applicable	0	17	16.2	8.1	8.1	1	2.59	ht5	hg8	M02	admixed	Yes

MN0301	MN03	9_18_2013	male	not applicable	0	15	15.7	7.7	8	1.04	2.34	ht5	hg8	A09	non-admixed	No
MN0302	MN03	9_18_2013	male	not applicable	0	8	12.9	6.9	6	0.87	2.22	ht5	hg8	A09	non-admixed	No
MN0303	MN03	9_18_2013	male	not applicable	0	18.5	17.5	8.5	9	1.06	2.28	ht5	hg8	A09	non-admixed	No
MN0304	MN03	9_18_2013	male	not applicable	0	9	14.1	7.1	7	0.99	1.84	ht5	hg8	A09	non-admixed	No
MN0305	MN03	9_21_2013	male	not applicable	0	16.5	16	7.8	8.2	1.05	2.45	ht5	hg8	A09	non-admixed	No
MN0306	MN03	9_21_2013	male	not applicable	0	20	16.8	8.1	8.7	1.07	2.64	ht5	hg8	A09	non-admixed	No
MN0307	MN03	9_21_2013	male	not applicable	0	18	18	8.9	9.1	1.02	2.17	ht5	hg8	A09	non-admixed	No
MN0308	MN03	9_21_2013	male	not applicable	0	6.5	11.8	5.9	5.9	1	1.87	ht5	hg8	A09	non-admixed	No
MN0309	MN03	9_21_2013	male	not applicable	0	9	13.7	6.8	6.9	1.01	1.89	ht5	hg8	A09	non-admixed	No
MN0310	MN03	9_21_2013	male	not applicable	0	15.5	15.2	7.2	8	1.11	2.42	ht5	hg8	A09	non-admixed	No
MN0311	MN03	9_21_2013	male	not applicable	0	16	15.1	7.6	7.5	0.99	2.84	ht5	hg8	A09	non-admixed	No
MN0312	MN03	9_21_2013	female	yes	5	19	15.9	7.9	8	1.01	2.97	ht5	hg8	A09	non-admixed	No
MN1202	MN12	9_20_2013	female	yes	5	23	16.4	7.4	9	1.22	2.84	ht20	hg4	M04	admixed	No
MN2401	MN24	9_24_2013	male	not applicable	0	17.5	16	8	8	1	2.73	ht20	hg4	M04	admixed	Yes
MN2402	MN24	9_24_2013	male	not applicable	0	6	11.9	6.2	5.7	0.92	1.85	ht2	hg8	M07	admixed	Yes
MN2601	MN26	9_24_2013	female	no	0	8	13	7.1	5.9	0.83	2.3	ht24	hg2	A05	non-admixed	No
MN2602	MN26	9_24_2013	male	not applicable	0	14	15.5	7.8	7.7	0.99	2.36	ht24	hg2	A05	non-admixed	No
MN2603	MN26	9_24_2013	female	yes	7	22	17.6	8.5	9.1	1.07	2.66	ht24	hg2	A05	non-admixed	No
MN2605	MN26	9_24_2013	male	not applicable	0	16	16.5	8.8	7.7	0.88	2.7	ht15	hg4	A05	non-admixed	No
MN2606	MN26	9_24_2013	male	not applicable	0	17	16.6	8.1	8.5	1.05	2.35	ht18	hg4	A05	non-admixed	No
MN2608	MN26	9_25_2013	female	yes	0	14.5	16.5	8.4	8.1	0.96	2.21	ht15	hg4	A05	non-admixed	No
MN2609	MN26	9_25_2013	female	no	0	14.5	16.4	8.4	8	0.95	2.27	ht15	hg4	A05	non-admixed	No
MN2610	MN26	9_25_2013	female	no	0	8.5	13.2	6.8	6.4	0.94	2.08	ht24	hg2	A05	non-admixed	No
MN2611	MN26	9_25_2013	male	not applicable	0	16.5	15.5	7.5	8	1.07	2.58	ht18	hg4	A05	non-admixed	No
MN2613	MN26	9_26_2013	female	no	0	7.5	13.3	7.1	6.2	0.87	1.95	ht15	hg4	A05	non-admixed	No
MN2614	MN26	9_26_2013	female	no	0	14	15.8	8.1	7.7	0.95	2.36	ht15	hg4	A05	non-admixed	No
MN2616	MN26	9_26_2013	male	not applicable	0	15	15	7.3	7.7	1.05	2.53	ht18	hg4	A05	non-admixed	No
MN2901	MN29	9_25_2013	male	not applicable	0	16.5	16.9	8.7	8.2	0.94	2.45	ht14	hg4	M07	admixed	Yes
MN2902	MN29	9_25_2013	female	no	0	12	15.6	7.7	7.9	1.03	1.92	ht20	hg4	M13	admixed	Yes
MN3201	MN32	9_25_2013	female	no	0	12	15.4	7.6	7.8	1.03	1.97	ht22	hg2	M13	admixed	Yes
MN3202	MN32	9_25_2013	male	not applicable	0	14.5	15.2	7.7	7.5	0.97	2.58	ht22	hg2	M13	admixed	Yes
MN3203	MN32	9_25_2013	female	no	0	8.5	13.8	7.3	6.5	0.89	2.01	ht22	hg2	M05	admixed	Yes
MN3204	MN32	9_25_2013	female	yes	0	11.5	13.7	6.7	7	1.04	2.35	ht22	hg2	M13	admixed	Yes
MN3205	MN32	9_25_2013	female	yes	0	10.5	13.8	6.7	7.1	1.06	2.08	ht12	hg11	M13	admixed	Yes
MN3206	MN32	9_25_2013	female	no	0	9	13.3	6.4	6.9	1.08	1.89	ht22	hg2	M13	admixed	Yes
MN3207	MN32	9_25_2013	female	yes	3	18.5	15.7	7.6	8.1	1.07	2.82	ht22	hg2	M13	admixed	Yes
MN3208	MN32	9_26_2013	female	no	0	9	12.7	6.2	6.5	1.05	2.13	ht12	hg11	M13	admixed	Yes
MN3209	MN32	9_26_2013	male	not applicable	0	15.5	15.4	7.4	8	1.08	2.42	ht12	hg11	M13	admixed	Yes
MN3210	MN32	9_26_2013	male	not applicable	0	15	15.4	7.2	8.2	1.14	2.23	ht12	hg11	M13	admixed	Yes
MN3211	MN32	9_26_2013	female	no	0	5.5	11.8	6.1	5.7	0.93	1.69	ht22	hg2	M13	admixed	Yes
MN3212	MN32	9_26_2013	male	not applicable	0	10	13.9	6.9	7	1.01	2.04	ht12	hg11	M13	admixed	Yes
MN3214	MN32	9_26_2013	female	yes	8	25.5	15.8	7.2	8.6	1.19	3.45	ht12	hg11	M13	admixed	Yes
MN3215	MN32	9_26_2013	male	not applicable	0	10.5	13.8	6.8	7	1.03	2.14	ht22	hg2	M13	admixed	Yes
MN4101	MN41	9_26_2013	male	not applicable	0	15	15.5	7.9	7.6	0.96	2.6	ht15	hg4	M07	admixed	Yes
MN4102	MN41	9_26_2013	female	no	0	10	14	7.1	6.9	0.97	2.1	ht22	hg2	M07	admixed	Yes
MN4103	MN41	9_26_2013	female	yes	6	19	15.3	7.3	8	1.1	2.97	ht22	hg2	M07	admixed	Yes
MN4104	MN41	9_26_2013	male	not applicable	0	15	16.8	8.6	8.2	0.95	2.23	ht22	hg2	M07	admixed	Yes
MN4105	MN41	9_26_2013	male	not applicable	0	15	15	7.3	7.7	1.05	2.53	ht22	hg2	M07	admixed	Yes
MN4106	MN41	9_26_2013	male	not applicable	0	17.5	17.2	8.8	8.4	0.95	2.48	ht15	hg4	M07	admixed	Yes
MN4107	MN41	9_26_2013	female	yes	6	17	15.3	7.3	8	1.1	2.66	ht22	hg2	M13	admixed	Yes
MT0101	MT01	9_7_2013	male	not applicable	0	10	8	1	7	7	2.04	ht6	hg8	M02	admixed	Yes
MT0102	MT01	9_7_2013	male	not applicable	0	20	16.5	8.5	8	0.94	3.13	ht6	hg8	M02	admixed	Yes
MT0103	MT01	9_7_2013	female	yes	7	22	17	8.5	8.5	1	3.04	ht6	hg8	M02	admixed	Yes
MT0104	MT01	9_12_2013	male	not applicable	0	20	17	8.5	8.5	1	2.77	ht6	hg8	M02	admixed	Yes
MT0105	MT01	9_12_2013	female	no	0	13	15	8	7	0.88	2.65	ht6	hg8	M02	admixed	Yes
MT0106	MT01	9_12_2013	female	no	0	6	6	1	5	5	2.4	ht6	hg8	M02	admixed	Yes
MT0107	MT01	9_12_2013	female	no	0	10	13.5	7	6.5	0.93	2.37	ht6	hg8	M02	admixed	Yes
MT0108	MT01	9_12_2013	male	not applicable	0	6.5	12	6.5	5.5	0.85	2.15	ht6	hg8	M02	admixed	Yes
MT0109	MT01	9_12_2013	female	yes	0	20	17	8.5	8.5	1	2.77	ht5	hg8	A02	admixed	Yes
MT0110	MT01	9_12_2013	female	yes	0	14	15.5	8.5	7	0.82	2.86	ht5	hg8	A02	non-admixed	Yes
MT0111	MT01	9_12_2013	male	not applicable	0	8	13	7	6	0.86	2.22	ht5	hg8	A02	non-admixed	Yes
MT0112	MT01	9_12_2013	female	yes	10	25	16.5	8.5	8	0.94	3.91	ht5	hg8	A02	non-admixed	Yes
MT0113	MT01	9_12_2013	male	not applicable	0	17	16	8	8	1	2.66	ht5	hg8	M02	admixed	Yes
MT0114	MT01	9_12_2013	female	yes	0	14	15.5	8	7.5	0.94	2.49	ht5	hg8	A02	non-admixed	Yes
MT0115	MT01	9_12_2013	female	no	0	15	15.5	8.5	7	0.82	3.06	ht5	hg8	A02	non-admixed	Yes
MT0116	MT01	9_12_2013	female	no	0	5.5	11	6	5	0.83	2.2	ht5	hg8	M02	non-admixed	Yes
MT1301	MT13	9_5_2013	female	no	0	6.5	11.5	6	5.5	0.92	2.15	ht1	hg8	A12	non-admixed	No
MT1302	MT13	9_5_2013	male	not applicable	0	14.5	15	7.5	7.5	1	2.58	ht1	hg8	A12	non-admixed	No
MT1303	MT13	9_5_2013	male	not applicable	0	8.5	13	6.5	6.5	1	2.01	ht1	hg8	A12	non-admixed	No
MT1304	MT13	9_6_2013	female	no	0	16	16	8	8	1	2.5	ht1	hg8	A12	non-admixed	No
MT1305	MT13	9_30_2013	female	yes	5	11	14.8	7.3	7.5	1.03	1.96	ht1	hg8	A12	non-admixed	No
MT1306	MT13	9_30_2013	female	no	0	17	16.4	7.9	8.5	1.08	2.35	ht1	hg8	A12	non-admixed	No
MT1307	MT13	9_30_2013	male	not applicable	0	9	11	5.5	5.5	1	2.98	ht1	hg8	A12	non-admixed	No
MT1308	MT13	9_30_2013	male	not applicable	0	14	16	8	8	1	2.19	ht1	hg8	A12	non-admixed	No
MT1401	MT14	9_5_2013	male	not applicable	0	12	15	8	7	0.88	2.45	ht22	hg2	M04	admixed	Yes
MT1402	MT14	9_6_2013	male	not applicable	0	15	15.5	8	7.5	0.94	2.67	ht22	hg2	A04	admixed	Yes
MT1501	MT15	9_7_2013	male	not applicable	0	15	15	7.5	7.5	1	2.67	ht5	hg8	M09	admixed	Yes
MT1502	MT15	9_29_2013	female	yes	0	14	15.5	7.5	8	1.07	2.19	ht5	hg8	M09	admixed	Yes
MT1503	MT15	9_29_2013	male	not applicable	0	13	14.5	7.5	7	0.93	2.65	ht5	hg8	M09	admixed	Yes
MT1701	MT17	9_7_2013	female	no	0	11	14	7	7	1	2.24	ht1	hg8	A04	non-admixed	Yes
MT1702	MT17	9_7_2013	female	no	0	17	17	9	8	0.89	2.66	ht1	hg8	M11	admixed	Yes
MT1704	MT17	9_7_2013	female	no	0	16	15.5	8	7.5	0.94	2.84	ht1	hg8	M11	admixed	Yes
MT1705	MT17	9_7_2013	male	not applicable	0	12	12	6	6	1	3.33	ht12	hg11	A11	non-admixed	Yes
MT1706	MT17	9_29_2013	male	not applicable	0	15	16.5	8.5	8	0.94	2.34	ht12	hg8	A11	admixed	Yes
MT1707	MT17	9_29_2013	female	no	0	10	14.5	7.5	7	0.93	2.04	ht12	hg11	M07	admixed	Yes
MT2101	MT21	9_7_2013	male	not applicable	0	19	15	7.5	7.5	1	3.38	ht12	hg11	A01	non-admixed	No
MT2102	MT21	9_7_2013	female	no	0	17	15.5	8	7.5	0.94	3.02	ht12	hg11	A01	non-admixed	No
MT2103	MT21	9_7_2013	male	not applicable	0	11	14.5	7.5	7	0.93	2.24	ht12	hg11	A01	non-admixed	No
MT2104	MT21	9_7_2013	female	no	0	18.5	14	6	8	1.33	2.89	ht12	hg11	A01	non-admixed	No
MT2105	MT21	9_7_2013	female	no	0	16	16	8	8	1	2.5	ht1	hg8	A01	non-admixed	No
MT2106	MT21	9_7_2013	male	not applicable	0	14	15.5	8	7.5	0.94	2.49	ht12	hg11	A01	non-admixed	No

MT2107	MT21	9_7_2013	female	no	0	17	16	8	8	1	2.66	ht12	hg11	A01	non-admixed	No
MT2108	MT21	9_7_2013	female	yes	0	16.5	15	8	7	0.88	3.37	ht1	hg8	A01	non-admixed	No
MT2109	MT21	9_7_2013	male	not applicable	0	18	16	8	8	1	2.81	ht22	hg2	M07	admixed	No
MT2110	MT21	9_10_2013	male	not applicable	0	14	15.5	8	7.5	0.94	2.49	ht12	hg11	A01	non-admixed	No
MT2112	MT21	9_10_2013	male	not applicable	0	17	13.5	5.5	8	1.45	2.66	ht12	hg11	A01	non-admixed	No
MT2113	MT21	9_10_2013	female	no	0	8	12	6.5	5.5	0.85	2.64	ht12	hg11	A01	non-admixed	No
MT2114	MT21	9_10_2013	female	yes	7	21	16	8	8	1	3.28	ht12	hg11	A01	non-admixed	No
MT2115	MT21	9_10_2013	female	yes	1	19	16	8	8	1	2.97	ht12	hg11	A01	non-admixed	No
MT2116	MT21	9_10_2013	female	yes	0	23	18	9	9	1	2.84	ht12	hg11	A01	non-admixed	No
MT2117	MT21	9_10_2013	female	no	0	14.5	15	7.5	7.5	1	2.58	ht12	hg11	A01	non-admixed	No
MT2118	MT21	9_10_2013	male	not applicable	0	16	15	7.5	7.5	1	2.84	ht12	hg11	A01	non-admixed	No
MT2120	MT21	9_10_2013	female	yes	7	18	15.5	7.5	8	1.07	2.81	ht1	hg8	A01	non-admixed	No
MT2121	MT21	9_10_2013	male	not applicable	0	15	15	7.5	7.5	1	2.67	ht12	hg11	A01	non-admixed	No
MT2601	MT26	9_7_2013	female	no	0	8.5	13.5	7	6.5	0.93	2.01	ht22	hg2	M07	admixed	Yes
MT2603	MT26	9_7_2013	male	not applicable	0	16	15	7.5	7.5	1	2.84	ht12	hg11	M07	admixed	Yes
MT3501	MT35	9_12_2013	male	not applicable	0	15	14	7	7	1	3.06	ht12	hg11	A11	non-admixed	Yes
MT3502	MT35	9_12_2013	male	not applicable	0	15	15	7.5	7.5	1	2.67	ht1	hg8	A11	non-admixed	Yes
MT3503	MT35	9_12_2013	male	not applicable	0	17	15	8	7	0.88	3.47	ht1	hg8	A11	non-admixed	Yes
MT3506	MT35	9_12_2013	female	no	0	23	17.5	9	8.5	0.94	3.18	ht12	hg11	A11	non-admixed	Yes
MT3507	MT35	9_12_2013	male	not applicable	0	16	15	7.5	7.5	1	2.84	ht1	hg8	A11	non-admixed	Yes
MT3508	MT35	9_12_2013	female	yes	7	18	16	8	8	1	2.81	ht1	hg8	A11	non-admixed	Yes
MT3509	MT35	9_12_2013	female	yes	12	30	17.5	8.5	9	1.06	3.7	ht1	hg8	A11	non-admixed	Yes
MT3511	MT35	9_29_2013	female	no	0	19	16.8	8.3	8.5	1.02	2.63	ht12	hg11	A11	non-admixed	Yes
MT3513	MT35	9_29_2013	male	not applicable	0	11	14.3	7.5	6.8	0.91	2.38	ht12	hg8	A11	non-admixed	Yes

Supplementary Table 1.2 Metadata of HL-Lab population (n=225)

Mouse ID	Sex	Age (in days)	Family	Breeding cage	Breeding scheme	Breeding facility
8858	female	174	FAM1	C17499	outbred	University of Luebeck, Germany
8862	female	195	FAM1	C17499	outbred	University of Luebeck, Germany
8872	female	203	FAM2	C17503	outbred	University of Luebeck, Germany
8874	female	195	FAM2	C17503	outbred	University of Luebeck, Germany
8883	female	169	FAM2	C17503	outbred	University of Luebeck, Germany
8884	male	162	FAM2	C17503	outbred	University of Luebeck, Germany
8885	male	196	FAM2	C17503	outbred	University of Luebeck, Germany
8888	female	198	FAM3	C17508	outbred	University of Luebeck, Germany
8889	female	170	FAM3	C17508	outbred	University of Luebeck, Germany
8894	female	170	FAM3	C17508	outbred	University of Luebeck, Germany
8901	male	194	FAM3	C17508	outbred	University of Luebeck, Germany
8902	male	160	FAM3	C17508	outbred	University of Luebeck, Germany
8906	female	165	FAM4	C17513	outbred	University of Luebeck, Germany
8907	female	193	FAM4	C17513	outbred	University of Luebeck, Germany
8910	male	160	FAM4	C17513	outbred	University of Luebeck, Germany
8913	female	172	FAM5	C17534	outbred	University of Luebeck, Germany
8914	female	172	FAM5	C17534	outbred	University of Luebeck, Germany
8915	female	172	FAM5	C17534	outbred	University of Luebeck, Germany
8929	female	172	FAM6	C17538	outbred	University of Luebeck, Germany
8930	female	172	FAM6	C17538	outbred	University of Luebeck, Germany
8931	female	172	FAM6	C17538	outbred	University of Luebeck, Germany
8948	male	159	FAM6	C17538	outbred	University of Luebeck, Germany
8949	male	194	FAM6	C17538	outbred	University of Luebeck, Germany
8950	female	176	FAM7	C17541	outbred	University of Luebeck, Germany
8951	female	176	FAM7	C17541	outbred	University of Luebeck, Germany
8952	female	174	FAM7	C17541	outbred	University of Luebeck, Germany
8953	female	174	FAM7	C17541	outbred	University of Luebeck, Germany
8954	male	161	FAM7	C17541	outbred	University of Luebeck, Germany
8955	male	196	FAM7	C17541	outbred	University of Luebeck, Germany
8963	female	165	FAM8	C17544	outbred	University of Luebeck, Germany
8964	female	163	FAM8	C17544	outbred	University of Luebeck, Germany
8965	female	163	FAM8	C17544	outbred	University of Luebeck, Germany
8966	female	163	FAM8	C17544	outbred	University of Luebeck, Germany
8968	male	185	FAM8	C17544	outbred	University of Luebeck, Germany
8970	female	177	FAM9	C17547	outbred	University of Luebeck, Germany
8971	female	177	FAM9	C17547	outbred	University of Luebeck, Germany
8972	female	175	FAM9	C17547	outbred	University of Luebeck, Germany
8973	female	175	FAM9	C17547	outbred	University of Luebeck, Germany
8974	male	162	FAM9	C17547	outbred	University of Luebeck, Germany
8976	female	174	FAM10	C17550	outbred	University of Luebeck, Germany
8977	female	174	FAM10	C17550	outbred	University of Luebeck, Germany
8978	female	172	FAM10	C17550	outbred	University of Luebeck, Germany
8979	female	172	FAM10	C17550	outbred	University of Luebeck, Germany
8991	male	195	FAM10	C17550	outbred	University of Luebeck, Germany
8995	female	174	FAM11	C17554	outbred	University of Luebeck, Germany
8996	female	195	FAM11	C17554	outbred	University of Luebeck, Germany
8997	female	195	FAM11	C17554	outbred	University of Luebeck, Germany
8998	female	195	FAM11	C17554	outbred	University of Luebeck, Germany
9001	male	159	FAM11	C17558	outbred	University of Luebeck, Germany
9014	female	174	FAM12	C17558	outbred	University of Luebeck, Germany
9015	male	172	FAM12	C17558	outbred	University of Luebeck, Germany
9019	female	195	FAM13	C17700	outbred	University of Luebeck, Germany
9022	male	160	FAM13	C17700	outbred	University of Luebeck, Germany
9031	female	172	FAM14	C17704	outbred	University of Luebeck, Germany
9034	male	159	FAM14	C17704	outbred	University of Luebeck, Germany
9037	female	172	FAM15	C17708	outbred	University of Luebeck, Germany
9039	male	198	FAM15	C17708	outbred	University of Luebeck, Germany
9040	male	162	FAM15	C17708	outbred	University of Luebeck, Germany
9044	female	172	FAM15	C17708	outbred	University of Luebeck, Germany
9059	female	194	FAM16	C17713	outbred	University of Luebeck, Germany
9060	female	194	FAM16	C17713	outbred	University of Luebeck, Germany
9061	female	200	FAM16	C17713	outbred	University of Luebeck, Germany
9063	female	172	FAM16	C17713	outbred	University of Luebeck, Germany
9064	male	172	FAM16	C17713	outbred	University of Luebeck, Germany
9067	female	174	FAM17	C17716	outbred	University of Luebeck, Germany
9068	female	174	FAM17	C17716	outbred	University of Luebeck, Germany
9070	female	173	FAM17	C17716	outbred	University of Luebeck, Germany
9085	male	159	FAM17	C17716	outbred	University of Luebeck, Germany
9086	male	165	FAM17	C17716	outbred	University of Luebeck, Germany
9087	female	141	FAM18	C17721	outbred	University of Luebeck, Germany
9088	female	141	FAM18	C17721	outbred	University of Luebeck, Germany
9089	female	173	FAM18	C17721	outbred	University of Luebeck, Germany
9090	female	173	FAM18	C17721	outbred	University of Luebeck, Germany
9091	female	173	FAM18	C17721	outbred	University of Luebeck, Germany
9103	male	127	FAM18	C17721	outbred	University of Luebeck, Germany
9104	male	162	FAM18	C17721	outbred	University of Luebeck, Germany
9105	female	174	FAM19	C17724	outbred	University of Luebeck, Germany
9106	female	174	FAM19	C17724	outbred	University of Luebeck, Germany
9107	female	173	FAM19	C17724	outbred	University of Luebeck, Germany
9109	male	173	FAM19	C17724	outbred	University of Luebeck, Germany
9110	female	177	FAM20	C17727	outbred	University of Luebeck, Germany
9111	female	177	FAM20	C17727	outbred	University of Luebeck, Germany
9112	female	195	FAM20	C17727	outbred	University of Luebeck, Germany
9114	male	176	FAM20	C17727	outbred	University of Luebeck, Germany
9115	male	163	FAM20	C17727	outbred	University of Luebeck, Germany
9119	female	174	FAM21	C17730	outbred	University of Luebeck, Germany
9120	female	174	FAM21	C17730	outbred	University of Luebeck, Germany
9121	female	195	FAM21	C17730	outbred	University of Luebeck, Germany

9122	female	195	FAM21	C17730	outbred	University of Luebeck, Germany
9123	female	195	FAM21	C17730	outbred	University of Luebeck, Germany
9129	male	160	FAM21	C17730	outbred	University of Luebeck, Germany
9130	male	173	FAM21	C17730	outbred	University of Luebeck, Germany
9131	female	176	FAM22	C17735	outbred	University of Luebeck, Germany
9132	female	176	FAM22	C17735	outbred	University of Luebeck, Germany
9133	female	167	FAM22	C17735	outbred	University of Luebeck, Germany
9134	female	175	FAM22	C17735	outbred	University of Luebeck, Germany
9135	female	167	FAM22	C17735	outbred	University of Luebeck, Germany
9144	male	162	FAM22	C17735	outbred	University of Luebeck, Germany
9145	male	169	FAM22	C17735	outbred	University of Luebeck, Germany
9146	male	197	FAM22	C17735	outbred	University of Luebeck, Germany
9147	female	174	FAM23	C17740	outbred	University of Luebeck, Germany
9148	female	174	FAM23	C17740	outbred	University of Luebeck, Germany
9149	female	167	FAM23	C17740	outbred	University of Luebeck, Germany
9150	female	175	FAM23	C17740	outbred	University of Luebeck, Germany
9151	female	167	FAM23	C17740	outbred	University of Luebeck, Germany
9153	female	176	FAM24	C17742	outbred	University of Luebeck, Germany
9155	female	203	FAM24	C17742	outbred	University of Luebeck, Germany
9156	female	203	FAM24	C17742	outbred	University of Luebeck, Germany
9157	female	203	FAM24	C17742	outbred	University of Luebeck, Germany
9161	male	197	FAM24	C17742	outbred	University of Luebeck, Germany
9175	female	202	FAM25	C17748	outbred	University of Luebeck, Germany
9183	female	202	FAM26	C17752	outbred	University of Luebeck, Germany
9184	female	202	FAM26	C17752	outbred	University of Luebeck, Germany
9185	male	169	FAM26	C17752	outbred	University of Luebeck, Germany
9187	male	197	FAM26	C17752	outbred	University of Luebeck, Germany
9189	female	194	FAM27	C17759	outbred	University of Luebeck, Germany
9190	female	194	FAM27	C17759	outbred	University of Luebeck, Germany
9191	female	200	FAM27	C17759	outbred	University of Luebeck, Germany
9193	male	201	FAM27	C17759	outbred	University of Luebeck, Germany
9205	male	195	FAM27	C17759	outbred	University of Luebeck, Germany
9206	male	160	FAM27	C17759	outbred	University of Luebeck, Germany
9208	female	90	FAM28	C17765	outbred	University of Luebeck, Germany
9209	female	90	FAM28	C17765	outbred	University of Luebeck, Germany
9210	female	90	FAM28	C17765	outbred	University of Luebeck, Germany
9211	female	90	FAM28	C17765	outbred	University of Luebeck, Germany
9214	female	199	FAM29	C17770	outbred	University of Luebeck, Germany
9215	female	166	FAM29	C17770	outbred	University of Luebeck, Germany
9216	female	166	FAM29	C17770	outbred	University of Luebeck, Germany
9228	male	159	FAM29	C17770	outbred	University of Luebeck, Germany
9230	male	200	FAM29	C17770	outbred	University of Luebeck, Germany
9231	female	202	FAM30	C17783	outbred	University of Luebeck, Germany
9232	female	202	FAM30	C17783	outbred	University of Luebeck, Germany
9233	female	199	FAM30	C17783	outbred	University of Luebeck, Germany
9234	female	166	FAM30	C17783	outbred	University of Luebeck, Germany
9235	female	166	FAM30	C17783	outbred	University of Luebeck, Germany
9248	male	162	FAM30	C17783	outbred	University of Luebeck, Germany
9250	male	203	FAM30	C17783	outbred	University of Luebeck, Germany
9255	female	200	FAM31	C17789	outbred	University of Luebeck, Germany
9256	female	200	FAM31	C17789	outbred	University of Luebeck, Germany
9258	female	167	FAM31	C17789	outbred	University of Luebeck, Germany
9259	male	160	FAM31	C17789	outbred	University of Luebeck, Germany
9262	female	199	FAM32	C17793	outbred	University of Luebeck, Germany
9264	male	166	FAM32	C17793	outbred	University of Luebeck, Germany
9266	male	159	FAM32	C17793	outbred	University of Luebeck, Germany
9271	female	203	FAM33	C17798	outbred	University of Luebeck, Germany
9272	female	203	FAM33	C17798	outbred	University of Luebeck, Germany
9273	female	167	FAM33	C17798	outbred	University of Luebeck, Germany
9274	female	200	FAM33	C17798	outbred	University of Luebeck, Germany
9275	female	167	FAM33	C17798	outbred	University of Luebeck, Germany
9281	male	162	FAM33	C17798	outbred	University of Luebeck, Germany
9282	male	175	FAM33	C17798	outbred	University of Luebeck, Germany
9285	female	200	FAM34	C17804	outbred	University of Luebeck, Germany
9286	male	159	FAM34	C17804	outbred	University of Luebeck, Germany
9287	male	173	FAM34	C17804	outbred	University of Luebeck, Germany
9300	female	200	FAM35	C17808	outbred	University of Luebeck, Germany
9301	female	200	FAM35	C17808	outbred	University of Luebeck, Germany
9302	female	173	FAM35	C17808	outbred	University of Luebeck, Germany
9303	female	165	FAM35	C17808	outbred	University of Luebeck, Germany
9304	female	165	FAM35	C17808	outbred	University of Luebeck, Germany
9318	male	159	FAM35	C17808	outbred	University of Luebeck, Germany
9319	male	201	FAM35	C17808	outbred	University of Luebeck, Germany
9321	female	194	FAM36	C17813	outbred	University of Luebeck, Germany
9322	female	165	FAM36	C17813	outbred	University of Luebeck, Germany
9323	female	165	FAM36	C17813	outbred	University of Luebeck, Germany
9324	female	173	FAM36	C17813	outbred	University of Luebeck, Germany
9336	male	173	FAM36	C17813	outbred	University of Luebeck, Germany
9337	male	165	FAM36	C17813	outbred	University of Luebeck, Germany
9338	male	159	FAM36	C17813	outbred	University of Luebeck, Germany
9339	female	202	FAM37	C17819	outbred	University of Luebeck, Germany
9340	female	202	FAM37	C17819	outbred	University of Luebeck, Germany
9342	female	167	FAM37	C17819	outbred	University of Luebeck, Germany
9343	female	175	FAM37	C17819	outbred	University of Luebeck, Germany
9353	female	196	FAM38	C17828	outbred	University of Luebeck, Germany
9354	male	161	FAM38	C17828	outbred	University of Luebeck, Germany
9355	female	165	FAM38	C17828	outbred	University of Luebeck, Germany
9356	female	165	FAM38	C17828	outbred	University of Luebeck, Germany
9357	female	173	FAM38	C17828	outbred	University of Luebeck, Germany
9359	male	173	FAM38	C17828	outbred	University of Luebeck, Germany
9360	male	173	FAM38	C17828	outbred	University of Luebeck, Germany

9365	female	200	FAM39	C17823	outbred	University of Luebeck, Germany
9366	female	167	FAM39	C17823	outbred	University of Luebeck, Germany
9367	female	167	FAM39	C17823	outbred	University of Luebeck, Germany
9368	female	175	FAM39	C17823	outbred	University of Luebeck, Germany
9375	male	175	FAM39	C17823	outbred	University of Luebeck, Germany
9376	male	173	FAM39	C17823	outbred	University of Luebeck, Germany
9381	female	203	FAM40	C17835	outbred	University of Luebeck, Germany
9382	male	203	FAM40	C17835	outbred	University of Luebeck, Germany
9388	male	168	FAM41	C17840	outbred	University of Luebeck, Germany
9389	male	154	FAM41	C17840	outbred	University of Luebeck, Germany
9392	male	203	FAM42	C17844	outbred	University of Luebeck, Germany
9393	female	203	FAM42	C17844	outbred	University of Luebeck, Germany
9394	female	203	FAM42	C17844	outbred	University of Luebeck, Germany
9406	male	167	FAM42	C17844	outbred	University of Luebeck, Germany
9407	male	161	FAM42	C17844	outbred	University of Luebeck, Germany
9408	female	186	FAM43	C17848	outbred	University of Luebeck, Germany
9409	female	186	FAM43	C17848	outbred	University of Luebeck, Germany
9410	female	192	FAM43	C17848	outbred	University of Luebeck, Germany
9411	female	192	FAM43	C17848	outbred	University of Luebeck, Germany
9412	female	193	FAM43	C17848	outbred	University of Luebeck, Germany
9416	male	151	FAM43	C17848	outbred	University of Luebeck, Germany
9417	male	165	FAM43	C17848	outbred	University of Luebeck, Germany
9418	male	193	FAM43	C17848	outbred	University of Luebeck, Germany
9429	female	190	FAM44	C17851	outbred	University of Luebeck, Germany
9430	female	190	FAM44	C17851	outbred	University of Luebeck, Germany
9431	female	169	FAM44	C17851	outbred	University of Luebeck, Germany
9432	female	133	FAM45	C17853	outbred	University of Luebeck, Germany
9433	female	133	FAM45	C17853	outbred	University of Luebeck, Germany
9436	female	183	FAM46	C17855	outbred	University of Luebeck, Germany
9437	female	169	FAM46	C17855	outbred	University of Luebeck, Germany
9438	male	161	FAM46	C17855	outbred	University of Luebeck, Germany
9439	female	196	FAM47	C17861	outbred	University of Luebeck, Germany
9440	female	196	FAM47	C17861	outbred	University of Luebeck, Germany
9442	male	167	FAM47	C17861	outbred	University of Luebeck, Germany
9443	male	161	FAM47	C17861	outbred	University of Luebeck, Germany
9444	male	175	FAM47	C17861	outbred	University of Luebeck, Germany
9448	female	203	FAM47	C17861	outbred	University of Luebeck, Germany
9462	female	196	FAM48	C17867	outbred	University of Luebeck, Germany
9464	female	191	FAM48	C17867	outbred	University of Luebeck, Germany
9465	female	191	FAM48	C17867	outbred	University of Luebeck, Germany
9471	female	194	FAM49	C17871	outbred	University of Luebeck, Germany
9472	female	194	FAM49	C17871	outbred	University of Luebeck, Germany
9473	female	173	FAM49	C17871	outbred	University of Luebeck, Germany
9474	female	165	FAM49	C17871	outbred	University of Luebeck, Germany
9475	female	165	FAM49	C17871	outbred	University of Luebeck, Germany
9480	male	165	FAM49	C17871	outbred	University of Luebeck, Germany

Supplementary Table 1.3 Metadata of MPI-Lab population (n=29)

Sample_ID	Sex	Age (in days)	Cage	Family	Litter	Strain	Breeding scheme	Breeding facility
MM17	male	400	CB1124	CB709	2	CB	Outbred	Max Planck Institute, Ploen, Germany
MM18	male	400	CB868	CB709	2	CB	Outbred	Max Planck Institute, Ploen, Germany
MM19	male	400	CB551	CB709	2	CB	Outbred	Max Planck Institute, Ploen, Germany
MM20	female	400	CB1146	CB709	2	CB	Outbred	Max Planck Institute, Ploen, Germany
MM21	female	400	CB1146	CB709	2	CB	Outbred	Max Planck Institute, Ploen, Germany
MM22	female	400	CB813	CB709	2	CB	Outbred	Max Planck Institute, Ploen, Germany
MM23	male	378	CB83	CB706	2	CB	Outbred	Max Planck Institute, Ploen, Germany
MM24	male	378	CB329	CB706	2	CB	Outbred	Max Planck Institute, Ploen, Germany
MM25	female	378	CB173	CB706	2	CB	Outbred	Max Planck Institute, Ploen, Germany
MM26	female	378	CB173	CB706	2	CB	Outbred	Max Planck Institute, Ploen, Germany
MM27	female	378	CB780	CB706	2	CB	Outbred	Max Planck Institute, Ploen, Germany
50011121	male	557	MC141	MC806	1	MC	Outbred	Max Planck Institute, Ploen, Germany
50011126	female	557	MC1123	MC806	1	MC	Outbred	Max Planck Institute, Ploen, Germany
50013455	female	374	MC1319	MC903	1	MC	Outbred	Max Planck Institute, Ploen, Germany
50013456	female	374	MC1319	MC903	1	MC	Outbred	Max Planck Institute, Ploen, Germany
50013466	male	372	MC1345	MC906	1	MC	Outbred	Max Planck Institute, Ploen, Germany
50013641	male	361	MC40	MC901	1	MC	Outbred	Max Planck Institute, Ploen, Germany
50013642	male	361	MC876	MC901	2	MC	Outbred	Max Planck Institute, Ploen, Germany
50013823	female	349	MC278	MC906	1	MC	Outbred	Max Planck Institute, Ploen, Germany
MC903A1F	female	374	MC1319	MC903	1	MC	Outbred	Max Planck Institute, Ploen, Germany
50019000	male	95	WSB1995	WSB137	2	WSB/EiJ	Inbred	Max Planck Institute, Ploen, Germany
50019001	male	95	WSB1995	WSB137	2	WSB/EiJ	Inbred	Max Planck Institute, Ploen, Germany
50019002	male	95	WSB1996	WSB137	2	WSB/EiJ	Inbred	Max Planck Institute, Ploen, Germany
50019003	male	95	WSB1996	WSB137	2	WSB/EiJ	Inbred	Max Planck Institute, Ploen, Germany
50019004	female	95	WSB1997	WSB137	2	WSB/EiJ	Inbred	Max Planck Institute, Ploen, Germany
50019005	male	94	WSB1998	WSB138	1	WSB/EiJ	Inbred	Max Planck Institute, Ploen, Germany
50019007	male	94	WSB1999	WSB138	1	WSB/EiJ	Inbred	Max Planck Institute, Ploen, Germany
50019008	female	94	WSB2000	WSB138	1	WSB/EiJ	Inbred	Max Planck Institute, Ploen, Germany
50019009	female	94	WSB2001	WSB138	1	WSB/EiJ	Inbred	Max Planck Institute, Ploen, Germany

Supplementary Table 1.4 Metadata of C57BL/6J population (n=13)

Identification ID	Sex	Strain	Age (in days)	Family	Cage	Breeding scheme	Breeding facility
B6_2	male	C57BL/6J	70	BL/6_1	513	Inbred	Max Planck institute, Ploen, Germany
B6_3	female	C57BL/6J	70	BL/6_1	520	Inbred	Max Planck institute, Ploen, Germany
B6_4	male	C57BL/6J	70	BL/6_2	1096	Inbred	Max Planck institute, Ploen, Germany
B6_5	male	C57BL/6J	70	BL/6_2	1096	Inbred	Max Planck institute, Ploen, Germany
B6_6	female	C57BL/6J	70	BL/6_3	1213	Inbred	Max Planck institute, Ploen, Germany
B6_7	female	C57BL/6J	72	BL/6_4	1594	Inbred	Max Planck institute, Ploen, Germany
B6_10	male	C57BL/6J	72	BL/6_4	667	Inbred	Max Planck institute, Ploen, Germany
B6_9	male	C57BL/6J	72	BL/6_4	667	Inbred	Max Planck institute, Ploen, Germany
B6_1	male	C57BL/6J	65	BL/6_5	105	Inbred	Max Planck institute, Ploen, Germany
B6_8	female	C57BL/6J	65	BL/6_5	114	Inbred	Max Planck institute, Ploen, Germany
B6_13	male	C57BL/6J	73	BL/6_6	1026	Inbred	Max Planck institute, Ploen, Germany
B6_14	male	C57BL/6J	73	BL/6_6	1026	Inbred	Max Planck institute, Ploen, Germany
B6_12	female	C57BL/6J	42	BL/6_6	3059	Inbred	Max Planck institute, Ploen, Germany

Supplementary Table 1.5 Metadata of DNA samples used for "Decontam"

Sample_ID	Sample_Analysed	X.SampleID	PlateNumber	Subject	Habitat	quant_reading	Sample_or_Control
8858	8858	8858	1	Mouse_Skin	HL-Lab	44123	True Sample
8862	8862	8862	1	Mouse_Skin	HL-Lab	12610	True Sample
8872	8872	8872	1	Mouse_Skin	HL-Lab	4802	True Sample
8874	8874	8874	1	Mouse_Skin	HL-Lab	13788	True Sample
8884	8884	8884	1	Mouse_Skin	HL-Lab	7506	True Sample
8885	8885	8885	1	Mouse_Skin	HL-Lab	24202	True Sample
8886	8886	8886	1	Mouse_Skin	HL-Lab	20704	True Sample
8887	8887	8887	1	Mouse_Skin	HL-Lab	14884	True Sample
8888	8888	8888	1	Mouse_Skin	HL-Lab	16233	True Sample
8889	8889	8889	1	Mouse_Skin	HL-Lab	8808	True Sample
8894	8894	8894	1	Mouse_Skin	HL-Lab	18152	True Sample
8901	8901	8901	1	Mouse_Skin	HL-Lab	11698	True Sample
8902	8902	8902	1	Mouse_Skin	HL-Lab	17018	True Sample
8903	8903	8903	1	Mouse_Skin	HL-Lab	6780	True Sample
8904	8904	8904	1	Mouse_Skin	HL-Lab	28166	True Sample
8905	8905	8905	1	Mouse_Skin	HL-Lab	548	True Sample
8906	8906	8906	1	Mouse_Skin	HL-Lab	4964	True Sample
8907	8907	8907	1	Mouse_Skin	HL-Lab	21738	True Sample
8908	8908	8908	1	Mouse_Skin	HL-Lab	12110	True Sample
8909	8909	8909	1	Mouse_Skin	HL-Lab	10701	True Sample
8910	8910	8910	1	Mouse_Skin	HL-Lab	8710	True Sample
8911	8911	8911	1	Mouse_Skin	HL-Lab	18633	True Sample
8912	8912	8912	1	Mouse_Skin	HL-Lab	5647	True Sample
8913	8913	8913	1	Mouse_Skin	HL-Lab	18902	True Sample
8914	8914	8914	1	Mouse_Skin	HL-Lab	16661	True Sample
8915	8915	8915	1	Mouse_Skin	HL-Lab	29182	True Sample
8924	8924	8924	1	Mouse_Skin	HL-Lab	12114	True Sample
8925	8925	8925	1	Mouse_Skin	HL-Lab	13285	True Sample
8926	8926	8926	1	Mouse_Skin	HL-Lab	18052	True Sample
8929	8929	8929	1	Mouse_Skin	HL-Lab	11335	True Sample
8930	8930	8930	1	Mouse_Skin	HL-Lab	2757	True Sample
8931	8931	8931	1	Mouse_Skin	HL-Lab	5218	True Sample
8948	8948	8948	1	Mouse_Skin	HL-Lab	18870	True Sample
8949	8949	8949	1	Mouse_Skin	HL-Lab	16538	True Sample
8950	8950	8950	1	Mouse_Skin	HL-Lab	12514	True Sample
8951	8951	8951	1	Mouse_Skin	HL-Lab	14420	True Sample
8952	8952	8952	1	Mouse_Skin	HL-Lab	6626	True Sample
8953	8953	8953	1	Mouse_Skin	HL-Lab	14703	True Sample
8954	8954	8954	1	Mouse_Skin	HL-Lab	20979	True Sample
8955	8955	8955	1	Mouse_Skin	HL-Lab	48331	True Sample
8962	8962	8962	1	Mouse_Skin	HL-Lab	19861	True Sample
8963	8963	8963	1	Mouse_Skin	HL-Lab	23109	True Sample
8964	8964	8964	1	Mouse_Skin	HL-Lab	7342	True Sample
8965	8965	8965	1	Mouse_Skin	HL-Lab	10194	True Sample
8966	8966	8966	1	Mouse_Skin	HL-Lab	3008	True Sample
8967	8967	8967	1	Mouse_Skin	HL-Lab	16410	True Sample
8968	8968	8968	1	Mouse_Skin	HL-Lab	16160	True Sample
8969	8969	8969	1	Mouse_Skin	HL-Lab	18396	True Sample
8970	8970	8970	1	Mouse_Skin	HL-Lab	12284	True Sample
8971	8971	8971	1	Mouse_Skin	HL-Lab	16439	True Sample
8972	8972	8972	1	Mouse_Skin	HL-Lab	16694	True Sample
8973	8973	8973	1	Mouse_Skin	HL-Lab	29493	True Sample
8974	8974	8974	1	Mouse_Skin	HL-Lab	12264	True Sample
8976	8976	8976	1	Mouse_Skin	HL-Lab	16439	True Sample
8977	8977	8977	1	Mouse_Skin	HL-Lab	20405	True Sample
8978	8978	8978	1	Mouse_Skin	HL-Lab	19727	True Sample
8979	8979	8979	1	Mouse_Skin	HL-Lab	25209	True Sample
8993	8993	8993	1	Mouse_Skin	HL-Lab	17152	True Sample
8995	8995	8995	1	Mouse_Skin	HL-Lab	25708	True Sample
8996	8996	8996	1	Mouse_Skin	HL-Lab	11502	True Sample
8998	8998	8998	1	Mouse_Skin	HL-Lab	15553	True Sample
9000	9000	9000	1	Mouse_Skin	HL-Lab	13455	True Sample
9001	9001	9001	1	Mouse_Skin	HL-Lab	6325	True Sample
9013	9013	9013	1	Mouse_Skin	HL-Lab	609	True Sample
9014	9014	9014	1	Mouse_Skin	HL-Lab	11419	True Sample
9015	9015	9015	1	Mouse_Skin	HL-Lab	18110	True Sample
9019	9019	9019	1	Mouse_Skin	HL-Lab	10195	True Sample
9020	9020	9020	1	Mouse_Skin	HL-Lab	10436	True Sample
9021	9021	9021	1	Mouse_Skin	HL-Lab	9172	True Sample
9022	9022	9022	1	Mouse_Skin	HL-Lab	4292	True Sample
9031	9031	9031	1	Mouse_Skin	HL-Lab	16841	True Sample
9034	9034	9034	1	Mouse_Skin	HL-Lab	17683	True Sample
9035	9035	9035	1	Mouse_Skin	HL-Lab	18636	True Sample
9036	9036	9036	1	Mouse_Skin	HL-Lab	17948	True Sample
9037	9037	9037	1	Mouse_Skin	HL-Lab	23904	True Sample
9038	9038	9038	1	Mouse_Skin	HL-Lab	9456	True Sample
9039	9039	9039	1	Mouse_Skin	HL-Lab	10162	True Sample
9040	9040	9040	1	Mouse_Skin	HL-Lab	9371	True Sample
9044	9044	9044	1	Mouse_Skin	HL-Lab	37388	True Sample
9059	9059	9059	1	Mouse_Skin	HL-Lab	43648	True Sample
9060	9060	9060	1	Mouse_Skin	HL-Lab	24947	True Sample
9061	9061	9061	1	Mouse_Skin	HL-Lab	10914	True Sample
9063	9063	9063	1	Mouse_Skin	HL-Lab	26787	True Sample
9064	9064	9064	1	Mouse_Skin	HL-Lab	24998	True Sample
9067	9067	9067	1	Mouse_Skin	HL-Lab	15679	True Sample
9068	9068	9068	1	Mouse_Skin	HL-Lab	11649	True Sample
9070	9070	9070	1	Mouse_Skin	HL-Lab	16171	True Sample
9084	9084	9084	1	Mouse_Skin	HL-Lab	31320	True Sample

9085	9085	9085	1	Mouse_Skin	HL-Lab	16770	True Sample
9086	9086	9086	1	Mouse_Skin	HL-Lab	7945	True Sample
9087	9087	9087	1	Mouse_Skin	HL-Lab	18145	True Sample
9088	9088	9088	1	Mouse_Skin	HL-Lab	16967	True Sample
9089	9089	9089	1	Mouse_Skin	HL-Lab	28699	True Sample
9090	9090	9090	1	Mouse_Skin	HL-Lab	18213	True Sample
9091	9091	9091	1	Mouse_Skin	HL-Lab	33638	True Sample
9103	9103	9103	1	Mouse_Skin	HL-Lab	12713	True Sample
9104	9104	9104	1	Mouse_Skin	HL-Lab	9713	True Sample
9105	9105	9105	1	Mouse_Skin	HL-Lab	7827	True Sample
9106	9106	9106	1	Mouse_Skin	HL-Lab	9447	True Sample
9107	9107	9107	1	Mouse_Skin	HL-Lab	16768	True Sample
9108	9108	9108	1	Mouse_Skin	HL-Lab	28345	True Sample
9109	9109	9109	1	Mouse_Skin	HL-Lab	25956	True Sample
9110	9110	9110	1	Mouse_Skin	HL-Lab	23748	True Sample
9111	9111	9111	1	Mouse_Skin	HL-Lab	16217	True Sample
9112	9112	9112	1	Mouse_Skin	HL-Lab	23401	True Sample
9113	9113	9113	1	Mouse_Skin	HL-Lab	8595	True Sample
9114	9114	9114	1	Mouse_Skin	HL-Lab	477	True Sample
9115	9115	9115	1	Mouse_Skin	HL-Lab	10616	True Sample
9119	9119	9119	1	Mouse_Skin	HL-Lab	20471	True Sample
9120	9120	9120	1	Mouse_Skin	HL-Lab	8769	True Sample
9121	9121	9121	1	Mouse_Skin	HL-Lab	5818	True Sample
9122	9122	9122	1	Mouse_Skin	HL-Lab	3711	True Sample
9123	9123	9123	1	Mouse_Skin	HL-Lab	14191	True Sample
9129	9129	9129	1	Mouse_Skin	HL-Lab	10105	True Sample
9130	9130	9130	1	Mouse_Skin	HL-Lab	41726	True Sample
9132	9132	9132	1	Mouse_Skin	HL-Lab	12320	True Sample
9133	9133	9133	1	Mouse_Skin	HL-Lab	25283	True Sample
9134	9134	9134	1	Mouse_Skin	HL-Lab	37765	True Sample
9135	9135	9135	1	Mouse_Skin	HL-Lab	16748	True Sample
9144	9144	9144	1	Mouse_Skin	HL-Lab	23006	True Sample
9146	9146	9146	1	Mouse_Skin	HL-Lab	6679	True Sample
9153	9153	9153	1	Mouse_Skin	HL-Lab	20398	True Sample
9175	9175	9175	1	Mouse_Skin	HL-Lab	9849	True Sample
9183	9183	9183	1	Mouse_Skin	HL-Lab	10228	True Sample
9184	9184	9184	1	Mouse_Skin	HL-Lab	8675	True Sample
9185	9185	9185	1	Mouse_Skin	HL-Lab	9302	True Sample
9186	9186	9186	1	Mouse_Skin	HL-Lab	16453	True Sample
9187	9187	9187	1	Mouse_Skin	HL-Lab	12284	True Sample
9189	9189	9189	1	Mouse_Skin	HL-Lab	20296	True Sample
9190	9190	9190	1	Mouse_Skin	HL-Lab	21307	True Sample
9191	9191	9191	1	Mouse_Skin	HL-Lab	16194	True Sample
9193	9193	9193	1	Mouse_Skin	HL-Lab	15827	True Sample
9205	9205	9205	1	Mouse_Skin	HL-Lab	14234	True Sample
9206	9206	9206	1	Mouse_Skin	HL-Lab	13394	True Sample
9207	9207	9207	1	Mouse_Skin	HL-Lab	20372	True Sample
9208	9208	9208	1	Mouse_Skin	HL-Lab	16179	True Sample
9209	9209	9209	1	Mouse_Skin	HL-Lab	11479	True Sample
9210	9210	9210	1	Mouse_Skin	HL-Lab	29132	True Sample
9211	9211	9211	1	Mouse_Skin	HL-Lab	12316	True Sample
9212	9212	9212	1	Mouse_Skin	HL-Lab	10107	True Sample
9214	9214	9214	1	Mouse_Skin	HL-Lab	7490	True Sample
9215	9215	9215	1	Mouse_Skin	HL-Lab	8491	True Sample
9216	9216	9216	1	Mouse_Skin	HL-Lab	9087	True Sample
9228	9228	9228	1	Mouse_Skin	HL-Lab	11492	True Sample
9229	9229	9229	1	Mouse_Skin	HL-Lab	9059	True Sample
9230	9230	9230	1	Mouse_Skin	HL-Lab	11722	True Sample
9231	9231	9231	1	Mouse_Skin	HL-Lab	8475	True Sample
9232	9232	9232	1	Mouse_Skin	HL-Lab	7807	True Sample
9233	9233	9233	1	Mouse_Skin	HL-Lab	5841	True Sample
9234	9234	9234	1	Mouse_Skin	HL-Lab	3440	True Sample
9235	9235	9235	1	Mouse_Skin	HL-Lab	11674	True Sample
9248	9248	9248	1	Mouse_Skin	HL-Lab	12289	True Sample
9249	9249	9249	1	Mouse_Skin	HL-Lab	30803	True Sample
9250	9250	9250	1	Mouse_Skin	HL-Lab	12325	True Sample
9255	9255	9255	1	Mouse_Skin	HL-Lab	16545	True Sample
9256	9256	9256	1	Mouse_Skin	HL-Lab	6281	True Sample
9259	9259	9259	1	Mouse_Skin	HL-Lab	13573	True Sample
9260	9260	9260	1	Mouse_Skin	HL-Lab	10470	True Sample
9262	9262	9262	1	Mouse_Skin	HL-Lab	12730	True Sample
9264	9264	9264	1	Mouse_Skin	HL-Lab	12194	True Sample
9266	9266	9266	1	Mouse_Skin	HL-Lab	19463	True Sample
9271	9271	9271	1	Mouse_Skin	HL-Lab	7750	True Sample
9272	9272	9272	1	Mouse_Skin	HL-Lab	6453	True Sample
9273	9273	9273	1	Mouse_Skin	HL-Lab	9444	True Sample
9274	9274	9274	1	Mouse_Skin	HL-Lab	8432	True Sample
9275	9275	9275	1	Mouse_Skin	HL-Lab	11573	True Sample
9281	9281	9281	1	Mouse_Skin	HL-Lab	15637	True Sample
9282	9282	9282	1	Mouse_Skin	HL-Lab	18457	True Sample
9283	9283	9283	1	Mouse_Skin	HL-Lab	18809	True Sample
9284	9284	9284	1	Mouse_Skin	HL-Lab	10237	True Sample
9285	9285	9285	1	Mouse_Skin	HL-Lab	11539	True Sample
9286	9286	9286	1	Mouse_Skin	HL-Lab	11462	True Sample
9287	9287	9287	1	Mouse_Skin	HL-Lab	30050	True Sample
9300	9300	9300	1	Mouse_Skin	HL-Lab	10663	True Sample
9301	9301	9301	1	Mouse_Skin	HL-Lab	10105	True Sample
9302	9302	9302	1	Mouse_Skin	HL-Lab	27103	True Sample
9303	9303	9303	1	Mouse_Skin	HL-Lab	10094	True Sample
9304	9304	9304	1	Mouse_Skin	HL-Lab	20452	True Sample
9318	9318	9318	1	Mouse_Skin	HL-Lab	11789	True Sample

9319	9319	9319	1	Mouse_Skin	HL-Lab	16966	True Sample
9321	9321	9321	1	Mouse_Skin	HL-Lab	24559	True Sample
9322	9322	9322	1	Mouse_Skin	HL-Lab	8705	True Sample
9323	9323	9323	1	Mouse_Skin	HL-Lab	12626	True Sample
9324	9324	9324	1	Mouse_Skin	HL-Lab	34787	True Sample
9336	9336	9336	1	Mouse_Skin	HL-Lab	32987	True Sample
9337	9337	9337	1	Mouse_Skin	HL-Lab	12060	True Sample
9338	9338	9338	1	Mouse_Skin	HL-Lab	10907	True Sample
9339	9339	9339	1	Mouse_Skin	HL-Lab	9853	True Sample
9340	9340	9340	1	Mouse_Skin	HL-Lab	13020	True Sample
9341	9341	9341	1	Mouse_Skin	HL-Lab	12559	True Sample
9342	9342	9342	1	Mouse_Skin	HL-Lab	20240	True Sample
9343	9343	9343	1	Mouse_Skin	HL-Lab	28631	True Sample
9345	9345	9345	1	Mouse_Skin	HL-Lab	3448	True Sample
9346	9346	9346	1	Mouse_Skin	HL-Lab	16988	True Sample
9353	9353	9353	1	Mouse_Skin	HL-Lab	11239	True Sample
9354	9354	9354	1	Mouse_Skin	HL-Lab	20998	True Sample
9355	9355	9355	1	Mouse_Skin	HL-Lab	1089	True Sample
9356	9356	9356	1	Mouse_Skin	HL-Lab	15561	True Sample
9357	9357	9357	1	Mouse_Skin	HL-Lab	25998	True Sample
9359	9359	9359	1	Mouse_Skin	HL-Lab	27125	True Sample
9360	9360	9360	1	Mouse_Skin	HL-Lab	27726	True Sample
9365	9365	9365	1	Mouse_Skin	HL-Lab	12719	True Sample
9366	9366	9366	1	Mouse_Skin	HL-Lab	12351	True Sample
9367	9367	9367	1	Mouse_Skin	HL-Lab	22495	True Sample
9368	9368	9368	1	Mouse_Skin	HL-Lab	22666	True Sample
9376	9376	9376	1	Mouse_Skin	HL-Lab	29778	True Sample
9380	9380	9380	1	Mouse_Skin	HL-Lab	7989	True Sample
9381	9381	9381	1	Mouse_Skin	HL-Lab	5809	True Sample
9382	9382	9382	1	Mouse_Skin	HL-Lab	6818	True Sample
9388	9388	9388	1	Mouse_Skin	HL-Lab	19331	True Sample
9389	9389	9389	1	Mouse_Skin	HL-Lab	12116	True Sample
9390	9390	9390	1	Mouse_Skin	HL-Lab	5341	True Sample
9391	9391	9391	1	Mouse_Skin	HL-Lab	10392	True Sample
9392	9392	9392	1	Mouse_Skin	HL-Lab	5617	True Sample
9393	9393	9393	1	Mouse_Skin	HL-Lab	5821	True Sample
9394	9394	9394	1	Mouse_Skin	HL-Lab	7216	True Sample
9405	9405	9405	1	Mouse_Skin	HL-Lab	27132	True Sample
9406	9406	9406	1	Mouse_Skin	HL-Lab	7922	True Sample
9408	9408	9408	1	Mouse_Skin	HL-Lab	28512	True Sample
9409	9409	9409	1	Mouse_Skin	HL-Lab	12080	True Sample
9410	9410	9410	1	Mouse_Skin	HL-Lab	19271	True Sample
9411	9411	9411	1	Mouse_Skin	HL-Lab	15153	True Sample
9412	9412	9412	1	Mouse_Skin	HL-Lab	6412	True Sample
9413	9413	9413	1	Mouse_Skin	HL-Lab	26298	True Sample
9416	9416	9416	1	Mouse_Skin	HL-Lab	11205	True Sample
9417	9417	9417	1	Mouse_Skin	HL-Lab	18196	True Sample
9418	9418	9418	1	Mouse_Skin	HL-Lab	11924	True Sample
9429	9429	9429	1	Mouse_Skin	HL-Lab	10889	True Sample
9430	9430	9430	1	Mouse_Skin	HL-Lab	14997	True Sample
9432	9432	9432	1	Mouse_Skin	HL-Lab	11563	True Sample
9433	9433	9433	1	Mouse_Skin	HL-Lab	10875	True Sample
9435	9435	9435	1	Mouse_Skin	HL-Lab	7952	True Sample
9436	9436	9436	1	Mouse_Skin	HL-Lab	18492	True Sample
9437	9437	9437	1	Mouse_Skin	HL-Lab	25534	True Sample
9438	3438	3438	1	Mouse_Skin	HL-Lab	25657	True Sample
9439	9439	9439	1	Mouse_Skin	HL-Lab	15418	True Sample
9440	9440	9440	1	Mouse_Skin	HL-Lab	18800	True Sample
9441	9441	9441	1	Mouse_Skin	HL-Lab	6137	True Sample
9442	9442	9442	1	Mouse_Skin	HL-Lab	12152	True Sample
9443	9443	9443	1	Mouse_Skin	HL-Lab	16655	True Sample
9444	9444	9444	1	Mouse_Skin	HL-Lab	25937	True Sample
9448	9448	9448	1	Mouse_Skin	HL-Lab	3300	True Sample
9462	9462	9462	1	Mouse_Skin	HL-Lab	20821	True Sample
9463	9463	9463	1	Mouse_Skin	HL-Lab	11291	True Sample
9464	9464	9464	1	Mouse_Skin	HL-Lab	16377	True Sample
9465	9465	9465	1	Mouse_Skin	HL-Lab	9064	True Sample
9466	9466	9466	1	Mouse_Skin	HL-Lab	8408	True Sample
9471	9471	9471	1	Mouse_Skin	HL-Lab	18146	True Sample
9472	9472	9472	1	Mouse_Skin	HL-Lab	30303	True Sample
9473	9473	9473	1	Mouse_Skin	HL-Lab	15514	True Sample
9474	9474	9474	1	Mouse_Skin	HL-Lab	24722	True Sample
9475	9475	9475	1	Mouse_Skin	HL-Lab	15582	True Sample
9480	9480	9480	1	Mouse_Skin	HL-Lab	11225	True Sample
9482	9482	9482	1	Mouse_Skin	HL-Lab	33470	True Sample
9949	9949	9949	1	Mouse_Skin	HL-Lab	9419	True Sample
50011121	50011121	50011121	1	Mouse_Skin	Lab-MPI	5716	True Sample
50011126	50011126	50011126	1	Mouse_Skin	Lab-MPI	11062	True Sample
50013455	50013455	50013455	1	Mouse_Skin	Lab-MPI	23991	True Sample
50013456	50013456	50013456	1	Mouse_Skin	Lab-MPI	15041	True Sample
50013466	50013466	50013466	1	Mouse_Skin	Lab-MPI	6537	True Sample
50013641	50013641	50013641	1	Mouse_Skin	Lab-MPI	5607	True Sample
50013642	50013642	50013642	1	Mouse_Skin	Lab-MPI	10239	True Sample
50013823	50013823	50013823	1	Mouse_Skin	Lab-MPI	56649	True Sample
50019000	50019000	50019000	1	Mouse_Skin	Lab-MPI	9342	True Sample
50019001	50019001	50019001	1	Mouse_Skin	Lab-MPI	6348	True Sample
50019002	50019002	50019002	1	Mouse_Skin	Lab-MPI	7706	True Sample
50019003	50019003	50019003	1	Mouse_Skin	Lab-MPI	5814	True Sample
50019004	50019004	50019004	1	Mouse_Skin	Lab-MPI	5146	True Sample
50019005	50019005	50019005	1	Mouse_Skin	Lab-MPI	9052	True Sample
50019007	50019007	50019007	1	Mouse_Skin	Lab-MPI	13173	True Sample

50019008	50019008	50019008	1	Mouse_Skin	Lab-MPI	7669	True Sample
50019009	50019009	50019009	1	Mouse_Skin	Lab-MPI	7958	True Sample
B11	B11DNAMeriem	B11DNAMeriem	1	Mouse_Skin	C57BL/6J	16461	True Sample
B12	B12DNAMeriem	B12DNAMeriem	1	Mouse_Skin	C57BL/6J	691	True Sample
B13	B13DNAMeriem	B13DNAMeriem	1	Mouse_Skin	C57BL/6J	670	True Sample
B14	B14DNAMeriem	B14DNAMeriem	1	Mouse_Skin	C57BL/6J	594	True Sample
B61	B61DNAMeriem	B61DNAMeriem	1	Mouse_Skin	C57BL/6J	11610	True Sample
B610	B610DNAMeriem	B610DNAMeriem	1	Mouse_Skin	C57BL/6J	14371	True Sample
B62	B62DNAMeriem	B62DNAMeriem	1	Mouse_Skin	C57BL/6J	16961	True Sample
B63	B63DNAMeriem	B63DNAMeriem	1	Mouse_Skin	C57BL/6J	1396	True Sample
B64	B64DNAMeriem	B64DNAMeriem	1	Mouse_Skin	C57BL/6J	776	True Sample
B65	B65DNAMeriem	B65DNAMeriem	1	Mouse_Skin	C57BL/6J	1568	True Sample
B66	B66DNAMeriem	B66DNAMeriem	1	Mouse_Skin	C57BL/6J	775	True Sample
B67	B67DNAMeriem	B67DNAMeriem	1	Mouse_Skin	C57BL/6J	2922	True Sample
B68	B68DNAMeriem	B68DNAMeriem	1	Mouse_Skin	C57BL/6J	36241	True Sample
B69	B69DNAMeriem	B69DNAMeriem	1	Mouse_Skin	C57BL/6J	9550	True Sample
B6Negextr	B6NegextrDNAMeriem	B6NegextrDNAMeriem	1	Mouse_Skin	C57BL/6J	534	Control Sample
Cont10_G15	Cont10_G15	Cont10_G15	1	Mouse_Skin	HL-Lab	553	Control Sample
Cont7_G15	Cont7_G15	Cont7_G15	1	Mouse_Skin	HL-Lab	543	Control Sample
Cont8_G15	Cont8_G15	Cont8_G15	1	Mouse_Skin	HL-Lab	695	Control Sample
ctr1	ES_ctrl_1	ctr1	1	Mouse_Skin	Wild	496	Control Sample
EnCJan	EnC_Jan	EnCJan	1	Mouse_Skin	Wild	490	Control Sample
EnCMeriem	EnC_Meriem	EnCMeriem	1	Mouse_Skin	Wild	458	Control Sample
JJM0101	JJM0101	JJM0101	1	Mouse_Skin	Wild	6090	True Sample
JJM0102	JJM0102	JJM0102	1	Mouse_Skin	Wild	631	True Sample
JJM0202	JJM0202	JJM0202	1	Mouse_Skin	Wild	4604	True Sample
JJM0203A	JJM0203A	JJM0203A	1	Mouse_Skin	Wild	5262	True Sample
JJM0203B	JJM0203B	JJM0203B	1	Mouse_Skin	Wild	3308	True Sample
JJM0204	JJM0204	JJM0204	1	Mouse_Skin	Wild	4727	True Sample
JJM0206	JJM0206	JJM0206	1	Mouse_Skin	Wild	1706	True Sample
JJM0208	JJM0208	JJM0208	1	Mouse_Skin	Wild	15633	True Sample
JJM0209	JJM0209	JJM0209	1	Mouse_Skin	Wild	4525	True Sample
JJM0210	JJM0210	JJM0210	1	Mouse_Skin	Wild	8530	True Sample
JJM0401	JJM0401	JJM0401	1	Mouse_Skin	Wild	5231	True Sample
JJM0402	JJM0402	JJM0402	1	Mouse_Skin	Wild	5645	True Sample
JJM0502	JJM0502	JJM0502	1	Mouse_Skin	Wild	6180	True Sample
JJM0503	JJM0503	JJM0503	1	Mouse_Skin	Wild	15675	True Sample
JJM0504	JJM0504	JJM0504	1	Mouse_Skin	Wild	3725	True Sample
JJM0601	JJM0601	JJM0601	1	Mouse_Skin	Wild	3521	True Sample
JJM0602	JJM0602	JJM0602	1	Mouse_Skin	Wild	4437	True Sample
JJM0603	JJM0603	JJM0603	1	Mouse_Skin	Wild	13888	True Sample
JJM0604	JJM0604	JJM0604	1	Mouse_Skin	Wild	3638	True Sample
JJM0701	JJM0701	JJM0701	1	Mouse_Skin	Wild	12644	True Sample
JJM0702	JJM0702	JJM0702	1	Mouse_Skin	Wild	26041	True Sample
JJM0801	JJM0801	JJM0801	1	Mouse_Skin	Wild	23648	True Sample
JJM0802	JJM0802	JJM0802	1	Mouse_Skin	Wild	19186	True Sample
JJM0901	JJM0901	JJM0901	1	Mouse_Skin	Wild	7716	True Sample
JJM0902	JJM0902	JJM0902	1	Mouse_Skin	Wild	23649	True Sample
JJM0903	JJM0903	JJM0903	1	Mouse_Skin	Wild	20619	True Sample
JJM0905	JJM0905	JJM0905	1	Mouse_Skin	Wild	56499	True Sample
JJM0906	JJM0906	JJM0906	1	Mouse_Skin	Wild	30708	True Sample
JJM0908	JJM0908	JJM0908	1	Mouse_Skin	Wild	15058	True Sample
JJM0909	JJM0909	JJM0909	1	Mouse_Skin	Wild	20097	True Sample
JJM0910	JJM0910	JJM0910	1	Mouse_Skin	Wild	18577	True Sample
JJM0911	JJM0911	JJM0911	1	Mouse_Skin	Wild	18116	True Sample
JJM0912	JJM0912	JJM0912	1	Mouse_Skin	Wild	100000	True Sample
JJM1001	JJM1001	JJM1001	1	Mouse_Skin	Wild	35246	True Sample
JJM1002	JJM1002	JJM1002	1	Mouse_Skin	Wild	35004	True Sample
JJM1201	JJM1201	JJM1201	1	Mouse_Skin	Wild	26668	True Sample
JJM1202	JJM1202	JJM1202	1	Mouse_Skin	Wild	17946	True Sample
JJM1203	JJM1203	JJM1203	1	Mouse_Skin	Wild	17247	True Sample
JJM1204	JJM1204	JJM1204	1	Mouse_Skin	Wild	25998	True Sample
JJM1301	JJM1301	JJM1301	1	Mouse_Skin	Wild	16594	True Sample
MC903A1F	MC903.A.1.F	MC903.A.1.F	1	Mouse_Skin	Lab-MPI	11121	True Sample
MJJ0101	MJJ0101	MJJ0101	1	Mouse_Skin	Wild	6514	True Sample
MJJ0102	MJJ0102	MJJ0102	1	Mouse_Skin	Wild	7296	True Sample
MJJ0103	MJJ0103	MJJ0103	1	Mouse_Skin	Wild	18572	True Sample
MJJ0104	MJJ0104	MJJ0104	1	Mouse_Skin	Wild	66061	True Sample
MJJ0105	MJJ0105	MJJ0105	1	Mouse_Skin	Wild	7828	True Sample
MJJ0106	MJJ0106	MJJ0106	1	Mouse_Skin	Wild	11324	True Sample
MJJ0107	MJJ0107	MJJ0107	1	Mouse_Skin	Wild	10511	True Sample
MJJ0108	MJJ0108	MJJ0108	1	Mouse_Skin	Wild	10594	True Sample
MJJ0109	MJJ0109	MJJ0109	1	Mouse_Skin	Wild	20249	True Sample
MJJ0111	MJJ0111	MJJ0111	1	Mouse_Skin	Wild	29581	True Sample
MJJ0112	MJJ0112	MJJ0112	1	Mouse_Skin	Wild	14442	True Sample
MJJ0113	MJJ0113	MJJ0113	1	Mouse_Skin	Wild	10374	True Sample
MJJ0114	MJJ0114	MJJ0114	1	Mouse_Skin	Wild	11249	True Sample
MJJ0115	MJJ0115	MJJ0115	1	Mouse_Skin	Wild	10327	True Sample
MJJ0116	MJJ0116	MJJ0116	1	Mouse_Skin	Wild	16573	True Sample
MJJ0117	MJJ0117	MJJ0117	1	Mouse_Skin	Wild	6760	True Sample
MJJ0301	MJJ0301	MJJ0301	1	Mouse_Skin	Wild	5897	True Sample
MJJ0601	MJJ0601	MJJ0601	1	Mouse_Skin	Wild	18256	True Sample
MJJ0602	MJJ0602	MJJ0602	1	Mouse_Skin	Wild	16341	True Sample
MJJ0603	MJJ0603	MJJ0603	1	Mouse_Skin	Wild	6040	True Sample
MJJ0604	MJJ0604	MJJ0604	1	Mouse_Skin	Wild	9149	True Sample
MJJ0605	MJJ0605	MJJ0605	1	Mouse_Skin	Wild	18890	True Sample
MJJ0606	MJJ0606	MJJ0606	1	Mouse_Skin	Wild	9095	True Sample
MJJ0607	MJJ0607	MJJ0607	1	Mouse_Skin	Wild	9478	True Sample
MJJ0608	MJJ0608	MJJ0608	1	Mouse_Skin	Wild	6911	True Sample
MJJ0609	MJJ0609	MJJ0609	1	Mouse_Skin	Wild	9194	True Sample
MJJ0610	MJJ0610	MJJ0610	1	Mouse_Skin	Wild	12413	True Sample

MJJ0611	MJJ0611	MJJ0611	1	Mouse_Skin	Wild	47802	True Sample
MJJ0701	MJJ0701	MJJ0701	1	Mouse_Skin	Wild	11599	True Sample
MJJ0702	MJJ0702	MJJ0702	1	Mouse_Skin	Wild	9855	True Sample
MJJ0703	MJJ0703	MJJ0703	1	Mouse_Skin	Wild	5156	True Sample
MJJ0901	MJJ0901	MJJ0901	1	Mouse_Skin	Wild	11627	True Sample
MJJ0902	MJJ0902	MJJ0902	1	Mouse_Skin	Wild	12696	True Sample
MJJ1001	MJJ1001	MJJ1001	1	Mouse_Skin	Wild	11323	True Sample
MJJ1002	MJJ1002	MJJ1002	1	Mouse_Skin	Wild	20809	True Sample
MJJ1003	MJJ1003	MJJ1003	1	Mouse_Skin	Wild	18089	True Sample
MJJ1004	MJJ1004	MJJ1004	1	Mouse_Skin	Wild	6076	True Sample
MJJ1005	MJJ1005	MJJ1005	1	Mouse_Skin	Wild	9389	True Sample
MJJ1101	MJJ1101	MJJ1101	1	Mouse_Skin	Wild	14720	True Sample
MM17	MM17	MM17	1	Mouse_Skin	Lab-MPI	21117	True Sample
MM18	MM18	MM18	1	Mouse_Skin	Lab-MPI	9731	True Sample
MM19	MM19	MM19	1	Mouse_Skin	Lab-MPI	8884	True Sample
MM20	MM20	MM20	1	Mouse_Skin	Lab-MPI	12224	True Sample
MM21	MM21	MM21	1	Mouse_Skin	Lab-MPI	17536	True Sample
MM22	MM22	MM22	1	Mouse_Skin	Lab-MPI	4951	True Sample
MM23	MM23	MM23	1	Mouse_Skin	Lab-MPI	12594	True Sample
MM24	MM24	MM24	1	Mouse_Skin	Lab-MPI	11034	True Sample
MM25	MM25	MM25	1	Mouse_Skin	Lab-MPI	28998	True Sample
MM26	MM26	MM26	1	Mouse_Skin	Lab-MPI	17521	True Sample
MM27	MM27	MM27	1	Mouse_Skin	Lab-MPI	3248	True Sample
MN0201	MN0201	MN0201	1	Mouse_Skin	Wild	9932	True Sample
MN0202	MN0202	MN0202	1	Mouse_Skin	Wild	14181	True Sample
MN0203	MN0203	MN0203	1	Mouse_Skin	Wild	5862	True Sample
MN0204	MN0204	MN0204	1	Mouse_Skin	Wild	6717	True Sample
MN0205	MN0205	MN0205	1	Mouse_Skin	Wild	10333	True Sample
MN0206	MN0206	MN0206	1	Mouse_Skin	Wild	16709	True Sample
MN0207	MN0207	MN0207	1	Mouse_Skin	Wild	8541	True Sample
MN0301	MN0301	MN0301	1	Mouse_Skin	Wild	6431	True Sample
MN0302	MN0302	MN0302	1	Mouse_Skin	Wild	6416	True Sample
MN0303	MN0303	MN0303	1	Mouse_Skin	Wild	4750	True Sample
MN0304	MN0304	MN0304	1	Mouse_Skin	Wild	9457	True Sample
MN0305	MN0305	MN0305	1	Mouse_Skin	Wild	21002	True Sample
MN0306	MN0306	MN0306	1	Mouse_Skin	Wild	9225	True Sample
MN0307	MN0307	MN0307	1	Mouse_Skin	Wild	4973	True Sample
MN0308	MN0308	MN0308	1	Mouse_Skin	Wild	5556	True Sample
MN0309	MN0309	MN0309	1	Mouse_Skin	Wild	6133	True Sample
MN0310	MN0310	MN0310	1	Mouse_Skin	Wild	3802	True Sample
MN0311	MN0311	MN0311	1	Mouse_Skin	Wild	17435	True Sample
MN0312	MN0312	MN0312	1	Mouse_Skin	Wild	937	True Sample
MN1202	MN1202	MN1202	1	Mouse_Skin	Wild	9276	True Sample
MN2401	MN2401	MN2401	1	Mouse_Skin	Wild	4929	True Sample
MN2402	MN2402	MN2402	1	Mouse_Skin	Wild	17556	True Sample
MN2601	MN2601	MN2601	1	Mouse_Skin	Wild	7271	True Sample
MN2602	MN2602	MN2602	1	Mouse_Skin	Wild	16516	True Sample
MN2603	MN2603	MN2603	1	Mouse_Skin	Wild	12538	True Sample
MN2605	MN2605	MN2605	1	Mouse_Skin	Wild	9423	True Sample
MN2606	MN2606	MN2606	1	Mouse_Skin	Wild	11553	True Sample
MN2608	MN2608	MN2608	1	Mouse_Skin	Wild	12735	True Sample
MN2609	MN2609	MN2609	1	Mouse_Skin	Wild	9465	True Sample
MN2610	MN2610	MN2610	1	Mouse_Skin	Wild	15943	True Sample
MN2611	MN2611	MN2611	1	Mouse_Skin	Wild	19746	True Sample
MN2613	MN2613	MN2613	1	Mouse_Skin	Wild	16849	True Sample
MN2614	MN2614	MN2614	1	Mouse_Skin	Wild	14397	True Sample
MN2616	MN2616	MN2616	1	Mouse_Skin	Wild	18379	True Sample
MN2901	MN2901	MN2901	1	Mouse_Skin	Wild	10631	True Sample
MN2902	MN2902	MN2902	1	Mouse_Skin	Wild	7494	True Sample
MN3201	MN3201	MN3201	1	Mouse_Skin	Wild	6367	True Sample
MN3202	MN3202	MN3202	1	Mouse_Skin	Wild	15338	True Sample
MN3203	MN3203	MN3203	1	Mouse_Skin	Wild	15491	True Sample
MN3204	MN3204	MN3204	1	Mouse_Skin	Wild	12028	True Sample
MN3205	MN3205	MN3205	1	Mouse_Skin	Wild	19615	True Sample
MN3206	MN3206	MN3206	1	Mouse_Skin	Wild	23507	True Sample
MN3207	MN3207	MN3207	1	Mouse_Skin	Wild	7594	True Sample
MN3208	MN3208	MN3208	1	Mouse_Skin	Wild	24009	True Sample
MN3209	MN3209	MN3209	1	Mouse_Skin	Wild	19718	True Sample
MN3210	MN3210	MN3210	1	Mouse_Skin	Wild	15513	True Sample
MN3211	MN3211	MN3211	1	Mouse_Skin	Wild	20342	True Sample
MN3212	MN3212	MN3212	1	Mouse_Skin	Wild	13948	True Sample
MN3214	MN3214	MN3214	1	Mouse_Skin	Wild	9338	True Sample
MN3215	MN3215	MN3215	1	Mouse_Skin	Wild	22959	True Sample
MN4101	MN4101	MN4101	1	Mouse_Skin	Wild	5229	True Sample
MN4102	MN4102	MN4102	1	Mouse_Skin	Wild	12862	True Sample
MN4103	MN4103	MN4103	1	Mouse_Skin	Wild	11893	True Sample
MN4104	MN4104	MN4104	1	Mouse_Skin	Wild	14203	True Sample
MN4105	MN4105	MN4105	1	Mouse_Skin	Wild	19945	True Sample
MN4106	MN4106	MN4106	1	Mouse_Skin	Wild	24545	True Sample
MN4107	MN4107	MN4107	1	Mouse_Skin	Wild	14129	True Sample
MT0101	MT0101	MT0101	1	Mouse_Skin	Wild	20612	True Sample
MT0102	MT0102	MT0102	1	Mouse_Skin	Wild	13016	True Sample
MT0103	MT0103	MT0103	1	Mouse_Skin	Wild	16992	True Sample
MT0104	MT0104	MT0104	1	Mouse_Skin	Wild	9271	True Sample
MT0105	MT0105	MT0105	1	Mouse_Skin	Wild	13985	True Sample
MT0106	MT0106	MT0106	1	Mouse_Skin	Wild	15797	True Sample
MT0107	MT0107	MT0107	1	Mouse_Skin	Wild	18691	True Sample
MT0108	MT0108	MT0108	1	Mouse_Skin	Wild	22316	True Sample
MT0109	MT0109	MT0109	1	Mouse_Skin	Wild	20454	True Sample
MT0110	MT0110	MT0110	1	Mouse_Skin	Wild	6395	True Sample
MT0111	MT0111	MT0111	1	Mouse_Skin	Wild	9951	True Sample

MT0112	MT0112	MT0112	1	Mouse_Skin	Wild	11109	True Sample
MT0113	MT0113	MT0113	1	Mouse_Skin	Wild	15287	True Sample
MT0114	MT0114	MT0114	1	Mouse_Skin	Wild	14370	True Sample
MT0115	MT0115	MT0115	1	Mouse_Skin	Wild	10715	True Sample
MT0116	MT0116	MT0116	1	Mouse_Skin	Wild	21490	True Sample
MT1301	MT1301	MT1301	1	Mouse_Skin	Wild	12903	True Sample
MT1302	MT1302	MT1302	1	Mouse_Skin	Wild	15238	True Sample
MT1303	MT1303	MT1303	1	Mouse_Skin	Wild	22706	True Sample
MT1304	MT1304	MT1304	1	Mouse_Skin	Wild	19735	True Sample
MT1305	MT1305	MT1305	1	Mouse_Skin	Wild	26871	True Sample
MT1306	MT1306	MT1306	1	Mouse_Skin	Wild	14788	True Sample
MT1307	MT1307	MT1307	1	Mouse_Skin	Wild	21247	True Sample
MT1308	MT1308	MT1308	1	Mouse_Skin	Wild	15568	True Sample
MT1401	MT1401	MT1401	1	Mouse_Skin	Wild	18186	True Sample
MT1402	MT1402	MT1402	1	Mouse_Skin	Wild	8118	True Sample
MT1501	MT1501	MT1501	1	Mouse_Skin	Wild	54057	True Sample
MT1502	MT1502	MT1502	1	Mouse_Skin	Wild	11066	True Sample
MT1503	MT1503	MT1503	1	Mouse_Skin	Wild	11705	True Sample
MT1701	MT1701	MT1701	1	Mouse_Skin	Wild	45796	True Sample
MT1702	MT1702	MT1702	1	Mouse_Skin	Wild	25669	True Sample
MT1704	MT1704	MT1704	1	Mouse_Skin	Wild	10165	True Sample
MT1705	MT1705	MT1705	1	Mouse_Skin	Wild	24522	True Sample
MT1706	MT1706	MT1706	1	Mouse_Skin	Wild	5837	True Sample
MT1707	MT1707	MT1707	1	Mouse_Skin	Wild	18611	True Sample
MT2101	MT2101	MT2101	1	Mouse_Skin	Wild	100000	True Sample
MT2102	MT2102	MT2102	1	Mouse_Skin	Wild	26309	True Sample
MT2103	MT2103	MT2103	1	Mouse_Skin	Wild	18797	True Sample
MT2104	MT2104	MT2104	1	Mouse_Skin	Wild	16618	True Sample
MT2105	MT2105	MT2105	1	Mouse_Skin	Wild	5764	True Sample
MT2106	MT2106	MT2106	1	Mouse_Skin	Wild	16424	True Sample
MT2107	MT2107	MT2107	1	Mouse_Skin	Wild	12490	True Sample
MT2108	MT2108	MT2108	1	Mouse_Skin	Wild	17473	True Sample
MT2109	MT2109	MT2109	1	Mouse_Skin	Wild	12466	True Sample
MT2110	MT2110	MT2110	1	Mouse_Skin	Wild	17054	True Sample
MT2112	MT2112	MT2112	1	Mouse_Skin	Wild	16459	True Sample
MT2113	MT2113	MT2113	1	Mouse_Skin	Wild	16739	True Sample
MT2114	MT2114	MT2114	1	Mouse_Skin	Wild	21742	True Sample
MT2115	MT2115	MT2115	1	Mouse_Skin	Wild	23220	True Sample
MT2116	MT2116	MT2116	1	Mouse_Skin	Wild	27079	True Sample
MT2117	MT2117	MT2117	1	Mouse_Skin	Wild	32636	True Sample
MT2118	MT2118	MT2118	1	Mouse_Skin	Wild	17624	True Sample
MT2120	MT2120	MT2120	1	Mouse_Skin	Wild	22343	True Sample
MT2121	MT2121	MT2121	1	Mouse_Skin	Wild	22517	True Sample
MT2601	MT2601	MT2601	1	Mouse_Skin	Wild	9431	True Sample
MT2603	MT2603	MT2603	1	Mouse_Skin	Wild	8576	True Sample
MT3501	MT3501	MT3501	1	Mouse_Skin	Wild	9654	True Sample
MT3502	MT3502	MT3502	1	Mouse_Skin	Wild	33340	True Sample
MT3503	MT3503	MT3503	1	Mouse_Skin	Wild	13350	True Sample
MT3506	MT3506	MT3506	1	Mouse_Skin	Wild	11973	True Sample
MT3507	MT3507	MT3507	1	Mouse_Skin	Wild	22886	True Sample
MT3508	MT3508	MT3508	1	Mouse_Skin	Wild	18523	True Sample
MT3509	MT3509	MT3509	1	Mouse_Skin	Wild	18544	True Sample
MT3511	MT3511	MT3511	1	Mouse_Skin	Wild	9179	True Sample
MT3513	MT3513	MT3513	1	Mouse_Skin	Wild	521	True Sample
NC1	NC_1	NC1	1	Mouse_Skin	Lab-MPI	479	Control Sample
NC2	NC_2	NC2	1	Mouse_Skin	Lab-MPI	29852	Control Sample
Negativecontrol10	cont_10	Negativecontrol10	1	Mouse_Skin	HL-Lab	496	Control Sample
Negativecontrol11	cont_11	Negativecontrol11	1	Mouse_Skin	HL-Lab	478	Control Sample
Negativecontrol12	cont_12	Negativecontrol12	1	Mouse_Skin	HL-Lab	494	Control Sample
Negativecontrol13	cont_13	Negativecontrol13	1	Mouse_Skin	HL-Lab	478	Control Sample
NegativeControl14	cont_14	NegativeControl14	1	Mouse_Skin	HL-Lab	496	Control Sample
NegativeControl2	cont_2	NegativeControl2	1	Mouse_Skin	HL-Lab	35011	Control Sample
NegativeControl3	cont_3	NegativeControl3	1	Mouse_Skin	HL-Lab	491	Control Sample
NegativeControl4	cont_4	NegativeControl4	1	Mouse_Skin	HL-Lab	487	Control Sample
Negativecontrol5	cont_5	Negativecontrol5	1	Mouse_Skin	HL-Lab	483	Control Sample
Negativecontrol6	cont_6	Negativecontrol6	1	Mouse_Skin	HL-Lab	491	Control Sample
NegCon1ES	Neg_c1	NegCon1ES	1	Mouse_Skin	Wild	7198	Control Sample
NegCont1G15	cont_1	NegCont1G15	1	Mouse_Skin	HL-Lab	470	Control Sample

Supplementary Table 1.6 Metadata of RNA samples used for "Decontam"

Sample_ID	Sample_Analysed	X.SampleID	PlateNumber	Subject	Habitat	quant_reading	Sample_or_Control
8858	8858	8858	G15_P1	Mouse_Skin	HL-Lab	381411	True Sample
8859	8859	8859	G15_P1	Mouse_Skin	HL-Lab	39730	True Sample
8860	8860	8860	G15_P2	Mouse_Skin	HL-Lab	22556	True Sample
8862	8862	8862	G15_P2	Mouse_Skin	HL-Lab	20911	True Sample
8872	8872	8872	G15_P3	Mouse_Skin	HL-Lab	15472	True Sample
8874	8874	8874	G15_P2	Mouse_Skin	HL-Lab	19166	True Sample
8875	8875	8875	G15_P2	Mouse_Skin	HL-Lab	16767	True Sample
8883	8883	8883	G15_P4_Phylosymbiosis_P2	Mouse_Skin	HL-Lab	24158	True Sample
8884	8884	8884	G15_P3	Mouse_Skin	HL-Lab	13592	True Sample
8885	8885	8885	G15_P2	Mouse_Skin	HL-Lab	32093	True Sample
8886	8886	8886	G15_P1	Mouse_Skin	HL-Lab	19191	True Sample
8887	8887	8887	G15_P1	Mouse_Skin	HL-Lab	19830	True Sample
8888	8888	8888	G15_P2	Mouse_Skin	HL-Lab	18966	True Sample
8889	8889	8889	G15_P4_Phylosymbiosis_P2	Mouse_Skin	HL-Lab	6512	True Sample
8894	8894	8894	G15_P4_Phylosymbiosis_P2	Mouse_Skin	HL-Lab	42578	True Sample
8901	8901	8901	G15_P2	Mouse_Skin	HL-Lab	20813	True Sample
8902	8902	8902	G15_P3	Mouse_Skin	HL-Lab	24015	True Sample
8903	8903	8903	G15_P4_Phylosymbiosis_P2	Mouse_Skin	HL-Lab	17331	True Sample
8904	8904	8904	G15_P1	Mouse_Skin	HL-Lab	69580	True Sample
8905	8905	8905	G15_P1	Mouse_Skin	HL-Lab	14711	True Sample
8906	8906	8906	G15_P4_Phylosymbiosis_P2	Mouse_Skin	HL-Lab	10101	True Sample
8907	8907	8907	G15_P2	Mouse_Skin	HL-Lab	10808	True Sample
8908	8908	8908	G15_P4_Phylosymbiosis_P2	Mouse_Skin	HL-Lab	19486	True Sample
8909	8909	8909	G15_P2	Mouse_Skin	HL-Lab	17783	True Sample
8910	8910	8910	G15_P3	Mouse_Skin	HL-Lab	15517	True Sample
8911	8911	8911	G15_P1	Mouse_Skin	HL-Lab	1222	True Sample
8912	8912	8912	G15_P1	Mouse_Skin	HL-Lab	9727	True Sample
8913	8913	8913	G15_P4_Phylosymbiosis_P2	Mouse_Skin	HL-Lab	44086	True Sample
8914	8914	8914	G15_P4_Phylosymbiosis_P2	Mouse_Skin	HL-Lab	18231	True Sample
8915	8915	8915	G15_P1	Mouse_Skin	HL-Lab	184428	True Sample
8924	8924	8924	G15_P3	Mouse_Skin	HL-Lab	14350	True Sample
8925	8925	8925	G15_P2	Mouse_Skin	HL-Lab	51430	True Sample
8926	8926	8926	G15_P4_Phylosymbiosis_P2	Mouse_Skin	HL-Lab	40492	True Sample
8929	8929	8929	G15_P4_Phylosymbiosis_P2	Mouse_Skin	HL-Lab	2973	True Sample
8930	8930	8930	G15_P4_Phylosymbiosis_P2	Mouse_Skin	HL-Lab	17694	True Sample
8931	8931	8931	G15_P4_Phylosymbiosis_P2	Mouse_Skin	HL-Lab	8954	True Sample
8948	8948	8948	G15_P3	Mouse_Skin	HL-Lab	26675	True Sample
8949	8949	8949	G15_P2	Mouse_Skin	HL-Lab	19256	True Sample
8950	8950	8950	G15_P1	Mouse_Skin	HL-Lab	46877	True Sample
8951	8951	8951	G15_P1	Mouse_Skin	HL-Lab	41104	True Sample
8952	8952	8952	G15_P4_Phylosymbiosis_P2	Mouse_Skin	HL-Lab	2721	True Sample
8953	8953	8953	G15_P4_Phylosymbiosis_P2	Mouse_Skin	HL-Lab	14310	True Sample
8954	8954	8954	G15_P3	Mouse_Skin	HL-Lab	6229	True Sample
8955	8955	8955	G15_P2	Mouse_Skin	HL-Lab	207841	True Sample
8962	8962	8962	G15_P1	Mouse_Skin	HL-Lab	10017	True Sample
8963	8963	8963	G15_P1	Mouse_Skin	HL-Lab	42798	True Sample
8964	8964	8964	G15_P4_Phylosymbiosis_P2	Mouse_Skin	HL-Lab	11330	True Sample
8965	8965	8965	G15_P4_Phylosymbiosis_P2	Mouse_Skin	HL-Lab	10944	True Sample
8966	8966	8966	G15_P4_Phylosymbiosis_P2	Mouse_Skin	HL-Lab	9811	True Sample
8967	8967	8967	G15_P3	Mouse_Skin	HL-Lab	14173	True Sample
8968	8968	8968	G15_P2	Mouse_Skin	HL-Lab	28565	True Sample
8969	8969	8969	G15_P2	Mouse_Skin	HL-Lab	25940	True Sample
8970	8970	8970	G15_P1	Mouse_Skin	HL-Lab	8457	True Sample
8971	8971	8971	G15_P1	Mouse_Skin	HL-Lab	38363	True Sample
8972	8972	8972	G15_P4_Phylosymbiosis_P2	Mouse_Skin	HL-Lab	76706	True Sample
8973	8973	8973	G15_P1	Mouse_Skin	HL-Lab	138787	True Sample
8974	8974	8974	G15_P3	Mouse_Skin	HL-Lab	7291	True Sample
8975	8975	8975	G15_P2	Mouse_Skin	HL-Lab	2546	True Sample
8976	8976	8976	G15_P2	Mouse_Skin	HL-Lab	22732	True Sample
8977	8977	8977	G15_P2	Mouse_Skin	HL-Lab	29018	True Sample
8978	8978	8978	G15_P1	Mouse_Skin	HL-Lab	29569	True Sample
8979	8979	8979	G15_P1	Mouse_Skin	HL-Lab	24478	True Sample
8980	8980	8980	G15_P1	Mouse_Skin	HL-Lab	52951	True Sample
8991	8991	8991	G15_P2	Mouse_Skin	HL-Lab	16304	True Sample
8992	8992	8992	G15_P2	Mouse_Skin	HL-Lab	7613	True Sample
8993	8993	8993	G15_P3	Mouse_Skin	HL-Lab	12207	True Sample
8994	8994	8994	G15_P2	Mouse_Skin	HL-Lab	23149	True Sample
8995	8995	8995	G15_P2	Mouse_Skin	HL-Lab	45236	True Sample
8996	8996	8996	G15_P2	Mouse_Skin	HL-Lab	26493	True Sample
8997	8997	8997	G15_P2	Mouse_Skin	HL-Lab	31781	True Sample
8998	8998	8998	G15_P2	Mouse_Skin	HL-Lab	21600	True Sample
8999	8999	8999	G15_P1	Mouse_Skin	HL-Lab	50411	True Sample
9000	9000	9000	G15_P2	Mouse_Skin	HL-Lab	14729	True Sample
9001	9001	9001	G15_P3	Mouse_Skin	HL-Lab	16291	True Sample
9013	9013	9013	G15_P1	Mouse_Skin	HL-Lab	12453	True Sample
9014	9014	9014	G15_P1	Mouse_Skin	HL-Lab	3867	True Sample
9015	9015	9015	G15_P1	Mouse_Skin	HL-Lab	20198	True Sample
9016	9016	9016	G15_P2	Mouse_Skin	HL-Lab	62017	True Sample
9017	9017	9017	G15_P2	Mouse_Skin	HL-Lab	33021	True Sample
9018	9018	9018	G15_P2	Mouse_Skin	HL-Lab	21338	True Sample
9019	9019	9019	G15_P2	Mouse_Skin	HL-Lab	4432	True Sample
9020	9020	9020	G15_P2	Mouse_Skin	HL-Lab	13357	True Sample
9021	9021 A	9021 A	G15_P2	Mouse_Skin	HL-Lab	16078	True Sample
9022	9022	9022	G15_P3	Mouse_Skin	HL-Lab	9736	True Sample
9029	9029	9029	G15_P2	Mouse_Skin	HL-Lab	19069	True Sample
9030	9030	9030	G15_P2	Mouse_Skin	HL-Lab	114340	True Sample
9031	9031	9031	G15_P1	Mouse_Skin	HL-Lab	15297	True Sample
9034	9034	9034	G15_P1	Mouse_Skin	HL-Lab	382786	True Sample

9035	9035	9035	G15_P2	Mouse_Skin	HL-Lab	25678	True Sample
9036	9036	9036	G15_P2	Mouse_Skin	HL-Lab	9807	True Sample
9037	9037	9037	G15_P1	Mouse_Skin	HL-Lab	16479	True Sample
9038	9038	9038	G15_P2	Mouse_Skin	HL-Lab	26809	True Sample
9039	9039	9039	G15_P2	Mouse_Skin	HL-Lab	14616	True Sample
9040	9040	9040	G15_P3	Mouse_Skin	HL-Lab	1811	True Sample
9043	9043	9043	G15_P1	Mouse_Skin	HL-Lab	13660	True Sample
9044	9044	9044	G15_P1	Mouse_Skin	HL-Lab	56869	True Sample
9059	9059	9059	G15_P1	Mouse_Skin	HL-Lab	371641	True Sample
9060	9060	9060	G15_P1	Mouse_Skin	HL-Lab	224557	True Sample
9061	9061	9061	G15_P3	Mouse_Skin	HL-Lab	22182	True Sample
9063	9063	9063	G15_P1	Mouse_Skin	HL-Lab	27060	True Sample
9064	9064	9064	G15_P1	Mouse_Skin	HL-Lab	12199	True Sample
9067	9067	9067	G15_P2	Mouse_Skin	HL-Lab	25538	True Sample
9068	9068	9068	G15_P2	Mouse_Skin	HL-Lab	7098	True Sample
9070	9070	9070	G15_P1	Mouse_Skin	HL-Lab	27532	True Sample
9084	9084	9084	G15_P1	Mouse_Skin	HL-Lab	24181	True Sample
9085	9085	9085	G15_P3	Mouse_Skin	HL-Lab	5376	True Sample
9086	9086	9086	G15_P3	Mouse_Skin	HL-Lab	9353	True Sample
9087	9087	9087	G15_P1	Mouse_Skin	HL-Lab	230725	True Sample
9088	9088	9088	G15_P1	Mouse_Skin	HL-Lab	203082	True Sample
9089	9089	9089	G15_P1	Mouse_Skin	HL-Lab	13680	True Sample
9090	9090	9090	G15_P1	Mouse_Skin	HL-Lab	35234	True Sample
9091	9091	9091	G15_P1	Mouse_Skin	HL-Lab	273795	True Sample
9103	9103	9103	G15_P3	Mouse_Skin	HL-Lab	18225	True Sample
9104	9104	9104	G15_P2	Mouse_Skin	HL-Lab	22326	True Sample
9105	9105	9105	G15_P2	Mouse_Skin	HL-Lab	31573	True Sample
9106	9106	9106	G15_P1	Mouse_Skin	HL-Lab	339191	True Sample
9107	9107	9107	G15_P1	Mouse_Skin	HL-Lab	97844	True Sample
9108	9108	9108	G15_P1	Mouse_Skin	HL-Lab	15714	True Sample
9109	9109	9109	G15_P1	Mouse_Skin	HL-Lab	68841	True Sample
9110	9110	9110	G15_P2	Mouse_Skin	HL-Lab	26710	True Sample
9111	9111	9111	G15_P2	Mouse_Skin	HL-Lab	37800	True Sample
9112	9112	9112	G15_P1	Mouse_Skin	HL-Lab	184599	True Sample
9113	9113	9113	G15_P2	Mouse_Skin	HL-Lab	8660	True Sample
9114	9114	9114	G15_P1	Mouse_Skin	HL-Lab	31116	True Sample
9115	9115	9115	G15_P3	Mouse_Skin	HL-Lab	15954	True Sample
9116	9116	9116	G15_P2	Mouse_Skin	HL-Lab	16027	True Sample
9119	9119	9119	G15_P2	Mouse_Skin	HL-Lab	11508	True Sample
9120	9120	9120	G15_P2	Mouse_Skin	HL-Lab	23765	True Sample
9121	9121	9121	G15_P2	Mouse_Skin	HL-Lab	8360	True Sample
9122	9122	9122	G15_P2	Mouse_Skin	HL-Lab	14140	True Sample
9123	9123	9123	G15_P3	Mouse_Skin	HL-Lab	21948	True Sample
9129	9129	9129	G15_P3	Mouse_Skin	HL-Lab	20488	True Sample
9130	9130	9130	G15_P1	Mouse_Skin	HL-Lab	149837	True Sample
9131	9131	9131	G15_P2	Mouse_Skin	HL-Lab	13800	True Sample
9132	9132	9132	G15_P1	Mouse_Skin	HL-Lab	199261	True Sample
9133	9133	9133	G15_P4_Phylosymbiosis_P2	Mouse_Skin	HL-Lab	89120	True Sample
9134	9134	9134	G15_P1	Mouse_Skin	HL-Lab	180584	True Sample
9135	9135	9135	G15_P4_Phylosymbiosis_P2	Mouse_Skin	HL-Lab	39893	True Sample
9144	9144	9144	G15_P3	Mouse_Skin	HL-Lab	36815	True Sample
9145	9145	9145	G15_P4_Phylosymbiosis_P2	Mouse_Skin	HL-Lab	7019	True Sample
9146	9146	9146	G15_P3	Mouse_Skin	HL-Lab	16104	True Sample
9147	9147	9147	G15_P1	Mouse_Skin	HL-Lab	73406	True Sample
9148	9148	9148	G15_P1	Mouse_Skin	HL-Lab	63883	True Sample
9149	9149	9149	G15_P4_Phylosymbiosis_P2	Mouse_Skin	HL-Lab	18496	True Sample
9150	9150	9150	G15_P1	Mouse_Skin	HL-Lab	25389	True Sample
9151	9151	9151	G15_P4_Phylosymbiosis_P2	Mouse_Skin	HL-Lab	15981	True Sample
9153	9153	9153	G15_P1	Mouse_Skin	HL-Lab	17466	True Sample
9154	9154	9154	G15_P1	Mouse_Skin	HL-Lab	42925	True Sample
9155	9155	9155	G15_P2	Mouse_Skin	HL-Lab	22975	True Sample
9156	9156	9156	G15_P2	Mouse_Skin	HL-Lab	10889	True Sample
9157	9157	9157	G15_P2	Mouse_Skin	HL-Lab	24961	True Sample
9159	9159	9159	G15_P2	Mouse_Skin	HL-Lab	20340	True Sample
9160	9160	9160	G15_P4_Phylosymbiosis_P2	Mouse_Skin	HL-Lab	10821	True Sample
9161	9161	9161	G15_P3	Mouse_Skin	HL-Lab	13654	True Sample
9175	9175	9175	G15_P3	Mouse_Skin	HL-Lab	14326	True Sample
9176	9176	9176	G15_P3	Mouse_Skin	HL-Lab	7302	True Sample
9178	9178	9178	G15_P2	Mouse_Skin	HL-Lab	16487	True Sample
9183	9183	9183	G15_P3	Mouse_Skin	HL-Lab	27290	True Sample
9184	9184	9184	G15_P3	Mouse_Skin	HL-Lab	15506	True Sample
9185	9185	9185	G15_P4_Phylosymbiosis_P2	Mouse_Skin	HL-Lab	18648	True Sample
9186	9186	9186	G15_P3	Mouse_Skin	HL-Lab	24878	True Sample
9187	9187	9187	G15_P3	Mouse_Skin	HL-Lab	9311	True Sample
9189	9189	9189	G15_P1	Mouse_Skin	HL-Lab	419173	True Sample
9190	9190	9190	G15_P1	Mouse_Skin	HL-Lab	372397	True Sample
9191	9191	9191	G15_P3	Mouse_Skin	HL-Lab	20484	True Sample
9193	9193	9193	G15_P2	Mouse_Skin	HL-Lab	18347	True Sample
9205	9205	9205	G15_P3	Mouse_Skin	HL-Lab	14699	True Sample
9206	9206	9206	G15_P3	Mouse_Skin	HL-Lab	12785	True Sample
9207	9207	9207	G15_P4_Phylosymbiosis_P2	Mouse_Skin	HL-Lab	72753	True Sample
9208	9208	9208	G15_P1	Mouse_Skin	HL-Lab	74424	True Sample
9209	9209	9209	G15_P4_Phylosymbiosis_P2	Mouse_Skin	HL-Lab	43900	True Sample
9210	9210	9210	G15_P1	Mouse_Skin	HL-Lab	346004	True Sample
9211	9211	9211	G15_P4_Phylosymbiosis_P2	Mouse_Skin	HL-Lab	29214	True Sample
9212	9212	9212	G15_P2	Mouse_Skin	HL-Lab	20392	True Sample
9213	9213	9213	G15_P2	Mouse_Skin	HL-Lab	3215	True Sample
9214	9214	9214	G15_P3	Mouse_Skin	HL-Lab	16968	True Sample
9215	9215	9215	G15_P4_Phylosymbiosis_P2	Mouse_Skin	HL-Lab	24184	True Sample
9216	9216	9216	G15_P4_Phylosymbiosis_P2	Mouse_Skin	HL-Lab	25347	True Sample
9228	9228	9228	G15_P3	Mouse_Skin	HL-Lab	21219	True Sample

9229	9229	9229	G15_P2	Mouse_Skin	HL-Lab	5352	True Sample
9230	9230	9230	G15_P2	Mouse_Skin	HL-Lab	4226	True Sample
9231	9231	9231	G15_P3	Mouse_Skin	HL-Lab	16684	True Sample
9232	9232	9232	G15_P3	Mouse_Skin	HL-Lab	29778	True Sample
9233	9233	9233	G15_P3	Mouse_Skin	HL-Lab	21966	True Sample
9234	9234	9234	G15_P4_Phylosymbiosis_P2	Mouse_Skin	HL-Lab	6239	True Sample
9235	9235	9235	G15_P4_Phylosymbiosis_P2	Mouse_Skin	HL-Lab	88085	True Sample
9248	9248	9248	G15_P3	Mouse_Skin	HL-Lab	15606	True Sample
9249	9249	9249	G15_P1	Mouse_Skin	HL-Lab	34963	True Sample
9250	9250	9250	G15_P2	Mouse_Skin	HL-Lab	3008	True Sample
9254	9254	9254	G15_P3	Mouse_Skin	HL-Lab	18034	True Sample
9255	9255	9255	G15_P1	Mouse_Skin	HL-Lab	532283	True Sample
9256	9256	9256	G15_P3	Mouse_Skin	HL-Lab	14180	True Sample
9257	9257	9257	G15_P4_Phylosymbiosis_P2	Mouse_Skin	HL-Lab	13576	True Sample
9258	9258	9258	G15_P4_Phylosymbiosis_P2	Mouse_Skin	HL-Lab	13983	True Sample
9259	9259	9259	G15_P3	Mouse_Skin	HL-Lab	29823	True Sample
9260	9260	9260	G15_P2	Mouse_Skin	HL-Lab	4439	True Sample
9262	9262	9262	G15_P3	Mouse_Skin	HL-Lab	16466	True Sample
9263	9263	9263	G15_P3	Mouse_Skin	HL-Lab	19313	True Sample
9264	9264	9264	G15_P4_Phylosymbiosis_P2	Mouse_Skin	HL-Lab	6501	True Sample
9265	9265	9265	G15_P3	Mouse_Skin	HL-Lab	11517	True Sample
9266	9266	9266	G15_P3	Mouse_Skin	HL-Lab	11734	True Sample
9271	9271	9271	G15_P3	Mouse_Skin	HL-Lab	22318	True Sample
9272	9272	9272	G15_P3	Mouse_Skin	HL-Lab	8114	True Sample
9273	9273	9273	G15_P4_Phylosymbiosis_P2	Mouse_Skin	HL-Lab	16777	True Sample
9274	9274	9274	G15_P3	Mouse_Skin	HL-Lab	10821	True Sample
9275	9275	9275	G15_P4_Phylosymbiosis_P2	Mouse_Skin	HL-Lab	5820	True Sample
9281	9281	9281	G15_P3	Mouse_Skin	HL-Lab	32089	True Sample
9282	9282	9282	G15_P1	Mouse_Skin	HL-Lab	26938	True Sample
9283	9283	9283	G15_P1	Mouse_Skin	HL-Lab	134782	True Sample
9284	9284	9284	G15_P3	Mouse_Skin	HL-Lab	34188	True Sample
9285	9285	9285	G15_P3	Mouse_Skin	HL-Lab	74951	True Sample
9286	9286	9286	G15_P3	Mouse_Skin	HL-Lab	2729	True Sample
9287	9287	9287	G15_P1	Mouse_Skin	HL-Lab	83168	True Sample
9300	9300	9300	G15_P3	Mouse_Skin	HL-Lab	26574	True Sample
9301	9301	9301	G15_P3	Mouse_Skin	HL-Lab	10324	True Sample
9302	9302	9302	G15_P1	Mouse_Skin	HL-Lab	141189	True Sample
9303	9303	9303	G15_P3	Mouse_Skin	HL-Lab	18132	True Sample
9304	9304	9304	G15_P3	Mouse_Skin	HL-Lab	18592	True Sample
9318	9318	9318	G15_P3	Mouse_Skin	HL-Lab	6547	True Sample
9319	9319	9319	G15_P2	Mouse_Skin	HL-Lab	18211	True Sample
9320	9320	9320	G15_P2	Mouse_Skin	HL-Lab	6580	True Sample
9321	9321	9321	G15_P1	Mouse_Skin	HL-Lab	267435	True Sample
9322	9322	9322	G15_P3	Mouse_Skin	HL-Lab	19483	True Sample
9323	9323	9323	G15_P3	Mouse_Skin	HL-Lab	21107	True Sample
9324	9324	9324	G15_P1	Mouse_Skin	HL-Lab	34770	True Sample
9336	9336	9336	G15_P1	Mouse_Skin	HL-Lab	115461	True Sample
9337	9337	9337	G15_P3	Mouse_Skin	HL-Lab	13587	True Sample
9338	9338	9338	G15_P3	Mouse_Skin	HL-Lab	12584	True Sample
9339	9339	9339	G15_P3	Mouse_Skin	HL-Lab	23605	True Sample
9340	9340	9340	G15_P3	Mouse_Skin	HL-Lab	21918	True Sample
9341	9341	9341	G15_P3	Mouse_Skin	HL-Lab	8715	True Sample
9342	9342	9342	G15_P4_Phylosymbiosis_P2	Mouse_Skin	HL-Lab	35692	True Sample
9343	9343	9343	G15_P1	Mouse_Skin	HL-Lab	140448	True Sample
9344	9344	9344	G15_P2	Mouse_Skin	HL-Lab	18401	True Sample
9345	9345	9345	G15_P2	Mouse_Skin	HL-Lab	8092	True Sample
9346	9346	9346	G15_P3	Mouse_Skin	HL-Lab	23153	True Sample
9352	9352	9352	G15_P2	Mouse_Skin	HL-Lab	50	True Sample
9353	9353	9353	G15_P2	Mouse_Skin	HL-Lab	7759	True Sample
9354	9354	9354	G15_P3	Mouse_Skin	HL-Lab	19611	True Sample
9355	9355	9355	G15_P3	Mouse_Skin	HL-Lab	11233	True Sample
9356	9356	9356	G15_P3	Mouse_Skin	HL-Lab	31318	True Sample
9357	9357	9357	G15_P1	Mouse_Skin	HL-Lab	55031	True Sample
9359	9359	9359	G15_P1	Mouse_Skin	HL-Lab	45487	True Sample
9360	9360	9360	G15_P1	Mouse_Skin	HL-Lab	142884	True Sample
9365	9365	9365	G15_P3	Mouse_Skin	HL-Lab	26515	True Sample
9366	9366	9366	G15_P4_Phylosymbiosis_P2	Mouse_Skin	HL-Lab	6229	True Sample
9367	9367	9367	G15_P4_Phylosymbiosis_P2	Mouse_Skin	HL-Lab	57069	True Sample
9368	9368	9368	G15_P1	Mouse_Skin	HL-Lab	63192	True Sample
9375	9375	9375	G15_P3	Mouse_Skin	HL-Lab	11337	True Sample
9376	9376	9376	G15_P1	Mouse_Skin	HL-Lab	189001	True Sample
9377	9377	9377	G15_P1	Mouse_Skin	HL-Lab	186227	True Sample
9380	9380	9380	G15_P2	Mouse_Skin	HL-Lab	16453	True Sample
9381	9381	9381	G15_P2	Mouse_Skin	HL-Lab	7718	True Sample
9382	9382	9382	G15_P2	Mouse_Skin	HL-Lab	15035	True Sample
9388	9388	9388	G15_P1	Mouse_Skin	HL-Lab	130517	True Sample
9389	9389	9389	G15_P3	Mouse_Skin	HL-Lab	23611	True Sample
9390	9390	9390	G15_P3	Mouse_Skin	HL-Lab	17374	True Sample
9391	9391	9391	G15_P3	Mouse_Skin	HL-Lab	16257	True Sample
9392	9392	9392	G15_P2	Mouse_Skin	HL-Lab	13059	True Sample
9393	9393	9393	G15_P2	Mouse_Skin	HL-Lab	4065	True Sample
9394	9394	9394	G15_P2	Mouse_Skin	HL-Lab	14223	True Sample
9405	9405	9405	G15_P1	Mouse_Skin	HL-Lab	46987	True Sample
9406	9406	9406	G15_P1	Mouse_Skin	HL-Lab	50103	True Sample
9407	9407	9407	G15_P3	Mouse_Skin	HL-Lab	11311	True Sample
9408	9408	9408	G15_P1	Mouse_Skin	HL-Lab	215999	True Sample
9409	9409	9409	G15_P2	Mouse_Skin	HL-Lab	5344	True Sample
9410	9410	9410	G15_P3	Mouse_Skin	HL-Lab	16433	True Sample
9411	9411	9411	G15_P3	Mouse_Skin	HL-Lab	29502	True Sample
9412	9412	9412	G15_P2	Mouse_Skin	HL-Lab	4874	True Sample
9416	9416	9416	G15_P3	Mouse_Skin	HL-Lab	18835	True Sample

9417	9417	9417	G15_P1	Mouse_Skin	HL-Lab	15644	True Sample
9418	9418	9418	G15_P2	Mouse_Skin	HL-Lab	4992	True Sample
9429	9429	9429	G15_P2	Mouse_Skin	HL-Lab	6951	True Sample
9430	9430	9430	G15_P1	Mouse_Skin	HL-Lab	138157	True Sample
9431	9431	9431	G15_P1	Mouse_Skin	HL-Lab	245141	True Sample
9432	9432	9432	G15_P3	Mouse_Skin	HL-Lab	11879	True Sample
9433	9433	9433	G15_P2	Mouse_Skin	HL-Lab	18938	True Sample
9435	9435	9435	G15_P2	Mouse_Skin	HL-Lab	9301	True Sample
9436	9436	9436	G15_P1	Mouse_Skin	HL-Lab	139682	True Sample
9437	9437	9437	G15_P1	Mouse_Skin	HL-Lab	108113	True Sample
9438	9438	9438	G15_P1	Mouse_Skin	HL-Lab	95308	True Sample
9439	9439	9439	G15_P3	Mouse_Skin	HL-Lab	24455	True Sample
9440	9440	9440	G15_P3	Mouse_Skin	HL-Lab	39053	True Sample
9441	9441	9441	G15_P2	Mouse_Skin	HL-Lab	8610	True Sample
9442	9442	9442	G15_P3	Mouse_Skin	HL-Lab	19518	True Sample
9443	9443	9443	G15_P3	Mouse_Skin	HL-Lab	24801	True Sample
9444	9444	9444	G15_P1	Mouse_Skin	HL-Lab	106945	True Sample
9448	9448	9448	G15_P2	Mouse_Skin	HL-Lab	9573	True Sample
9462	9462	9462	G15_P3	Mouse_Skin	HL-Lab	135541	True Sample
9463	9463	9463	G15_P3	Mouse_Skin	HL-Lab	14622	True Sample
9464	9464	9464	G15_P1	Mouse_Skin	HL-Lab	159920	True Sample
9465	9465	9465	G15_P2	Mouse_Skin	HL-Lab	24488	True Sample
9466	9466	9466	G15_P3	Mouse_Skin	HL-Lab	16682	True Sample
9471	9471	9471	G15_P3	Mouse_Skin	HL-Lab	17115	True Sample
9472	9472	9472	G15_P3	Mouse_Skin	HL-Lab	70377	True Sample
9473	9473	9473	G15_P1	Mouse_Skin	HL-Lab	110612	True Sample
9474	9474	9474	G15_P1	Mouse_Skin	HL-Lab	285687	True Sample
9475	9475	9475	G15_P3	Mouse_Skin	HL-Lab	61540	True Sample
9480	9480	9480	G15_P3	Mouse_Skin	HL-Lab	11860	True Sample
9482	9482	9482	G15_P1	Mouse_Skin	HL-Lab	195935	True Sample
50011121	50011121	50011121	G15_P4_Phylosymbiosis_P2	Mouse_Skin	MPI-Lab	3864	True Sample
50011126	50011126	50011126	Phylosymbiosis_P1	Mouse_Skin	MPI-Lab	4670	True Sample
50013455	50013455	50013455	G15_P4_Phylosymbiosis_P2	Mouse_Skin	MPI-Lab	11650	True Sample
50013456	50013456	50013456	G15_P4_Phylosymbiosis_P2	Mouse_Skin	MPI-Lab	11489	True Sample
50013466	50013466	50013466	G15_P4_Phylosymbiosis_P2	Mouse_Skin	MPI-Lab	8331	True Sample
50013641	50013641	50013641	G15_P4_Phylosymbiosis_P2	Mouse_Skin	MPI-Lab	16930	True Sample
50013642	50013642	50013642	G15_P4_Phylosymbiosis_P2	Mouse_Skin	MPI-Lab	12997	True Sample
50013823	50013823	50013823	G15_P4_Phylosymbiosis_P2	Mouse_Skin	MPI-Lab	18290	True Sample
50019000	50019000	50019000	Phylosymbiosis_P1	Mouse_Skin	MPI-Lab	435	True Sample
50019001	50019001	50019001	Phylosymbiosis_P1	Mouse_Skin	MPI-Lab	5331	True Sample
50019002	50019002	50019002	Phylosymbiosis_P1	Mouse_Skin	MPI-Lab	50	True Sample
50019003	50019003	50019003	Phylosymbiosis_P1	Mouse_Skin	MPI-Lab	11140	True Sample
50019004	50019004	50019004	Phylosymbiosis_P1	Mouse_Skin	MPI-Lab	3462	True Sample
50019005	50019005	50019005	Phylosymbiosis_P1	Mouse_Skin	MPI-Lab	3788	True Sample
50019007	50019007	50019007	Phylosymbiosis_P1	Mouse_Skin	MPI-Lab	7148	True Sample
50019008	50019008	50019008	Phylosymbiosis_P1	Mouse_Skin	MPI-Lab	6427	True Sample
50019009	50019009	50019009	Phylosymbiosis_P1	Mouse_Skin	MPI-Lab	10830	True Sample
9021_B	9021 B	9021 B	G15_P3	Mouse_Skin	HL-Lab	14939	True Sample
B11	B11RNA	B11RNA	Espelette_P3_B6IL10	Mouse_Skin	C57BL/6J	42715	True Sample
B12	B12RNA	B12RNA	Espelette_P3_B6IL10	Mouse_Skin	C57BL/6J	39699	True Sample
B13	B13RNA	B13RNA	Espelette_P3_B6IL10	Mouse_Skin	C57BL/6J	35613	True Sample
B14	B14RNA	B14RNA	Espelette_P3_B6IL10	Mouse_Skin	C57BL/6J	41658	True Sample
B61	B61RNA	B61RNA	Espelette_P3_B6IL10	Mouse_Skin	C57BL/6J	47550	True Sample
B610	B610RNA	B610RNA	Espelette_P3_B6IL10	Mouse_Skin	C57BL/6J	19715	True Sample
B62	B62RNA	B62RNA	Espelette_P3_B6IL10	Mouse_Skin	C57BL/6J	27322	True Sample
B63	B63RNA	B63RNA	Espelette_P3_B6IL10	Mouse_Skin	C57BL/6J	30881	True Sample
B64	B64RNA	B64RNA	Espelette_P3_B6IL10	Mouse_Skin	C57BL/6J	29755	True Sample
B65	B65RNA	B65RNA	Espelette_P3_B6IL10	Mouse_Skin	C57BL/6J	42058	True Sample
B66	B66RNA	B66RNA	Espelette_P3_B6IL10	Mouse_Skin	C57BL/6J	49181	True Sample
B67	B67RNA	B67RNA	Espelette_P3_B6IL10	Mouse_Skin	C57BL/6J	41983	True Sample
B68	B68RNA	B68RNA	Espelette_P3_B6IL10	Mouse_Skin	C57BL/6J	6789	True Sample
B69	B69RNA	B69RNA	Espelette_P3_B6IL10	Mouse_Skin	C57BL/6J	27963	True Sample
B6Negextr	B6NegextrRNA	B6NegextrRNA	Espelette_P3_B6IL10	Mouse_Skin	C57BL/6J	15087	Control Sample
Control_10	Control 10	Control 10	G15_P3	Mouse_Skin	HL-Lab	5154	Control Sample
Control_7	Control 7	Control 7	G15_P2	Mouse_Skin	HL-Lab	15490	Control Sample
ctr2	NC_plate2	NC_plate2	G15_P4_Phylosymbiosis_P2	Mouse_Skin	MPI-Lab	7992	Control Sample
EnCJan	EnC_Jan	EnC_Jan	Espelette_P1	Mouse_Skin	Wild	1128	Control Sample
EnCMeriem	EnC_Meriem	EnC_Meriem	Espelette_P2	Mouse_Skin	Wild	50	Control Sample
JJM0101	JJM0101	JJM0101	Espelette_P3_B6IL10	Mouse_Skin	Wild	59613	True Sample
JJM0102	JJM0102	JJM0102	Espelette_P3_B6IL10	Mouse_Skin	Wild	39558	True Sample
JJM0202	JJM0202	JJM0202	Espelette_P3_B6IL10	Mouse_Skin	Wild	30347	True Sample
JJM0203A	JJM0203A	JJM0203A	Espelette_P3_B6IL10	Mouse_Skin	Wild	34436	True Sample
JJM0203B	JJM0203B	JJM0203B	Espelette_P3_B6IL10	Mouse_Skin	Wild	25244	True Sample
JJM0204	JJM0204	JJM0204	Espelette_P3_B6IL10	Mouse_Skin	Wild	30494	True Sample
JJM0205	JJM0205	JJM0205	Espelette_P3_B6IL10	Mouse_Skin	Wild	25058	True Sample
JJM0206	JJM0206	JJM0206	Espelette_P3_B6IL10	Mouse_Skin	Wild	7142	True Sample
JJM0207	JJM0207	JJM0207	Espelette_P3_B6IL10	Mouse_Skin	Wild	48745	True Sample
JJM0208	JJM0208	JJM0208	Espelette_P3_B6IL10	Mouse_Skin	Wild	374602	True Sample
JJM0209	JJM0209	JJM0209	Espelette_P3_B6IL10	Mouse_Skin	Wild	8482	True Sample
JJM0210	JJM0210	JJM0210	Espelette_P3_B6IL10	Mouse_Skin	Wild	12233	True Sample
JJM0401	JJM0401	JJM0401	Espelette_P3_B6IL10	Mouse_Skin	Wild	31788	True Sample
JJM0402	JJM0402	JJM0402	Espelette_P3_B6IL10	Mouse_Skin	Wild	63567	True Sample
JJM0501	JJM0501	JJM0501	Espelette_P3_B6IL10	Mouse_Skin	Wild	24919	True Sample
JJM0502	JJM0502	JJM0502	Espelette_P3_B6IL10	Mouse_Skin	Wild	31999	True Sample
JJM0503	JJM0503	JJM0503	Espelette_P3_B6IL10	Mouse_Skin	Wild	17015	True Sample
JJM0504	JJM0504	JJM0504	Espelette_P3_B6IL10	Mouse_Skin	Wild	18317	True Sample
JJM0601	JJM0601	JJM0601	Espelette_P3_B6IL10	Mouse_Skin	Wild	23802	True Sample
JJM0602	JJM0602	JJM0602	Espelette_P3_B6IL10	Mouse_Skin	Wild	21632	True Sample
JJM0603	JJM0603	JJM0603	Espelette_P3_B6IL10	Mouse_Skin	Wild	4130	True Sample
JJM0604	JJM0604	JJM0604	Espelette_P3_B6IL10	Mouse_Skin	Wild	6340	True Sample
JJM0701	JJM0701	JJM0701	Espelette_P3_B6IL10	Mouse_Skin	Wild	32817	True Sample

JJM0702	JJM0702	JJM0702	Espelette_P2	Mouse_Skin	Wild	285633	True Sample
JJM0801	JJM0801	JJM0801	Espelette_P2	Mouse_Skin	Wild	125543	True Sample
JJM0802	JJM0802	JJM0802	Espelette_P2	Mouse_Skin	Wild	59320	True Sample
JJM0901	JJM0901	JJM0901	Espelette_P2	Mouse_Skin	Wild	51568	True Sample
JJM0902	JJM0902	JJM0902	Espelette_P2	Mouse_Skin	Wild	79312	True Sample
JJM0903	JJM0903	JJM0903	Espelette_P2	Mouse_Skin	Wild	46782	True Sample
JJM0904	JJM0904	JJM0904	Espelette_P2	Mouse_Skin	Wild	150321	True Sample
JJM0905	JJM0905	JJM0905	Espelette_P2	Mouse_Skin	Wild	251522	True Sample
JJM0906	JJM0906	JJM0906	Espelette_P2	Mouse_Skin	Wild	333814	True Sample
JJM0908	JJM0908	JJM0908	Espelette_P2	Mouse_Skin	Wild	35513	True Sample
JJM0909	JJM0909	JJM0909	Espelette_P2	Mouse_Skin	Wild	35089	True Sample
JJM0910	JJM0910	JJM0910	Espelette_P2	Mouse_Skin	Wild	78385	True Sample
JJM0911	JJM0911	JJM0911	Espelette_P2	Mouse_Skin	Wild	60651	True Sample
JJM0912	JJM0912	JJM0912	Espelette_P2	Mouse_Skin	Wild	99317	True Sample
JJM1001	JJM1001	JJM1001	Espelette_P2	Mouse_Skin	Wild	259899	True Sample
JJM1002	JJM1002	JJM1002	Espelette_P2	Mouse_Skin	Wild	60672	True Sample
JJM1201	JJM1201	JJM1201	Espelette_P2	Mouse_Skin	Wild	92951	True Sample
JJM1202	JJM1202	JJM1202	Espelette_P2	Mouse_Skin	Wild	166497	True Sample
JJM1203	JJM1203	JJM1203	Espelette_P2	Mouse_Skin	Wild	52653	True Sample
JJM1204	JJM1204	JJM1204	Espelette_P2	Mouse_Skin	Wild	68456	True Sample
JJM1301	JJM1301	JJM1301	Espelette_P2	Mouse_Skin	Wild	41202	True Sample
MC903A1F	MC903_A_1_F	MC903_A_1_F	G15_P4_Phylosymbiosis_P2	Mouse_Skin	MPI-Lab	6505	True Sample
MJJ0101	MJJ0101	MJJ0101	Espelette_P2	Mouse_Skin	Wild	943	True Sample
MJJ0102	MJJ0102	MJJ0102	Espelette_P2	Mouse_Skin	Wild	62867	True Sample
MJJ0103	MJJ0103	MJJ0103	Espelette_P2	Mouse_Skin	Wild	22982	True Sample
MJJ0104	MJJ0104	MJJ0104	Espelette_P2	Mouse_Skin	Wild	48111	True Sample
MJJ0105	MJJ0105	MJJ0105	Espelette_P2	Mouse_Skin	Wild	42576	True Sample
MJJ0106	MJJ0106	MJJ0106	Espelette_P2	Mouse_Skin	Wild	60210	True Sample
MJJ0107	MJJ0107	MJJ0107	Espelette_P2	Mouse_Skin	Wild	26516	True Sample
MJJ0108	MJJ0108	MJJ0108	Espelette_P2	Mouse_Skin	Wild	33276	True Sample
MJJ0109	MJJ0109	MJJ0109	Espelette_P2	Mouse_Skin	Wild	57555	True Sample
MJJ0111	MJJ0111	MJJ0111	Espelette_P2	Mouse_Skin	Wild	57626	True Sample
MJJ0112	MJJ0112	MJJ0112	Espelette_P2	Mouse_Skin	Wild	51057	True Sample
MJJ0113	MJJ0113	MJJ0113	Espelette_P2	Mouse_Skin	Wild	32382	True Sample
MJJ0114	MJJ0114	MJJ0114	Espelette_P2	Mouse_Skin	Wild	18983	True Sample
MJJ0115	MJJ0115	MJJ0115	Espelette_P2	Mouse_Skin	Wild	112078	True Sample
MJJ0116	MJJ0116	MJJ0116	Espelette_P2	Mouse_Skin	Wild	51422	True Sample
MJJ0117	MJJ0117	MJJ0117	Espelette_P2	Mouse_Skin	Wild	29020	True Sample
MJJ0301	MJJ0301	MJJ0301	Espelette_P2	Mouse_Skin	Wild	8849	True Sample
MJJ0601	MJJ0601	MJJ0601	Espelette_P2	Mouse_Skin	Wild	34748	True Sample
MJJ0602	MJJ0602	MJJ0602	Espelette_P2	Mouse_Skin	Wild	71991	True Sample
MJJ0603	MJJ0603	MJJ0603	Espelette_P2	Mouse_Skin	Wild	26566	True Sample
MJJ0604	MJJ0604	MJJ0604	Espelette_P2	Mouse_Skin	Wild	34133	True Sample
MJJ0605	MJJ0605	MJJ0605	Espelette_P2	Mouse_Skin	Wild	125217	True Sample
MJJ0606	MJJ0606	MJJ0606	Espelette_P2	Mouse_Skin	Wild	16568	True Sample
MJJ0607	MJJ0607	MJJ0607	Espelette_P2	Mouse_Skin	Wild	31893	True Sample
MJJ0608	MJJ0608	MJJ0608	Espelette_P2	Mouse_Skin	Wild	42825	True Sample
MJJ0609	MJJ0609	MJJ0609	Espelette_P2	Mouse_Skin	Wild	56925	True Sample
MJJ0610	MJJ0610	MJJ0610	Espelette_P2	Mouse_Skin	Wild	31047	True Sample
MJJ0611	MJJ0611	MJJ0611	Espelette_P2	Mouse_Skin	Wild	51339	True Sample
MJJ0701	MJJ0701	MJJ0701	Espelette_P2	Mouse_Skin	Wild	25041	True Sample
MJJ0702	MJJ0702	MJJ0702	Espelette_P2	Mouse_Skin	Wild	67857	True Sample
MJJ0703	MJJ0703	MJJ0703	Espelette_P2	Mouse_Skin	Wild	3262	True Sample
MJJ0901	MJJ0901	MJJ0901	Espelette_P2	Mouse_Skin	Wild	40060	True Sample
MJJ0902	MJJ0902	MJJ0902	Espelette_P2	Mouse_Skin	Wild	68095	True Sample
MJJ1001	MJJ1001	MJJ1001	Espelette_P2	Mouse_Skin	Wild	98998	True Sample
MJJ1002	MJJ1002	MJJ1002	Espelette_P2	Mouse_Skin	Wild	96082	True Sample
MJJ1003	MJJ1003	MJJ1003	Espelette_P2	Mouse_Skin	Wild	48609	True Sample
MJJ1004	MJJ1004	MJJ1004	Espelette_P2	Mouse_Skin	Wild	8611	True Sample
MJJ1005	MJJ1005	MJJ1005	Espelette_P2	Mouse_Skin	Wild	9484	True Sample
MJJ1101	MJJ1101	MJJ1101	Espelette_P2	Mouse_Skin	Wild	115451	True Sample
MM17	MM17	MM17	G15_P4_Phylosymbiosis_P2	Mouse_Skin	MPI-Lab	13888	True Sample
MM18	MM18	MM18	G15_P4_Phylosymbiosis_P2	Mouse_Skin	MPI-Lab	23125	True Sample
MM19	MM19	MM19	G15_P4_Phylosymbiosis_P2	Mouse_Skin	MPI-Lab	2917	True Sample
MM20	MM20	MM20	G15_P4_Phylosymbiosis_P2	Mouse_Skin	MPI-Lab	3392	True Sample
MM21	MM21	MM21	G15_P4_Phylosymbiosis_P2	Mouse_Skin	MPI-Lab	3196	True Sample
MM22	MM22	MM22	G15_P4_Phylosymbiosis_P2	Mouse_Skin	MPI-Lab	11311	True Sample
MM23	MM23	MM23	G15_P4_Phylosymbiosis_P2	Mouse_Skin	MPI-Lab	10359	True Sample
MM24	MM24	MM24	G15_P4_Phylosymbiosis_P2	Mouse_Skin	MPI-Lab	12770	True Sample
MM25	MM25	MM25	G15_P4_Phylosymbiosis_P2	Mouse_Skin	MPI-Lab	8785	True Sample
MM26	MM26	MM26	G15_P4_Phylosymbiosis_P2	Mouse_Skin	MPI-Lab	9774	True Sample
MM27	MM27	MM27	G15_P4_Phylosymbiosis_P2	Mouse_Skin	MPI-Lab	4955	True Sample
MN0201	MN0201	MN0201	Espelette_P2	Mouse_Skin	Wild	32942	True Sample
MN0202	MN0202	MN0202	Espelette_P2	Mouse_Skin	Wild	6235	True Sample
MN0203	MN0203	MN0203	Espelette_P2	Mouse_Skin	Wild	22928	True Sample
MN0204	MN0204	MN0204	Espelette_P2	Mouse_Skin	Wild	13010	True Sample
MN0205	MN0205	MN0205	Espelette_P2	Mouse_Skin	Wild	46381	True Sample
MN0206	MN0206	MN0206	Espelette_P2	Mouse_Skin	Wild	26167	True Sample
MN0207	MN0207	MN0207	Espelette_P2	Mouse_Skin	Wild	42868	True Sample
MN0301	MN0301	MN0301	Espelette_P2	Mouse_Skin	Wild	16920	True Sample
MN0302	MN0302	MN0302	Espelette_P2	Mouse_Skin	Wild	9689	True Sample
MN0303	MN0303	MN0303	Espelette_P2	Mouse_Skin	Wild	13029	True Sample
MN0304	MN0304	MN0304	Espelette_P2	Mouse_Skin	Wild	23204	True Sample
MN0305	MN0305	MN0305	Espelette_P2	Mouse_Skin	Wild	70529	True Sample
MN0306	MN0306	MN0306	Espelette_P2	Mouse_Skin	Wild	29538	True Sample
MN0307	MN0307	MN0307	Espelette_P2	Mouse_Skin	Wild	50	True Sample
MN0308	MN0308	MN0308	Espelette_P2	Mouse_Skin	Wild	37201	True Sample
MN0309	MN0309	MN0309	Espelette_P2	Mouse_Skin	Wild	12745	True Sample
MN0310	MN0310	MN0310	Espelette_P2	Mouse_Skin	Wild	11738	True Sample
MN0311	MN0311	MN0311	Espelette_P2	Mouse_Skin	Wild	76655	True Sample
MN0312	MN0312	MN0312	Espelette_P2	Mouse_Skin	Wild	43833	True Sample

MN1201	MN1201	MN1201	Espelette_P2	Mouse_Skin	Wild	6371	True Sample
MN1202	MN1202	MN1202	Espelette_P2	Mouse_Skin	Wild	12929	True Sample
MN2401	MN2401	MN2401	Espelette_P2	Mouse_Skin	Wild	4073	True Sample
MN2402	MN2402	MN2402	Espelette_P2	Mouse_Skin	Wild	113735	True Sample
MN2601	MN2601	MN2601	Espelette_P2	Mouse_Skin	Wild	658	True Sample
MN2602	MN2602	MN2602	Espelette_P2	Mouse_Skin	Wild	56519	True Sample
MN2603	MN2603	MN2603	Espelette_P2	Mouse_Skin	Wild	10289	True Sample
MN2604	MN2604	MN2604	Espelette_P2	Mouse_Skin	Wild	69155	True Sample
MN2605	MN2605	MN2605	Espelette_P2	Mouse_Skin	Wild	56922	True Sample
MN2606	MN2606	MN2606	Espelette_P2	Mouse_Skin	Wild	242956	True Sample
MN2608	MN2608	MN2608	Espelette_P2	Mouse_Skin	Wild	50	True Sample
MN2609	MN2609	MN2609	Espelette_P2	Mouse_Skin	Wild	8513	True Sample
MN2610	MN2610	MN2610	Espelette_P2	Mouse_Skin	Wild	110356	True Sample
MN2611	MN2611	MN2611	Espelette_P2	Mouse_Skin	Wild	240701	True Sample
MN2612	MN2612	MN2612	Espelette_P2	Mouse_Skin	Wild	63098	True Sample
MN2613	MN2613	MN2613	Espelette_P2	Mouse_Skin	Wild	4444	True Sample
MN2614	MN2614	MN2614	Espelette_P1	Mouse_Skin	Wild	57613	True Sample
MN2615	MN2615	MN2615	Espelette_P1	Mouse_Skin	Wild	62259	True Sample
MN2616	MN2616	MN2616	Espelette_P1	Mouse_Skin	Wild	95325	True Sample
MN2901	MN2901	MN2901	Espelette_P1	Mouse_Skin	Wild	70225	True Sample
MN2902	MN2902	MN2902	Espelette_P1	Mouse_Skin	Wild	10512	True Sample
MN3201	MN3201	MN3201	Espelette_P1	Mouse_Skin	Wild	9540	True Sample
MN3202	MN3202	MN3202	Espelette_P1	Mouse_Skin	Wild	102097	True Sample
MN3203	MN3203	MN3203	Espelette_P1	Mouse_Skin	Wild	10310	True Sample
MN3204	MN3204	MN3204	Espelette_P1	Mouse_Skin	Wild	90178	True Sample
MN3205	MN3205	MN3205	Espelette_P1	Mouse_Skin	Wild	50129	True Sample
MN3206	MN3206	MN3206	Espelette_P1	Mouse_Skin	Wild	55020	True Sample
MN3207	MN3207	MN3207	Espelette_P1	Mouse_Skin	Wild	41996	True Sample
MN3208	MN3208	MN3208	Espelette_P1	Mouse_Skin	Wild	59976	True Sample
MN3209	MN3209	MN3209	Espelette_P1	Mouse_Skin	Wild	81255	True Sample
MN3210	MN3210	MN3210	Espelette_P1	Mouse_Skin	Wild	130225	True Sample
MN3211	MN3211	MN3211	Espelette_P1	Mouse_Skin	Wild	60906	True Sample
MN3212	MN3212	MN3212	Espelette_P1	Mouse_Skin	Wild	65709	True Sample
MN3213	MN3213	MN3213	Espelette_P1	Mouse_Skin	Wild	105516	True Sample
MN3214	MN3214	MN3214	Espelette_P1	Mouse_Skin	Wild	29984	True Sample
MN3215	MN3215	MN3215	Espelette_P1	Mouse_Skin	Wild	91332	True Sample
MN4101	MN4101	MN4101	Espelette_P1	Mouse_Skin	Wild	33049	True Sample
MN4102	MN4102	MN4102	Espelette_P1	Mouse_Skin	Wild	24247	True Sample
MN4103	MN4103	MN4103	Espelette_P1	Mouse_Skin	Wild	26560	True Sample
MN4104	MN4104	MN4104	Espelette_P1	Mouse_Skin	Wild	206184	True Sample
MN4105	MN4105	MN4105	Espelette_P1	Mouse_Skin	Wild	134535	True Sample
MN4106	MN4106	MN4106	Espelette_P1	Mouse_Skin	Wild	139036	True Sample
MN4107	MN4107	MN4107	Espelette_P1	Mouse_Skin	Wild	53070	True Sample
MT0101	MT0101	MT0101	Espelette_P1	Mouse_Skin	Wild	118996	True Sample
MT0102	MT0102	MT0102	Espelette_P1	Mouse_Skin	Wild	117477	True Sample
MT0103	MT0103	MT0103	Espelette_P1	Mouse_Skin	Wild	121042	True Sample
MT0104	MT0104	MT0104	Espelette_P1	Mouse_Skin	Wild	52182	True Sample
MT0105	MT0105	MT0105	Espelette_P1	Mouse_Skin	Wild	76276	True Sample
MT0106	MT0106	MT0106	Espelette_P1	Mouse_Skin	Wild	51249	True Sample
MT0107	MT0107	MT0107	Espelette_P1	Mouse_Skin	Wild	85594	True Sample
MT0108	MT0108	MT0108	Espelette_P1	Mouse_Skin	Wild	136878	True Sample
MT0109	MT0109	MT0109	Espelette_P1	Mouse_Skin	Wild	85777	True Sample
MT0110	MT0110	MT0110	Espelette_P1	Mouse_Skin	Wild	14250	True Sample
MT0111	MT0111	MT0111	Espelette_P1	Mouse_Skin	Wild	88463	True Sample
MT0112	MT0112	MT0112	Espelette_P1	Mouse_Skin	Wild	58233	True Sample
MT0113	MT0113	MT0113	Espelette_P1	Mouse_Skin	Wild	75731	True Sample
MT0114	MT0114	MT0114	Espelette_P1	Mouse_Skin	Wild	44985	True Sample
MT0115	MT0115	MT0115	Espelette_P1	Mouse_Skin	Wild	42234	True Sample
MT0116	MT0116	MT0116	Espelette_P1	Mouse_Skin	Wild	92722	True Sample
MT1301	MT1301	MT1301	Espelette_P1	Mouse_Skin	Wild	109006	True Sample
MT1302	MT1302	MT1302	Espelette_P1	Mouse_Skin	Wild	110862	True Sample
MT1303	MT1303	MT1303	Espelette_P1	Mouse_Skin	Wild	57611	True Sample
MT1304	MT1304	MT1304	Espelette_P1	Mouse_Skin	Wild	101012	True Sample
MT1305	MT1305	MT1305	Espelette_P1	Mouse_Skin	Wild	166267	True Sample
MT1306	MT1306	MT1306	Espelette_P1	Mouse_Skin	Wild	162370	True Sample
MT1307	MT1307	MT1307	Espelette_P1	Mouse_Skin	Wild	177439	True Sample
MT1308	MT1308	MT1308	Espelette_P1	Mouse_Skin	Wild	83515	True Sample
MT1401	MT1401	MT1401	Espelette_P1	Mouse_Skin	Wild	161673	True Sample
MT1402	MT1402	MT1402	Espelette_P1	Mouse_Skin	Wild	16834	True Sample
MT1501	MT1501	MT1501	Espelette_P1	Mouse_Skin	Wild	131558	True Sample
MT1502	MT1502	MT1502	Espelette_P1	Mouse_Skin	Wild	14670	True Sample
MT1503	MT1503	MT1503	Espelette_P1	Mouse_Skin	Wild	9659	True Sample
MT1701	MT1701	MT1701	Espelette_P1	Mouse_Skin	Wild	117919	True Sample
MT1702	MT1702	MT1702	Espelette_P1	Mouse_Skin	Wild	124451	True Sample
MT1703	MT1703	MT1703	Espelette_P1	Mouse_Skin	Wild	54144	True Sample
MT1704	MT1704	MT1704	Espelette_P1	Mouse_Skin	Wild	92104	True Sample
MT1705	MT1705	MT1705	Espelette_P1	Mouse_Skin	Wild	50724	True Sample
MT1706	MT1706	MT1706	Espelette_P1	Mouse_Skin	Wild	5184	True Sample
MT1707	MT1707	MT1707	Espelette_P1	Mouse_Skin	Wild	15063	True Sample
MT2101	MT2101	MT2101	Espelette_P1	Mouse_Skin	Wild	128080	True Sample
MT2102	MT2102	MT2102	Espelette_P1	Mouse_Skin	Wild	84756	True Sample
MT2103	MT2103	MT2103	Espelette_P1	Mouse_Skin	Wild	33072	True Sample
MT2104	MT2104	MT2104	Espelette_P1	Mouse_Skin	Wild	96031	True Sample
MT2105	MT2105	MT2105	Espelette_P1	Mouse_Skin	Wild	12663	True Sample
MT2106	MT2106	MT2106	Espelette_P1	Mouse_Skin	Wild	9478	True Sample
MT2107	MT2107	MT2107	Espelette_P1	Mouse_Skin	Wild	86	True Sample
MT2108	MT2108	MT2108	Espelette_P1	Mouse_Skin	Wild	37230	True Sample
MT2109	MT2109	MT2109	Espelette_P1	Mouse_Skin	Wild	9697	True Sample
MT2110	MT2110	MT2110	Espelette_P1	Mouse_Skin	Wild	108194	True Sample
MT2111	MT2111	MT2111	Espelette_P1	Mouse_Skin	Wild	31401	True Sample
MT2112	MT2112	MT2112	Espelette_P1	Mouse_Skin	Wild	46448	True Sample

MT2113	MT2113	MT2113	Espelette_P1	Mouse_Skin	Wild	176517	True Sample
MT2114	MT2114	MT2114	Espelette_P1	Mouse_Skin	Wild	51011	True Sample
MT2115	MT2115	MT2115	Espelette_P1	Mouse_Skin	Wild	58274	True Sample
MT2116	MT2116	MT2116	Espelette_P1	Mouse_Skin	Wild	52460	True Sample
MT2117	MT2117	MT2117	Espelette_P1	Mouse_Skin	Wild	493007	True Sample
MT2118	MT2118	MT2118	Espelette_P1	Mouse_Skin	Wild	54596	True Sample
MT2119	MT2119	MT2119	Espelette_P1	Mouse_Skin	Wild	151607	True Sample
MT2120	MT2120	MT2120	Espelette_P1	Mouse_Skin	Wild	222958	True Sample
MT2121	MT2121	MT2121	Espelette_P1	Mouse_Skin	Wild	67960	True Sample
MT2601	MT2601	MT2601	Espelette_P1	Mouse_Skin	Wild	6143	True Sample
MT2603	MT2603	MT2603	Espelette_P1	Mouse_Skin	Wild	2565	True Sample
MT3501	MT3501	MT3501	Espelette_P1	Mouse_Skin	Wild	20678	True Sample
MT3502	MT3502	MT3502	Espelette_P1	Mouse_Skin	Wild	414471	True Sample
MT3503	MT3503	MT3503	Espelette_P1	Mouse_Skin	Wild	109323	True Sample
MT3504	MT3504	MT3504	Espelette_P1	Mouse_Skin	Wild	168112	True Sample
MT3505	MT3505	MT3505	Espelette_P1	Mouse_Skin	Wild	64932	True Sample
MT3506	MT3506	MT3506	Espelette_P1	Mouse_Skin	Wild	55688	True Sample
MT3507	MT3507	MT3507	Espelette_P1	Mouse_Skin	Wild	94345	True Sample
MT3508	MT3508	MT3508	Espelette_P1	Mouse_Skin	Wild	83162	True Sample
MT3509	MT3509	MT3509	Espelette_P1	Mouse_Skin	Wild	193942	True Sample
NC1	NC101	NC101	G15_P4_Phylosymbiosis_P2	Mouse_Skin	MPI-Lab	2727	Control Sample
NC2	NC_plate1	NC_plate1	Phylosymbiosis_P1	Mouse_Skin	MPI-Lab	9063	Control Sample
Negativecontrol11	Control 11	Control 11	G15_P3	Mouse_Skin	HL-Lab	6430	Control Sample
Negativecontrol12	Control 12	Control 12	G15_P3	Mouse_Skin	HL-Lab	8701	Control Sample
Negativecontrol13	Control 13	Control 13	G15_P4_Phylosymbiosis_P2	Mouse_Skin	HL-Lab	5577	Control Sample
NegativeControl14	Control 14	Control 14	G15_P4_Phylosymbiosis_P2	Mouse_Skin	HL-Lab	11155	Control Sample
NegativeControl2	Control 2	Control 2	G15_P1	Mouse_Skin	HL-Lab	50	Control Sample
NegativeControl3	Control 3	Control 3	G15_P1	Mouse_Skin	HL-Lab	638	Control Sample
NegativeControl4	Control 4	Control 4	G15_P1	Mouse_Skin	HL-Lab	8943	Control Sample
Negativecontrol5	Control 5	Control 5	G15_P2	Mouse_Skin	HL-Lab	5795	Control Sample
Negativecontrol6	Control 6	Control 6	G15_P2	Mouse_Skin	HL-Lab	6370	Control Sample
NegativeControl8	Control 8	Control 8	G15_P2	Mouse_Skin	HL-Lab	16825	Control Sample
NegativeControl9	Control 9	Control 9	G15_P3	Mouse_Skin	HL-Lab	8579	Control Sample
NegCon1ES	ES ctrl1	ES ctrl1	Espelette_P3_B6IL10	Mouse_Skin	Wild	50	Control Sample
NegCont1G15	Control 1	Control 1	G15_P1	Mouse_Skin	HL-Lab	1184	Control Sample
