# Supplementary material for: Assessing similarities and disparities in the skin microbiota between wild and laboratory populations of house mice
Source: ISME J. 2020 Jun 9;14(10):2367–80. doi: 10.1038/s41396-020-0690-7 (PMC7490391; doi:10.1038/s41396-020-0690-7)
Supplement: Supplementary file 10 — Supplementary Table 2 [file 41396_2020_690_MOESM10_ESM.pdf]

Supplementary Table 2.1 Summary statistics of the abundances of major phyla and genera, and unclassified Bacteria in wild (n=203), HL-Lab (n=225), MPI-Lab (n=29), and C57BL/6J (n=13) in standing and active communities. SD: standard deviation, Un: unclassified.

Taxon	Min	Max	Mean	SD	Group	
Firmicutes	0.011	0.903	0.399118226601	0.19805801808443	Wild	Phyla DNA
Actinobacteria	0.016	0.9345	0.2787684729064	0.17909947479658		
Proteobacteria	0.007	0.892	0.1851551724138	0.16843219780602		
Bacteroidetes	0	0.779	0.0880615763547	0.14942884627967		
Cyanobacteria	0	0.1445	0.0074876847291	0.01464729205058		
Unclassified_Bacteria	0	0.0585	0.0052586206897	0.00931188754084		
Firmicutes	0.001	0.8935	0.2254688888889	0.12434369279739	HL-Lab	
Actinobacteria	0	0.284	0.0645955555556	0.06640950124161		
Proteobacteria	0.0055	0.9815	0.1830422222222	0.1250396428011		
Bacteroidetes	0	0.871	0.2463222222222	0.19482940556247		
Cyanobacteria	0.004	0.99	0.12746	0.14259952101202		
Unclassified_Bacteria	0	0.1295	0.0246888888889	0.03475206971112		
Firmicutes	0.2315	0.455	0.340724137931	0.05813094611781	MPI-Lab	
Actinobacteria	0.0015	0.289	0.0569137931034	0.06955753899849		
Proteobacteria	0.014	0.309	0.1175862068966	0.06578225294831		
Bacteroidetes	0.2015	0.733	0.4376551724138	0.12498229184913		
Cyanobacteria	0	0.033	0.0079655172414	0.00917676708799		
Unclassified_Bacteria	0	0.0275	0.005275862069	0.00602018362157		
Firmicutes	0.1715	0.482	0.3166923076923	0.0879544243869	C57BL/6J	
Actinobacteria	0.1975	0.4245	0.304	0.07093805983626		
Proteobacteria	0.155	0.327	0.2391538461538	0.05446802755416		
Bacteroidetes	0.0225	0.1675	0.1001923076923	0.04397420572598		
Cyanobacteria	0.0025	0.0415	0.0154230769231	0.01115393457083		
Unclassified_Bacteria	0	0.0065	0.0006153846154	0.00181606082582		
Staphylococcus	0.0035	0.8955	0.3032167487685	0.20120012404596	Wild	Genera DNA
Unclassified_Muribaculaceae	0	0.565	0.0581009852217	0.10915975620741		
Acinetobacter	0	0.4035	0.0164285714286	0.04157893687329		
Streptococcus	0	0.336	0.0104556650246	0.02973123012055		
Corynebacterium_1	0	0.3915	0.0289162561576	0.06074135950266		
Alistipes	0	0.173	0.010645320197	0.0325148452808		
Unclassified_Bacteria	0	0.0585	0.0052586206897	0.00931188754084		
Staphylococcus	0	0.2815	0.0438133333333	0.05007102812149	HL-Lab	
Unclassified_Muribaculaceae	0	0.595	0.1674088888889	0.14889296674335		
Acinetobacter	0	0.4755	0.0318133333333	0.05318381869919		
Streptococcus	0	0.1825	0.02716	0.0318268361607		
Corynebacterium_1	0	0.2725	0.0299911111111	0.03922323258231		
Alistipes	0	0.2175	0.0245844444444	0.05613708490461		
Unclassified_Bacteria	0	0.1295	0.0246888888889	0.03475206971112		
Staphylococcus	0	0.194	0.0430862068966	0.05972213471054	MPI-Lab	
Unclassified_Muribaculaceae	0.1545	0.582	0.3157068965517	0.09649116514344		
Acinetobacter	0	0.138	0.021775862069	0.03119025323		
Streptococcus	0	0.083	0.0227586206897	0.02072809544765		
Corynebacterium_1	0	0.0415	0.0059827586207	0.00898757352307		
Alistipes	0.006	0.118	0.0550344827586	0.03098471138968		
Unclassified_Bacteria	0	0.0275	0.005275862069	0.00602018362157		
Staphylococcus	0.014	0.2215	0.1111923076923	0.07272853018289	C57BL/6J	
Unclassified_Muribaculaceae	5.00E-04	0.119	0.0524615384615	0.03755299673931		
Acinetobacter	0	0.065	0.0267307692308	0.02211957596849		
Streptococcus	0.013	0.0945	0.0437692307692	0.02688836131784		
Corynebacterium_1	0.0245	0.223	0.1059615384615	0.06326974709477		
Alistipes	0	0.0325	0.0054615384615	0.01066626601812		
Unclassified_Bacteria	0	0.0065	0.0006153846154	0.00181606082582		
Firmicutes	0.0015	0.884	0.4790689655172	0.21459883751269	Wild	Phyla RNA
Actinobacteria	0.0045	0.8965	0.2273694581281	0.18205634791199		
Proteobacteria	0.005	0.9235	0.1868226600985	0.14230988909237		
Bacteroidetes	0	0.416	0.0494334975369	0.05917679302527		
Cyanobacteria	0	0.744	0.0272315270936	0.09506183808726		
Unclassified_Bacteria	0	0.0395	0.0029458128079	0.00482074926435		
Firmicutes	0.052	0.9245	0.4212044444444	0.25346882376935	HL-Lab	
Actinobacteria	0	0.4895	0.06644	0.07139352524164		
Proteobacteria	0.002	0.8105	0.1702866666667	0.14262664735495		
Bacteroidetes	0.018	0.547	0.1678377777778	0.09965436538197		
Cyanobacteria	0	0.401	0.0386	0.0529425438228		
Unclassified_Bacteria	0	0.162	0.02644	0.03654409274131		
Firmicutes	0.1405	0.766	0.4019310344828	0.13897055984079	MPI-Lab	
Actinobacteria	0.0045	0.299	0.0717068965517	0.0772651415167		
Proteobacteria	0.051	0.6305	0.1968448275862	0.1374694189011		
Bacteroidetes	0.0055	0.3935	0.1642413793103	0.11118899777676		
Cyanobacteria	0	0.0735	0.0101551724138	0.01349014418558		
Unclassified_Bacteria	0	0.266	0.0274827586207	0.05461643570631		
Firmicutes	0.4925	0.9405	0.7793076923077	0.14718462590422	C57BL/6J	
Actinobacteria	0.0025	0.229	0.0649230769231	0.08166441650485		
Proteobacteria	0.007	0.165	0.0469230769231	0.0510456683413		
Bacteroidetes	0.0225	0.1985	0.0982692307692	0.05038088897944		
Cyanobacteria	0	0.011	0.0020769230769	0.00308792104987		
Unclassified_Bacteria	0	0.004	0.0008461538462	0.00112517804859		
Staphylococcus	0.001	0.794	0.2535	0.17092447379639	Wild	Genera RNA
Unclassified_Muribaculaceae	0	0.2035	0.0076995073892	0.01994009220099		
Acinetobacter	0	0.15	0.0128694581281	0.01728729153786		
Streptococcus	0	0.1095	0.0086600985222	0.01704929469202		
Corynebacterium_1	0	0.108	0.0091551724138	0.01600129358461		
Alistipes	0	0.0525	0.0030221674877	0.00664672563112		
Unclassified_Bacteria	0	0.0395	0.0029458128079	0.00482074926435		
Staphylococcus	0	0.1575	0.0253844444444	0.02721684545621	HL-Lab	
Unclassified_Muribaculaceae	0	0.418	0.1098155555556	0.09375658537717		
Acinetobacter	0	0.6025	0.0283355555556	0.0532986203066		
Streptococcus	0.003	0.542	0.0755022222222	0.10644860113003		
Corynebacterium_1	0	0.118	0.0169711111111	0.0215598277042		
Alistipes1	0	0.129	0.0090133333333	0.01471916571296		
Unclassified_Bacteria	0	0.162	0.02644	0.03654409274131		
Staphylococcus	0	0.0695	0.0156724137931	0.01519825874781	MPI-Lab	
Unclassified_Muribaculaceae	5.00E-04	0.225	0.0818103448276	0.06019409288073		
Acinetobacter	0	0.517	0.0391551724138	0.09747500626976		
Streptococcus	0.01	0.7175	0.1612413793103	0.15188697752239		
Corynebacterium_1	0	0.0205	0.0059310344828	0.00637478870509		
Alistipes2	0	0.1505	0.0402413793103	0.04029170880007		
Unclassified_Bacteria	0	0.266	0.0274827586207	0.05461643570631		
Staphylococcus	0.005	0.611	0.3164615384615	0.19176596647329	C57BL/6J	
Unclassified_Muribaculaceae	0.014	0.1295	0.0668846153846	0.03113288310222		
Acinetobacter	0.0025	0.0515	0.0168076923077	0.01506034868667		
Streptococcus	0	0.0065	0.0011923076923	0.0020870479704		
Corynebacterium_1	0.001	0.213	0.0425769230769	0.0754085666425		
Alistipes	0.003	0.038	0.0124615384615	0.01125178048589		
Unclassified_Bacteria	0	0.004	0.0008461538462	0.00112517804859		

Supplementary Table 2.2 Pairwise comparison of relative abundances of major taxa across mouse populations: wild (n=203), HL-Lab (n=225), MPI-Lab (n=29), and C57BL/6J (n=13). Un: unclassified

Taxon	Wild - HL-Lab	Wild - MPI-Lab	Wild - C57BL/6J	HL-Lab - MPI-Lab	HL-Lab - C57BL/6J	MPI-Lab - C57BL/6J	
Firmicutes	$< 10^{-16}$	0.30281647887259	0.250249611296463	0.0000000234557584196464	0.00215891560897185	0.414355685257487	
Actinobacteria	$< 10^{-16}$	0.000000000010762521327156	0.276471213731879	0.66156299250066	0.0000000674181461129524	0.0000145437038869651	
Proteobacteria	0.116052734665424	0.138375699228209	0.0145140932592215	0.010896369917824	0.0158669759788264	0.0000755344711531253	**Phyla DNA**
Bacteroidetes	$< 10^{-16}$	0.000000000000415131079566705	0.00257415155383737	0.0000000284841714740674	0.000591893267340583	0.0000000156747604577621	
Cyanobacteria	$< 10^{-16}$	0.309872922335601	0.000361585898538	9.04E-15	6.68E-06	0.016365418961245	
Staphylococcus	$< 10^{-16}$	3.60E-13	0.000487002499236	0.156911884376669	0.000523330806782	0.001914021625728	
Un_Muribaculaceae	$< 10^{-16}$	3.87E-14	0.05573688100829	1.15E-07	0.005553151633216	4.70E-07	
Acinetobacter	1.13E-09	0.082308647591945	0.028782145286178	0.252486989700294	0.503277585230722	0.252486989700294	**Genera DNA**
Streptococcus	$< 10^{-16}$	0.000123449125195	3.64E-07	0.792370108213054	0.007253969055356	0.019209971228989	
Corynebacterium_1	0.035646418266385	0.000568775129911	3.19E-06	3.80E-05	6.83E-06	1.96E-06	
Alistipes	$< 10^{-16}$	$< 10^{-16}$	0.649365552292401	1.14E-10	0.058286520103888	2.57E-06	
Firmicutes	0.008708443404237	0.033443365695521	1.39E-05	0.650898925432714	1.39E-05	1.15E-05	
Actinobacteria	1.63E-33	1.66E-08	5.67E-05	0.516675265945415	0.553522262065238	0.480957076420857	
Proteobacteria	0.255346784512324	0.674479185592843	9.64E-06	0.293867766431207	0.003232334743116	5.82E-05	**Phyla RNA**
Bacteroidetes	$< 10^{-16}$	2.32E-08	0.000329595429453	0.753361094902215	0.018826428733687	0.116366499673442	
Cyanobacteria	$< 10^{-16}$	0.05589782744901	0.021828210399684	0.001591805284024	4.32E-06	0.001591805284024	
Staphylococcus	$< 10^{-16}$	$< 10^{-16}$	0.175333229558827	0.220377850428414	2.61E-06	3.26E-05	
Un_Muribaculaceae	$< 10^{-16}$	3.67E-14	1.83E-08	0.411566730964925	0.533535861384047	0.633941920084828	
Acinetobacter	0.401423321260364	0.564225206680452	0.401423321260364	0.757045033993197	0.757045033993197	0.757045033993197	**Genera RNA**
Streptococcus	$< 10^{-16}$	$< 10^{-16}$	0.000790727046746	3.12E-05	3.90E-09	4.50E-07	
Corynebacterium_1	0.033214962817445	1	0.045727906075835	0.081139315702397	0.265165155843554	0.081139315702397	
Alistipes	5.08E-10	2.23E-11	3.37E-06	3.71E-07	0.027463574405101	0.027463574405101	

Correction for multiple testing was performed according to Benjamini and Hochberg (1995) [49]

Supplementary Table 2.3 Summary statistics of the abundances of the 6 most abundant genera within each population's standing and active communities: wild (n=203), HL-Lab (n=225), MPI-Lab (n=29), C57BL/6J (n=13). SD: standard deviation

	Min	Max	Mean	SD	Group
Staphylococcus	0.0035	0.8955	0.303216748768	0.20120012404596	
Un_Muribaculaceae	0	0.565	0.058100985222	0.10915975620741	
Streptomyces	0	0.399	0.049938423645	0.0666047332891	
Un_Actinobacteria	0	0.4285	0.033662561576	0.0524818985988	**Wild DNA**
Saccharopolyspora	0	0.523	0.030086206897	0.07595615727912	
Burkholderia.Caballeronia.Paraburkholderia	0	0.274	0.030041871921	0.04112660927597	
Other	0.073	0.9475	0.4949532	0.2072163	
Un_Muribaculaceae	0	0.595	0.167408888889	0.14889296674335	
Un_Chloroplast	0.0035	0.99	0.126822222222	0.14291786147633	
Ureaplasma	0	0.416	0.096446666667	0.14141536401174	
Staphylococcus	0	0.2815	0.043813333333	0.05007102812149	**HL-Lab DNA**
Acinetobacter	0	0.4755	0.031813333333	0.05318381869919	
Halomonas	0	0.1935	0.03002	0.04020661814748	
Other	0.007	0.9795	0.5036756	0.1772223	
Un_Muribaculaceae	0.1545	0.582	0.315706896552	0.09649116514344	
Lachnospiraceae_NK4A136_group	0.0255	0.1625	0.088724137931	0.03455290617478	
Alistipes	0.006	0.118	0.055034482759	0.03098471138968	
Staphylococcus	0	0.194	0.043086206897	0.05972213471054	**MPI-Lab DNA**
Un_Lachnospiraceae	0.012	0.0845	0.041810344828	0.01829393624807	
Un_Clostridiales_vadinBB60_group	0.002	0.044	0.023637931034	0.01185093166299	
Other	0.177	0.7075	0.432	0.1245489	
Cutibacterium	0.0285	0.191	0.122	0.05854627799157	
Staphylococcus	0.014	0.2215	0.111192307692	0.07272853018289	
Corynebacterium_1	0.0245	0.223	0.105961538462	0.06326974709477	
Pelomonas	0	0.1555	0.059423076923	0.03956473922249	**C57BL/6J DNA**
Un_Muribaculaceae	5.00E-04	0.119	0.052461538462	0.03755299673931	
Streptococcus	0.013	0.0945	0.043769230769	0.02688836131784	
Other	0.3825	0.613	0.50519231	0.07310083	
Staphylococcus	0.001	0.794	0.2535	0.17092447379639	
Pseudomonas	0	0.6205	0.039068965517	0.07789857694155	
Saccharopolyspora	0	0.406	0.033554187192	0.06833022264299	
Streptomyces	0	0.4115	0.032165024631	0.05236234767308	**Wild RNA**
Un_Chloroplast	0	0.744	0.026315270936	0.09504995618928	
Romboutsia	0	0.143	0.022736453202	0.0305990641032	
Other	0.109	0.967	0.5926601	0.1851832	
Un_Muribaculaceae	0	0.418	0.109815555556	0.09375658537717	
Streptococcus	0.003	0.542	0.075502222222	0.10644860113003	
Un_Lachnospiraceae	0	0.2845	0.064351111111	0.06713308883899	
Ureaplasma	0	0.3635	0.057035555556	0.08907024002848	**HL-Lab RNA**
Campylobacter	0	0.4065	0.038984444444	0.06573532208367	
Un_Chloroplast	0	0.401	0.036077777778	0.05058181089338	
Other	0.183	0.9505	0.6182333	0.1626785	
Streptococcus	0.01	0.7175	0.16124137931	0.15188697752239	
Un_Muribaculaceae	5.00E-04	0.225	0.081810344828	0.06019409288073	
Campylobacter	0	0.5175	0.081172413793	0.14408580503236	
Gemella	0	0.3	0.040775862069	0.07653624097293	**MPI-Lab RNA**
Lachnospiraceae_NK4A136_group	5.00E-04	0.1095	0.040362068966	0.03139548891382	
Alistipes	0	0.1505	0.04024137931	0.04029170880007	
Other	0.2405	0.764	0.5543966	0.1513301	
Staphylococcus	0.005	0.611	0.316461538462	0.19176596647329	
Aerococcus	0	0.555	0.206192307692	0.17777848323845	
Un_Lachnospiraceae	0.0185	0.235	0.078769230769	0.06379087427701	
Un_Muribaculaceae	0.014	0.1295	0.066884615385	0.03113288310222	**C57BL/6J RNA**
Corynebacterium_1	0.001	0.213	0.042576923077	0.0754085666425	
Lachnospiraceae_NK4A136_group	0.0065	0.0895	0.036461538462	0.02226119712495	
Other	0.0725	0.654	0.252653846154	0.20252256917598	

Supplementary Table 2.4 Summary statistics of the abundances of the core DNA (n=131) and RNA (n=191) genera, within each population wild (n=203), HL-Lab (n=225), MPI-Lab (n=29), C57BL/6J (n=13). SD:Standard deviation

Core genera in Wild DNA	Min	Max	Mean	SD
Acinetobacter	0	0.4035	0.016429	0.041579
Alistipes	0	0.173	0.010645	0.032515
Bacillus	0	0.1775	0.002808	0.013128
Bacteroides	0	0.0515	0.002761	0.006909
Brachybacterium	0	0.144	0.009367	0.014576
Bradyrhizobium	0	0.028	0.001303	0.003119
Brevibacterium	0	0.1905	0.024951	0.033416
Brevundimonas	0	0.016	0.000894	0.002109
Burkholderia.Caballeronia.Paraburkholderia	0	0.274	0.030042	0.041127
Clostridium_sensu_stricto_1	0	0.119	0.00668	0.018161
Corynebacterium_1	0	0.3915	0.028916	0.060741
Cupriavidus	0	0.035	0.001808	0.004163
Curtobacterium	0	0.024	0.000788	0.002238
Cutibacterium	0	0.05	0.004313	0.006947
Desulfovibrio	0	0.023	0.001111	0.002954
Dietzia	0	0.262	0.007534	0.024345
Erwinia	0	0.1435	0.00267	0.011279
Flavobacterium	0	0.0465	0.001401	0.005015
Glycomyces	0	0.0225	0.001539	0.003258
Gordonia	0	0.0885	0.006367	0.012575
Haematobacter	0	0.083	0.001397	0.00646
Halomonas	0	0.149	0.01686	0.02931
Janibacter	0	0.034	0.00283	0.005621
Jeotgalicoccus	0	0.045	0.001212	0.003755
Kocuria	0	0.6545	0.006488	0.047446
Lachnospiraceae_NK4A136_group	0	0.0645	0.003377	0.009114
Lactobacillus	0	0.198	0.012138	0.028454
Leuconostoc	0	0.527	0.019754	0.066876
Massilia	0	0.0565	0.00249	0.008158
Methylobacterium	0	0.0105	0.001025	0.002196
Microbacterium	0	0.021	0.001071	0.002435
Muribaculum	0	0.0165	0.002007	0.003339
Mycobacterium	0	0.045	0.002557	0.005907
Nesterenkonia	0	0.059	0.001059	0.004469
Nocardioides	0	0.0115	0.000946	0.00189
Nocardiopsis	0	0.152	0.009374	0.018147
Paeniclostridium	0	0.019	0.001116	0.003013
Pantoea	0	0.0705	0.003027	0.008566
Paracoccus	0	0.136	0.001717	0.010079
Parasutterella	0	0.049	0.00302	0.005467
Pseudomonas	0	0.54	0.01398	0.048877
Pseudonocardia	0	0.0505	0.0035	0.005945
Rahnella	0	0.129	0.002562	0.010512
Rhodococcus	0	0.045	0.002291	0.005336
Romboutsia	0	0.043	0.004268	0.006018
Saccharopolyspora	0	0.523	0.030086	0.075956
Salinicoccus	0	0.0215	0.001259	0.003042
Shewanella	0	0.0605	0.005217	0.009822
Sphingomonas	0	0.0775	0.003887	0.009228
Stackebrandtia	0	0.0175	0.000941	0.002311
Staphylococcus	0.0035	0.8955	0.303217	0.2012
Streptococcus	0	0.336	0.010456	0.029731
Streptomyces	0	0.399	0.049938	0.066605
Turicibacter	0	0.094	0.005431	0.012386
Unclassified_Actinobacteria	0	0.4285	0.033663	0.052482
Unclassified_Bacteria	0	0.0585	0.005259	0.009312
Unclassified_Betaproteobacteriales	0	0.5425	0.026337	0.074174
Unclassified_Burkholderiaceae	0	0.0355	0.001094	0.003154
Unclassified_Chloroplast	0	0.143	0.0073	0.014589
Unclassified_Enterobacteriaceae	0	0.8615	0.011254	0.066432
Unclassified_Halomonadaceae	0	0.064	0.004054	0.008105
Unclassified_Lachnospiraceae	0	0.019	0.001288	0.002477
Unclassified_Micrococcales	0	0.052	0.001569	0.004757
Unclassified_Muribaculaceae	0	0.565	0.058101	0.10916
Unclassified_Nocardioidaceae	0	0.0165	0.001059	0.002604
Unclassified_Pseudonocardiaceae	0	0.13	0.016224	0.025289
Unclassified_Rhizobiaceae	0	0.024	0.001692	0.003577
Unclassified_Ruminococcaceae	0	0.017	0.001071	0.002324
Unclassified_Sphingomonadaceae	0	0.044	0.001352	0.004297
Ureaplasma	0	0.1595	0.014039	0.023024
A2	0	0.0055	6.16E-05	0.000453
Aerococcus	0	0.141	0.001611	0.010214
Campylobacter	0	0.06	0.000633	0.004649
Candidatus_Saccharimonas	0	0.0045	0.000227	0.00069
Corynebacterium	0	0.0185	0.000399	0.00178
Deinococcus	0	0.021	0.000966	0.002506
Enhydrobacter	0	0.021	0.000456	0.00188
Facklamia	0	0.016	0.000751	0.001972
Faecalibaculum	0	0.013	0.000663	0.001711
Gemella	0	0.01	0.000256	0.001081
Helicobacter	0	0.7055	0.014495	0.083953
Lachnoclostridium	0	0.015	0.000335	0.001609
Lachnospiraceae_UCG.001	0	0.0025	4.43E-05	0.000299
Micrococcus	0	0.0115	0.000416	0.001355
Muribacter	0	0.0085	0.000111	0.00075
Odoribacter	0	0.012	0.000527	0.001706
Rikenellaceae_RC9_gut_group	0	0.013	0.000562	0.001608

Core genera in HL-Lab DNA	Min	Max	Mean	SD
Acinetobacter	0	0.4755	0.03181333	0.0531838
Alistipes	0	0.2175	0.02458444	0.0561371
Bacillus	0	0.0145	0.00075333	0.0021749
Bacteroides	0	0.118	0.01365333	0.0165738
Brachybacterium	0	0.01	0.00019111	0.0010613
Bradyrhizobium	0	0.044	0.00210667	0.0057663
Brevibacterium	0	0.0225	0.00065111	0.0023139
Brevundimonas	0	0.0365	0.00176444	0.0044564
Burkholderia.Caballeronia.Paraburkholderia	0	0.048	0.00398889	0.0071055
Clostridium_sensu_stricto_1	0	0.0115	0.00021111	0.0011024
Corynebacterium_1	0	0.2725	0.02999111	0.0392232
Cupriavidus	0	0.0125	0.00033556	0.0013437
Curtobacterium	0	0.005	4.22E-05	0.0004475
Cutibacterium	0	0.097	0.01271778	0.0211772
Desulfovibrio	0	0.04	0.00490444	0.0090322
Dietzia	0	5.00E-04	2.22E-06	3.33E-05
Erwinia	0	0.0125	0.00029333	0.00148
Flavobacterium	0	0.0155	0.00033556	0.0017151
Glycomyces	0	0	0	0
Gordonia	0	0	0	0
Haematobacter	0	0.088	0.00436	0.0097787
Halomonas	0	0.1935	0.03002	0.0402066
Janibacter	0	0.028	0.00016	0.0019153
Jeotgalicoccus	0	0.32	0.00791333	0.0285659
Kocuria	0	0.0305	0.00079111	0.0034051
Lachnospiraceae_NK4A136_group	0	0.099	0.01595333	0.0203233
Lactobacillus	0	0.0845	0.01148889	0.0112163
Leuconostoc	0	0.0265	0.00090667	0.003534
Massilia	0	0.01	0.00034667	0.0013943
Methylobacterium	0	0.018	0.00063778	0.0024442
Microbacterium	0	0.029	0.00053111	0.0028542
Muribaculum	0	0.0415	0.00718	0.00853
Mycobacterium	0	0.014	0.00013333	0.0010846
Nesterenkonia	0	0	0	0
Nocardioides	0	0.071	0.00122	0.0058012
Nocardiopsis	0	0.006	6.00E-05	0.0004907
Paeniclostridium	0	0.0025	1.11E-05	0.0001667
Pantoea	0	0.2395	0.00132	0.0159906
Paracoccus	0	0.0445	0.00284444	0.0060253
Parasutterella	0	0.0715	0.01230444	0.0168829
Pseudomonas	0	0.0855	0.00936222	0.0132596
Pseudonocardia	0	0.012	0.00027111	0.0012712
Rahnella	0	0.002	8.89E-06	0.0001333
Rhodococcus	0	0.009	0.00054	0.0015778
Romboutsia	0	0.0485	0.00642444	0.0090797
Saccharopolyspora	0	0.016	0.00035556	0.0019296
Salinicoccus	0	0.008	0.00011556	0.0007749
Shewanella	0	0.096	0.01337333	0.0202932
Sphingomonas	0	0.028	0.00188889	0.0040088
Stackebrandtia	0	0	0	0
Staphylococcus	0	0.2815	0.04381333	0.050071
Streptococcus	0	0.1825	0.02716	0.0318268
Streptomyces	0	0.0135	0.00022667	0.0013454
Turicibacter	0	0.0795	0.00450222	0.009373
Unclassified_Actinobacteria	0	0.028	0.00059111	0.0029481
Unclassified_Bacteria	0	0.1295	0.02468889	0.0347521
Unclassified_Betaproteobacteriales	0	0.976	0.01656444	0.0823482
Unclassified_Burkholderiaceae	0	0.024	0.00086889	0.0025716
Unclassified_Chloroplast	0.0035	0.99	0.12682222	0.1429179
Unclassified_Enterobacteriaceae	0	0.0635	0.00174222	0.0060312
Unclassified_Halomonadaceae	0	0.0645	0.00684667	0.0112401
Unclassified_Lachnospiraceae	0	0.1095	0.01381556	0.0171155
Unclassified_Micrococcales	0	0.001	6.67E-06	7.44E-05
Unclassified_Muribaculaceae	0	0.595	0.16740889	0.148893
Unclassified_Nocardioidaceae	0	5.00E-04	2.22E-06	3.33E-05
Unclassified_Pseudonocardiaceae	0	0.0125	0.00022	0.0012569
Unclassified_Rhizobiaceae	0	0.0255	0.00250667	0.0043593
Unclassified_Ruminococcaceae	0	0.0295	0.00399556	0.004563
Unclassified_Sphingomonadaceae	0	0.0745	0.00161778	0.0059618
Ureaplasma	0	0.416	0.09644667	0.1414154
A2	0	0.0625	0.00155556	0.0048677
Aerococcus	0	0.4625	0.01850889	0.0504233
Campylobacter	0	0.2295	0.01622667	0.0323297
Candidatus_Saccharimonas	0	0.0135	0.00138444	0.0025129
Corynebacterium	0	0.0215	0.00154	0.0036637
Deinococcus	0	0.2105	0.00548889	0.0170134
Enhydrobacter	0	0.045	0.00270667	0.0056147
Facklamia	0	0.191	0.00532667	0.0188131
Faecalibaculum	0	0.03	0.00221778	0.0044412
Gemella	0	0.1085	0.00622667	0.0144855
Helicobacter	0	0.031	0.00148222	0.0040377
Lachnoclostridium	0	0.0385	0.00176667	0.0043551
Lachnospiraceae_UCG.001	0	0.0275	0.00210222	0.0041639
Micrococcus	0	0.0445	0.00258889	0.0059235
Muribacter	0	0.047	0.00314222	0.0074371
Odoribacter	0	0.0405	0.00209778	0.0046387
Rikenellaceae_RC9_gut_group	0	0.0215	0.00164222	0.0034833

Core genera in MPI-Lab DNA	Min	Max	Mean	SD
Acinetobacter	0	0.138	0.021776	0.03119
Alistipes	0.006	0.118	0.055034	0.030985
Bacillus	0	0.0125	0.001534	0.003696
Bacteroides	0.001	0.064	0.021655	0.014605
Brachybacterium	0	0.0045	0.000276	0.00104
Bradyrhizobium	0	0	0	0
Brevibacterium	0	0.0095	0.001431	0.002534
Brevundimonas	0	0.014	0.001034	0.003125
Burkholderia.Caballeronia.Paraburkholderia	0	0.038	0.010103	0.011621
Clostridium_sensu_stricto_1	0	0	0	0
Corynebacterium_1	0	0.0415	0.005983	0.008988
Cupriavidus	0	0.012	0.000879	0.002502
Curtobacterium	0	0	0	0
Cutibacterium	0	0.101	0.016448	0.020857
Desulfovibrio	0	0.017	0.000793	0.003189
Dietzia	0	0	0	0
Erwinia	0	0	0	0
Flavobacterium	0	0.0075	0.00081	0.001961
Glycomyces	0	0	0	0
Gordonia	0	0	0	0
Haematobacter	0	0.06	0.004103	0.011541
Halomonas	0	0	0	0
Janibacter	0	0	0	0
Jeotgalicoccus	0	0.0105	0.000897	0.00248
Kocuria	0	0.23	0.016414	0.050968
Lachnospiraceae_NK4A136_group	0.0255	0.1625	0.088724	0.034553
Lactobacillus	0	0.048	0.012241	0.013427
Leuconostoc	0	5.00E-04	1.72E-05	9.28E-05
Massilia	0	0	0	0
Methylobacterium	0	0.0205	0.000983	0.003844
Microbacterium	0	0.007	0.000414	0.00157
Muribaculum	0	0.0345	0.015466	0.008976
Mycobacterium	0	0	0	0
Nesterenkonia	0	0	0	0
Nocardioides	0	0.001	3.45E-05	0.000186
Nocardiopsis	0	0	0	0
Paeniclostridium	0	0	0	0
Pantoea	0	0	0	0
Paracoccus	0	0.08	0.007	0.016528
Parasutterella	0	0.032	0.00919	0.008134
Pseudomonas	0	0.058	0.012069	0.012945
Pseudonocardia	0	0.0025	8.62E-05	0.000464
Rahnella	0	0	0	0
Rhodococcus	0	0	0	0
Romboutsia	0	0	0	0
Saccharopolyspora	0	0	0	0
Salinicoccus	0	0	0	0
Shewanella	0	0	0	0
Sphingomonas	0	0.0115	0.000672	0.002323
Stackebrandtia	0	0	0	0
Staphylococcus	0	0.194	0.043086	0.059722
Streptococcus	0	0.083	0.022759	0.020728
Streptomyces	0	0	0	0
Turicibacter	0	0.005	0.000466	0.001195
Unclassified_Actinobacteria	0	0	0	0
Unclassified_Bacteria	0	0.0275	0.005276	0.00602
Unclassified_Betaproteobacteriales	0	0.0015	5.17E-05	0.000279
Unclassified_Burkholderiaceae	0	0.0145	0.001828	0.004322
Unclassified_Chloroplast	0	0.033	0.005672	0.008051
Unclassified_Enterobacteriaceae	0	0.0145	0.002155	0.004241
Unclassified_Halomonadaceae	0	0	0	0
Unclassified_Lachnospiraceae	0.012	0.0845	0.04181	0.018294
Unclassified_Micrococcales	0	0	0	0
Unclassified_Muribaculaceae	0.1545	0.582	0.315707	0.096491
Unclassified_Nocardioidaceae	0	0.003	0.000103	0.000557
Unclassified_Pseudonocardiaceae	0	0	0	0
Unclassified_Rhizobiaceae	0	0	0	0
Unclassified_Ruminococcaceae	0	0.0265	0.007966	0.0077
Unclassified_Sphingomonadaceae	0	0.011	0.00069	0.002332
Ureaplasma	0	0.001	3.45E-05	0.000186
A2	0	0.0135	0.003948	0.003678
Aerococcus	0	0	0	0
Campylobacter	0	0.016	0.00269	0.004649
Candidatus_Saccharimonas	0	0.0075	0.000259	0.001393
Corynebacterium	0	0.017	0.002	0.004583
Deinococcus	0	0.005	0.000379	0.001107
Enhydrobacter	0	0.022	0.001931	0.004387
Facklamia	0	0.006	0.000207	0.001114
Faecalibaculum	0	0.0155	0.002379	0.003144
Gemella	0	0.012	0.001052	0.002836
Helicobacter	0	0.0145	0.001155	0.00321
Lachnoclostridium	0	0.0145	0.002672	0.003152
Lachnospiraceae_UCG.001	0	0.014	0.002914	0.003523
Micrococcus	0	0.0085	0.000897	0.002285
Muribacter	0	0.0105	0.000862	0.002478
Odoribacter	0	0.0105	0.002362	0.002542
Rikenellaceae_RC9_gut_group	0	0.0235	0.003448	0.004427

Core genera in C57BL/6J DNA	Min	Max	Mean	SD
Acinetobacter	0	0.065	0.026731	0.02212
Alistipes	0	0.0325	0.005462	0.010666
Bacillus	0	0.004	0.000308	0.001109
Bacteroides	0	0.0255	0.004731	0.009045
Brachybacterium	0	0	0	0
Bradyrhizobium	0	0	0	0
Brevibacterium	0	0	0	0
Brevundimonas	0	0.02	0.004154	0.006709
Burkholderia.Caballeronia.Paraburkholderia	0	0.045	0.006615	0.012986
Clostridium_sensu_stricto_1	0	0	0	0
Corynebacterium_1	0.0245	0.223	0.105962	0.06327
Cupriavidus	0	0	0	0
Curtobacterium	0	0.0085	0.000962	0.00252
Cutibacterium	0.0285	0.191	0.122	0.058546
Desulfovibrio	0	0	0	0
Dietzia	0	0.001	7.69E-05	0.000277
Erwinia	0	0	0	0
Flavobacterium	0	0.023	0.003231	0.007482
Glycomyces	0	0	0	0
Gordonia	0	0	0	0
Haematobacter	0	0.013	0.005115	0.004861
Halomonas	0	0	0	0
Janibacter	0	0	0	0
Jeotgalicoccus	0	0	0	0
Kocuria	0	0.003	0.000269	0.000832
Lachnospiraceae_NK4A136_group	0	0.041	0.0105	0.01171
Lactobacillus	0	0.021	0.003385	0.006384
Leuconostoc	0	0	0	0
Massilia	0	0.0625	0.015192	0.02122
Methylobacterium	0	0	0	0
Microbacterium	0	0.032	0.005846	0.010199
Muribaculum	0	0.0075	0.000923	0.002149
Mycobacterium	0	0.0125	0.000962	0.003467
Nesterenkonia	0	0	0	0
Nocardioides	0	0.0385	0.009808	0.011867
Nocardiopsis	0	0	0	0
Paeniclostridium	0	0	0	0
Pantoea	0	0	0	0
Paracoccus	0	0.0135	0.005538	0.00519
Parasutterella	0	0.009	0.000808	0.002496
Pseudomonas	0	0.0265	0.007154	0.008844
Pseudonocardia	0	0	0	0
Rahnella	0	0	0	0
Rhodococcus	0	0.003	0.000231	0.000832
Romboutsia	0	0	0	0
Saccharopolyspora	0	0	0	0
Salinicoccus	0	0.0185	0.001423	0.005131
Shewanella	0	0	0	0
Sphingomonas	0	0.0135	0.001308	0.003789
Stackebrandtia	0	0	0	0
Staphylococcus	0.014	0.2215	0.111192	0.072729
Streptococcus	0.013	0.0945	0.043769	0.026888
Streptomyces	0	0	0	0
Turicibacter	0	0.0325	0.004692	0.011483
Unclassified_Actinobacteria	0	0.018	0.001385	0.004992
Unclassified_Bacteria	0	0.0065	0.000615	0.001816
Unclassified_Betaproteobacteriales	0	0	0	0
Unclassified_Burkholderiaceae	0	0.019	0.002962	0.00661
Unclassified_Chloroplast	0	0.029	0.012462	0.009284
Unclassified_Enterobacteriaceae	0	0	0	0
Unclassified_Halomonadaceae	0	0	0	0
Unclassified_Lachnospiraceae	0	0.035	0.010885	0.012068
Unclassified_Micrococcales	0	0.037	0.006385	0.01113
Unclassified_Muribaculaceae	5.00E-04	0.119	0.052462	0.037553
Unclassified_Nocardioidaceae	0	0.0135	0.0015	0.003969
Unclassified_Pseudonocardiaceae	0	0	0	0
Unclassified_Rhizobiaceae	0	0.022	0.002846	0.006401
Unclassified_Ruminococcaceae	0	0.017	0.0025	0.005323
Unclassified_Sphingomonadaceae	0	0.01	0.001923	0.003047
Ureaplasma	0	0	0	0
A2	0	0.025	0.002692	0.007195
Aerococcus	0	0.173	0.032615	0.059793
Campylobacter	0	0	0	0
Candidatus_Saccharimonas	0	0	0	0
Corynebacterium	0	0.0225	0.002	0.006215
Deinococcus	0	0	0	0
Enhydrobacter	0	0.02	0.004808	0.005577
Facklamia	0	0.0065	5.00E-04	0.001803
Faecalibaculum	0	0.0625	0.010346	0.018066
Gemella	0	0.0085	0.000654	0.002357
Helicobacter	0	0	0	0
Lachnoclostridium	0	0.008	0.000615	0.002219
Lachnospiraceae_UCG.001	0	0.0185	0.001538	0.005113
Micrococcus	0	0.039	0.006308	0.012554
Muribacter	0	0	0	0
Odoribacter	0	0.0085	0.000654	0.002357
Rikenellaceae_RC9_gut_group	0	0	0	0

Roseburia	0	0.0025	3.20E-05	0.000228
Rothia	0	0.012	0.00034	0.001498
Ruminiclostridium_9	0	0.003	0.000101	0.000417
Ruminococcaceae_UCG.014	0	0.028	0.00153	0.004776
Unclassified_Clostridiales	0	0.0095	0.000325	0.001109
Unclassified_Clostridiales_vadinBB60_group	0	0.0055	0.000145	0.000633
Unclassified_Corynebacteriales	0	0.054	0.001059	0.004829
Unclassified_Desulfovibrionaceae	0	0.0045	0.000133	0.000588
Unclassified_Prevotellaceae	0	0.006	0.000483	0.001214
Unclassified_Rhodobacteraceae	0	0.182	0.002145	0.014965
Unclassified_Weeksellaceae	0	0.019	0.000288	0.001643
Alcanivorax	0	0	0	0
Amaricoccus	0	0.0305	0.000261	0.002242
Anaeroplasma	0	0.0045	2.22E-05	0.000316
Anaerotruncus	0	0	0	0
Lachnospiraceae_UCG.006	0	0.003	0.000103	0.000436
Mucispirillum	0	0.006	0.000374	0.000979
Nosocomiicoccus	0	0.047	0.000268	0.003306
Oscillibacter	0	0.006	8.87E-05	0.000514
Parabacteroides	0	0.006	0.000259	0.000902
Porphyromonas	0	0.0075	0.000236	0.00102
Prevotellaceae_NK3B31_group	0	0.0065	0.00036	0.001036
Prevotellaceae_UCG.001	0	0.0065	0.000433	0.001159
Ralstonia	0	0.034	0.001049	0.003208
Rodentibacter	0	0.003	6.65E-05	0.000365
Ruminiclostridium	0	0.004	8.37E-05	0.000402
Ruminiclostridium_6	0	0.012	0.000401	0.001436
Ruminococcus_1	0	0.0055	5.67E-05	0.000429
Unclassified_Bacteroidales	0	0.016	0.000209	0.001234
Unclassified_Gastranaerophilales	0	0.0025	0.000121	0.000455
Unclassified_Rhodospirillales	0	0.013	8.62E-05	0.000934
Veillonella	0	0.0115	0.000217	0.00104
Actinomyces	0	0.0095	0.000197	0.000912
Alloprevotella	0	0.034	5.00E-04	0.002598
Anaerococcus	0	0.008	0.00051	0.001549
Chryseobacterium	0	0.0325	0.00047	0.002492
Dolosigranulum	0	0.008	8.62E-05	0.000667
Fusobacterium	0	0.0045	6.65E-05	0.000455
Ignavigranum	0	0.0125	0.000207	0.001106
Kytococcus	0	0.001	4.93E-06	7.02E-05
Pelomonas	0	0.0075	0.000177	0.000815
Peptoniphilus	0	0.0015	7.39E-06	0.000105
Skermanella	0	0.0135	8.87E-05	0.000987
Tepidimonas	0	0.0025	3.69E-05	0.000248
Unclassified_Rhodanobacteraceae	0	0.009	0.000118	0.000901
Unclassified_Xanthobacteraceae	0	0.0125	0.000692	0.001827

Roseburia	0	0.015	0.00144222	0.0030777
Rothia	0	0.0495	0.00175778	0.0049077
Ruminiclostridium_9	0	0.0155	0.00093333	0.0023121
Ruminococcaceae_UCG.014	0	0.0495	0.00444889	0.0103362
Unclassified_Clostridiales	0	0.0195	0.00156444	0.0031163
Unclassified_Clostridiales_vadinBB60_group	0	0.02	0.00111556	0.0026784
Unclassified_Corynebacteriales	0	0.0785	0.00289556	0.0076499
Unclassified_Desulfovibrionaceae	0	0.0215	0.00083556	0.0021577
Unclassified_Prevotellaceae	0	0.053	0.00149556	0.0045203
Unclassified_Rhodobacteraceae	0	0.114	0.00286889	0.0095511
Unclassified_Weeksellaceae	0	0.455	0.01148222	0.0402659
Alcanivorax	0	0	0	0
Amaricoccus	0	0.024	0.00062222	0.0025993
Anaeroplasma	0	0.0045	6.00E-05	0.0004353
Anaerotruncus	0	0.009	6.44E-05	0.0006332
Lachnospiraceae_UCG.006	0	0.012	0.00057556	0.0016713
Mucispirillum	0	0.088	0.00126889	0.0064127
Nosocomiicoccus	0	0.0195	0.00091556	0.0026074
Oscillibacter	0	0.023	0.00097778	0.0027746
Parabacteroides	0	0.029	0.00161333	0.0042225
Porphyromonas	0	0.203	0.00137556	0.0138115
Prevotellaceae_NK3B31_group	0	0.0085	0.00067111	0.0016362
Prevotellaceae_UCG.001	0	0.0265	0.00122	0.0030365
Ralstonia	0	0.0155	0.00048889	0.0017787
Rodentibacter	0	0.0535	0.00173111	0.0061463
Ruminiclostridium	0	0.0315	0.00121333	0.003497
Ruminiclostridium_6	0	0.0185	0.00099556	0.0026542
Ruminococcus_1	0	0.017	0.00025333	0.001612
Unclassified_Bacteroidales	0	0.0455	0.00122222	0.0049486
Unclassified_Gastranaerophilales	0	0.01	0.00043556	0.0014757
Unclassified_Rhodospirillales	0	0.0135	0.00034444	0.0016957
Veillonella	0	0.0135	0.00062889	0.0022428
Actinomyces	0	0.025	0.00052889	0.00226
Alloprevotella	0	0.19	0.00244667	0.0136205
Anaerococcus	0	0.035	0.00131111	0.0038604
Chryseobacterium	0	0.021	0.00053333	0.0023295
Dolosigranulum	0	0.0165	0.00037333	0.0018746
Fusobacterium	0	0.004	7.33E-05	0.0005002
Ignavigranum	0	0.0975	0.00683111	0.0156723
Kytococcus	0	0.0065	2.89E-05	0.0004333
Pelomonas	0	0.007	0.00018444	0.001019
Peptoniphilus	0	0.0335	0.00050444	0.0027029
Skermanella	0	0.009	0.00011111	0.0008176
Tepidimonas	0	0.01	8.44E-05	0.0007452
Unclassified_Rhodanobacteraceae	0	0.009	4.00E-05	6.00E-04
Unclassified_Xanthobacteraceae	0	0.0545	0.00130222	0.004841

Roseburia	0	0.0085	0.001052	0.001795
Rothia	0	0.01	0.001879	0.003101
Ruminiclostridium_9	0	0.0045	0.001224	0.001623
Ruminococcaceae_UCG.014	0	0.0125	0.001966	0.004004
Unclassified_Clostridiales	0	0.031	0.013397	0.008145
Unclassified_Clostridiales_vadinBB60_group	0.002	0.044	0.023638	0.011851
Unclassified_Corynebacteriales	0	0	0	0
Unclassified_Desulfovibrionaceae	0	0	0	0
Unclassified_Prevotellaceae	0	0.0025	8.62E-05	0.000464
Unclassified_Rhodobacteraceae	0	0.0105	0.000466	0.002009
Unclassified_Weeksellaceae	0	0.0085	0.000466	0.001679
Alcanivorax	0	0.02	0.0045	0.005485
Amaricoccus	0	0.015	0.001414	0.003213
Anaeroplasma	0	0.01	0.001328	0.002759
Anaerotruncus	0	0.0095	0.002172	0.002726
Lachnospiraceae_UCG.006	0	0.008	0.001	0.001973
Mucispirillum	0	0.0465	0.018983	0.01231
Nosocomiicoccus	0	0.0095	0.002	0.002739
Oscillibacter	0	0.021	0.006431	0.006636
Parabacteroides	0	0.011	0.002121	0.002908
Porphyromonas	0	0.009	0.001172	0.002237
Prevotellaceae_NK3B31_group	0	0.0085	0.001276	0.002068
Prevotellaceae_UCG.001	0	0.028	0.01019	0.007886
Ralstonia	0	0.0225	0.007138	0.007146
Rodentibacter	0	0.019	0.00231	0.004403
Ruminiclostridium	0	0.017	0.00681	0.004254
Ruminiclostridium_6	0	0.0215	0.006983	0.005567
Ruminococcus_1	0	0.035	0.014328	0.008682
Unclassified_Bacteroidales	0	0.0195	0.00169	0.003992
Unclassified_Gastranaerophilales	0	0.0115	0.002293	0.003646
Unclassified_Rhodospirillales	0	0.0125	0.001845	0.00305
Veillonella	0	0.013	0.001914	0.003735
Actinomyces	0	0.0085	0.000845	0.002147
Alloprevotella	0	0.0015	5.17E-05	0.000279
Anaerococcus	0	0.0035	0.000293	0.000774
Chryseobacterium	0	0	0	0
Dolosigranulum	0	0.011	0.000948	0.00248
Fusobacterium	0	0.0045	0.000155	0.000836
Ignavigranum	0	0.003	0.000155	0.000614
Kytococcus	0	0.0015	5.17E-05	0.000279
Pelomonas	0	0.01	0.000345	0.001857
Peptoniphilus	0	0	0	0
Skermanella	0	0	0	0
Tepidimonas	0	0.0075	0.000517	0.001868
Unclassified_Rhodanobacteraceae	0	0	0	0
Unclassified_Xanthobacteraceae	0	0	0	0

Roseburia	0	0.015	0.002769	0.005566
Rothia	0	0.019	0.002269	0.005395
Ruminiclostridium_9	0	0.007	0.000577	0.001935
Ruminococcaceae_UCG.014	0	0	0	0
Unclassified_Clostridiales	0	0.008	0.001577	0.00244
Unclassified_Clostridiales_vadinBB60_group	0	0	0	0
Unclassified_Corynebacteriales	0	0	0	0
Unclassified_Desulfovibrionaceae	0	0	0	0
Unclassified_Prevotellaceae	0	0	0	0
Unclassified_Rhodobacteraceae	0	0.0255	0.003962	0.00735
Unclassified_Weeksellaceae	0	0	0	0
Alcanivorax	0	0	0	0
Amaricoccus	0	0.0185	0.005231	0.006515
Anaeroplasma	0	0	0	0
Anaerotruncus	0	0.002	0.000154	0.000555
Lachnospiraceae_UCG.006	0	0.012	0.001115	0.003343
Mucispirillum	0	0.014	0.001654	0.004249
Nosocomiicoccus	0	0.0225	0.001731	0.00624
Oscillibacter	0	0.0025	0.000192	0.000693
Parabacteroides	0	0	0	0
Porphyromonas	0	0.022	0.003192	0.007809
Prevotellaceae_NK3B31_group	0	0.015	0.003231	0.005085
Prevotellaceae_UCG.001	0	0.023	0.005115	0.007911
Ralstonia	0	0	0	0
Rodentibacter	0	0	0	0
Ruminiclostridium	0	0.0105	0.002269	0.003539
Ruminiclostridium_6	0	0	0	0
Ruminococcus_1	0	0	0	0
Unclassified_Bacteroidales	0	0.015	0.001154	0.00416
Unclassified_Gastranaerophilales	0	0	0	0
Unclassified_Rhodospirillales	0	0	0	0
Veillonella	0	0.0355	0.005192	0.010692
Actinomyces	0	0.0225	0.005615	0.007866
Alloprevotella	0	0.02	0.003962	0.007546
Anaerococcus	0	0.09	0.015192	0.023929
Chryseobacterium	0	0.019	0.006923	0.008446
Dolosigranulum	0	0.027	0.008231	0.008698
Fusobacterium	0	0.0305	0.008269	0.011629
Ignavigranum	0	0.0425	0.006308	0.012051
Kytococcus	0	0.022	0.004077	0.007271
Pelomonas	0	0.1555	0.059423	0.039565
Peptoniphilus	0	0.0505	0.006731	0.014502
Skermanella	0	0.045	0.012269	0.012498
Tepidimonas	0	0.029	0.004115	0.008486
Unclassified_Rhodanobacteraceae	0	0.043	0.005923	0.012351
Unclassified_Xanthobacteraceae	0	0.0155	0.003423	0.005751

**Core genera in Wild RNA**	**Min**	**Max**	**Mean**	**SD**
Acinetobacter	0	0.15	0.012869	0.017287
Actinomycetospora	0	0.23	0.003185	0.017604
Aerococcus	0	0.0945	0.002608	0.008515
Aeromicrobium	0	0.0205	0.001167	0.002259
Alistipes	0	0.0525	0.003022	0.006647
Anaerococcus	0	0.0185	0.001177	0.002638
Anaerostipes	0	0.0445	0.003419	0.007715
Aureimonas	0	0.007	0.000732	0.001426
Bacillus	0	0.239	0.005761	0.017822
Bacteroides	0	0.416	0.017431	0.046592
Blautia	0	0.0395	0.00251	0.005736
Brachybacterium	0	0.083	0.005522	0.008834
Brevibacterium	0	0.06	0.006384	0.009058
Brevundimonas	0	0.054	0.00099	0.004081
Campylobacter	0	0.2225	0.007793	0.027336
Christensenellaceae_R.7_group	0	0.0425	0.003456	0.006549
Chryseobacterium	0	0.015	0.000589	0.001633
Clostridium_sensu_stricto_1	0	0.217	0.018796	0.041262
Collinsella	0	0.0125	0.000988	0.002127
Comamonas	0	0.079	0.00166	0.00787
Coprococcus_1	0	0.014	0.000628	0.001671
Corynebacterium_1	0	0.108	0.009155	0.016001
Curtobacterium	0	0.0305	0.001207	0.003013
Cutibacterium	0	0.065	0.003645	0.009169
Deinococcus	0	0.0095	0.000672	0.001637
Delftia	0	0.031	0.002246	0.003765
Dialister	0	0.057	0.005571	0.010701
Dietzia	0	0.2455	0.011475	0.026873
Dorea	0	0.025	0.001517	0.003564
Enterococcus	0	0.2785	0.002155	0.019758
Erwinia	0	0.2555	0.007399	0.02182
Erysipelotrichaceae_UCG.003	0	0.024	0.002234	0.004447
Facklamia	0	0.0235	0.000894	0.002314
Faecalibacterium	0	0.0255	0.001884	0.003866
Flavobacterium	0	0.053	0.003495	0.007152
Frigoribacterium	0	0.024	0.001264	0.002974
Frondihabitans	0	0.0085	0.000719	0.001651
Gordonia	0	0.167	0.003392	0.013467
Halomonas	0	0.0195	0.00198	0.003229
Helicobacter	0	0.6725	0.010685	0.061048
Hymenobacter	0	0.019	0.00066	0.001788
Ignavigranum	0	0.0115	0.000591	0.001382

**Core genera in HL-Lab RNA**	**Min**	**Max**	**Mean**	**SD**
Acinetobacter	0	0.6025	0.02833556	0.0532986
Actinomycetospora	0	0.0085	9.78E-05	0.0007773
Aerococcus	0	0.378	0.02642889	0.0659205
Aeromicrobium	0	0.0165	0.00017556	0.0014436
Alistipes	0	0.129	0.00901333	0.0147192
Anaerococcus	0	0.0365	0.00150222	0.0043344
Anaerostipes	0	0.0195	0.00065333	0.0024426
Aureimonas	0	0.012	5.78E-05	0.0008025
Bacillus	0	0.024	0.00043333	0.0019866
Bacteroides	0	0.1145	0.01386444	0.0177235
Blautia	0	0.009	0.00022889	0.0010825
Brachybacterium	0	0.009	0.00016889	0.0009719
Brevibacterium	0	0.0435	0.00106889	0.0039135
Brevundimonas	0	0.012	0.00065111	0.0018454
Campylobacter	0	0.4065	0.03898444	0.0657353
Christensenellaceae_R.7_group	0	0.018	0.00030667	0.0016815
Chryseobacterium	0	0.007	0.00016	0.0008705
Clostridium_sensu_stricto_1	0	0.0165	0.00052889	0.002205
Collinsella	0	0.0125	0.00015111	0.0011931
Comamonas	0	0.0215	0.00034667	0.0016942
Coprococcus_1	0	0	0	0
Corynebacterium_1	0	0.118	0.01697111	0.0215598
Curtobacterium	0	5.00E-04	2.22E-06	3.33E-05
Cutibacterium	0	0.13	0.01868	0.0303025
Deinococcus	0	0.0695	0.00299333	0.0071529
Delftia	0	0.003	2.67E-05	0.0002617
Dialister	0	0.018	0.00063778	0.0021179
Dietzia	0	0	0	0
Dorea	0	0.0155	9.56E-05	0.0010642
Enterococcus	0	0.03	0.00365333	0.0062296
Erwinia	0	0.0115	8.00E-05	0.0007921
Erysipelotrichaceae_UCG.003	0	0.014	6.44E-05	0.0009338
Facklamia	0	0.1075	0.00434222	0.0136785
Faecalibacterium	0	0.0185	0.00053778	0.0022751
Flavobacterium	0	0.0745	0.00291111	0.0077657
Frigoribacterium	0	0.002	8.89E-06	0.0001333
Frondihabitans	0	0.0015	6.67E-06	1.00E-04
Gordonia	0	0.009	9.56E-05	0.0007711
Halomonas	0	0.1155	0.00996	0.0176332
Helicobacter	0	0.0205	0.00143556	0.0025274
Hymenobacter	0	0.019	0.00046444	0.0019436
Ignavigranum	0	0.04	0.00322667	0.0073223

**Core genera in MPI-Lab RNA**	**Min**	**Max**	**Mean**	**SD**
Acinetobacter	0	0.517	0.039155	0.097475
Actinomycetospora	0	0	0	0
Aerococcus	0	0	0	0
Aeromicrobium	0	0	0	0
Alistipes	0	0.1505	0.040241	0.040292
Anaerococcus	0	0.013	0.001534	0.002777
Anaerostipes	0	0	0	0
Aureimonas	0	0	0	0
Bacillus	0	0.0125	0.002552	0.003301
Bacteroides	0	0.056	0.013931	0.01279
Blautia	0	0.006	0.000483	0.001448
Brachybacterium	0	0	0	0
Brevibacterium	0	0.008	0.001552	0.00249
Brevundimonas	0	0.0505	0.004707	0.011845
Campylobacter	0	0.5175	0.081172	0.144086
Christensenellaceae_R.7_group	0	0.004	0.000276	0.000882
Chryseobacterium	0	0.006	0.00031	0.001145
Clostridium_sensu_stricto_1	0	0.002	0.000121	0.000415
Collinsella	0	0	0	0
Comamonas	0	0.037	0.001759	0.007015
Coprococcus_1	0	0	0	0
Corynebacterium_1	0	0.0205	0.005931	0.006375
Curtobacterium	0	0.017	0.000586	0.003157
Cutibacterium	0	0.237	0.019897	0.043026
Deinococcus	0	0.0105	0.000707	0.00252
Delftia	0	0	0	0
Dialister	0	0.0165	0.000569	0.003064
Dietzia	0	0	0	0
Dorea	0	0.0035	0.000293	0.000892
Enterococcus	0	0.07	0.004983	0.013465
Erwinia	0	0	0	0
Erysipelotrichaceae_UCG.003	0	0	0	0
Facklamia	0	0.005	0.000259	0.001023
Faecalibacterium	0	0.005	0.000293	0.001013
Flavobacterium	0	0.0095	5.00E-04	0.001964
Frigoribacterium	0	5.00E-04	1.72E-05	9.28E-05
Frondihabitans	0	0	0	0
Gordonia	0	0	0	0
Halomonas	0	0.0205	0.001879	0.005104
Helicobacter	0	0.1475	0.006914	0.027183
Hymenobacter	0	0.0105	0.000552	0.002041
Ignavigranum	0	0	0	0

**Core genera in C57BL/6J RNA**	**Min**	**Max**	**Mean**	**SD**
Acinetobacter	0.0025	0.0515	0.016808	0.01506
Actinomycetospora	0	0	0	0
Aerococcus	0	0.555	0.206192	0.177778
Aeromicrobium	0	5.00E-04	3.85E-05	0.000139
Alistipes	0.003	0.038	0.012462	0.011252
Anaerococcus	0	5.00E-04	3.85E-05	0.000139
Anaerostipes	0	0	0	0
Aureimonas	0	5.00E-04	7.69E-05	0.000188
Bacillus	0	0	0	0
Bacteroides	0	0.0015	0.000577	0.000534
Blautia	0	0	0	0
Brachybacterium	0	0.001	0.000115	0.0003
Brevibacterium	0	0	0	0
Brevundimonas	0	0.0065	0.0015	0.001756
Campylobacter	0	0	0	0
Christensenellaceae_R.7_group	0	0	0	0
Chryseobacterium	0	0.0035	0.000346	0.000966
Clostridium_sensu_stricto_1	0	5.00E-04	3.85E-05	0.000139
Collinsella	0	0	0	0
Comamonas	0	0	0	0
Coprococcus_1	0	0	0	0
Corynebacterium_1	0.001	0.213	0.042577	0.075409
Curtobacterium	0	0	0	0
Cutibacterium	0	0.018	0.004231	0.004914
Deinococcus	0	0.001	7.69E-05	0.000277
Delftia	0	0	0	0
Dialister	0	0	0	0
Dietzia	0	0	0	0
Dorea	0	0	0	0
Enterococcus	0	0.005	0.001577	0.001946
Erwinia	0	0	0	0
Erysipelotrichaceae_UCG.003	0	0	0	0
Facklamia	0	0.0015	0.000115	0.000416
Faecalibacterium	0	0	0	0
Flavobacterium	0	0.008	0.001	0.002208
Frigoribacterium	0	5.00E-04	3.85E-05	0.000139
Frondihabitans	0	0	0	0
Gordonia	0	5.00E-04	3.85E-05	0.000139
Halomonas	0	0	0	0
Helicobacter	0	0	0	0
Hymenobacter	0	0.002	0.000231	0.000599
Ignavigranum	0	0.0035	0.000385	0.000961

Janibacter	0	0.023	0.001424	0.003557
Jeotgalicoccus	0	0.032	0.001882	0.003874
Kluyvera	0	0.0455	0.00249	0.006277
Kocuria	0	0.179	0.001436	0.01263
Lachnoclostridium	0	0.063	0.001495	0.006177
Lachnospiraceae_NK4A136_group	0	0.0685	0.003158	0.0072
Lactobacillus	0	0.304	0.015978	0.033915
Lactococcus	0	0.0285	0.000788	0.002453
Leuconostoc	0	0.21	0.008978	0.023967
Lysinibacillus	0	0.0055	0.000586	0.001021
Marmoricola	0	0.0165	0.001426	0.002452
Massilia	0	0.0325	0.002441	0.005329
Methylobacterium	0	0.038	0.003606	0.006836
Microbacterium	0	0.0245	0.001537	0.002815
Muribacter	0	0.0355	0.001579	0.003937
Mycobacterium	0	0.0315	0.002424	0.004551
Nocardioides	0	0.023	0.002557	0.003877
Nocardiopsis	0	0.188	0.012074	0.025807
Novosphingobium	0	0.013	0.000837	0.001883
Paenibacillus	0	0.013	0.001204	0.002062
Paeniclostridium	0	0.0455	0.004936	0.007563
Pantoea	0	0.1115	0.004387	0.010852
Parabacteroides	0	0.0215	0.001067	0.002783
Paracoccus	0	0.0095	0.000667	0.001331
Pedobacter	0	0.0205	0.000909	0.002296
Prevotella_9	0	0.05	0.003685	0.006851
Promicromonospora	0	0.0215	0.000754	0.002359
Pseudogracilibacillus	0	0.085	0.00214	0.008807
Pseudomonas	0	0.6205	0.039069	0.077899
Pseudonocardia	0	0.3825	0.01569	0.053028
Rathayibacter	0	0.0185	0.000603	0.001848
Rhodococcus	0	0.035	0.003406	0.006004
Romboutsia	0	0.143	0.022736	0.030599
Roseburia	0	0.0095	0.001086	0.001944
Ruminiclostridium	0	0.0135	0.00064	0.001926
Ruminococcaceae_UCG.005	0	0.022	0.001103	0.002543
Ruminococcaceae_UCG.010	0	0.009	0.000475	0.001179
Ruminococcaceae_UCG.014	0	0.032	0.00232	0.004236
Saccharopolyspora	0	0.406	0.033554	0.06833
Salinicoccus	0	0.04	0.001369	0.004302
Shewanella	0	0.012	0.000759	0.001668
Sphingobacterium	0	0.0135	0.000468	0.001296
Sphingobium	0	0.0115	0.000522	0.001446
Sphingomonas	0	0.171	0.012768	0.023375
Sporosarcina	0	0.255	0.003722	0.018814
Stackebrandtia	0	0.0115	0.000475	0.001324
Staphylococcus	0.001	0.794	0.2535	0.170924
Stenotrophomonas	0	0.019	0.00064	0.001889
Streptococcus	0	0.1095	0.00866	0.017049
Streptomyces	0	0.4115	0.032165	0.052362
Subdoligranulum	0	0.0465	0.00433	0.008052
Terrisporobacter	0	0.163	0.010515	0.027402
Turicibacter	0	0.135	0.011539	0.022445
Unclassified_Actinobacteria	0	0.4265	0.021766	0.051036
Unclassified_Alphaproteobacteria	0	0.029	0.000628	0.002376
Unclassified_Bacillaceae	0	0.0835	0.004187	0.010071
Unclassified_Bacillales	0	0.0255	0.001103	0.002921
Unclassified_Bacteria	0	0.0395	0.002946	0.004821
Unclassified_Bacteroidales	0	0.059	0.001793	0.005873
Unclassified_Beijerinckiaceae	0	0.0925	0.008884	0.01667
Unclassified_Betaproteobacteriales	0	0.0625	0.001727	0.005761
Unclassified_Burkholderiaceae	0	0.023	0.001448	0.002926
Unclassified_Carnobacteriaceae	0	0.222	0.002244	0.015816
Unclassified_Chitinophagaceae	0	0.0085	0.000709	0.001438
Unclassified_Chloroplast	0	0.744	0.026315	0.09505
Unclassified_Clostridiales	0	0.027	0.002362	0.004871
Unclassified_Corynebacteriales	0	0.0745	0.001158	0.006514
Unclassified_Devosiaceae	0	0.011	0.000805	0.001799
Unclassified_Enterobacteriaceae	0	0.8485	0.016067	0.062968
Unclassified_Flavobacteriaceae	0	0.0455	0.000961	0.003786
Unclassified_Gammaproteobacteria	0	0.0125	0.000756	0.001749
Unclassified_Halomonadaceae	0	0.0105	0.000463	0.001249
Unclassified_Lachnospiraceae	0	0.1405	0.019355	0.031334
Unclassified_Microbacteriaceae	0	0.01	0.00083	0.001708
Unclassified_Micrococcales	0	0.0055	0.000534	0.00112
Unclassified_Muribaculaceae	0	0.2035	0.0077	0.01994
Unclassified_Nocardiaceae	0	0.045	0.001788	0.005812
Unclassified_Nocardioidaceae	0	0.017	0.001638	0.002802
Unclassified_Peptostreptococcaceae	0	0.01	0.000933	0.00161
Unclassified_Planococcaceae	0	0.0245	0.001143	0.002802
Unclassified_Propionibacteriaceae	0	0.0065	0.000485	0.000966
Unclassified_Pseudonocardiaceae	0	0.14	0.017897	0.028376
Unclassified_Rhizobiaceae	0	0.0585	0.005015	0.008273
Unclassified_Rhizobiales	0	0.011	0.000796	0.001568
Unclassified_Rhodobacteraceae	0	0.028	0.002111	0.003657
Unclassified_Ruminococcaceae	0	0.049	0.006096	0.010295
Unclassified_Sphingomonadaceae	0	0.0885	0.009855	0.014032
Unclassified_Xanthobacteraceae	0	0.2565	0.018059	0.027297
Variovorax	0	0.008	0.000717	0.001506
Williamsia	0	0.0235	0.0022	0.004341
A2	0	0.004	8.13E-05	0.000391

Janibacter	0	0.0165	8.22E-05	0.0011024
Jeotgalicoccus	0	0.098	0.00586	0.0147504
Kluyvera	0	0	0	0
Kocuria	0	0.012	0.00036667	0.00152
Lachnoclostridium	0	0.0925	0.00916	0.0128645
Lachnospiraceae_NK4A136_group	0	0.173	0.02910889	0.0285401
Lactobacillus	0	0.1385	0.01515556	0.0155832
Lactococcus	0	0.011	0.00020444	0.0010734
Leuconostoc	0	0.0695	0.00541556	0.0119993
Lysinibacillus	0	0.0135	0.00010667	0.0009784
Marmoricola	0	0.0325	0.00029111	0.0023128
Massilia	0	0.074	0.00145111	0.008209
Methylobacterium	0	0.0095	0.00047556	0.0014324
Microbacterium	0	0.0215	0.00066222	0.0027385
Muribacter	0	0.061	0.00249111	0.0069017
Mycobacterium	0	0.004	0.00011556	0.0005489
Nocardioides	0	0.054	0.00084	0.004083
Nocardiopsis	0	0.0045	4.67E-05	0.0003853
Novosphingobium	0	0.005	5.33E-05	0.0004812
Paenibacillus	0	0.0215	1.00E-04	0.0014346
Paeniclostridium	0	0	0	0
Pantoea	0	0.008	0.00012	0.0007433
Parabacteroides	0	0.0145	0.00076889	0.0023054
Paracoccus	0	0.0365	0.00334	0.0065369
Pedobacter	0	0.0585	0.00071111	0.0042854
Prevotella_9	0	0.051	0.0041	0.0094317
Promicromonospora	0	0	0	0
Pseudogracilibacillus	0	0.0035	4.22E-05	0.000365
Pseudomonas	0	0.1175	0.01243111	0.0162071
Pseudonocardia	0	0.0025	3.11E-05	0.0002342
Rathayibacter	0	0.002	1.33E-05	0.0001488
Rhodococcus	0	0.0505	0.00210222	0.0052869
Romboutsia	0	0.047	0.00704444	0.0101022
Roseburia	0	0.2395	0.01477556	0.0301705
Ruminiclostridium	0	0.0555	0.00659333	0.0102304
Ruminococcaceae_UCG.005	0	0.0105	0.00014667	0.0008403
Ruminococcaceae_UCG.010	0	0.002	1.33E-05	0.0001411
Ruminococcaceae_UCG.014	0	0.021	0.00098889	0.0027167
Saccharopolyspora	0	0.0035	8.00E-05	0.0004255
Salinicoccus	0	0	0	0
Shewanella	0	0.05	0.00591111	0.0108698
Sphingobacterium	0	0.0025	1.11E-05	0.0001667
Sphingobium	0	0.0355	0.00088889	0.0035846
Sphingomonas	0	0.018	0.00101111	0.0023925
Sporosarcina	0	0.1645	0.00551333	0.0208125
Stackebrandtia	0	0	0	0
Staphylococcus	0	0.1575	0.02538444	0.0272168
Stenotrophomonas	0	0.0135	0.00012667	0.0011861
Streptococcus	0.003	0.542	0.07550222	0.1064486
Streptomyces	0	0.0265	0.00015333	0.0018095
Subdoligranulum	0	0.0245	0.00078889	0.0032388
Terrisporobacter	0	0	0	0
Turicibacter	0	0.1245	0.00436222	0.0136302
Unclassified_Actinobacteria	0	0.0225	0.00045333	0.0022122
Unclassified_Alphaproteobacteria	0	0.0055	0.00014889	0.000593
Unclassified_Bacillaceae	0	0.004	7.11E-05	0.00045
Unclassified_Bacillales	0	0.0655	0.00117111	0.0063092
Unclassified_Bacteria	0	0.162	0.02644	0.0365441
Unclassified_Bacteroidales	0	0.0655	0.00176667	0.0072809
Unclassified_Beijerinckiaceae	0	0.017	0.00023778	0.0013153
Unclassified_Betaproteobacteriales	0	0.804	0.00520667	0.0537524
Unclassified_Burkholderiaceae	0	0.0265	0.00093111	0.0032546
Unclassified_Carnobacteriaceae	0	0.0125	0.00010889	0.0009308
Unclassified_Chitinophagaceae	0	0.027	0.00022889	0.0020354
Unclassified_Chloroplast	0	0.401	0.03607778	0.0505818
Unclassified_Clostridiales	0	0.026	0.00371556	0.0051382
Unclassified_Corynebacteriales	0	0.0225	0.00287111	0.0041205
Unclassified_Devosiaceae	0	0	0	0
Unclassified_Enterobacteriaceae	0	0.0165	0.00052222	0.0021577
Unclassified_Flavobacteriaceae	0	0.011	0.00011778	0.0008625
Unclassified_Gammaproteobacteria	0	0.0215	0.00089111	0.003188
Unclassified_Halomonadaceae	0	0.0435	0.00218667	0.0051214
Unclassified_Lachnospiraceae	0	0.2845	0.06435111	0.0671331
Unclassified_Microbacteriaceae	0	0.044	0.00059778	0.0035168
Unclassified_Micrococcales	0	0	0	0
Unclassified_Muribaculaceae	0	0.418	0.10981556	0.0937566
Unclassified_Nocardiaceae	0	0	0	0
Unclassified_Nocardioidaceae	0	0.0065	2.89E-05	0.0004333
Unclassified_Peptostreptococcaceae	0	0.022	0.00019778	0.0015733
Unclassified_Planococcaceae	0	0.168	0.00428444	0.0220271
Unclassified_Propionibacteriaceae	0	0.008	0.00013778	0.0008666
Unclassified_Pseudonocardiaceae	0	0.007	4.44E-05	0.0004821
Unclassified_Rhizobiaceae	0	0.023	0.00135111	0.002945
Unclassified_Rhizobiales	0	0.0175	0.00073556	0.0023126
Unclassified_Rhodobacteraceae	0	0.0255	0.00197111	0.0047277
Unclassified_Ruminococcaceae	0	0.0495	0.01012444	0.0082271
Unclassified_Sphingomonadaceae	0	0.056	0.00303333	0.0069259
Unclassified_Xanthobacteraceae	0	0.4855	0.02740222	0.0775675
Variovorax	0	0.0265	0.00026889	0.0020089
Williamsia	0	0.0015	8.89E-06	0.0001053
A2	0	0.0875	0.00908222	0.0163091

Janibacter	0	0.003	0.000103	0.000557
Jeotgalicoccus	0	0.143	0.007638	0.026755
Kluyvera	0	0	0	0
Kocuria	0	0.006	0.000448	0.001372
Lachnoclostridium	0	0.014	0.001966	0.003642
Lachnospiraceae_NK4A136_group	5.00E-04	0.1095	0.040362	0.031395
Lactobacillus	0	0.046	0.010362	0.012211
Lactococcus	0	0.0045	0.000207	0.000871
Leuconostoc	0	0	0	0
Lysinibacillus	0	0.003	0.000121	0.000561
Marmoricola	0	0.0045	0.000259	0.000912
Massilia	0	5.00E-04	1.72E-05	9.28E-05
Methylobacterium	0	0.0025	0.000224	0.000635
Microbacterium	0	0.0025	8.62E-05	0.000464
Muribacter	0	0.0465	0.009	0.013641
Mycobacterium	0	0	0	0
Nocardioides	0	0.0105	0.000948	0.002772
Nocardiopsis	0	0	0	0
Novosphingobium	0	0.003	0.000103	0.000557
Paenibacillus	0	0.003	0.000241	0.000786
Paeniclostridium	0	0	0	0
Pantoea	0	0.0045	0.00031	0.000967
Parabacteroides	0	0.0145	0.001897	0.003797
Paracoccus	0	0.0555	0.00681	0.013462
Pedobacter	0	0	0	0
Prevotella_9	0	0.0065	5.00E-04	0.001427
Promicromonospora	0	0	0	0
Pseudogracilibacillus	0	0	0	0
Pseudomonas	0	0.053	0.012862	0.013267
Pseudonocardia	0	0	0	0
Rathayibacter	0	5.00E-04	1.72E-05	9.28E-05
Rhodococcus	0	0.001	3.45E-05	0.000186
Romboutsia	0	0.0025	0.000138	0.000533
Roseburia	0	0.0105	0.001914	0.003
Ruminiclostridium	0	0.0295	0.006534	0.007944
Ruminococcaceae_UCG.005	0	0.0055	0.00031	0.001113
Ruminococcaceae_UCG.010	0	0.0035	0.000121	0.00065
Ruminococcaceae_UCG.014	0	0.015	0.00219	0.003899
Saccharopolyspora	0	0	0	0
Salinicoccus	0	0	0	0
Shewanella	0	0.0035	0.000241	0.000903
Sphingobacterium	0	0.0115	0.000397	0.002135
Sphingobium	0	0.0065	0.000276	0.001229
Sphingomonas	0	0.0355	0.004414	0.009165
Sporosarcina	0	0.006	0.000293	0.001192
Stackebrandtia	0	0	0	0
Staphylococcus	0	0.0695	0.015672	0.015198
Stenotrophomonas	0	0.0215	0.003379	0.005628
Streptococcus	0.01	0.7175	0.161241	0.151887
Streptomyces	0	5.00E-04	1.72E-05	9.28E-05
Subdoligranulum	0	0.0095	0.00081	0.00237
Terrisporobacter	0	0	0	0
Turicibacter	0	0.0055	0.000397	0.001256
Unclassified_Actinobacteria	0	0.047	0.002741	0.009154
Unclassified_Alphaproteobacteria	0	0.003	0.000224	0.000702
Unclassified_Bacillaceae	0	0.005	0.000345	0.001053
Unclassified_Bacillales	0	0	0	0
Unclassified_Bacteria	0	0.266	0.027483	0.054616
Unclassified_Bacteroidales	0	0.0275	0.003672	0.007392
Unclassified_Beijerinckiaceae	0	0.006	0.000207	0.001114
Unclassified_Betaproteobacteriales	0	0.057	0.002621	0.010724
Unclassified_Burkholderiaceae	0	0.027	0.002931	0.006974
Unclassified_Carnobacteriaceae	0	0.0675	0.00819	0.016639
Unclassified_Chitinophagaceae	0	0	0	0
Unclassified_Chloroplast	0	0.0735	0.008828	0.013605
Unclassified_Clostridiales	0	0.0775	0.011707	0.016573
Unclassified_Corynebacteriales	0	0.0135	0.001466	0.003006
Unclassified_Devosiaceae	0	0	0	0
Unclassified_Enterobacteriaceae	0	0.0145	0.001793	0.00315
Unclassified_Flavobacteriaceae	0	0	0	0
Unclassified_Gammaproteobacteria	0	0.06	0.012534	0.020471
Unclassified_Halomonadaceae	0	0.019	0.001	0.003715
Unclassified_Lachnospiraceae	5.00E-04	0.1145	0.033897	0.029937
Unclassified_Microbacteriaceae	0	0.0085	0.000552	0.002067
Unclassified_Micrococcales	0	0	0	0
Unclassified_Muribaculaceae	5.00E-04	0.225	0.08181	0.060194
Unclassified_Nocardiaceae	0	0	0	0
Unclassified_Nocardioidaceae	0	0	0	0
Unclassified_Peptostreptococcaceae	0	0	0	0
Unclassified_Planococcaceae	0	0	0	0
Unclassified_Propionibacteriaceae	0	0.002	0.000155	0.000484
Unclassified_Pseudonocardiaceae	0	0	0	0
Unclassified_Rhizobiaceae	0	0	0	0
Unclassified_Rhizobiales	0	0	0	0
Unclassified_Rhodobacteraceae	0	0.0035	0.000397	0.000948
Unclassified_Ruminococcaceae	0	0.036	0.004776	0.00769
Unclassified_Sphingomonadaceae	0	0.051	0.001948	0.009452
Unclassified_Xanthobacteraceae	0	0.0085	0.000586	0.001904
Variovorax	0	0.0065	0.000414	0.001553
Williamsia	0	0.0055	0.00019	0.001021
A2	0	0.0045	0.000569	0.001266

Janibacter	0	5.00E-04	3.85E-05	0.000139
Jeotgalicoccus	0	0.0025	0.000231	0.000696
Kluyvera	0	0	0	0
Kocuria	0	0.03	0.006846	0.008422
Lachnoclostridium	0	0.045	0.006923	0.011798
Lachnospiraceae_NK4A136_group	0.0065	0.0895	0.036462	0.022261
Lactobacillus	5.00E-04	0.0225	0.004923	0.006377
Lactococcus	0	0	0	0
Leuconostoc	0	0	0	0
Lysinibacillus	0	0	0	0
Marmoricola	0	0.003	0.000346	0.000899
Massilia	5.00E-04	0.0355	0.007462	0.010162
Methylobacterium	0	0.001	7.69E-05	0.000277
Microbacterium	0	0.0015	0.000269	0.000563
Muribacter	0	0	0	0
Mycobacterium	0	0	0	0
Nocardioides	0	0.0105	0.000962	0.002883
Nocardiopsis	0	0	0	0
Novosphingobium	0	0	0	0
Paenibacillus	0	0	0	0
Paeniclostridium	0	5.00E-04	3.85E-05	0.000139
Pantoea	0	0	0	0
Parabacteroides	0	0.004	0.000808	0.001109
Paracoccus	0	0.0045	5.00E-04	0.001242
Pedobacter	0	0	0	0
Prevotella_9	0	0	0	0
Promicromonospora	0	0	0	0
Pseudogracilibacillus	0	0	0	0
Pseudomonas	0	0.013	0.003077	0.004122
Pseudonocardia	0	0.0015	0.000154	0.000427
Rathayibacter	0	0	0	0
Rhodococcus	0	0	0	0
Romboutsia	0	0	0	0
Roseburia	0	0.016	0.004231	0.004438
Ruminiclostridium	0.0055	0.0865	0.024962	0.025524
Ruminococcaceae_UCG.005	0	0.0055	0.000462	0.00152
Ruminococcaceae_UCG.010	0	0	0	0
Ruminococcaceae_UCG.014	0	5.00E-04	0.000115	0.000219
Saccharopolyspora	0	0	0	0
Salinicoccus	0	0	0	0
Shewanella	0	0	0	0
Sphingobacterium	0	0	0	0
Sphingobium	0	0	0	0
Sphingomonas	0	5.00E-04	7.69E-05	0.000188
Sporosarcina	0	0	0	0
Stackebrandtia	0	0	0	0
Staphylococcus	0.005	0.611	0.316462	0.191766
Stenotrophomonas	0	0	0	0
Streptococcus	0	0.0065	0.001192	0.002087
Streptomyces	0	5.00E-04	3.85E-05	0.000139
Subdoligranulum	0	0	0	0
Terrisporobacter	0	0	0	0
Turicibacter	0	0.021	0.001615	0.005824
Unclassified_Actinobacteria	0	5.00E-04	7.69E-05	0.000188
Unclassified_Alphaproteobacteria	0	0	0	0
Unclassified_Bacillaceae	0	0	0	0
Unclassified_Bacillales	0	0	0	0
Unclassified_Bacteria	0	0.004	0.000846	0.001125
Unclassified_Bacteroidales	0	0	0	0
Unclassified_Beijerinckiaceae	0	5.00E-04	7.69E-05	0.000188
Unclassified_Betaproteobacteriales	0	0	0	0
Unclassified_Burkholderiaceae	0	0.001	0.000154	0.000315
Unclassified_Carnobacteriaceae	0	0	0	0
Unclassified_Chitinophagaceae	0	5.00E-04	3.85E-05	0.000139
Unclassified_Chloroplast	0	0.0015	0.000308	0.00048
Unclassified_Clostridiales	0.002	0.0145	0.006808	0.004226
Unclassified_Corynebacteriales	0	0	0	0
Unclassified_Devosiaceae	0	0	0	0
Unclassified_Enterobacteriaceae	0	5.00E-04	3.85E-05	0.000139
Unclassified_Flavobacteriaceae	0	0	0	0
Unclassified_Gammaproteobacteria	0	0	0	0
Unclassified_Halomonadaceae	0	0	0	0
Unclassified_Lachnospiraceae	0.0185	0.235	0.078769	0.063791
Unclassified_Microbacteriaceae	0	0.0015	0.000154	0.000427
Unclassified_Micrococcales	0	5.00E-04	3.85E-05	0.000139
Unclassified_Muribaculaceae	0.014	0.1295	0.066885	0.031133
Unclassified_Nocardiaceae	0	0	0	0
Unclassified_Nocardioidaceae	0	0	0	0
Unclassified_Peptostreptococcaceae	0	0	0	0
Unclassified_Planococcaceae	0	0	0	0
Unclassified_Propionibacteriaceae	0	0.0075	0.000615	0.002073
Unclassified_Pseudonocardiaceae	0	0	0	0
Unclassified_Rhizobiaceae	0	0.0105	0.002385	0.003292
Unclassified_Rhizobiales	0	0	0	0
Unclassified_Rhodobacteraceae	0	0.005	0.000692	0.00148
Unclassified_Ruminococcaceae	0.001	0.0265	0.009846	0.008754
Unclassified_Sphingomonadaceae	0	0.0165	0.002808	0.004558
Unclassified_Xanthobacteraceae	0	0.0035	0.000269	0.000971
Variovorax	0	0.001	7.69E-05	0.000277
Williamsia	0	5.00E-04	3.85E-05	0.000139
A2	0.001	0.181	0.020923	0.048669

Alloprevotella	0	0.018	0.000429	0.001683
ASF356	0	0.0435	0.000638	0.0035
Atopostipes	0	0.0135	0.000584	0.001748
Burkholderia.Caballeronia.Paraburkholderia	0	0.0205	0.00034	0.001673
Butyricicoccus	0	0.002	3.94E-05	0.000207
Candidatus_Saccharimonas	0	5.00E-04	7.39E-06	6.05E-05
Corynebacterium	0	0.0125	0.000187	0.001055
Desulfovibrio	0	0.0175	0.000298	0.001407
Enhydrobacter	0	0.0235	0.000493	0.001894
Faecalibaculum	0	0.0065	0.000106	0.000576
GCA.900066575	0	0.003	0.000118	0.000435
Gemella	0	0.0415	0.000325	0.002971
Haematobacter	0	0.072	0.000749	0.005303
Lachnospiraceae_UCG.001	0	0.0725	0.000613	0.00514
Lachnospiraceae_UCG.006	0	0.009	0.000374	0.001212
Leucobacter	0	0.0125	0.000374	0.001295
Micrococcus	0	0.0215	0.000554	0.001996
Mucispirillum	0	0.0055	0.000268	0.000812
Muribaculum	0	0.007	0.000182	0.000775
Odoribacter	0	0.0035	0.00016	0.000541
Oscillibacter	0	0.0075	0.000254	0.00091
Parasutterella	0	0.0125	0.000451	0.001393
Prevotellaceae_UCG.001	0	0.0025	4.43E-05	0.000234
Rikenella	0	0.001	7.39E-06	7.83E-05
Rodentibacter	0	0.001	2.71E-05	0.000159
Rothia	0	0.0175	0.000365	0.00153
Ruminiclostridium_5	0	0.009	0.000333	0.001025
Ruminiclostridium_9	0	0.0075	0.000409	0.001053
Unclassified_Clostridiales_vadinBB60_group	0	0.0055	0.000342	0.000936
Unclassified_Desulfovibrionaceae	0	0.0155	0.000355	0.001387
Unclassified_Eggerthellaceae	0	0.0035	5.67E-05	0.000338
Unclassified_Firmicutes	0	0.015	0.000672	0.002007
Unclassified_Prevotellaceae	0	0.006	0.000251	0.000702
Unclassified_Weeksellaceae	0	0.0085	0.000411	0.001037
Ureaplasma	0	0.025	0.000808	0.00333
Alcanivorax	0	0.0015	4.68E-05	0.000231
Anaerotruncus	0	0.002	4.19E-05	0.000221
Bergeyella	0	0.0035	0.000101	0.00046
Neisseria	0	0.003	2.22E-05	0.000235
Nosocomiicoccus	0	0.0045	0.000167	0.000652
Ralstonia	0	0.0035	0.000108	0.000417
Rikenellaceae_RC9_gut_group	0	0.005	0.000175	0.000605
Ruminiclostridium_6	0	0.012	0.000406	0.001269
Ruminococcus_1	0	0.0065	0.000254	0.000744
Unclassified_Gastranaerophilales	0	0.0035	4.93E-05	0.000307
Unclassified_Neisseriaceae	0	5.00E-04	4.93E-06	4.95E-05
Unclassified_Pasteurellaceae	0	0.001	1.72E-05	0.000115
Alkanindiges	0	0.0035	0.00015	0.000508
Bilophila	0	0.0025	3.69E-05	0.000263
Dolosigranulum	0	0.0085	0.000145	0.000906
Enterorhabdus	0	0.0025	6.40E-05	0.000324
Lachnospiraceae_FCS020_group	0	0.0025	4.43E-05	0.000264
Pelomonas	0	0.003	5.17E-05	0.000316
Prevotellaceae_NK3B31_group	0	0.0045	0.000145	0.000526
Ruminococcaceae_UCG.009	0	0.0015	2.22E-05	0.000143
Skermanella	0	0.004	4.68E-05	0.000321
Unclassified_Coriobacteriales	0	0.001	1.23E-05	0.000105
Unclassified_Peptococcaceae	0	0.0035	6.16E-05	0.000355

Alloprevotella	0	0.041	0.00237333	0.0051104
ASF356	0	0.0315	0.00148889	0.0032975
Atopostipes	0	0.2185	0.00646444	0.0241714
Burkholderia.Caballeronia.Paraburkholderia	0	0.0255	0.00202667	0.0041853
Butyricicoccus	0	0.019	0.00252444	0.0037489
Candidatus_Saccharimonas	0	0.011	0.00105333	0.0022617
Corynebacterium	0	0.026	0.00162222	0.0029771
Desulfovibrio	0	0.0625	0.00737111	0.0113589
Enhydrobacter	0	0.0275	0.00221333	0.0042256
Faecalibaculum	0	0.036	0.00237778	0.0053866
GCA.900066575	0	0.018	0.00096667	0.0023428
Gemella	0	0.2345	0.01430889	0.0330451
Haematobacter	0	0.0605	0.00373111	0.0076888
Lachnospiraceae_UCG.001	0	0.335	0.01654444	0.0336788
Lachnospiraceae_UCG.006	0	0.049	0.00354222	0.0071242
Leucobacter	0	0.081	0.00472444	0.0103567
Micrococcus	0	0.3715	0.00458889	0.0252996
Mucispirillum	0	0.17	0.00215778	0.0116447
Muribaculum	0	0.048	0.00671111	0.0084778
Odoribacter	0	0.0195	0.00098222	0.0025578
Oscillibacter	0	0.0445	0.00394	0.0069845
Parasutterella	0	0.0575	0.00967333	0.0144873
Prevotellaceae_UCG.001	0	0.015	0.00076	0.001653
Rikenella	0	0.0075	0.00069333	0.0014735
Rodentibacter	0	0.023	0.00205111	0.004189
Rothia	0	0.069	0.00280889	0.0067481
Ruminiclostridium_5	0	0.0245	0.00137778	0.0031756
Ruminiclostridium_9	0	0.032	0.00357333	0.0055037
Unclassified_Clostridiales_vadinBB60_group	0	0.0075	0.00082444	0.0015423
Unclassified_Desulfovibrionaceae	0	0.011	0.00124889	0.002067
Unclassified_Eggerthellaceae	0	0.011	0.00106	0.0022028
Unclassified_Firmicutes	0	0.0085	0.00083333	0.0016717
Unclassified_Prevotellaceae	0	0.037	0.00228889	0.0042726
Unclassified_Weeksellaceae	0	0.0525	0.00241556	0.0060348
Ureaplasma	0	0.3635	0.05703556	0.0890702
Alcanivorax	0	0	0	0
Anaerotruncus	0	0.0065	0.00024222	0.000844
Bergeyella	0	0.0085	4.67E-05	0.0005816
Neisseria	0	0.0015	6.67E-06	1.00E-04
Nosocomiicoccus	0	0.0935	0.00197333	0.0076824
Ralstonia	0	0.02	0.00050667	0.0019969
Rikenellaceae_RC9_gut_group	0	0.0045	0.00030889	0.0008551
Ruminiclostridium_6	0	0.014	0.00054	0.0015799
Ruminococcus_1	0	0.025	0.00141778	0.0040116
Unclassified_Gastranaerophilales	0	0.0045	4.67E-05	0.0003552
Unclassified_Neisseriaceae	0	0.0055	6.67E-05	0.0004795
Unclassified_Pasteurellaceae	0	0	0	0
Alkanindiges	0	0	0	0
Bilophila	0	0.0085	0.00012889	0.0007508
Dolosigranulum	0	0.015	0.00035778	0.0016318
Enterorhabdus	0	0.007	0.00014889	0.0008352
Lachnospiraceae_FCS020_group	0	0.0095	0.00018	0.0008114
Pelomonas	0	0.0145	0.00025111	0.0014448
Prevotellaceae_NK3B31_group	0	0.014	0.00056444	0.0016594
Ruminococcaceae_UCG.009	0	0.004	6.89E-05	0.0003609
Skermanella	0	0.006	6.89E-05	0.0005793
Unclassified_Coriobacteriales	0	0.0085	0.00012889	0.0007418
Unclassified_Peptococcaceae	0	0.003	0.00025556	0.0006252

Alloprevotella	0	0.0035	0.000121	0.00065
ASF356	0	0.016	0.001828	0.00346
Atopostipes	0	0.0455	0.002483	0.00873
Burkholderia.Caballeronia.Paraburkholderia	0	0.049	0.012259	0.015357
Butyricicoccus	0	0.0025	0.000138	0.000533
Candidatus_Saccharimonas	0	0.0035	0.000121	0.00065
Corynebacterium	0	0.24	0.01819	0.053703
Desulfovibrio	0	0.004	0.000397	0.001072
Enhydrobacter	0	0.02	0.002086	0.004099
Faecalibaculum	0	0.002	8.62E-05	0.00038
GCA.900066575	0	0.0035	0.000207	0.000785
Gemella	0	0.3	0.040776	0.076536
Haematobacter	0	0.0315	0.003483	0.006858
Lachnospiraceae_UCG.001	0	0.0105	0.00169	0.002476
Lachnospiraceae_UCG.006	0	0.0125	0.000897	0.002451
Leucobacter	0	0.0025	8.62E-05	0.000464
Micrococcus	0	0.0125	0.001259	0.002624
Mucispirillum	0	0.069	0.016534	0.022786
Muribaculum	0	0.0195	0.002621	0.004613
Odoribacter	0	0.01	0.00231	0.003004
Oscillibacter	0	0.013	0.00219	0.003121
Parasutterella	0	0.0125	0.003	0.003608
Prevotellaceae_UCG.001	0	0.013	0.003121	0.003565
Rikenella	0	0	0	0
Rodentibacter	0	0.087	0.01219	0.023119
Rothia	0	0.07	0.002638	0.012974
Ruminiclostridium_5	0	0.0045	0.000655	0.001225
Ruminiclostridium_9	0	0.0095	0.000879	0.002077
Unclassified_Clostridiales_vadinBB60_group	0	0.022	0.00631	0.007177
Unclassified_Desulfovibrionaceae	0	0.001	3.45E-05	0.000186
Unclassified_Eggerthellaceae	0	0.003	0.000138	0.000581
Unclassified_Firmicutes	0	0.0085	0.00081	0.002335
Unclassified_Prevotellaceae	0	0.006	0.000362	0.001369
Unclassified_Weeksellaceae	0	0.0115	0.000966	0.002525
Ureaplasma	0	0	0	0
Alcanivorax	0	0.024	0.006017	0.007507
Anaerotruncus	0	0.0065	0.000759	0.001521
Bergeyella	0	0.0395	0.005552	0.010219
Neisseria	0	0.243	0.009966	0.045021
Nosocomiicoccus	0	0.015	0.001862	0.003691
Ralstonia	0	0.052	0.011086	0.016406
Rikenellaceae_RC9_gut_group	0	0.006	0.000897	0.001561
Ruminiclostridium_6	0	0.007	0.001603	0.002068
Ruminococcus_1	0	0.0235	0.004862	0.005388
Unclassified_Gastranaerophilales	0	0.0085	0.001207	0.002437
Unclassified_Neisseriaceae	0	0.037	0.004241	0.009172
Unclassified_Pasteurellaceae	0	0.033	0.003103	0.006938
Alkanindiges	0	0	0	0
Bilophila	0	0.002	0.000138	0.000461
Dolosigranulum	0	0.0075	0.000483	0.001623
Enterorhabdus	0	0	0	0
Lachnospiraceae_FCS020_group	0	0	0	0
Pelomonas	0	0.0035	0.000121	0.00065
Prevotellaceae_NK3B31_group	0	0.0015	0.000103	0.000338
Ruminococcaceae_UCG.009	0	0.0075	0.000828	0.001952
Skermanella	0	0.0025	8.62E-05	0.000464
Unclassified_Coriobacteriales	0	0.001	3.45E-05	0.000186
Unclassified_Peptococcaceae	0	0.0045	0.000517	0.00125

Alloprevotella	0	5.00E-04	3.85E-05	0.000139
ASF356	0	0.025	0.003846	0.006938
Atopostipes	0	0	0	0
Burkholderia.Caballeronia.Paraburkholderia	0	0	0	0
Butyricicoccus	0	0.01	0.002231	0.002505
Candidatus_Saccharimonas	0	0	0	0
Corynebacterium	0	5.00E-04	7.69E-05	0.000188
Desulfovibrio	0	0	0	0
Enhydrobacter	0	0.01	0.0025	0.003096
Faecalibaculum	0	0.033	0.008962	0.010359
GCA.900066575	0	0.0045	0.001385	0.001293
Gemella	0	0	0	0
Haematobacter	0	0.003	0.000269	0.000832
Lachnospiraceae_UCG.001	0	0.025	0.008538	0.007227
Lachnospiraceae_UCG.006	5.00E-04	0.0115	0.004308	0.003717
Leucobacter	0	0	0	0
Micrococcus	0	0.0065	0.001346	0.00183
Mucispirillum	0	0.015	0.006846	0.00493
Muribaculum	0	0.005	0.001654	0.00139
Odoribacter	0	0	0	0
Oscillibacter	0.0025	0.038	0.010692	0.009863
Parasutterella	0	0.007	0.001462	0.002165
Prevotellaceae_UCG.001	0.002	0.019	0.010308	0.005345
Rikenella	0	0	0	0
Rodentibacter	0	0	0	0
Rothia	0	0.0215	0.003077	0.005919
Ruminiclostridium_5	0	0.007	0.002346	0.001819
Ruminiclostridium_9	5.00E-04	0.015	0.005692	0.004151
Unclassified_Clostridiales_vadinBB60_group	0	0.005	0.001423	0.001718
Unclassified_Desulfovibrionaceae	0	0	0	0
Unclassified_Eggerthellaceae	0	0.002	0.000538	0.000594
Unclassified_Firmicutes	0	0.0025	0.000769	0.000881
Unclassified_Prevotellaceae	0	0	0	0
Unclassified_Weeksellaceae	0	0	0	0
Ureaplasma	0	0	0	0
Alcanivorax	0	0	0	0
Anaerotruncus	0	0.0085	0.001731	0.00227
Bergeyella	0	0	0	0
Neisseria	0	0	0	0
Nosocomiicoccus	0	0	0	0
Ralstonia	0	0	0	0
Rikenellaceae_RC9_gut_group	0	0	0	0
Ruminiclostridium_6	0	5.00E-04	3.85E-05	0.000139
Ruminococcus_1	0	0.0025	0.000577	0.001096
Unclassified_Gastranaerophilales	0	0.0095	0.001769	0.002751
Unclassified_Neisseriaceae	0	0.001	7.69E-05	0.000277
Unclassified_Pasteurellaceae	0	0	0	0
Alkanindiges	0	0.0035	0.000692	0.001217
Bilophila	0	0.006	0.001577	0.001525
Dolosigranulum	0	0.002	0.000346	0.000591
Enterorhabdus	0	0.001	0.000231	0.00033
Lachnospiraceae_FCS020_group	0	0.0015	0.000577	0.000641
Pelomonas	0	0.0135	0.001731	0.003689
Prevotellaceae_NK3B31_group	0	0.0095	0.0035	0.003055
Ruminococcaceae_UCG.009	0	0.0075	0.001731	0.002058
Skermanella	0	0.001	0.000269	0.000388
Unclassified_Coriobacteriales	0	0.0015	0.000308	0.000522
Unclassified_Peptococcaceae	0	0.0025	0.000385	0.000712

Supplementary Table 2.5 Summary statistics of the abundances iof the unique genera within each population wild (n=203), HL-Lab (n=225), MPI-Lab (n=29), C57BL/6J (n=13). SD: Standard deviation

Unique Wild DNA genera	Min	Max	Mean	SD
Streptomyces	0	0.4162754303599	0.05616293858	0.07177371528
Unclassified_Actinobacteria	0	0.4442716433385	0.03789008217	0.05637794116
Saccharopolyspora	0	0.5328578706062	0.03397945208	0.08235766399
Leuconostoc	0	0.5688073394495	0.02149284623	0.07140093486
Unclassified_Pseudonocardiaceae	0	0.1454952434247	0.01795178533	0.02706902551
Unclassified_Enterobacteriaceae	0	0.8772912423625	0.01210700957	0.06786292109
Brachybacterium	0	0.1775585696671	0.0108257509	0.01724882903
Nocardiopsis	0	0.1665753424658	0.01039986969	0.01984810491
Dietzia	0	0.2760800842993	0.00919523824	0.02887105462
Clostridium_sensu_stricto_1	0	0.1370967741935	0.00768189545	0.02090496298
Gordonia	0	0.0932069510269	0.00736251543	0.0142306982
Kocuria	0	0.6719712525667	0.00693189847	0.04884278009
Pseudonocardia	0	0.0633626097867	0.00404417639	0.00701885344
Pantoea	0	0.0838287752675	0.00351458689	0.01001239047
Mycobacterium	0	0.0956429330499	0.00341287659	0.00945856232
Bacillus	0	0.1916846652268	0.00337814711	0.01462266171
Janibacter	0	0.0366705471478	0.00321761984	0.00634744957
Erwinia	0	0.1582138919515	0.00299878453	0.01247348417
Rahnella	0	0.1368700265252	0.0029026786	0.01140328327
Rhodococcus	0	0.0487012987013	0.00270028684	0.00631957082
Cupriavidus	0	0.0369783412573	0.00208041154	0.00472263484
Flavobacterium	0	0.0761670761671	0.00186330608	0.0074009149
Glycomyces	0	0.0277949351452	0.0018177228	0.00396173028
Bradyrhizobium	0	0.0325960419092	0.001524597	0.00361670278
Salinicoccus	0	0.0244318181818	0.00144600852	0.00344466074
Paeniclostridium	0	0.0249507550886	0.00130247044	0.00362968411
Nesterenkonia	0	0.067816091954	0.00121723386	0.00513229036
Unclassified_Nocardioidaceae	0	0.0181318681319	0.0012062758	0.00295883171
Methylobacterium	0	0.0121716848174	0.00119672927	0.00259278409
Stackebrandtia	0	0.0196961170512	0.00104518465	0.0025250763
Curtobacterium	0	0.0249091852621	0.00093623034	0.00251409554

Unique HL-Lab DNA genera	Min	Max	Mean	SD
Unclassified_Weeksellaceae	0	0.468347916	0.01284383286	0.04242138
Gemella	0	0.118008949	0.00711911795	0.01615377
Facklamia	0	0.297276265	0.0068723045	0.0263259
Deinococcus	0	0.249555424	0.00658522427	0.02039666
Ruminococcaceae_UCG.014	0	0.052997859	0.00492527205	0.01119654
Muribacter	0	0.052484645	0.00357327992	0.00835883
Unclassified_Corynebacteriales	0	0.088550479	0.00334879787	0.00879455
Helicobacter	0	0.037237237	0.00174940087	0.00485429
Corynebacterium	0	0.023489933	0.00174606332	0.00412598
Unclassified_Prevotellaceae	0	0.068167203	0.00167917925	0.00551129
Candidatus_Saccharimonas	0	0.015151515	0.00148665542	0.00268049
Unclassified_Desulfovibrionaceae	0	0.029923452	0.00098369052	0.00274187

Unique MPI-Lab DNA genera	Min	Max	Mean	SD
Alcanivorax	0	0.027894	0.0053322	0.00674923
Anaeroplasma	0	0.01133787	0.0015402	0.0032185
Anaerotruncus	0	0.01024811	0.0024617	0.00303381
Lachnospiraceae_UCG.006	0	0.00915856	0.0011361	0.00225814
Mucispirillum	0	0.04752172	0.0208931	0.01297022
Nosocomiicoccus	0	0.01025918	0.0023109	0.00317594
Oscillibacter	0	0.02641803	0.0074092	0.00795762
Parabacteroides	0	0.01301775	0.0024466	0.0034422
Porphyromonas	0	0.00977729	0.0013076	0.00248092
Ralstonia	0	0.02622378	0.0080908	0.0081789
Rodentibacter	0	0.02193995	0.002692	0.00505797
Ruminiclostridium_6	0	0.02437642	0.0078546	0.00636526
Ruminococcus_1	0	0.03661088	0.0162419	0.0094228
Unclassified_Bacteroidales	0	0.02629804	0.0020722	0.00521803
Unclassified_Gastranaerophilales	0	0.01241901	0.0026484	0.00422443
Unclassified_Rhodospirillales	0	0.01349892	0.0020531	0.00335467

Unique C57BL/6J DNA genera	Min	Max	Mean	SD
Actinomyces	0	0.027523	0.006914	0.009798
Alloprevotella	0	0.027186	0.00505	0.009676
Anaerococcus	0	0.12008	0.019889	0.032201
Chryseobacterium	0	0.022438	0.008238	0.009917
Dolosigranulum	0	0.029818	0.010296	0.01068
Fusobacterium	0	0.037631	0.010222	0.014625
Ignavigranum	0	0.050625	0.007572	0.014282
Kytococcus	0	0.024296	0.004792	0.008377
Pelomonas	0	0.191857	0.073919	0.049832
Peptoniphilus	0	0.067378	0.008563	0.019034
Skermanella	0	0.054579	0.015185	0.015332
Tepidimonas	0	0.042616	0.00555	0.012205
Unclassified_Rhodanobacteraceae	0	0.054054	0.007552	0.015577
Unclassified_Xanthobacteraceae	0	0.018332	0.004354	0.007171

Unique Wild RNA genera	Min	Max	Mean	SD
Actinomycetospora	0	0.2345741968383	0.00337128824	0.01809768745
Aeromicrobium	0	0.0220905172414	0.0012788958	0.00246411078
Anaerostipes	0	0.0496929089894	0.00381584164	0.00857169017
Aureimonas	0	0.0085007727975	0.00081918676	0.00163078663
Blautia	0	0.0461718293396	0.00280776088	0.00646188471
Brachybacterium	0	0.0884389984017	0.00603352783	0.00971489463
Christensenellaceae_R.7_group	0	0.0468836183122	0.00387468821	0.0073110833
Chryseobacterium	0	0.0163043478261	0.00065268769	0.00181267951
Clostridium_sensu_stricto_1	0	0.2265005662514	0.02063830512	0.04524621682
Collinsella	0	0.0146831530139	0.00114049812	0.00251716399
Comamonas	0	0.1078498293515	0.00202410525	0.01014722168
Coprococcus_1	0	0.0153089119738	0.00070253936	0.00186346316
Curtobacterium	0	0.031475748194	0.0013087964	0.00320311286
Delftia	0	0.0340846619021	0.00247661443	0.00418139215
Dialister	0	0.0666277030976	0.00620593885	0.01190625102
Dietzia	0	0.2702256466703	0.0125086086	0.02951131278
Dorea	0	0.0275785990072	0.00170302521	0.00398832687
Erwinia	0	0.2762162162162	0.00790198973	0.02336649563
Erysipelotrichaceae_UCG.003	0	0.0264754550469	0.00249094318	0.00496048822
Faecalibacterium	0	0.0309842041312	0.0021266047	0.00440120035
Frigoribacterium	0	0.0255047821467	0.00136639911	0.00319009218
Frondihabitans	0	0.0089192025184	0.00079773412	0.00181068715
Gordonia	0	0.1726990692865	0.00368398809	0.01413448904
Hymenobacter	0	0.0212765957447	0.00074715375	0.00203926737
Janibacter	0	0.0240460010455	0.00152827466	0.00378747252
Kluyvera	0	0.0464048954615	0.00266606795	0.00662488348
Lactococcus	0	0.0305793991416	0.00085328717	0.00264151725
Lysinibacillus	0	0.0119695321001	0.00067504174	0.00133216294
Marmoricola	0	0.0180131004367	0.00155392012	0.00268746305
Methylobacterium	0	0.0392156862745	0.00390742086	0.00730546166
Microbacterium	0	0.0292887029289	0.0017217459	0.00325611164
Mycobacterium	0	0.0343511450382	0.0026396874	0.00496022973
Nocardiopsis	0	0.2073910645339	0.01285590664	0.02743014078
Novosphingobium	0	0.014404432133	0.00093394242	0.002119445
Paenibacillus	0	0.025027203482	0.00137817875	0.00267855352
Paeniclostridium	0	0.048275862069	0.00541465115	0.00835514518
Pantoea	0	0.1195069667738	0.00472273166	0.01156125153
Pedobacter	0	0.0242173656232	0.00102425911	0.00265463152
Promicromonospora	0	0.0224425887265	0.00080746145	0.00248328516
Pseudogracilibacillus	0	0.0905700586042	0.00230951145	0.00944274714
Pseudonocardia	0	0.3947368421053	0.01662103118	0.05547159309
Rathayibacter	0	0.0190918472652	0.00064480893	0.00194231442
Ruminococcaceae_UCG.005	0	0.0243497509685	0.00123893161	0.00285036811
Ruminococcaceae_UCG.010	0	0.0103448275862	0.00053616627	0.00134164334
Saccharopolyspora	0	0.4326052210975	0.03597978904	0.07248140599
Salinicoccus	0	0.0495356037152	0.00151856694	0.00493528277
Sphingobacterium	0	0.0157987126975	0.00054816397	0.00158845415
Sphingobium	0	0.0135853514471	0.00058390015	0.00162151764
Stackebrandtia	0	0.0127142067441	0.00051126453	0.00143131266
Streptomyces	0	0.4782103428239	0.03453000145	0.05671850535
Subdoligranulum	0	0.0512961941533	0.00485118459	0.0089215523
Terrisporobacter	0	0.1729442970822	0.01162145882	0.03030590487

Unique HL-Lab RNA genera	Min	Max	Mean	SD
Alloprevotella	0	0.043203372	0.00259468761	0.00560697
Atopostipes	0	0.280667951	0.00769906089	0.02958661
Candidatus_Saccharimonas	0	0.012216405	0.00117936704	0.00253023
Desulfovibrio	0	0.069715561	0.00823627699	0.01272879
Leucobacter	0	0.082695253	0.00537754204	0.01139094
Rikenella	0	0.008479367	0.00077205964	0.00164598
Unclassified_Desulfovibrionaceae	0	0.01108871	0.00135637761	0.00224379
Unclassified_Prevotellaceae	0	0.045594578	0.00260278911	0.00497474
Unclassified_Weeksellaceae	0	0.052631579	0.00268273571	0.00635308
Ureaplasma	0	0.37377892	0.0634500573	0.0985099

Unique MPI-Lab RNA genera	Min	Max	Mean	SD
Alcanivorax	0	0.02950408	0.0071462	0.0091939
Bergeyella	0	0.03957916	0.0059629	0.01065828
Neisseria	0	0.25273011	0.0104267	0.0468107
Nosocomiicoccus	0	0.01591512	0.0020949	0.00400537
Ralstonia	0	0.06528562	0.0132087	0.01972646
Rikenellaceae_RC9_gut_group	0	0.00736196	0.0009995	0.00176948
Ruminiclostridium_6	0	0.00958702	0.001891	0.00254082
Ruminococcus_1	0	0.02829621	0.0054488	0.00615281
Unclassified_Neisseriaceae	0	0.03942461	0.0050293	0.01082897
Unclassified_Pasteurellaceae	0	0.03306613	0.0034287	0.00730984

Unique C57BL/6J RNA genera	Min	Max	Mean	SD
Alkanindiges	0	0.003659	0.000717	0.00126
Bilophila	0	0.006273	0.001614	0.001592
Dolosigranulum	0	0.002091	0.000355	0.000614
Enterorhabdus	0	0.001009	0.000234	0.000334
Lachnospiraceae_FCS020_group	0	0.001517	0.000587	0.00065
Pelomonas	0	0.01461	0.001823	0.003985
Prevotellaceae_NK3B31_group	0	0.010281	0.003594	0.003205
Ruminococcaceae_UCG.009	0	0.007568	0.001757	0.002076
Skermanella	0	0.001011	0.000271	0.000391
Unclassified_Coriobacteriales	0	0.001513	0.000313	0.000531
Unclassified_Peptococcaceae	0	0.002706	0.000402	0.000764

Unclassified_Actinobacteria	0	0.4449660928534	0.02321892782	0.0531615144
Unclassified_Alphaproteobacteria	0	0.0321329639889	0.00070379604	0.00264223596
Unclassified_Bacillaceae	0	0.0889717634523	0.0047505566	0.01179266343
Unclassified_Bacillales	0	0.0478781284004	0.00136203203	0.00442299778
Unclassified_Beijerinckiaceae	0	0.1029842012873	0.00978717176	0.01843969579
Unclassified_Burkholderiaceae	0	0.0275284260922	0.00164550844	0.0034078692
Unclassified_Chitinophagaceae	0	0.0100413467218	0.00080811335	0.00170413023
Unclassified_Devosiaceae	0	0.0124364047484	0.0009020044	0.00206625375
Unclassified_Flavobacteriaceae	0	0.0544584081388	0.00112429205	0.00459789052
Unclassified_Microbacteriaceae	0	0.0113058224986	0.00092659646	0.0019353219
Unclassified_Micrococcales	0	0.0061693774537	0.00059805985	0.00125845299
Unclassified_Nocardiaceae	0	0.0471204188482	0.00194003882	0.00627254337
Unclassified_Nocardioidaceae	0	0.0192198982476	0.00178384517	0.00307030165
Unclassified_Peptostreptococcaceae	0	0.0109529025192	0.00103397239	0.00178680261
Unclassified_Planococcaceae	0	0.0292188431723	0.00129912476	0.00322545663
Unclassified_Propionibacteriaceae	0	0.0074116305587	0.00053543134	0.00107886578
Unclassified_Pseudonocardiaceae	0	0.1518640350877	0.01917129162	0.0302372529
Unclassified_Rhizobiales	0	0.0113402061856	0.00089825531	0.00176091218
Variovorax	0	0.0090446579989	0.00079216434	0.00168277915
Williamsia	0	0.025627044711	0.00238160097	0.00472235482

Supplementary Table 2.6 Pairwise comparison of alpha diversity indices based on genera distribution across mouse populations: wild (n=203), HL-Lab (n=225), MPI-Lab (n=29), and C57BL/6J (n=13)

Index	Wild - HL-Lab	Wild - MPI-Lab	Wild - C57BL/6J	HL-Lab - MPI-Lab	HL-Lab - C57BL/6J	MPI-Lab - C57BL/6J
Shannon DNA	0.0124	0.0124	0.0027	0.2248	0.0514	0.0815
Chao1 DNA	$< 10^{-16}$	0.00243	0.00024	0.2171	0.04387	0.01096
Shannon RNA	0.62588	0.31778	0.00132	0.31778	0.00095	0.02593
Chao1 RNA	$< 10^{-16}$	8.90E-13	0.0002	0.27729	0.00039	0.00277

Correction for multiple testing was performed according to Benjamini and Hochberg (1995) [49]
