# Supplementary material for: Assessing similarities and disparities in the skin microbiota between wild and laboratory populations of house mice
Source: ISME J. 2020 Jun 9;14(10):2367–80. doi: 10.1038/s41396-020-0690-7 (PMC7490391; doi:10.1038/s41396-020-0690-7)
Supplement: Supplementary file 11 — Supplementary Table 3 [file 41396_2020_690_MOESM11_ESM.pdf]

Supplementary Table 3.1 Summary of indicator genera analysis in the standing and active communities. Only associations with **adjusted** p-value ≤ 0.05 and Stat ≥ 50 are shown . Stat: association statistics. Un: unclassified

	Genus	Group	Stat
**DNA Relative abundance**	Un_Clostridiales_vadinBB60_group	MPI-Lab	0.858796166582844
	Lachnospiraceae_NK4A136_group	MPI-Lab	0.845752163487589
	Cutibacterium	C57BL/6J	0.83016284353454
	Ruminococcus_1	MPI-Lab	0.817013454808965
	Pelomonas	C57BL/6J	0.802732421806609
	Un_Clostridiales	MPI-Lab	0.761383536670386
	Mucispirillum	MPI-Lab	0.733699027903692
	Un_Lachnospiraceae	MPI-Lab	0.703466914480783
	Ruminiclostridium_6	MPI-Lab	0.668201010304569
	Staphylococcus	Wild	0.661726419013892
	Skermanella	C57BL/6J	0.658562997180445
	Un_Muribaculaceae	MPI-Lab	0.644139688154011
	Muribaculum	MPI-Lab	0.607131691877166
	Corynebacterium_1	C57BL/6J	0.602823787637826
	Dolosigranulum	C57BL/6J	0.60054315321444
	Ruminiclostridium	MPI-Lab	0.592739938836835
	Oscillibacter	MPI-Lab	0.587743652411208
	Alcanivorax	MPI-Lab	0.585921446228656
	Ralstonia	MPI-Lab	0.585070056900232
	Un_Chloroplast	HL-Lab	0.580064300859302
	Streptomyces	Wild	0.544874962505346
	Anaerotruncus	MPI-Lab	0.543853261524233
	Chryseobacterium	C57BL/6J	0.543662107536935
	Fusobacterium	C57BL/6J	0.533330792835739
	Brevibacterium	Wild	0.531011166792621
	Nocardioides	C57BL/6J	0.518627834989225
	Prevotellaceae_UCG.001	MPI-Lab	0.500939069750195
**DNA Presence absence**	*Streptomyces*	Wild	0.913728826100542
	Ruminococcus_1	Lab_MPI	0.877533573668443
	Skermanella	C57BL/6J	0.870476689143729
	Pelomonas	C57BL/6J	0.86547470128168
	Un_Actinobacteria	Wild	0.824833175692198
	Nocardiopsis	Wild	0.824093517396923
	Un_Clostridiales_vadinBB60_group	Lab_MPI	0.817261386240846
	Brachybacterium	Wild	0.809843286945495
	Saccharopolyspora	Wild	0.791152703257882
	Alcanivorax	Lab_MPI	0.790569415042095
	Un_Pseudonocardiaceae	Wild	0.765504043894372
	Gordonia	Wild	0.756205995302456
	Brevibacterium	Wild	0.706024014596831
	Ruminiclostridium_6	Lab_MPI	0.695749318896161
	Ruminiclostridium	Lab_MPI	0.644003778310813
	Mucispirillum	Lab_MPI	0.632696974558857
	Pseudonocardia	Wild	0.630313496268647
	Janibacter	Wild	0.612530710030856
	Prevotellaceae_UCG.001	Lab_MPI	0.610397317907679
	Dietzia	Wild	0.60282423485803
	Rikenellaceae_RC9_gut_group	Lab_MPI	0.599983400606413
	Dolosigranulum	C57BL/6J	0.599332309109241
	Campylobacter	Lab_HL	0.590744111794041
	Oscillibacter	Lab_MPI	0.587434394085728
	A2	Lab_MPI	0.578485462635546
	Un_Clostridiales	Lab_MPI	0.576191383284768
	*Clostridium*_sensu_stricto_1	Wild	0.575061183386095
	Anaerotruncus	Lab_MPI	0.552300681324829
	Fusobacterium	C57BL/6J	0.545096497512336
	Un_Rhodospirillales	Lab_MPI	0.541391400668242
	Leuconostoc	Wild	0.537113651418793
	Glycomyces	Wild	0.523634938088643
	Ralstonia	Lab_MPI	0.520135048791502
	Nocardioides	C57BL/6J	0.518765077325971
	Rahnella	Wild	0.501044735778258
**RNA Relative abundance**	Prevotellaceae_UCG.001	C57BL/6J	0.752262407808561
	Staphylococcus	Wild and C57BL/6J	0.717251561015568
	Aerococcus	C57BL/6J	0.678252108047774
	Prevotellaceae_NK3B31_group	C57BL/6J	0.630151667528785
	Bilophila	C57BL/6J	0.594403962973657
	Alcanivorax	MPI-Lab	0.575824691567237
	Un_Clostridiales_vadinBB60_group	MPI-Lab	0.532305105963443
	Burkholderia.Caballeronia.Paraburkholderia	MPI-Lab	0.531735226352294
	Ruminiclostridium	C57BL/6J	0.530783099630432
	Alistipes	MPI-Lab	0.528635002626643
	Faecalibaculum	C57BL/6J	0.521506805204402
	Streptococcus	MPI-Lab	0.512137429952805
	Oscillibacter	C57BL/6J	0.511345029455417
	Ralstonia	MPI-Lab	0.501580529125575
**RNA Presence absence**	*Nocardiopsis*	Wild	0.878205296292439
	Saccharopolyspora	Wild	0.876320753761767
	Streptomyces	Wild	0.863198819921201
	Un_Pseudonocardiaceae	Wild	0.847850619709344
	Dietzia	Wild	0.834571664645666
	Bilophila	C57BL/6J	0.832733036283729
	Erwinia	Wild	0.796675115090713
	Delftia	Wild	0.789906100849759
	Brachybacterium	Wild	0.787052741224574
	Un_Actinobacteria	Wild	0.728396661004768
	Paeniclostridium	Wild	0.712289347416263
	Alcanivorax	MPI-Lab	0.704700403567909
	Un_Nocardioidaceae	Wild	0.695836489784628
	Mycobacterium	Wild	0.693786704588114
	Kocuria	C57BL/6J	0.692812472120439
	Pseudonocardia	Wild	0.674859749325068
	Terrisporobacter	Wild	0.667491239234977
	Prevotellaceae_NK3B31_group	C57BL/6J	0.6604367872539
	Ruminococcaceae_UCG.009	C57BL/6J	0.657815189241259
	GCA.900066575	C57BL/6J	0.655037805640859
	Gordonia	Wild	0.65049613290346
	Clostridium_sensu_stricto_1	Wild	0.648856436290832
	Un_Beijerinckiaceae	Wild	0.645074963063321
	Brevibacterium	Wild	0.644797463339129
	Un_Xanthobacteraceae	Wild	0.642407809876191
	Pelomonas	C57BL/6J	0.641567020370538
	Un_Gastranaerophilales	C57BL/6J	0.631012569652621
	Kluyvera	Wild	0.611523686243276
	Lachnospiraceae_UCG.006	C57BL/6J	0.609049162710068
	Ralstonia	MPI-Lab	0.599732571080389
	Un_Peptostreptococcaceae	Wild	0.59913693416974
	Faecalibaculum	C57BL/6J	0.597728389682811
	Erysipelotrichaceae_UCG.003	Wild	0.590502441959341
	Anaerotruncus	C57BL/6J	0.588909424720391
	Salinicoccus	Wild	0.580505212181478
	Un_Bacillaceae	Wild	0.577249626823705
	Burkholderia.Caballeronia.Paraburkholderia	MPI-Lab	0.572895193604212
	Pantoea	Wild	0.571282384274463
	Paenibacillus	Wild	0.570492416375115
	Un_Enterobacteriaceae	Wild	0.56769853249562
	Ruminiclostridium_9	C57BL/6J	0.566697863120993
	Ruminiclostridium_5	C57BL/6J	0.566236534273938
	Lachnospiraceae_FCS020_group	C57BL/6J	0.565573295182311
	Actinomycetospora	Wild	0.548327093050508
	Bergeyella	MPI-Lab	0.545347955533364
	Un_Devosiaceae	Wild	0.544377088122812
	Christensenellaceae_R.7_group	Wild	0.541036839124868
	Aeromicrobium	Wild	0.53573415635574
	Oscillibacter	C57BL/6J	0.533340011590263
	Curtobacterium	Wild	0.53114343805599
	Un_Bacillales	Wild	0.53024060253676
	Turicibacter	Wild	0.525990261443044
	Williamsia	Wild	0.523127424424832
	Collinsella	Wild	0.510427139095315
	Ruminococcus_1	MPI-Lab	0.509115993360192
	Dialister	Wild	0.506402016401459
	Subdoligranulum	Wild	0.504234388923649
	Anaerostipes	Wild	0.501316411516462

Supplementary Table 3.2 Importance components of the RandomForest framework based on DNA and RNA core genera

Core genera DNA	C57BL/6J	HL-Lab	MPI-Lab	Wild	MeanDecreaseAccuracy	MeanDecreaseGini
Streptomyces	428.109185090393	746.7834099276	498.5514645132	537.39757320149	805.04917969974	25.84792157114
Unclassified_Chloroplast	13.0251214101726	870.6784234665	404.0397643159	598.63249464247	966.178046783825	21.3193364618158
Brevibacterium	377.965644535547	551.0264132452	163.4769302978	431.66349182688	636.033995388991	17.3912410410146
Unclassified_Actinobacteria	213.832422401982	479.7138519911	391.6997661825	373.65177731033	537.647130178706	15.9850535644966
Brachybacterium	317.222238879419	499.0324217612	285.8178742727	366.64142296643	525.133887056949	14.8547826945967
Nocardiopsis	281.321505506036	492.0767449952	343.1641414893	299.59167499476	503.569586999281	12.543360706895
Staphylococcus	100.554323943329	502.8648527671	184.2545789116	362.55413784183	580.532820121749	10.960038927713
Campylobacter	292.294778653892	369.655961098	20.31164591104	452.57793829723	509.332030250984	8.54275481423388
Gordonia	229.064541575051	484.7541642898	286.1677463498	252.19346323682	483.687825126936	7.72378449263129
Saccharopolyspora	235.234804497888	379.6769298298	281.8187787768	189.39734105643	395.910472628737	7.70783444436699
Unclassified_Pseudonocardiaceae	209.425950814647	378.8531827188	256.3812511652	153.91383198818	390.965163155659	6.52664643619278
Unclassified_Clostridiales_vadinBB60_group	148.415094154208	378.3971880727	531.1386896377	216.46802297397	567.654454836069	6.06066880237641
Lachnospiraceae_NK4A136_group	54.8051927734639	230.3909726772	404.241266728	274.39120306939	445.026624131633	5.64424681762481
Unclassified_Lachnospiraceae	66.6465687385319	248.7037550742	359.74927497	400.4871554605	484.492469137533	5.53138480727649
Ruminococcus_1	127.270951053287	372.1073854951	474.7199440281	201.51534707442	502.196385940605	5.39189150757774
Burkholderia.Caballeronia.Paraburkholderia	53.9431815484359	539.6021262749	27.1067818124	344.26286694589	572.44596977776	4.98718330066153
Dietzia	92.8784719077736	437.5022523485	239.247490837	212.22530790776	437.396263882175	4.74026492892648
Unclassified_Muribaculaceae	87.8960856403909	363.8707263935	321.0570602809	240.66500071638	447.92852480678	4.64557233052818
Pelomonas	559.217734553253	433.3928167387	50.42487561944	147.95006539147	550.341279475784	3.93949761400004
Mucispirillum	-40.3639815624591	278.1913349796	380.8087887978	185.06738344816	412.161875676194	3.77274876511849
Alcanivorax	91.2576744230801	425.7221046966	407.4935056526	171.92608158091	465.376687142811	3.6685039626709
Cutibacterium	409.86595087191	258.4859882274	106.1500509136	244.60362836555	441.472531620069	3.19759708742721
Unclassified_Clostridiales	-20.9238512014771	153.6167922654	336.8502971385	203.70454076408	369.400214167434	2.67933278559446
Ureaplasma	127.590783892988	251.9448227207	127.9733225434	353.74617551319	373.707231989013	2.41375606158695
Pseudonocardia	131.652841714421	205.9563830638	145.1478316042	130.72330527282	225.564229749047	2.32417293340955
Skermanella	361.403646381155	200.6567003448	81.22351504763	95.507971434495	362.959733523698	2.06051894956334
Bacteroides	49.3084211946724	188.2493735567	190.4484485641	215.44585614064	272.721634691419	1.9553510890087
Alistipes	-8.37750449133275	167.4880548026	287.9610330636	191.87990253683	333.240172973003	1.90816323760293
Janibacter	109.017042148058	216.1839967123	150.3478753844	42.524383904498	220.678643368262	1.84042105197717
Ruminiclostridium	-19.0418716632127	99.58488058265	288.5283728526	218.52577228875	323.585530971161	1.80124067146103
Halomonas	196.956026604849	255.7119078658	278.4741976551	112.71803601359	301.418994930761	1.78687857570981
Unclassified_Bacteria	112.937149374299	204.9612798327	87.5776009398	281.53625234563	310.351144904639	1.73820377885065
Clostridium_sensu_stricto_1	115.219764657615	249.9597815952	137.2841231235	173.98191535843	262.162505623905	1.61333210255906
Ralstonia	81.9564627767914	255.9147127642	231.8594016705	58.984837275033	298.304629199082	1.61291043514842
Prevotellaceae_UCG.001	59.6588475916896	103.3025477267	228.5665050399	143.90285525101	271.17189625871	1.48257002388168
Streptococcus	140.964898061905	93.98803036954	17.51366187745	165.78358042883	200.556145795718	1.22969410281215
Muribaculum	118.431141630493	140.6018376023	182.2539598728	188.79163920469	273.888772065616	1.21766377109419
Aerococcus	55.8489612775084	138.8941680714	176.6609436418	173.38784148428	237.573566068227	1.16652639210426

Unclassified_Weeksellaceae	87.5176822009982	109.832662801	78.03619880956	226.48574929901	240.890156990329	1.13527981894031
Parasutterella	103.870976548719	166.4356586469	23.44911555587	209.81237519379	250.036075837146	1.10012429108055
Gemella	18.7066497120361	127.0232347173	13.916402924	272.84269569884	281.682547081492	1.0686686173361
Glycomyces	88.7788634474478	251.6401717281	114.7547903891	67.96303089381	250.111246731962	1.06227072380602
Shewanella	124.226937972975	172.3568906685	202.0805662225	134.02367366963	249.993431452093	1.05222088407237
Dolosigranulum	194.854078683257	83.76771480558	-6.545906035293	155.44573777463	228.608298954653	1.03117671465293
Unclassified_Betaproteobacteriales	135.747173176414	166.9300831898	146.7053290301	142.47044436013	235.904950540669	1.02728465803065
Unclassified_Micrococcales	66.4734749651595	294.7469937249	110.6585965752	1.7564542683307	294.582587455712	1.01788083534061
Mycobacterium	31.5888166328929	209.4443868646	108.8279193484	52.787847605621	206.604759786067	0.999213850221694
Rahnella	84.7580994928664	268.6608133292	106.0087034918	97.828486965239	268.933082681142	0.978095157536373
Leuconostoc	81.1182470287162	234.7460031468	93.02902231173	129.39051190233	248.793507379608	0.930085536684582
Lachnospiraceae_UCG.001	-10.4233667328821	92.17848466595	48.21931639842	263.8022597044	266.931288994999	0.902012908229633
Massilia	176.239495715264	285.2227915219	67.85667745084	-1.093878857373	288.140902200445	0.884464790043921
Corynebacterium_1	160.627140661179	103.555904268	142.2606332689	55.372901068552	214.204492101961	0.847932907353901
Anaerotruncus	-26.6011824278502	144.0191494176	124.0164169277	83.617851524038	178.642339287691	0.804969864277008
Anaerococcus	168.153241403915	-13.3357307901	51.52243728659	83.299112434199	137.545530576585	0.735701810751511
Unclassified_Ruminococcaceae	20.5220288240006	46.62678321359	83.67928292044	136.31905165978	152.021968509759	0.714834972325692
A2	-5.69887141423445	-0.22375440674	130.7100224792	187.83675971888	207.258517329285	0.708599696564619
Lactobacillus	66.24702050759	187.4450683716	-14.04345277561	49.594675158606	188.606695786426	0.706149570372256
Ruminiclostridium_6	71.6355487254092	32.56246114176	145.6003590115	92.946085147987	157.059769642311	0.677329402246498
Unclassified_Halomonadaceae	80.3732136131689	133.0041169845	162.2784610393	97.074542376601	204.001667111692	0.663671782387043
Oscillibacter	24.7835003033169	77.54898641125	127.9608768	135.49239727591	178.226952039901	0.633057315100046
Faecalibaculum	68.610744972862	85.94317450991	48.18663585737	156.18681488162	182.096984736045	0.62963537536166
Fusobacterium	157.970484776403	140.5233304659	-1.088237866012	34.998594897264	185.448865800709	0.604968623854798
Nosocomiicoccus	-8.75525034534385	29.83360425984	90.75989492297	212.47103540558	213.584840818324	0.576662783262747
Romboutsia	113.412764356541	88.97418410969	134.2403155393	71.278667506419	169.115008939158	0.542947150410483
Haematobacter	54.4316340434973	26.71917187691	7.500797741847	103.68309684868	110.49219337906	0.534144666286937
Acinetobacter	7.65489654788599	96.45868425774	8.039804903337	39.781115003458	99.6801891029134	0.533684445055841
Desulfovibrio	73.9169639757264	117.9597809673	37.93299389908	124.7141270526	164.006454504545	0.521423290006808
Unclassified_Enterobacteriaceae	60.8156434720129	107.9870150862	12.33896651797	57.161663017199	119.13783203996	0.500208753888117
Paracoccus	57.5827638161515	8.286780613765	44.41086078965	108.21189794153	112.595764339954	0.489699862545774
Curtobacterium	-8.08616200878931	186.1527296921	63.73784053776	36.598812573121	183.131900699763	0.47973218128593
Nocardioides	104.75574225092	31.0212552953	54.32877108929	69.222457849798	111.215775751475	0.471713077540297
Actinomyces	84.7726220722655	12.51964542798	-25.19284765519	111.82933988253	117.65741436154	0.465419224417674
Roseburia	-13.3158089533168	20.94199036107	4.255828973408	186.40817269074	183.706453886007	0.456212986231386
Enhydrobacter	66.3084516076667	22.89969070039	-25.34119929994	143.55645708752	143.165985194666	0.434521797924564
Pseudomonas	-20.3579783549716	61.68479153395	21.29187705093	12.54373898935	54.8608611904025	0.419245591376278
Ignavigranum	-9.19455805572401	107.4458304637	58.86387649763	178.75333960485	192.729318957492	0.41612572998754
Rikenellaceae_RC9_gut_group	62.8296687884289	56.97502499543	62.54978941734	106.9785372962	140.577852160507	0.406654997484349
Amaricoccus	77.8185349311371	-21.4723773924	-20.00113945574	101.56473197529	86.2971597059721	0.402686475794906
Muribacter	47.7547292297231	43.79260911896	2.547135797609	150.78192887228	152.706876693906	0.398810891191449
Tepidimonas	61.1924462908127	23.89369019663	-11.56954461469	89.065973625793	90.5612612838562	0.396977578882151

Kocuria	11.5849626787375	120.167399623	49.64863481176	23.129841639432	123.092541636102	0.393640807031284
Chryseobacterium	106.284477630708	62.64380493752	47.39368898783	36.654598235048	119.00737912719	0.387446456000939
Cupriavidus	39.6790356627975	130.2275194899	-13.91292699964	61.437664586377	133.704502581246	0.379079026977636
Nesterenkonia	47.3341368918546	148.03732548	66.63776701227	1.4092634685557	146.251848203478	0.376213469667618
Rodentibacter	20.742009264175	41.73090032561	-17.02948561837	211.33865928807	208.122719161553	0.369008088128415
Veillonella	47.1058129945735	11.62872996145	-7.824785458993	111.9964422131	111.268737970372	0.367630610062132
Kytococcus	83.5062694773575	78.9682346751	-0.471917916239	37.318003849418	112.386560595874	0.353167392671262
Candidatus_Saccharimonas	50.5416025816676	62.17918179818	50.74677841916	142.6468464761	156.840693292939	0.343952791779477
Rothia	-13.9907309978841	39.84979660859	-4.417624047302	139.27259379347	134.657032671667	0.322429277604383
Salinicoccus	9.39402747546257	139.7045502049	57.51280189832	39.775543362791	143.62650609621	0.322120618696147
Turicibacter	19.2898302572111	50.87701219439	104.9951149574	41.899513083768	112.272217291246	0.313022489022456
Micrococcus	-17.3475881026941	11.17660225478	20.53710976472	123.37999952068	115.740135864806	0.309819538371867
Unclassified_Nocardioidaceae	-19.4701353450688	184.2187228268	18.16271044776	-18.00196717596	182.426123880356	0.29496823780797
Unclassified_Rhizobiaceae	1.35062477491798	48.09410573954	129.4917698659	15.670163371136	119.575954416676	0.290666582268868
Prevotellaceae_NK3B31_group	73.4839185921488	26.04556819863	-22.59175987613	75.344291726538	85.1871712161999	0.287074089889702
Deinococcus	47.5398120497664	49.70707505647	23.83650685781	115.97544044964	130.256217553898	0.271351367804401
Parabacteroides	46.4975891484632	67.5417536787	20.0871511862	111.86398271652	132.906383031614	0.268310395230673
Ruminococcaceae_UCG.014	54.6527495587538	95.74055647214	11.58521247019	78.026986067846	125.425150912389	0.267425594013045
Pantoea	37.5507917458831	140.2374039228	47.3269253047	52.078900301956	143.305189757257	0.264329371925186
Unclassified_Rhodospirillales	29.1099763421019	14.39614346065	74.40861598146	90.592426227985	99.8401526358226	0.256150700587341
Stackebrandtia	39.5265112280853	131.5182391972	54.24218206047	-7.421261563651	130.359827647437	0.255662543144761
Corynebacterium	-11.5722936031765	17.17094697913	-17.6709247853	101.50599490391	90.8861693661979	0.249623755201128
Porphyromonas	-20.4787718944327	43.38322185509	51.72212049059	39.654423013132	65.2440339267531	0.24443189054029
Alloprevotella	12.8147457841213	38.12630516624	37.63448405162	109.35527899703	115.658578716674	0.243649080672322
Unclassified_Rhodanobacteraceae	54.5315322994577	49.24686695258	14.2985547372	17.617328157533	70.2441493499088	0.239436994080223
Erwinia	36.7265341671047	100.1911855045	45.57054585829	39.474371485169	104.063916821694	0.23574050584222
Odoribacter	2.0103800007829	77.55397011073	10.60813830919	79.998321079911	107.890436966367	0.233772754557817
Microbacterium	38.061218081002	35.15113402491	2.763484916455	11.289765734528	47.3453046800064	0.233419220692812
Unclassified_Rhodobacteraceae	-13.686053816969	16.63153680956	24.06163134026	52.499021270861	52.242722435545	0.228239863142177
Facklamia	12.426858798942	41.74706463281	32.33556912081	119.20508069984	126.071190699801	0.219945550334951
Ruminiclostridium_9	-14.2162248488613	18.26185696908	27.67785705711	114.53858631454	113.791916683801	0.218254731124205
Paeniclostridium	34.1049557055857	103.3439682846	48.20122636782	16.656655477306	103.793828058856	0.205615864646879
Lachnoclostridium	11.6325742259418	34.91441594955	36.79166098643	78.185272298712	90.8685402839686	0.204824788630535
Peptoniphilus	61.7423149860179	-17.7717804506	21.48121314347	70.714272290948	68.1944903111739	0.202608478489804
Jeotgalicoccus	52.7695223873268	27.52947835224	42.23642340343	74.707260746827	96.5440204930456	0.196725638710411
Flavobacterium	9.37945044652361	101.3123225903	2.519029081874	-2.604161722016	92.7517044758251	0.194017234763634
Unclassified_Prevotellaceae	34.6369565574656	20.57872319751	23.29630113479	87.454577208177	90.4519596264347	0.192188175349418
Unclassified_Burkholderiaceae	-16.0259868032909	35.95827084436	3.879562921041	8.1300776499459	29.2948335149792	0.189923409577598
Rhodococcus	12.855850396244	104.5454257371	41.20694335962	44.00204933005	111.421162192646	0.183503085957699
Unclassified_Desulfovibrionaceae	16.5283756733922	77.18850173969	68.78458925048	84.034096945257	122.01498306048	0.179951485681421
Sphingomonas	2.60792322350946	43.2240218912	26.31406775888	-4.899937407502	36.3070985156458	0.178047003028998
Brevundimonas	11.2663883319596	-19.3041562558	-14.64961227121	47.690441043146	21.947372149869	0.174456910536231

Unclassified_Corynebacteriales	27.2473395714728	41.10001118752	77.37293113365	51.491292258923	93.9343845329532	0.173289846772189
Unclassified_Gastranaerophilales	20.3105801555933	30.37660173114	10.58952729119	74.589385749658	77.131613639639	0.170703253806834
Unclassified_Bacteroidales	-3.82717142909702	-12.2926223993	22.0045572165	66.965982643748	49.8764373220053	0.166668904862313
Unclassified_Sphingomonadaceae	3.2635203911616	17.46087380253	0.463776434009	10.178603038207	18.9912409249311	0.160360042993607
Bacillus	16.7987117670386	69.93697958773	-3.630477357294	15.448311001092	68.5229171667275	0.147299550782358
Helicobacter	35.0096532907724	38.3976440482	-2.059145151926	25.040158191158	48.143004847379	0.145982442860533
Anaeroplasma	11.0452586226643	31.17310854057	46.16276020841	24.661087913985	54.833186485913	0.12289530204609
Methylobacterium	34.0540516723206	25.35915233523	8.570301209941	33.227401340051	46.8128650646812	0.121186188376612
Lachnospiraceae_UCG.006	-2.10365969114819	1.140592414429	-3.01599086327	58.982115209164	47.0812919876756	0.119841136080776
Unclassified_Xanthobacteraceae	10.9735519834652	-4.31132024864	36.38546729424	22.314567709452	24.4844484271079	0.114786817403476
Bradyrhizobium	29.879730102264	14.85803466896	44.35902403091	25.56180875167	48.4560230516904	0.11248077586671

**Core genera RNA**	**C57BL_6J_RNA**	**Lab_HL_RNA**	**Lab_MPI_RNA**	**WildRNA**	**MeanDecreaseAccuracy**	**MeanDecreaseGini**
Streptomyces	295.194085925424	498.8732228323	335.7646521999	415.72168503514	534.919872009067	17.1187048390264
Staphylococcus	429.687330472001	625.4924499476	420.6030183195	309.53076485131	677.600901504251	12.7907957903763
Nocardiopsis	282.164196779504	431.5054393369	333.5651909941	316.86518909956	441.9057566453	11.8270151119996
Saccharopolyspora	281.25767931808	477.1655072745	347.5404168972	347.21304626559	497.523865587356	11.7250693136862
Unclassified_Actinobacteria	162.740843712291	369.9142440353	146.3512756532	337.58973379819	414.710032313347	11.6153214645714
Brachybacterium	162.806993353688	327.5004519235	311.0583055464	299.39867640089	366.16989907278	10.8326523881231
Streptococcus	522.901214388667	619.5116166956	424.5737291971	298.35552103879	684.76059995474	10.2795760790905
Unclassified_Pseudonocardiaceae	243.847991146949	383.4238786384	301.3324546898	269.20039509815	399.490502581324	9.03235022749164
Dietzia	254.363199367276	411.7407492216	302.799041286	273.37900376446	413.189459049058	8.71944026202439
Unclassified_Muribaculaceae	242.349842586215	345.5583097553	203.8197948106	348.66822553799	437.51026107684	8.1866597927381
Delftia	226.984232733671	393.2403920612	280.9516914866	286.41825828968	404.45603957458	6.27503767412557
Alcanivorax	123.389764718705	562.8997034349	565.7372767496	135.47426154697	602.2037419194	5.42930997934189
Erwinia	201.065506375415	279.4381790008	236.1825226214	210.1585356076	301.472973580228	5.29087413805569
Pseudonocardia	69.7865291689974	304.8774885862	228.4557770053	178.23289115845	312.212810650201	4.66702425384171
Paeniclostridium	142.576474388544	336.8922177796	228.4789555898	148.41714234046	335.912866076053	4.55886464134489
Lachnospiraceae_NK4A136_group	183.58639077321	216.5791725072	162.418718811	297.74618434302	347.31554680287	4.47492586251669
Unclassified_Enterobacteriaceae	179.529983489834	265.3476436299	85.80599953261	197.85241571534	295.035380407403	4.09769319884886
Brevibacterium	185.628059671583	199.0056839117	8.989226179218	187.43728632036	232.300575121266	3.88568151258464
Bacillus	216.767291534771	396.3010577382	213.8375204585	124.70463297197	401.279721086868	3.58272287692564
Campylobacter	286.790730846702	312.5219484471	151.2864781136	211.08044053212	343.889950320067	3.11827624030017
Prevotellaceae_UCG.001	329.271535871058	203.9791719409	86.6576642511	210.49878147747	372.514736161429	2.83521740060963
Unclassified_Beijerinckiaceae	52.5592631226686	228.973650987	143.1462864432	110.74386685855	241.447288639214	2.72523846904041
Muribaculum	93.5780400692448	205.2826342107	27.92279935409	303.26458679664	325.211040397829	2.63592081389545
Clostridium_sensu_stricto_1	101.913238177641	222.3719256322	113.9566953367	171.14461879034	233.975604304374	2.62740034393785
Bergeyella	83.0568365654882	332.2021963927	329.3106745647	59.418856847324	378.194531544635	2.38110692908646
Unclassified_Clostridiales_vadinBB60_group	47.6210667639305	294.7591157652	351.8668198327	94.610778494202	379.355604902599	2.32129367000119
Terrisporobacter	146.088268444579	297.7852909386	181.7835638312	195.05073433979	298.493846704926	2.31124044121245

Mucispirillum	101.854066140994	198.7726851645	275.7431170044	173.29680323075	345.633598628539	2.28784714229403
Gordonia	98.622064484858	201.7438246162	165.7198902315	107.42380995828	209.374547904599	2.2419083429438
Unclassified_Chloroplast	263.404466888374	327.4311385599	74.30754161829	128.01689609122	373.001911214262	2.00252276746446
Unclassified_Nocardioidaceae	118.713422075103	194.5165204537	147.0758390651	62.599930681419	197.177730873168	1.88639200150321
Unclassified_Pasteurellaceae	43.3352978919493	340.4361652607	357.7639130304	79.715953741622	391.643848534128	1.81131866975
Lachnospiraceae_UCG.001	65.9826548091086	169.6137552662	59.14821830283	241.2733030313	270.345293915026	1.80927084530747
Unclassified_Carnobacteriaceae	92.4483423904326	293.3371230663	308.8306821566	32.52611275014	338.97463099749	1.75206219977717
A2	170.562915201233	155.4568296696	105.4866790819	270.16596989648	303.853888023233	1.66385308381134
Alistipes	73.7132393893074	69.83376469122	255.1267293855	121.51023385422	251.973949008957	1.646074267598
Ralstonia	65.1276435267404	132.6314692514	274.3899198234	85.229816296113	276.194221190181	1.59122006582615
Stenotrophomonas	104.491174673489	318.4884670354	210.8355775047	2.1681287947901	333.266961916799	1.57886908910195
Erysipelotrichaceae_UCG.003	122.419668638534	245.3731541144	159.690594694	205.22128982775	260.358735600526	1.55048534644233
Dorea	122.158789408356	324.0016173648	-8.238697707975	234.97485264336	332.747170640117	1.54375342771556
Rodentibacter	54.1447917790431	161.9697739743	268.6748147037	169.84847116624	272.322404232853	1.47657749983341
Unclassified_Bacillaceae	113.881914373525	201.4148322068	-17.83010735176	95.400681099005	201.051400668959	1.44049377115003
Unclassified_Gammaproteobacteria	58.1456948768978	201.1670345875	302.8902096576	76.443514800084	311.019569615773	1.43491041434447
Unclassified_Gastranaerophilales	210.272239491213	231.2705513397	23.1084200982	44.486394966203	263.946260644455	1.41468571569549
Paenibacillus	114.206446357046	253.6281303372	21.64590922622	67.925705729045	250.774622790931	1.39382261367347
Mycobacterium	99.4962582547421	125.8981069626	118.2390769021	64.234401707013	140.862490731707	1.28341841235118
Christensenellaceae_R.7_group	106.051822580848	211.1713093376	49.57062712325	189.93010926689	234.938028498028	1.25383506064358
Pantoea	106.756373957622	197.2535140787	-31.6965993482	60.313979661047	196.686186991453	1.23333425182391
Burkholderia.Caballeronia.Paraburkholderia	65.1130473366174	75.30807417837	196.9796104699	171.91885848891	241.851963247253	1.21941691294154
Neisseria	40.6680146336571	286.2125290852	205.4211388032	-0.876973842259	294.413924330936	1.16020523743518
Halomonas	139.772587843936	237.1955761511	179.0244246945	178.95890851056	299.737185844241	1.11885552113716
Unclassified_Xanthobacteraceae	112.935544112475	107.0593214307	97.01757907474	143.64811496438	161.119896562681	1.1012368643092
Aerococcus	242.553043957307	79.89665620882	153.6777766143	141.76844403632	270.659986183057	1.10063037615448
Collinsella	93.7489445291276	215.0223376587	134.0621505445	205.16405597661	239.191094132815	1.07787102150119
Ruminococcaceae_UCG.009	142.172198783539	143.6198993346	50.04720690967	89.349843164528	205.930712360986	1.02295171542141
Kluyvera	97.7900303074109	202.6827117194	125.9022070182	81.097422129627	202.381526196579	1.00409773227646
Lachnoclostridium	23.4248149727932	188.7815314132	99.92818498117	141.69806999165	216.790165117437	0.983402664865863
Bilophila	247.040261880929	41.19767471355	-43.8864384517	83.517747633929	216.996178322998	0.97423319802506
Unclassified_Clostridiales	81.9955444441315	128.8586893529	178.1440013206	101.52263073184	226.65855151191	0.974188528747743
Cutibacterium	32.7953643782705	194.5683840673	86.22629097657	180.69047813329	243.027935988295	0.970727239664759
Unclassified_Bacteria	101.26755780103	151.6664085435	63.93786672212	203.57823739658	240.111367184263	0.949017779931482
Ruminiclostridium	186.961436974122	127.8435027852	43.75892514469	179.03698575394	250.415004692468	0.944700925689458
Massilia	265.401756058588	185.0536020682	138.1740945317	-0.699312606615	254.915368615073	0.939599622978511
Desulfovibrio	89.8914687351299	166.053975072	83.33832570972	215.84135985104	247.409696749098	0.915582964163737
Butyricicoccus	103.390407922599	131.1242195492	97.2516826087	215.8851893993	245.066512562013	0.903343863036328
Unclassified_Ruminococcaceae	37.3924721737625	194.686778522	106.5531015266	121.32933445409	230.228962431647	0.897189647971697
Gemella	78.4253857983133	105.225783531	67.93580136524	190.14478851466	212.234898570218	0.881949368382769
Coprococcus_1	89.2465812563071	237.3410296884	121.9703302372	173.68529933982	241.490879024535	0.865343552098531
Oscillibacter	178.89664980271	124.5841628246	-14.27401604694	189.49354690194	239.322671796676	0.8605396989708

Parasutterella	26.4456208706456	153.4171989984	55.72511990125	208.75749051576	235.162637051203	0.833801784154599
Acinetobacter	42.0167343824797	164.8628398743	23.171967357	111.51990906547	195.666179713824	0.824505492437551
Unclassified_Lachnospiraceae	78.6115284150787	184.1602331881	23.71858606949	142.42393696302	231.481329377218	0.799339374660726
Dialister	68.6602985478987	263.5396854692	8.379102908824	155.78902208979	282.584557275295	0.790449391013566
Ruminococcus_1	0.180001102971555	83.59092271868	197.0547130294	122.28806507098	209.094257125012	0.777483099621045
Kocuria	197.763641133106	122.3668036064	19.19261794236	-8.306097187967	188.66660439997	0.756741908270458
Romboutsia	130.634682184437	164.0168556227	153.3431611865	143.41257601975	231.430610429757	0.750774091422522
Ureaplasma	67.499024406968	153.3666007047	87.90691463925	205.32411955172	229.845334364924	0.747735482774955
Williamsia	37.5848216717385	177.2742098748	50.2498323796	37.099014431266	176.359630036322	0.737822573421624
Unclassified_Neisseriaceae	25.2533130910985	156.6292803404	154.8091851314	69.337648129804	197.395967546511	0.733755379297805
Subdoligranulum	84.7917395041757	141.4607362611	34.23953202682	163.62874201122	188.406699449859	0.725845781854659
Sphingomonas	84.6942268380469	237.4607924666	-34.80167915058	56.607481807528	241.951655514482	0.714499360693817
Shewanella	58.3796268263295	171.7208981131	109.0882672108	180.19413605871	236.214893158712	0.704543677466618
Corynebacterium	39.0832703680836	57.36000790483	118.2640865133	119.28059311194	159.415413520753	0.695496405508134
Ruminococcaceae_UCG.014	66.4396878668754	173.416775956	34.4182612866	162.11309731557	212.716282569198	0.677375985031582
Frigoribacterium	45.2837791520733	189.0587249325	50.41145134985	51.88934740777	189.47806774723	0.670947665775729
Roseburia	17.4576183467414	148.9985241652	67.57898309863	160.02167008409	208.354377111744	0.670548525117996
Lactobacillus	98.1325620815931	159.832917458	14.29840297348	73.753816074314	176.588341777661	0.668880850169225
Muribacter	69.8601247877285	63.24601924125	135.6527478732	47.06178640617	138.14871424409	0.66391576483018
Corynebacterium_1	40.5398822351571	173.960551111	67.43238376877	68.955786874746	193.189585390252	0.65432287780836
Bacteroides	178.591411702507	96.00464467802	66.70340863472	46.376619973965	185.424882392922	0.651352615712282
Haematobacter	28.719532344518	87.07215974616	4.207688443224	164.38711203311	172.325571939707	0.620576488780259
Ruminiclostridium_9	124.751304190354	118.6655400645	63.32339499703	165.43308915266	225.661490634845	0.618420695574627
Faecalibaculum	128.426160725348	77.29125681789	62.44020567251	157.19112933779	191.549548988798	0.614851750887212
Salinicoccus	74.6184850317191	167.2050589413	96.29027041019	76.265654579379	167.23773218722	0.57776532798556
Ruminococcaceae_UCG.005	-9.23198309480277	206.685036708	0.012886306882	116.45014745333	210.523252732618	0.542261485369514
Pseudomonas	102.290582442922	116.0886559189	22.77956214151	89.49274948507	169.136466295446	0.535552776403439
Blautia	61.9635731043238	179.8366037279	14.42036333575	130.38044421388	198.025356869554	0.527579415910112
Curtobacterium	67.0717598453804	183.269241147	47.09868458171	35.934298475644	181.97919147701	0.524135406895104
Paracoccus	11.9585905429588	79.04616822088	3.525002841962	177.47503020979	182.269828079405	0.520221804795161
Anaerotruncus	98.1215933332864	18.9026297107	0.979891301889	93.260088897023	104.454568678045	0.480814780934197
Brevundimonas	101.507473517823	117.4329969544	-1.026079582701	15.458118186969	134.993600596875	0.468447221633797
Lachnospiraceae_UCG.006	135.549557464161	99.23031017104	24.03448833089	118.71902452381	189.908487094884	0.457196689023021
Micrococcus	5.21702926765925	107.1910129015	7.849734809627	135.69586404552	155.034839002714	0.447926972955465
Marmoricola	9.28910127704574	88.71409836552	15.4151758129	6.4469761684569	86.1345782679919	0.441515718646726
Unclassified_Peptostreptococcaceae	66.186669848993	97.73336211187	80.54106287474	65.66793535033	109.991504277556	0.434340195039788
Prevotellaceae_NK3B31_group	139.542471360075	24.83832191953	31.28221044896	103.95856628281	154.76470044595	0.431761338273291
Alkanindiges	102.077597171031	210.9698478922	68.53545466727	-1.939764149591	212.016011409508	0.420350291154687
Unclassified_Prevotellaceae	46.916566008178	106.2658718769	29.73035360879	175.03414290176	191.280789449267	0.418445035984043
Aeromicrobium	7.47572389987456	95.90864139619	72.76215784944	10.656329595126	97.4767652517278	0.404900999200976
Enhydrobacter	33.4461601350587	74.7649279692	15.82426170518	141.51869145249	158.123426286817	0.403862544512332
Ignavigranum	16.7787445248612	130.6469165255	71.61110257412	145.98779835799	175.511452186856	0.396245859635468

Ruminococcaceae_UCG.010	54.4656967323973	165.9507736425	10.29575202032	107.80674905278	169.972880652852	0.395475928058868
Parabacteroides	20.3580720979054	42.70030803473	4.433312113265	47.302020406521	59.7879896044456	0.386399443044428
Enterococcus	6.82525937363708	60.78801630643	-12.39101639892	95.36815834461	103.39090945881	0.384105408642045
Microbacterium	1.56344560312172	56.58039564609	62.25369704545	22.119680614558	66.1663845556894	0.377895556513144
Unclassified_Sphingomonadaceae	12.2106405167713	68.24436423377	109.468689712	55.846060364755	116.796521888246	0.375079461015915
Leucobacter	42.5837142024528	106.7886097843	72.30451134908	141.1783369557	168.439743148561	0.362375926405642
Leuconostoc	74.0653862107986	88.22128876436	128.8162218306	11.941524662384	144.975835700552	0.360882121070647
Rothia	15.9490183030118	2.411425168791	14.13014791915	124.60664159007	118.330589546707	0.352417973354033
Nocardioides	12.7923306320336	86.36342978898	37.41866451959	21.177299504229	88.2556453499392	0.351337494816548
Unclassified_Corynebacteriales	89.2977978538838	100.4128300454	-9.901123415987	81.546458740165	130.362971052998	0.334281872465495
Unclassified_Peptococcaceae	10.5057214871968	81.2574963818	36.13823046673	57.495398720736	104.153550288145	0.324201645746773
Ruminiclostridium_6	40.8302290051619	17.10023925	52.70732110141	2.8158606609311	47.9914505202986	0.319890913003755
Faecalibacterium	50.7885200664241	60.64954737662	35.38953522146	110.57716775994	120.715476143373	0.316416063342157
Anaerostipes	45.5309204459786	93.33968006632	64.11779633766	111.99755749556	138.135860354841	0.306188697314452
Unclassified_Bacteroidales	49.5706452257966	89.9235980004	25.22565724726	8.1822839704025	95.2894811370214	0.306155414544476
Candidatus_Saccharimonas	33.9947044403834	107.5848069953	27.15342230848	143.68998339308	158.254129351012	0.304369401689236
Rikenellaceae_RC9_gut_group	32.8662341026356	57.49817690996	35.18432033176	34.838805401008	80.1445801721183	0.293681845290378
Pelomonas	76.6418585484948	18.90842205416	7.355051679659	70.058403360992	84.312758491218	0.290577713486845
Nosocomiicoccus	30.5839595698914	-3.99534755786	21.61738978832	119.73377572185	101.201790029173	0.287118694196022
Deinococcus	31.6483273131202	65.21042778926	53.11651584114	104.1428661403	129.029144397651	0.283404399606089
Helicobacter	73.9043495557623	101.317019103	6.689670197997	22.955548322537	116.424780435503	0.282778240764124
Dolosigranulum	15.0965582830951	-37.6746533456	-12.95190317826	76.688488733558	15.4728470375881	0.279351408490268
Odoribacter	50.6373981920456	-1.35681953744	71.8626174517	78.257377661823	91.4882215464571	0.277400552242052
Unclassified_Rhizobiaceae	45.1379948149964	60.18157252201	106.4995254668	26.811636603215	112.192038058269	0.276402474030813
Ruminiclostridium_5	90.7513002525884	40.07735258468	-8.476878599566	63.177437860467	99.9946212769318	0.249555110534471
Janibacter	38.6886060675921	124.2447109766	24.76593878352	28.772497328837	124.780382526164	0.243215065440389
Prevotella_9	53.2943238160624	49.59567918831	48.07963333782	85.135234513571	110.198533366692	0.234230674122543
Turicibacter	43.1852481748624	64.25663956541	56.63192272632	58.581998196979	98.6386314915014	0.22910277968644
Atopostipes	33.0062799959733	70.77866432654	13.40726082689	106.64545289252	123.317264727793	0.226878819538319
Aureimonas	0.737233770776668	131.9517801741	61.42239027139	9.5774272118413	131.725104405868	0.225771199304059
GCA.900066575	84.9274769141589	18.27375841731	25.58104556758	77.453014955952	108.118729713902	0.219308978469569
Rikenella	26.7548118938495	71.88807069973	35.64002488664	144.24347079302	150.10696295587	0.215261398220806
Unclassified_Eggerthellaceae	34.9982326897488	33.5181278613	27.36605543324	120.66051700082	127.664560048221	0.212031867826314
Unclassified_Halomonadaceae	34.1422551289325	49.48773629526	43.89932154102	90.257894099594	109.323425381806	0.211366014138643
Unclassified_Desulfovibrionaceae	68.1545906246845	79.4963997336	69.9517719538	68.539946826684	121.657337337205	0.210830083964913
Facklamia	23.6088457172784	75.30970505526	51.82584352699	79.463527077147	115.359564390172	0.209711041025809
Unclassified_Betaproteobacteriales	34.8839020455856	18.31174828349	-14.66978930477	22.818823460218	28.1017491300042	0.206308487873365
Alloprevotella	51.3558205040484	69.21074593763	67.99863474165	68.398099975104	113.864616552883	0.203071882368249
Anaerococcus	38.612936430663	43.92335585773	20.81453267869	53.553685339433	70.846228787225	0.200563540911645
Unclassified_Firmicutes	23.229172908031	41.77208085274	-1.218761106346	44.197635558451	60.4395038878698	0.199404980494489
Actinomycetospora	38.4290606184939	67.59377459018	52.85326828425	37.639657809093	76.9744758043717	0.192327612002518
Skermanella	75.7672709502479	73.01364784548	-2.223355863778	6.4828878568799	93.4698735833805	0.191569207511199

ASF356	29.5043131208846	23.67387794102	9.2344156363	47.631861536433	55.7245017491332	0.191497384429447
Unclassified_Weeksellaceae	39.8616240808737	23.98177785972	6.77108759391	89.424083088127	92.4876983070353	0.186997223860407
Flavobacterium	19.8457542578334	77.94285466481	57.55695888742	26.373270484937	94.9456232160139	0.181754364943986
Unclassified_Devosiaceae	37.4617521999231	92.99282791135	48.73778703145	-0.426250311203	92.3395177406688	0.178614094161489
Sphingobacterium	33.6833008349007	101.2059157906	13.6234787434	19.918474940487	101.56240646074	0.177150158004584
Unclassified_Burkholderiaceae	10.5672257874746	-3.84098197921	31.16197356781	13.324246916405	17.2166171827797	0.176947419633908
Lysinibacillus	44.2890858085211	95.42539454415	3.766162984273	21.469464975495	95.4234938062783	0.176115680891921
Rhodococcus	64.8773281126695	26.17891315521	74.60017939727	28.775290042139	84.5826356409034	0.173842682958645
Jeotgalicoccus	30.3630317419354	14.35671383576	5.125812838934	46.234111306049	46.7944885106925	0.172752147787259
Unclassified_Nocardiaceae	33.9194810828212	111.5057570078	51.63733067913	52.226170061287	113.893015631974	0.168808184883364
Methylobacterium	26.1124341262805	68.99754078403	30.92116795424	27.011011714245	77.3290270973949	0.160614186799818
Unclassified_Rhodobacteraceae	3.62917401432503	19.85418638394	39.39940325215	24.528166610197	44.0995001834662	0.157082818569095
Unclassified_Chitinophagaceae	3.83096346614576	70.4540992874	44.77066820051	15.512974663618	72.9470705835233	0.154036186712613
Unclassified_Propionibacteriaceae	-3.55827989117931	75.13660572154	-1.87309077881	11.285596041232	74.023779068331	0.119413006058823
Variovorax	3.84482936586888	52.81549617151	1.527220037022	8.1910946358401	51.2321521631424	0.104374260925956
Lachnospiraceae_FCS020_group	38.8401039341469	18.4862918561	26.19194716449	59.706855949285	69.9118545358556	0.100862199430075
Comamonas	21.0372145209338	67.44025780767	1.640733616556	8.9228689511706	65.5032459041189	0.100273912818973
Unclassified_Rhizobiales	19.4109566306185	21.51525524905	39.51313573015	28.759677030655	46.3974330695943	0.097357320284474
Unclassified_Micrococcales	-24.8211938575132	106.8235109338	42.05341750037	2.0630349510127	106.760567157373	0.089179674801586
Sporosarcina	30.6158376581937	36.13640712889	29.46185928402	14.330527274291	52.6295972485253	0.087094373789469
Rathayibacter	24.5894536713288	83.81157608181	-2.843559958688	20.418202917271	83.9686011933223	0.078672548339226
Hymenobacter	-7.13695969205052	-2.42736275213	1.109641467798	8.5999785897972	2.07620119197625	0.077236101714577
Unclassified_Planococcaceae	20.4711594598848	57.04653927527	31.93094981176	40.695288876505	72.0531212507307	0.076663787323109
Promicromonospora	28.4054131846299	74.3882935545	34.95477075927	8.7660022751583	74.633754595426	0.075694909635215
Sphingobium	12.9211086232331	24.85138098763	-1.251024655648	37.389367441566	43.0327283621974	0.073322971280829
Enterorhabdus	32.9091394010676	23.8668870123	25.87245234084	6.8425955207387	39.5053770026734	0.07295617929178
Unclassified_Bacillales	25.0723338711182	41.87058183925	29.67347683578	14.14023349812	48.3625009746368	0.072777228720938
Lactococcus	21.6830547118576	41.4943186115	11.71377110896	7.872093745131	44.4944206392224	0.070269792530135
Unclassified_Alphaproteobacteria	21.0193375595777	50.65542499622	-7.504756201159	-3.503624105277	44.6025335877589	0.064754674525973
Stackebrandtia	27.4147751383079	69.18938248096	32.72925712575	-0.806196863879	69.2656129771616	0.06334816158451
Novosphingobium	21.5520486125573	65.67228129692	-0.524896509113	5.9603947581683	64.6521853632705	0.063276055145305
Frondihabitans	21.2975814338305	76.83168743342	30.88791459204	16.166432865953	76.9101804732372	0.061036024033456
Pseudogracilibacillus	21.6774387486726	61.5860596077	29.02319876551	18.922320707819	63.4925925889416	0.060506191621653
Chryseobacterium	-6.30056663046682	53.07344237226	-12.66576968922	-0.375607107257	47.2674558026825	0.060382818162234
Unclassified_Flavobacteriaceae	19.5320791686357	39.53524607648	26.5483087568	16.789598064652	45.0733470921747	0.05466581898945
Unclassified_Coriobacteriales	18.1232748043887	-14.0459912088	0.739358227397	23.452058838012	7.16433135514637	0.049255914278682
Pedobacter	16.4205817268232	19.03074463967	27.53165314753	0.4084103022316	24.5674727103888	0.04757335053177
Unclassified_Microbacteriaceae	1.5421238969264	11.14835134953	13.27585807765	3.0650256529948	14.4712419208781	0.039082663265948

Supplementary Table 3.3 Summary of indicator core *Staphylococcus* analysis in the standing and active communities. Only associations with adjusted p-value ≤ 0.05 are shown

	ASV	Group	Stat
**DNA Relative abundance**	ASV_2	Wild	0.547751443788229
	ASV_3	Wild	0.504657204001405
	ASV_1	C57BL/6J	0.468243650806376
	ASV_17	C57BL/6J	0.460108339544634
	ASV_20	Wild	0.445231418629768
	ASV_15	Wild	0.437110386646897
	ASV_4	Wild	0.425753339351648
	ASV_71	Wild	0.408970905956387
	ASV_27	Wild	0.396535980632989
	ASV_7	Wild	0.394863102316992
	ASV_118	C57BL/6J	0.39076035209259
	ASV_11	Wild	0.349562934136263
	ASV_107	HL-Lab	0.330369145003436
	ASV_155	Wild	0.303491952298545
	ASV_76	Wild	0.30108553140026
	ASV_132	Wild	0.286719436190406
	ASV_19	MPI-Lab	0.276298560517467
	ASV_74	Wild	0.254619219904143
	ASV_77	HL-Lab and MPI-Lab	0.240082649840776
	ASV_534	Wild	0.225199550871043
	ASV_186	Wild	0.221882647073218
**DNA Presence absence**	ASV_3	Wild	0.893523129978777
	ASV_4	Wild	0.885096433666378
	ASV_2	Wild	0.883213601053788
	ASV_11	Wild	0.820830167096418
	ASV_7	Wild	0.805586936516368
	ASV_15	Wild	0.754789974883551
	ASV_20	Wild	0.71557221032251
	ASV_1	Wild and C57BL/6J	0.643603858420696
	ASV_27	Wild	0.62623199028043
	ASV_71	Wild	0.594413107291815
	ASV_155	Wild	0.473280149985416
	ASV_17	C57BL/6J	0.472667031269418
	ASV_76	Wild	0.431510062441134
	ASV_69	Wild	0.424448651739461
	ASV_74	Wild	0.419535970147636
	ASV_269	Wild	0.387181727372914
	ASV_132	Wild	0.350823207722812
	ASV_222	Wild	0.333333333333333
	ASV_77	HL-Lab	0.329922246720153
	ASV_186	Wild	0.3192956325279
	ASV_167	Wild	0.308704716982372
	ASV_117	HL-Lab	0.303580893888878
	ASV_106	Wild	0.295723640385563
	ASV_107	HL-Lab	0.287754905882053
	ASV_118	C57BL/6J	0.284486446014944
	ASV_198	Wild	0.279576918477686
	ASV_534	Wild	0.260787414509885
	ASV_582	Wild	0.212132034355964
**RNA Relative abundance**	ASV_1	C57BL_6J	0.812963516970457
	ASV_17	C57BL_6J	0.746452462388221
	ASV_2	Wild	0.625811940013124
	ASV_3	Wild	0.613021137214643
	ASV_11	Wild	0.510285815016952
	ASV_4	Wild	0.467204883467697
	ASV_539	Wild	0.443581946043484
	ASV_2581	C57BL_6J	0.443532762572744
	ASV_20	Wild	0.443317125593993
	ASV_15	Wild	0.438153404056983
	ASV_27	Wild	0.407916786907463
	ASV_7	Wild	0.407576598995604
	ASV_71	Wild	0.403013134710732
	ASV_19	HL-Lab and MPI-Lab	0.397711663435338
	ASV_132	Wild	0.379563981309542
	ASV_155	Wild	0.357665376268558
	ASV_74	Wild	0.351984839051111
	ASV_582	MPI-Lab	0.344755218951092
	ASV_534	Wild	0.328286967141431
	ASV_76	Wild	0.323738690252657
	ASV_269	Wild	0.309529403317353
	ASV_106	Wild	0.301453261537107
	ASV_198	Wild	0.295851033558208
	ASV_222	Wild	0.25429673572549
	ASV_167	Wild	0.245794514408422
	ASV_186	Wild	0.243687240664362
**RNA Presence absence**	ASV_17	C57BL/6J	0.880262787827889
	ASV_2581	C57BL/6J	0.5
	ASV_118	C57BL/6J	0.461282311976277
	ASV_2	Wild	0.968740979637115
	ASV_3	Wild	0.936092952295236
	ASV_4	Wild	0.941206251865017
	ASV_7	Wild	0.883779048552786
	ASV_11	Wild	0.934392483486991
	ASV_15	Wild	0.749842594016148
	ASV_20	Wild	0.841576972292617
	ASV_27	Wild	0.810960947060743
	ASV_74	Wild	0.65649517552908
	ASV_76	Wild	0.652809572197546
	ASV_106	Wild	0.663835797544615
	ASV_132	Wild	0.588348405414552
	ASV_167	Wild	0.548464226846274
	ASV_198	Wild	0.556581178890961
	ASV_71	Wild	0.556366090118302
	ASV_155	Wild	0.592247020845304
	ASV_69	Wild	0.523634938088643
	ASV_539	Wild	0.519421464437234
	ASV_269	Wild	0.489246054790082
	ASV_222	Wild	0.480384461415261
	ASV_534	Wild	0.471404520791032
	ASV_79	Wild	0.295831658874859
	ASV_1	Wild and C57BL_6J	0.71293651798598
	ASV_186	Wild and C57BL_6J	0.33497884701857
	ASV_582	Wild and MPI-Lab	0.383265776410093
	ASV_19	C57BL/6J, HL-Lab and MPI-Lab	0.280502332635219

Supplementary Table 3.4 Imporatance components of the RandomForest framework based on DNA and RNA core *Staphylococcus* ASVs

ASVs DNA	C57BL/6J	HL-Lab	MPI-Lab	Wild	MeanDecreaseAccuracy	MeanDecreaseGini
ASV_2	614.893357567252	1357.14611147533	-326.28843552	735.5940079096	1537.49823554127	63.6295411252661
ASV_3	447.196946627932	682.117038613387	575.15644559	806.880707861	996.164933715021	39.8727455916415
ASV_4	542.824502737668	1595.00241447943	661.08133593	463.0463553809	1630.98638913911	38.2586740132666
ASV_19	52.9265272243006	480.49076416643	857.14468633	149.7605549621	782.053044346672	18.4709911385733
ASV_11	311.353528219232	529.881770718875	378.51820691	450.7860097589	649.352479036878	16.1007637752136
ASV_7	296.306704460416	510.419827482403	427.11941351	252.0120741241	613.763124189677	12.6173216592632
ASV_1	931.785972787312	1163.52028393958	298.15977818	169.8739358129	1333.75491048934	12.2531021902484
ASV_15	284.356658809056	826.261597282671	382.85384117	-45.200691189	829.007823763666	8.40549654789523
ASV_77	219.879273794101	485.807938821627	103.69995357	781.8291925871	904.56216011581	7.29428241062632
ASV_107	1.40036039120998	519.139905366914	149.33180753	362.2718634776	629.154467103996	5.87471575610007
ASV_249	1.26754025512107	644.617245726144	-171.35277732	-6.26294921217	513.325517323005	3.56992031595214
ASV_27	-122.023908640935	408.282220925726	205.06495381	32.8101420324	408.854231666936	3.39123118495029
ASV_20	144.020605525866	74.9177412371702	218.67958311	139.8380850505	168.307103607757	3.37972581102716
ASV_17	189.678280583684	399.66710488862	52.032374022	79.0772975008	375.381590057327	2.87764308134583
ASV_118	152.987579657274	57.6850350336457	17.751348015	101.1905845423	138.467440970008	2.50701728704241
ASV_117	-22.6675583978919	334.712261520647	336.97941524	261.7294781304	468.069934959499	2.45984867709444
ASV_79	-29.911735141533	298.466633241224	-293.71621959	52.73915742503	193.244295399212	1.56149974589635
ASV_76	153.011548079319	58.1585191385497	126.87786906	99.23153481326	136.137772975582	0.976041121001358
ASV_71	77.4366442821267	209.124427895313	97.603562805	-59.0583600059	202.111764803541	0.910089702677218
ASV_69	75.8267913046305	370.538161707944	113.71571962	-57.6634960172	369.177723121645	0.727994545796181
ASV_155	59.8811530275997	211.59181884263	78.632906235	-98.4877569767	178.544798374945	0.664353281670371
ASV_198	39.6867563206535	265.464866439762	67.487400654	-149.270962442	180.609524360805	0.541652101015531
ASV_186	21.6275338449092	52.6055089097531	57.858217213	-46.6335024587	8.50609561149964	0.416986127576503
ASV_74	51.6667593757179	193.389883878298	67.370176365	-8.07184562677	193.798240420423	0.310875858358661
ASV_269	15.3313347448919	29.1038774717986	20.569429718	-79.0575630272	-44.9998210945434	0.286244205479478
ASV_167	18.9439154445467	101.863308844418	25.681813251	6.76159361489	102.699699561726	0.076631249181797
ASV_222	10.864456082856	65.5829816764054	18.080644793	19.54687684425	70.9392968172697	0.070362140885578
ASV_132	12.4324150274829	63.4146207041267	20.141041312	0.43976267285	64.0789219475526	0.05431385298443
ASV_106	10.87540447149	57.3129297942294	16.423426601	24.02321146678	60.9489274835845	0.043686304626072
ASV_582	5.05229470836124	19.0537378203695	4.6269149453	5.934099881209	20.2748128807333	0.014814941640314
ASV_534	3.48539681161517	24.216076716534	5.5222615422	15.79941392964	29.3629837020861	0.010736570830182
ASV_539	2.30650667887662	21.5078637106989	3.5086761736	-4.06279626849	19.2961812162089	0.007677106643212
ASV_2581	0	0	0	0	0	0

ASVs RNA	C57BL_6J_RNA	Lab_HL_RNA	Lab_MPI_RNA	WildRNA	MeanDecreaseAccuracy	MeanDecreaseGini

ASV_2	590.892889656933	1235.95311515082	543.13972074	587.9724174595	1229.84448537501	41.4234096415531
ASV_4	476.884755842743	786.741339460707	428.73672702	371.6056452476	834.807104425846	29.1529447031734
ASV_11	467.627386623423	839.09805771256	432.09976996	301.1863785466	871.39958564089	26.6917694250799
ASV_3	432.595144185962	558.032384634409	390.80730407	318.8983116604	628.194471481039	24.7521605227994
ASV_7	384.855680586517	483.82913255189	383.05434354	277.0890349665	571.598495539136	16.1132034102164
ASV_1	812.146801825875	780.487689584544	191.48795917	88.37890536112	887.927304553492	10.8831425196254
ASV_20	277.327168689523	121.775341747045	253.23068872	81.65924565263	208.145976649765	10.5856389224229
ASV_15	224.097878253056	582.853348338282	-239.97748649	70.54209506161	594.661718502572	9.54050072778041
ASV_27	249.481884624235	396.334356360018	212.57953135	30.63329964902	398.630300182164	9.10000348285327
ASV_582	204.046324533892	1026.03592322846	874.75503782	85.03449400461	1183.20030574783	7.97718669043559
ASV_17	743.736876511964	497.345370274832	121.40067815	128.674061224	712.553678186485	6.98088981080873
ASV_19	99.621342145387	417.819515235839	97.842233459	338.9428674073	523.806948617281	6.23398552458117
ASV_71	-142.272497214529	473.111733475063	215.63227738	26.13095919202	473.993231201366	4.27345225262746
ASV_76	193.631507123667	377.661760885991	170.39860852	1.774071152192	380.624286966853	3.99581287549144
ASV_106	210.664550119048	475.691531178687	200.3595981	-143.689119862	475.001890648768	3.62570202057307
ASV_74	165.024720376486	293.226845847938	140.60113023	18.90856418947	295.991462928382	2.98359181379904
ASV_107	34.4769644037351	147.398028147936	-49.840686794	243.5627567293	253.888911296652	2.30536479336104
ASV_249	79.0014067242903	-214.129476777075	207.53828638	58.43097344013	-73.5183544948984	2.24246294689484
ASV_77	39.2959021428629	27.218391787468	46.535265472	238.6501053684	206.191470263461	1.67954890262498
ASV_117	-4.59438492322837	168.112797961108	142.4828212	103.811112498	210.741427665504	1.58673163767896
ASV_118	55.7636743255317	-34.5030807974109	171.81247846	58.8925553856	60.5263705661119	1.52626209236292
ASV_2581	163.28675418081	271.665053337885	38.960953867	38.75789092576	287.190019143021	1.36550428563116
ASV_132	113.802003264856	216.206788749121	97.091832556	-67.4503970271	216.009567403236	1.31184570759563
ASV_79	69.6350461322426	304.687568692838	-135.55539592	63.63235936867	293.855281767255	1.25864020647962
ASV_186	117.230837942078	540.412477192565	174.5273916	69.49032449808	547.156920970318	1.21965472299732
ASV_155	96.3871303582781	188.707984422002	84.529428302	-79.3949058329	187.972193550395	1.05853753136747
ASV_198	99.9818714619985	219.683582197114	88.849236726	27.33624403259	224.412162381813	1.0034856162282
ASV_167	61.7323526560314	116.064748819977	52.355550629	-47.1024724144	115.903981134835	0.462076708123731
ASV_69	74.3119578279655	162.280962454809	66.683749887	-63.9530939175	161.738088017489	0.435180674069621
ASV_269	55.0760037688367	127.093078583169	49.120721755	18.28389128786	128.623125519299	0.27335758246513
ASV_539	50.3957572262634	96.4082005631085	41.79595441	-44.806419577	96.0417775067419	0.267760091176176
ASV_534	35.4301003950931	75.7874792873196	30.552429551	-25.1704509973	75.7858000208743	0.123080795436097
ASV_222	32.2927797335905	77.7602495472209	27.501638829	-21.3936557588	77.7057072945519	0.11760586467084
