# Supplementary material for: Assessing similarities and disparities in the skin microbiota between wild and laboratory populations of house mice
Source: ISME J. 2020 Jun 9;14(10):2367–80. doi: 10.1038/s41396-020-0690-7 (PMC7490391; doi:10.1038/s41396-020-0690-7)
Supplement: Supplementary file 12 — Supplementary Table 4 [file 41396_2020_690_MOESM12_ESM.pdf]

Supplementary Table 4.1 Taxonomy of *Staphylococcus* and *Streptomyces* clone- and ASVs represennative sequences

Staphylococcus Clones	Sample	Group	Species match	Highest S_ab score
JJM0501_B2	JJM0501	Wild DNA	saprophyticus, xylosus	0.99
JJM0501_B4	JJM0501	Wild DNA	saprophyticus, xylosus	0.99
JJM0501_C9	JJM0501	Wild DNA	saprophyticus, xylosus	0.983
JJM0501_B6	JJM0501	Wild DNA	cohnii	0.959
JJM0501_A1	JJM0501	Wild DNA	equorum	0.984
JJM0501_C2	JJM0501	Wild DNA	arlettae	0.978
JJM0501_A1_2	JJM0501	Wild DNA	saprophyticus, xylosus	0.88
JJM0501_A4	JJM0501	Wild DNA	saprophyticus	0.955
JJM0501_A8	JJM0501	Wild DNA	saprophyticus, xylosus	0.903
JJM0501_A6	JJM0501	Wild DNA	saprophyticus, xylosus	0.887
JJM0501_B10	JJM0501	Wild DNA	saprophyticus, xylosus	0.879
JJM0501_A4_2	JJM0501	Wild DNA	saprophyticus	0.948
JJM0501_A7	JJM0501	Wild DNA	nepalensis	0.954
JJM0501_C10	JJM0501	Wild DNA	succinus	0.975
JJM0501_B10_B02	JJM0501	Wild DNA	succinus	0.982
JJM0501_B8	JJM0501	Wild DNA	saprophyticus, xylosus	0.946
JJM0501_C8	JJM0501	Wild DNA	succinus	0.966
JJM0501_A3	JJM0501	Wild DNA	lentus	0.976
JJM0501_C7	JJM0501	Wild DNA	lentus	0.889
JJM1203_F1	JJM1203	Wild DNA	cohnii, nepalensis	0.935
JJM1203_F8	JJM1203	Wild DNA	saprophyticus, xylosus	0.999
JJM1203_E7	JJM1203	Wild DNA	equorum	0.992
JJM1203_B8	JJM1203	Wild DNA	equorum	0.992
JJM1203_B6	JJM1203	Wild DNA	saprophyticus, xylosus	0.99
JJM1203_E3	JJM1203	Wild DNA	equorum	0.991
JJM1203_B7	JJM1203	Wild DNA	equorum	0.991
JJM1203_F3	JJM1203	Wild DNA	saprophyticus, xylosus	0.902
JJM1203_E4	JJM1203	Wild DNA	saprophyticus, xylosus	0.989
JJM1203_B9	JJM1203	Wild DNA	equorum	0.98
JJM1203_F9	JJM1203	Wild DNA	saprophyticus, xylosus	0.991
JJM1203_F5	JJM1203	Wild DNA	nepalensis	0.975
JJM1203_B3	JJM1203	Wild DNA	equorum	0.97
JJM1203_B2	JJM1203	Wild DNA	equorum	0.974
JJM1203_B1	JJM1203	Wild DNA	equorum	0.973
JJM1203_E8	JJM1203	Wild DNA	cohnii	0.983
JJM1203_F10	JJM1203	Wild DNA	cohnii, nepalensis	0.876
JJM1203_B5	JJM1203	Wild DNA	cohnii, nepalensis	0.891
JJM1203_E2	JJM1203	Wild DNA	cohnii, nepalensis	0.892
JJM1203_B4	JJM1203	Wild DNA	cohnii	0.973

JJM1203_E1	JJM1203	Wild DNA	cohnii	0.958
JJM1203_G1	JJM1203	Wild DNA	cohnii	0.945
JJM1203_F4	JJM1203	Wild DNA	succinus	0.991
JJM1203_F2	JJM1203	Wild DNA	cohnii	0.948
MJJ0112_C3	MJJ0112	Wild DNA	equorum	0.961
MJJ0112_C10_2	MJJ0112	Wild DNA	saprophyticus, xylosus	0.99
MJJ0112_C10	MJJ0112	Wild DNA	equorum	0.983
MJJ0112_C9_2	MJJ0112	Wild DNA	cohnii	0.962
MJJ0112_C7	MJJ0112	Wild DNA	equorum	0.991
MJJ0112_A4	MJJ0112	Wild DNA	equorum	0.991
MJJ0112_A3	MJJ0112	Wild DNA	equorum	0.986
MJJ0112_B1	MJJ0112	Wild DNA	equorum	0.97
MJJ0112_B3	MJJ0112	Wild DNA	equorum	0.983
MJJ0112_C6	MJJ0112	Wild DNA	equorum	0.979
MJJ0112_C1_2	MJJ0112	Wild DNA	equorum	0.983
MJJ0112_B7	MJJ0112	Wild DNA	equorum	0.983
MJJ0112_A6	MJJ0112	Wild DNA	equorum	0.975
MJJ0112_D1	MJJ0112	Wild DNA	equorum	0.983
MJJ0112_C8	MJJ0112	Wild DNA	equorum	0.982
MJJ0112_A1	MJJ0112	Wild DNA	saprophyticus, xylosus	0.97
MJJ0112_C2	MJJ0112	Wild DNA	equorum	0.975
MJJ0112_C1	MJJ0112	Wild DNA	cohnii, nepalensis	0.867
MJJ0112_B9	MJJ0112	Wild DNA	saprophyticus, xylosus	0.98
MJJ0112_B2	MJJ0112	Wild DNA	equorum	0.965
MJJ0112_A10	MJJ0112	Wild DNA	xylosus	0.982
MJJ0112_C9	MJJ0112	Wild DNA	equorum	0.991
MJJ0112_C8_2	MJJ0112	Wild DNA	equorum	0.974
MJJ0113_H2	MJJ0113	Wild DNA	saprophyticus, xylosus	0.982
MJJ0113_F5	MJJ0113	Wild DNA	equorum	0.983
MJJ0113_H6	MJJ0113	Wild DNA	saprophyticus, xylosus	0.988
MJJ0113_G4	MJJ0113	Wild DNA	saprophyticus, xylosus	0.988
MJJ0113_F1	MJJ0113	Wild DNA	equorum	0.983
MJJ0113_H10	MJJ0113	Wild DNA	saprophyticus, xylosus	0.999
MJJ0113_G6	MJJ0113	Wild DNA	equorum	0.975
MJJ0113_E1	MJJ0113	Wild DNA	saprophyticus, xylosus	0.999
MJJ0113_H8	MJJ0113	Wild DNA	saprophyticus, xylosus	0.907
MJJ0113_H1	MJJ0113	Wild DNA	saprophyticus, xylosus	0.899
MJJ0113_F6	MJJ0113	Wild DNA	saprophyticus, xylosus	0.914
MJJ0113_F2	MJJ0113	Wild DNA	equorum	0.97
MJJ0113_D1	MJJ0113	Wild DNA	saprophyticus, xylosus	0.995
MJJ0113_E4	MJJ0113	Wild DNA	saprophyticus, xylosus	0.893
MJJ0113_H9	MJJ0113	Wild DNA	saprophyticus, xylosus	0.888

MJJ0113_G5	MJJ0113	Wild DNA	gallinarum	0.98
MJJ0113_G2	MJJ0113	Wild DNA	saprophyticus, xylosus	0.9
MJJ0113_H7	MJJ0113	Wild DNA	saprophyticus, xylosus	0.972
MJJ0113_E3	MJJ0113	Wild DNA	saprophyticus, xylosus	0.995
MJJ0113_H4	MJJ0113	Wild DNA	saprophyticus, xylosus	0.883
MJJ0113_G3	MJJ0113	Wild DNA	saprophyticus, xylosus	0.996
MJJ0113_F7	MJJ0113	Wild DNA	saprophyticus, xylosus	0.876
MJJ0113_D2	MJJ0113	Wild DNA	gallinarum	0.954
MJJ0113_F4	MJJ0113	Wild DNA	saprophyticus, xylosus	0.899
MJJ0113_H5	MJJ0113	Wild DNA	sciuri	0.975
MJJ0113_E2	MJJ0113	Wild DNA	sciuri	0.965
MJJ0608_A5	MJJ0608	Wild DNA	equorum	0.989
MJJ0608_C6	MJJ0608	Wild DNA	saprophyticus, xylosus	0.999
MJJ0608_A9	MJJ0608	Wild DNA	saprophyticus, xylosus	0.997
MJJ0608_D4	MJJ0608	Wild DNA	xylosus	0.992
MJJ0608_C4	MJJ0608	Wild DNA	equorum	0.973
MJJ0608_D3	MJJ0608	Wild DNA	cohnii, nepalensis	0.889
MJJ0608_A8	MJJ0608	Wild DNA	saprophyticus, xylosus	0.987
MJJ0608_B5	MJJ0608	Wild DNA	saprophyticus, xylosus	0.963
MJJ0608_A2	MJJ0608	Wild DNA	saprophyticus, xylosus	0.878
MJJ0608_B8	MJJ0608	Wild DNA	cohnii, nepalensis	0.899
MJJ0608_A1	MJJ0608	Wild DNA	cohnii	0.983
MJJ0608_B9	MJJ0608	Wild DNA	cohnii	0.976
MJJ0608_B2	MJJ0608	Wild DNA	cohnii, nepalensis	0.975
MJJ0608_C5	MJJ0608	Wild DNA	saprophyticus, xylosus	0.996
MJJ0608_B7	MJJ0608	Wild DNA	cohnii	0.961
MJJ0608_C3	MJJ0608	Wild DNA	saprophyticus, xylosus	0.886
MJJ0608_B1	MJJ0608	Wild DNA	cohnii	0.965
MJJ0608_A4	MJJ0608	Wild DNA	cohnii	0.976
MJJ0608_A6	MJJ0608	Wild DNA	cohnii, nepalensis	0.867
MJJ0608_A7	MJJ0608	Wild DNA	cohnii, nepalensis	0.861
MJJ0608_B10	MJJ0608	Wild DNA	succinus	0.992
MJJ0608_A3	MJJ0608	Wild DNA	succinus	0.949
MN0207W_F1	MN0207	Wild DNA	xylosus	0.955
MN0207W_F9	MN0207	Wild DNA	saprophyticus, xylosus	0.897
MN0207W_F6	MN0207	Wild DNA	saprophyticus, xylosus	0.889
MN0207W_E3	MN0207	Wild DNA	saprophyticus, xylosus	0.894
MN0207W_E2	MN0207	Wild DNA	saprophyticus, xylosus	0.906
MN0207W_F2	MN0207	Wild DNA	saprophyticus, xylosus	0.882
MN0207W_F5	MN0207	Wild DNA	saprophyticus, xylosus	0.88
MN0207W_E6	MN0207	Wild DNA	saprophyticus, xylosus	0.89
MN0207W_D1	MN0207	Wild DNA	saprophyticus, xylosus	0.989

MN0207W_C4	MN0207	Wild DNA	saprophyticus, xylosus	0.878
MN0207W_B5	MN0207	Wild DNA	saprophyticus, xylosus	0.888
MN0207W_A2	MN0207	Wild DNA	xylosus	0.982
MN0207W_F10	MN0207	Wild DNA	xylosus	0.955
MN0207W_E1	MN0207	Wild DNA	succinus	0.887
MN0207W_E10	MN0207	Wild DNA	succinus	0.98
MN0207W_E5	MN0207	Wild DNA	succinus	0.975
MN0207W_D7	MN0207	Wild DNA	lentus	0.991
MN0207W_A7	MN0207	Wild DNA	lentus	0.974
MN24_E6	MN2401	Wild DNA	arlettae	0.983
MN2401_F2	MN2401	Wild DNA	saprophyticus, xylosus	0.995
MN2401_F4	MN2401	Wild DNA	saprophyticus, xylosus	0.967
MN2401_3G	MN2401	Wild DNA	equorum	0.985
MN2401_G7	MN2401	Wild DNA	saprophyticus, xylosus	0.95
MN2401_F3	MN2401	Wild DNA	saprophyticus, xylosus	0.931
MN2401_H4	MN2401	Wild DNA	saprophyticus, xylosus	0.962
MN2401_C7	MN2401	Wild DNA	arlettae	0.986
MN2401_H5	MN2401	Wild DNA	saprophyticus, xylosus	0.885
MN2401_5G	MN2401	Wild DNA	equorum	0.957
MN2401_G9	MN2401	Wild DNA	succinus	0.993
MN2401_G8	MN2401	Wild DNA	arlettae	0.947
MN2401_E6	MN2401	Wild DNA	arlettae	0.983
MN2401_1E	MN2401	Wild DNA	arlettae	0.98
MN2401_H2	MN2401	Wild DNA	succinus	0.988
MN2401_4G	MN2401	Wild DNA	saprophyticus, xylosus	0.895
MN2401_H3	MN2401	Wild DNA	succinus	0.974
MN2401_E7	MN2401	Wild DNA	succinus	0.985
MN2401_1G	MN2401	Wild DNA	succinus	0.989
MN2401_5E	MN2401	Wild DNA	succinus	0.961
MN2401_2E	MN2401	Wild DNA	succinus	0.98
MN2401_E9	MN2401	Wild DNA	succinus	0.959
MN2401_E8	MN2401	Wild DNA	saprophyticus, xylosus	0.992
MN2401_2G	MN2401	Wild DNA	saprophyticus, xylosus	0.841
MN2401_3E	MN2401	Wild DNA	saprophyticus, xylosus	0.896
MN2614_G9	MN2614	Wild DNA	equorum	0.992
MN2614_E9	MN2614	Wild DNA	equorum	0.992
MN2614_E5	MN2614	Wild DNA	equorum	0.992
MN2614_G5	MN2614	Wild DNA	equorum	0.983
MN2614_E2	MN2614	Wild DNA	saprophyticus, xylosus	0.988
MN2614_E8	MN2614	Wild DNA	saprophyticus, xylosus	0.988
MN2614_H2	MN2614	Wild DNA	equorum	0.996
MN2614_F6	MN2614	Wild DNA	equorum	0.98

MN2614_D9	MN2614	Wild DNA	saprophyticus, xylosus	0.983
MN2614_H1	MN2614	Wild DNA	equorum	0.974
MN2614_F10	MN2614	Wild DNA	xylosus	0.999
MN2614_F8	MN2614	Wild DNA	equorum	0.982
MN2614_F1	MN2614	Wild DNA	xylosus	0.999
MN2614_D7	MN2614	Wild DNA	equorum	0.982
MN2614_E4	MN2614	Wild DNA	saprophyticus, xylosus	0.967
MN2614_D3	MN2614	Wild DNA	equorum	0.973
MN2614_G7	MN2614	Wild DNA	equorum	0.969
MN2614_E6	MN2614	Wild DNA	equorum	0.969
MN2614_D10	MN2614	Wild DNA	saprophyticus, xylosus	0.907
MN2614_D8	MN2614	Wild DNA	equorum	0.974
MN2614_D5	MN2614	Wild DNA	succinus	0.992
MN2614_G3	MN2614	Wild DNA	succinus	0.986
MN2614_D6	MN2614	Wild DNA	succinus	0.972
MN2614_F3	MN2614	Wild DNA	succinus	0.954
MN2614_E7	MN2614	Wild DNA	lentus	0.948
MN2901_E9	MN2901	Wild DNA	saprophyticus, xylosus	0.988
MN2901_G7	MN2901	Wild DNA	saprophyticus, xylosus	0.988
MN2901_H9	MN2901	Wild DNA	saprophyticus, xylosus	0.999
MN2901_G8	MN2901	Wild DNA	saprophyticus, xylosus	0.989
MN2901_E6	MN2901	Wild DNA	equorum	0.983
MN2901_B8	MN2901	Wild DNA	equorum	0.978
MN2901_B5	MN2901	Wild DNA	equorum	0.982
MN2901_B3	MN2901	Wild DNA	saprophyticus, xylosus	0.988
MN2901_H1	MN2901	Wild DNA	saprophyticus, xylosus	0.902
MN2901_B6	MN2901	Wild DNA	equorum	0.973
MN2901_H10	MN2901	Wild DNA	saprophyticus, xylosus	0.922
MN2901_E4	MN2901	Wild DNA	saprophyticus, xylosus	0.905
MN2901_G9	MN2901	Wild DNA	saprophyticus, xylosus	0.957
MN2901_F8	MN2901	Wild DNA	saprophyticus, xylosus	895
MN2901_F1	MN2901	Wild DNA	saprophyticus, xylosus	0.891
MN2901_B10	MN2901	Wild DNA	saprophyticus, xylosus	0.862
MN2901_H2	MN2901	Wild DNA	cohnii	0.957
MN2901_E2	MN2901	Wild DNA	succinus	0.957
MN2901_B7	MN2901	Wild DNA	sciuri	0.963
MN2901_G10	MN2901	Wild DNA	sciuri	0.955
MN3211_C1	MN3211	Wild DNA	equorum	0.985
MN3211_B1	MN3211	Wild DNA	saprophyticus, xylosus	1
MN3211_D10	MN3211	Wild DNA	equorum	0.987
MN3211_D4	MN3211	Wild DNA	saprophyticus, xylosus	0.9
MN3211_D2	MN3211	Wild DNA	saprophyticus, xylosus	0.925

MN3211_A9	MN3211	Wild DNA	saprophyticus, xylosus	0.911
MN3211_A6	MN3211	Wild DNA	equorum	0.973
MN3211_A3	MN3211	Wild DNA	saprophyticus, xylosus	0.919
MN3211_C5	MN3211	Wild DNA	saprophyticus, xylosus	0.93
MN3211_C6	MN3211	Wild DNA	cohnii , napelensis	0.89
MN3211_C3	MN3211	Wild DNA	nepalensis, cohnii	0.892
MN3211_A5	MN3211	Wild DNA	cohnii	0.983
MN3211_C8	MN3211	Wild DNA	saprophyticus, xylosus	0.906
MN3211_C7	MN3211	Wild DNA	equorum	0.966
MN3211_B4	MN3211	Wild DNA	xylosus	0.982
MN3211_A2	MN3211	Wild DNA	saprophyticus, xylosus	0.863
MN3211_B9	MN3211	Wild DNA	saprophyticus, xylosus	0.886
MN3211_A7	MN3211	Wild DNA	saprophyticus, cohnii	0.944
MN3211_D5	MN3211	Wild DNA	equorum	0.975
MN3211_A1	MN3211	Wild DNA	equorum	0.889
MN3211_B3	MN3211	Wild DNA	sciuri	0.966
9193_A05_E05_G9	9193	HL-Lab DNA	hominis	0.896
9193_A04_E04_G8	9193	HL-Lab DNA	hominis subsp	0.907
9215_C04_G04_C6	9215	HL-Lab DNA	epidermidis	0.889
9215_C02_G02_C2	9215	HL-Lab DNA	epidermidis	0.895
9215_B02_F02_A2	9215	HL-Lab DNA	epidermidis	0.895
9215_B06_B06_A7	9215	HL-Lab DNA	epidermidis	0.902
9215_B03_E03_A4	9215	HL-Lab DNA	epidermidis	0.896
9215_C03_G03_C3	9215	HL-Lab DNA	pasteuri	0.906
9215_B04_F04_A5	9215	HL-Lab DNA	epidermidis	0.9
9215_C06_G06_C8	9215	HL-Lab DNA	epidermidis	0.906
9215_D02_H02_D2	9215	HL-Lab DNA	epidermidis	0.905
9215_B09_F09_A10	9215	HL-Lab DNA	epidermidis	0.906
9215_B01_F01_A1	9215	HL-Lab DNA	pasteuri	0.91
9215_D06_H06_D06	9215	HL-Lab DNA	epidermidis	0.91
9215_D01_H01_D1	9215	HL-Lab DNA	epidermidis	0.911
9215_D03_H03_D3	9215	HL-Lab DNA	pasteuri	0.912
9215_B05_F05_A6	9215	HL-Lab DNA	pasteuri	0.912
9215_C1_G1_C1	9215	HL-Lab DNA	pasteuri	0.913
9215_C05_G05_C7	9215	HL-Lab DNA	epidermidis	0.913
9215_D04_H04_D4	9215	HL-Lab DNA	pasteuri	0.918
9215_D05_H05_D5	9215	HL-Lab DNA	epidermidis	0.915
9215_D9_D01_D02	9215	HL-Lab DNA	epidermidis	0.919
9215_A8_E01_E02	9215	HL-Lab DNA	pasteuri	0.931
9215_C9_C01_C02	9215	HL-Lab DNA	epidermidis	0.925
9215_A8_B01_B02	9215	HL-Lab DNA	epidermidis	0.945

Supplementary Table 4.2 Taxonomy of core *Staphylococcus* ASVs represennative sequences

***Staphylococcus* core ASV**	**Matched Clone**	**Score**	**Taxonomy**
ASV_1	MJJ0113_F7	100	saprophyticus, xylosus
ASV_1	MJJ0113_G2	100	saprophyticus, xylosus
ASV_1	MJJ0113_H1	100	saprophyticus, xylosus
ASV_1	MJJ0113_H4	100	saprophyticus, xylosus
ASV_1	MJJ0113_H8	100	saprophyticus, xylosus
ASV_1	MJJ0113_H9	100	saprophyticus, xylosus
ASV_1	MN0207W_B5	100	saprophyticus, xylosus
ASV_1	MN0207W_D1	100	saprophyticus, xylosus
ASV_1	MN0207W_E2	100	saprophyticus, xylosus
ASV_1	MN0207W_E3	100	saprophyticus, xylosus
ASV_1	MN0207W_F2	100	saprophyticus, xylosus
ASV_1	MN0207W_F6	100	saprophyticus, xylosus
ASV_1	MN0207W_F9	100	saprophyticus, xylosus
ASV_1	MN2901_E4	100	saprophyticus, xylosus
ASV_1	MN2901_F1	100	saprophyticus, xylosus
ASV_1	MN2901_F8	100	saprophyticus, xylosus
ASV_1	MN2901_H1	100	saprophyticus, xylosus
ASV_1	MN2901_H10	100	saprophyticus, xylosus
ASV_1	MN3211_A3	100	saprophyticus, xylosus
ASV_1	MN3211_A9	100	saprophyticus, xylosus
ASV_1	MN3211_B1	100	saprophyticus, xylosus
ASV_1	MN3211_C8	100	saprophyticus, xylosus
ASV_1	MN3211_D2	100	saprophyticus, xylosus
ASV_2	JJM0501_A1	99.524	equorum
ASV_2	MJJ0112_A4	99.524	equorum
ASV_2	MJJ0112_B2	99.524	equorum
ASV_2	MJJ0112_B7	99.524	equorum
ASV_2	MJJ0112_C7	99.524	equorum
ASV_2	MJJ0112_C9	99.524	equorum
ASV_2	MJJ0113_G6	99.524	equorum
ASV_2	MN2614_G7	99.524	equorum
ASV_2	MN2614_G9	99.524	equorum
ASV_2	MN2614_H1	99.524	equorum
ASV_2	MN2901_B5	99.524	equorum
ASV_2	MN2901_E6	99.524	equorum
ASV_2	MN3211_A7	99.524	saprophyticus, cohnii
ASV_2	MN3211_C1	99.524	equorum
ASV_3	JJM0501_B2	100	saprophyticus, xylosus
ASV_3	JJM0501_B4	100	saprophyticus, xylosus

ASV_3	MJJ0112_A1	100	saprophyticus, xylosus
ASV_3	MJJ0113_H2	100	saprophyticus, xylosus
ASV_3	MJJ0113_H7	100	saprophyticus, xylosus
ASV_3	MN0207W_A2	100	xylosus
ASV_3	MN2614_E2	100	saprophyticus, xylosus
ASV_3	MN2614_E8	100	saprophyticus, xylosus
ASV_3	MN2614_F10	100	xylosus
ASV_3	MN2901_G7	100	saprophyticus, xylosus
ASV_4	JJM0501_A6	100	saprophyticus, xylosus
ASV_4	JJM0501_A8	100	saprophyticus, xylosus
ASV_4	JJM0501_C9	100	saprophyticus, xylosus
ASV_4	MJJ0112_C1	100	cohnii, nepalensis
ASV_4	MJJ0113_D1	100	saprophyticus, xylosus
ASV_4	MJJ0113_E1	100	saprophyticus, xylosus
ASV_4	MJJ0113_E4	100	saprophyticus, xylosus
ASV_4	MJJ0113_F6	100	saprophyticus, xylosus
ASV_4	MJJ0113_F7	100	saprophyticus, xylosus
ASV_4	MJJ0113_G2	100	saprophyticus, xylosus
ASV_4	MJJ0113_H1	100	saprophyticus, xylosus
ASV_4	MJJ0113_H4	100	saprophyticus, xylosus
ASV_4	MJJ0113_H8	100	saprophyticus, xylosus
ASV_4	MJJ0113_H9	100	saprophyticus, xylosus
ASV_4	MN0207W_B5	100	saprophyticus, xylosus
ASV_4	MN0207W_D1	100	saprophyticus, xylosus
ASV_4	MN0207W_E2	100	saprophyticus, xylosus
ASV_4	MN0207W_E3	100	saprophyticus, xylosus
ASV_4	MN0207W_F2	100	saprophyticus, xylosus
ASV_4	MN0207W_F6	100	saprophyticus, xylosus
ASV_4	MN0207W_F9	100	saprophyticus, xylosus
ASV_4	MN2901_E4	100	saprophyticus, xylosus
ASV_4	MN2901_F1	100	saprophyticus, xylosus
ASV_4	MN2901_F8	100	saprophyticus, xylosus
ASV_4	MN2901_H1	100	saprophyticus, xylosus
ASV_4	MN2901_H10	100	saprophyticus, xylosus
ASV_4	MN3211_A3	100	saprophyticus, xylosus
ASV_4	MN3211_A9	100	saprophyticus, xylosus
ASV_4	MN3211_B1	100	saprophyticus, xylosus
ASV_4	MN3211_C8	100	saprophyticus, xylosus
ASV_4	MN3211_D2	100	saprophyticus, xylosus
ASV_7	MN0207W_E10	99.524	succinus
ASV_7	MN2401_1G	99.524	succinus
ASV_7	MN2614_G3	99.524	succinus

ASV_11	JJM0501_A1	99.524	equorum
ASV_11	MJJ0112_A4	99.524	equorum
ASV_11	MJJ0112_B2	99.524	equorum
ASV_11	MJJ0112_B7	99.524	equorum
ASV_11	MJJ0112_C7	99.524	equorum
ASV_11	MJJ0112_C9	99.524	equorum
ASV_11	MJJ0113_G6	99.524	equorum
ASV_11	MN2614_G7	99.524	equorum
ASV_11	MN2614_G9	99.524	equorum
ASV_11	MN2614_H1	99.524	equorum
ASV_11	MN2901_B5	99.524	equorum
ASV_11	MN2901_E6	99.524	equorum
ASV_11	MN3211_A7	99.524	saprophyticus, cohnii
ASV_11	MN3211_C1	99.524	equorum
ASV_15	MJJ0608_B1	98.578	cohnii
ASV_17	JJM0501_A6	99.524	saprophyticus, xylosus
ASV_17	JJM0501_A8	99.524	saprophyticus, xylosus
ASV_17	JJM0501_C9	99.524	saprophyticus, xylosus
ASV_17	MJJ0112_C1	99.524	cohnii, nepalensis
ASV_17	MJJ0113_D1	99.524	saprophyticus, xylosus
ASV_17	MJJ0113_E1	99.524	saprophyticus, xylosus
ASV_17	MJJ0113_E4	99.524	saprophyticus, xylosus
ASV_17	MJJ0113_F6	99.524	saprophyticus, xylosus
ASV_17	MJJ0113_F7	99.524	saprophyticus, xylosus
ASV_17	MJJ0113_G2	99.524	saprophyticus, xylosus
ASV_17	MJJ0113_H1	99.524	saprophyticus, xylosus
ASV_17	MJJ0113_H4	99.524	saprophyticus, xylosus
ASV_17	MJJ0113_H8	99.524	saprophyticus, xylosus
ASV_17	MJJ0113_H9	99.524	saprophyticus, xylosus
ASV_17	MN0207W_B5	99.524	saprophyticus, xylosus
ASV_17	MN0207W_D1	99.524	saprophyticus, xylosus
ASV_17	MN0207W_E2	99.524	saprophyticus, xylosus
ASV_17	MN0207W_E3	99.524	saprophyticus, xylosus
ASV_17	MN0207W_F2	99.524	saprophyticus, xylosus
ASV_17	MN0207W_F6	99.524	saprophyticus, xylosus
ASV_17	MN0207W_F9	99.524	saprophyticus, xylosus
ASV_17	MN2901_E4	99.524	saprophyticus, xylosus
ASV_17	MN2901_F1	99.524	saprophyticus, xylosus
ASV_17	MN2901_F8	99.524	saprophyticus, xylosus
ASV_17	MN2901_H1	99.524	saprophyticus, xylosus
ASV_17	MN2901_H10	99.524	saprophyticus, xylosus
ASV_17	MN3211_A3	99.524	saprophyticus, xylosus

ASV_17	MN3211_A9	99.524	saprophyticus, xylosus
ASV_17	MN3211_B1	99.524	saprophyticus, xylosus
ASV_17	MN3211_C8	99.524	saprophyticus, xylosus
ASV_17	MN3211_D2	99.524	saprophyticus, xylosus
ASV_19	9215_A8_B01_B02	99.052	epidermidis
ASV_19	9215_B02_F02_A2	99.052	epidermidis
ASV_19	9215_B09_F09_A10	99.052	epidermidis
ASV_19	9215_C02_G02_C2	99.052	epidermidis
ASV_19	9215_C04_G04_C6	99.052	epidermidis
ASV_19	9215_C05_G05_C7	99.052	epidermidis
ASV_19	9215_C06_G06_C8	99.052	epidermidis
ASV_19	9215_D01_H01_D1	99.052	epidermidis
ASV_19	9215_D02_H02_D2	99.052	epidermidis
ASV_19	9215_D05_H05_D5	99.052	epidermidis
ASV_19	9215_D9_D01_D02	99.052	epidermidis
ASV_20	MJJ0113_G4	100	saprophyticus, xylosus
ASV_20	MJJ0113_H6	100	saprophyticus, xylosus
ASV_20	MJJ0608_A9	100	saprophyticus, xylosus
ASV_20	MN2901_B3	100	saprophyticus, xylosus
ASV_20	MN2901_E9	100	saprophyticus, xylosus
ASV_27	MJJ0608_B8	98.578	cohnii, nepalensis
ASV_69	MN0207W_E1	96.894	succinus
ASV_71	MN0207W_D7	99.057	lentus
ASV_71	MN2614_E7	99.057	lentus
ASV_74	MJJ0608_B9	98.578	cohnii
ASV_76	MN0207W_E10	99.524	succinus
ASV_76	MN2401_1G	99.524	succinus
ASV_76	MN2614_G3	99.524	succinus
ASV_77	9193_A04_E04_G8	98.578	hominis
ASV_77	9193_A05_E05_G9	98.578	hominis
ASV_79	MN0207W_E1	98.125	succinus
ASV_106	MJJ0112_A3	99.524	equorum
ASV_106	MJJ0112_C2	99.524	equorum
ASV_106	MJJ0608_C4	99.524	equorum
ASV_107	9193_A04_E04_G8	99.052	hominis
ASV_107	9193_A05_E05_G9	99.052	hominis
ASV_117	9215_B01_F01_A1	96.698	pasteuri
ASV_117	9215_B05_F05_A6	96.698	pasteuri
ASV_117	9215_C1_G1_C1	96.698	pasteuri
ASV_117	9215_C03_G03_C3	96.698	pasteuri
ASV_117	9215_D03_H03_D3	96.698	pasteuri
ASV_117	9215_D04_H04_D4	96.698	pasteuri

ASV_118	9215_A8_B01_B02	97.156	epidermidis
ASV_118	9215_B02_F02_A2	97.156	epidermidis
ASV_118	9215_B09_F09_A10	97.156	epidermidis
ASV_118	9215_C02_G02_C2	97.156	epidermidis
ASV_118	9215_C04_G04_C6	97.156	epidermidis
ASV_118	9215_C05_G05_C7	97.156	epidermidis
ASV_118	9215_C06_G06_C8	97.156	epidermidis
ASV_118	9215_D01_H01_D1	97.156	epidermidis
ASV_118	9215_D02_H02_D2	97.156	epidermidis
ASV_118	9215_D05_H05_D5	97.156	epidermidis
ASV_118	9215_D9_D01_D02	97.156	epidermidis
ASV_132	JJM0501_A6	100	saprophyticus, xylosus
ASV_132	JJM0501_A8	100	saprophyticus, xylosus
ASV_132	JJM0501_C9	100	saprophyticus, xylosus
ASV_132	MJJ0112_C1	100	cohnii, nepalensis
ASV_132	MJJ0113_D1	100	saprophyticus, xylosus
ASV_132	MJJ0113_E1	100	saprophyticus, xylosus
ASV_132	MJJ0113_E4	100	saprophyticus, xylosus
ASV_132	MJJ0113_F6	100	saprophyticus, xylosus
ASV_132	MJJ0113_F7	100	saprophyticus, xylosus
ASV_132	MJJ0113_G2	100	saprophyticus, xylosus
ASV_132	MJJ0113_H1	100	saprophyticus, xylosus
ASV_132	MJJ0113_H4	100	saprophyticus, xylosus
ASV_132	MJJ0113_H8	100	saprophyticus, xylosus
ASV_132	MJJ0113_H9	100	saprophyticus, xylosus
ASV_132	MN0207W_B5	100	saprophyticus, xylosus
ASV_132	MN0207W_D1	100	saprophyticus, xylosus
ASV_132	MN0207W_E2	100	saprophyticus, xylosus
ASV_132	MN0207W_E3	100	saprophyticus, xylosus
ASV_132	MN0207W_F2	100	saprophyticus, xylosus
ASV_132	MN0207W_F6	100	saprophyticus, xylosus
ASV_132	MN0207W_F9	100	saprophyticus, xylosus
ASV_132	MN2901_E4	100	saprophyticus, xylosus
ASV_132	MN2901_F1	100	saprophyticus, xylosus
ASV_132	MN2901_F8	100	saprophyticus, xylosus
ASV_132	MN2901_H1	100	saprophyticus, xylosus
ASV_132	MN2901_H10	100	saprophyticus, xylosus
ASV_132	MN3211_A3	100	saprophyticus, xylosus
ASV_132	MN3211_A9	100	saprophyticus, xylosus
ASV_132	MN3211_B1	100	saprophyticus, xylosus
ASV_132	MN3211_C8	100	saprophyticus, xylosus
ASV_132	MN3211_D2	100	saprophyticus, xylosus

ASV_155	MJJ0608_B8	98.104	cohnii, nepalensis
ASV_167	MJJ0112_A3	99.524	equorum
ASV_167	MJJ0112_C2	99.524	equorum
ASV_167	MJJ0608_C4	99.524	equorum
ASV_186	MN2401_1E	98.578	arlettae
ASV_186	MN2401_E6	98.578	arlettae
ASV_198	MN0207W_E10	99.048	succinus
ASV_198	MN2401_1G	99.048	succinus
ASV_198	MN2614_G3	99.048	succinus
ASV_222	JJM1203_G1	98.104	cohnii
ASV_222	MJJ0608_B1	98.104	cohnii
ASV_249	9193_A04_E04_G8	99.052	hominis
ASV_249	9193_A05_E05_G9	99.052	hominis
ASV_269	MJJ0113_G5	98.578	gallinarum
ASV_534	MJJ0113_G4	99.524	saprophyticus, xylosus
ASV_534	MJJ0113_H6	99.524	saprophyticus, xylosus
ASV_534	MJJ0608_A9	99.524	saprophyticus, xylosus
ASV_534	MN2901_B3	99.524	saprophyticus, xylosus
ASV_534	MN2901_E9	99.524	saprophyticus, xylosus
ASV_539	MN3211_C7	99.524	equorum
ASV_582	MJJ0113_H5	99.057	sciuri
ASV_2581	9215_A8_B01_B02	97.156	epidermidis
ASV_2581	9215_B02_F02_A2	97.156	epidermidis
ASV_2581	9215_B09_F09_A10	97.156	epidermidis
ASV_2581	9215_C02_G02_C2	97.156	epidermidis
ASV_2581	9215_C04_G04_C6	97.156	epidermidis
ASV_2581	9215_C05_G05_C7	97.156	epidermidis
ASV_2581	9215_C06_G06_C8	97.156	epidermidis
ASV_2581	9215_D01_H01_D1	97.156	epidermidis
ASV_2581	9215_D02_H02_D2	97.156	epidermidis
ASV_2581	9215_D05_H05_D5	97.156	epidermidis
ASV_2581	9215_D9_D01_D02	97.156	epidermidis

Supplementary Table 4.3 Taxonomy of *Staphylococcus* and *Streptomyces* clone- and ASVs represennative sequences

Streptomyces Clones	Sample	Group	Species match, and S_ab score	Highest S_ab score
C10_MJJ1004_C07	MJJ1004	Wild DNA	albidoflavus (0.842)	0.842
A5_MJJ1002_A05	MJJ1002	Wild DNA	somaliensis (0.869)	0.869
H8_MJJ0117_H06	MJJ0117	Wild DNA	qinglanensis (0.809)	0.809
H1__MJJ0117_H01	MJJ0117	Wild DNA	sulphureus (0.826), xiaopingdaonensis (0.819)	0.826
E1_MJJ0102	MJJ0102	Wild DNA	rubrus (0.819), chungwhensis (0.811)	0.819
G4_MJJ0117_G04	MJJ0117	Wild DNA	qinglanensis (0.771)	0.771
C3_MJJ1004_C02	MJJ1004	Wild DNA	violascens (0.895), albidoflavus (0.893)	0.895
D2_MT0113	MT0113	Wild DNA	qinglanensis (0.811), panacagri (0.809)	0.811
C2_MJJ1004_C01	MJJ1004	Wild DNA	cacaoi (0.839), qinglanensis (0.836)	0.839
A9_MJJ1002_A09	MJJ1002	Wild DNA	chrestomyceticus (0.846), platensis (0.84)	0.846
E3_MT1501_E03	MT1501	Wild DNA	qinglanensis (0.755), armeniacus (0.752)	0.755
A1_MJJ1002_A01	MJJ1002	Wild DNA	bohaiensis (0.818)	0.818
E2_MT1501_E02	MT1501	Wild DNA	ambofaciens (0.873)	0.873
E5_MT1501_E05	MT1501	Wild DNA	synnematoformans (0.785), aculeolatus (0.783)	0.785
A3_MJJ1002_A03	MJJ1002	Wild DNA	cavourensis (0.871), flavovirens (0.869)	0.871
E2_MJJ0102	MJJ0102	Wild DNA	albidoflavus (0.814)	0.814
H9_MJJ0117_H07	MJJ0117	Wild DNA	qinglanensis (0.758)	0.758
E12_MT1501_E10	MT1501	Wild DNA	synnematoformans (0.828)	0.828
G9_MJJ0117_G11	MJJ0117	Wild DNA	qinglanensis (0.803), nanshensis (0.793)	0.803
G2_MT3509	MT3509	Wild DNA	qinglanensis (0.801), nanshensis (0.79)	0.801
B5_MJJ1002_B05	MJJ1002	Wild DNA	albidoflavus (0.865)	0.865
G8_MJJ0117_G08	MJJ0117	Wild DNA	albidoflavus (0.902)	0.902
B10_MJJ1002_B09	MJJ1002	Wild DNA	carnosus (0.858), pactum (0.858), olivaceus (0.858), litmocidini (0.858)	0.858
G11_MJJ0117_G11	MJJ0117	Wild DNA	albidoflavus (0.877)	0.877
E7_MT1501_E07	MT1501	Wild DNA	aurantiogriseus (0.818), mutabilis (0.815)	0.818
F7_MT1501_F03	MT1501	Wild DNA	iconiensis (0.816), albiaxialis (0.799)	0.816
C11_MJJ1004_C08	MJJ1004	Wild DNA	qinglanensis (0.787), nanshensis (0.777)	0.787
D2_MJJ1004_D02	MJJ1004	Wild DNA	sannurensis (0.792), sodiiphilus (0.785)	0.792
B1_MJJ1002_B01	MJJ1002	Wild DNA	carnosus (0.857), pactum (0.857), olivaceus (0.857), litmocidini (0.857)	0.857
E4_MT1501_E04	MT1501	Wild DNA	sannurensis (0.778)	0.778
G3_MJJ0117_G03	MJJ0117	Wild DNA	sannurensis (0.794), monomycini (0.794)	0.794
C9__MJJ1004_C06	MJJ1004	Wild DNA	armeniacus (0.743)	0.743
B9_MJJ1002_B08	MJJ1002	Wild DNA	carnosus (0.862), pactum (0.862), olivaceus (0.862), litmocidini (0.862)	0.862
F2_MT1501_F01	MT1501	Wild DNA	violens (0.825)	0.825
C1_MT0113	MT0113	Wild DNA	armeniacus (0.792)	0.792
C5_MJJ1004_C04	MJJ1004	Wild DNA	albidoflavus (0.863)	0.863
C5_MJJ1004_C04	MJJ1004	Wild DNA	albidoflavus (0.863)	0.863
D9_MJJ1004_D08	MJJ1004	Wild DNA	sannurensis (0.865)	0.865
A10_MJJ1002_A10	MJJ1002	Wild DNA	armeniacus (0.784)	0.784

F8_MT1501_F04	MT1501	Wild DNA	koyangensis (0.803)	0.803
A1_MN2615	MN2615	Wild DNA	guanduensis (0.746), rubidus (0.744)	0.746
F3_MJJ0102	MJJ0102	Wild DNA	iconiensis (0.832), chumphonensis (0.823)	0.832
G2_MJJ0117_G02	MJJ0117	Wild DNA	qinglanensis (0.791), nanshensis (0.786)	0.791
G3_MT3509	MT3509	Wild DNA	qinglanensis (0.764)	0.764
C6_MJJ1004_C05	MJJ1004	Wild DNA	ferralitis (0.861)	0.861
B2_MJJ1002_B02	MJJ1002	Wild DNA	abyssalis (0.772), armeniacus (0.77)	0.772
A3_MN2615	MN2615	Wild DNA	cocklensis (0.812), paucisporeus (0.812)	0.812
A7_MJJ1002_A07	MJJ1002	Wild DNA	sannurensis (0.859)	0.859
B8_MJJ1002_B07	MJJ1002	Wild DNA	albidoflavus (0.855)	0.855
C4_MJJ1004_C03	MJJ1004	Wild DNA	cavourensis (0.872), flavovirens (0.87)	0.872
D4_MJJ1004_D04	MJJ1004	Wild DNA	bohaiensis (0.837)	0.837
A2_MJJ1002_A02	MJJ1002	Wild DNA	chumphonensis (0.785), chungwhensis (0.785), sparsus (0.78), yanglinensis (0.78)	0.785
G5_MJJ0117_G05	MJJ0117	Wild DNA	qinglanensis (0.816)	0.816
B7__MJJ1002_B06	MJJ1002	Wild DNA	qinglanensis (0.834)	0.834
H1_MT3509	MT3509	Wild DNA	albiaxialis (0.835)	0.835
F2_MJJ0102	MJJ0102	Wild DNA	sp. (0.779)	0.779
B3_MJJ1002_B03	MJJ1002	Wild DNA	albidoflavus (0.855)	0.855
E1_MT1501_E01	MT1501	Wild DNA	armeniacus (0.778)	0.778
H5_MJJ0117_H03	MJJ0117	Wild DNA	qinglanensis (0.753)	0.753
B1_MN2615	MN2615	Wild DNA	sulphureus (0.785), nanshensis (0.785)	0.785
B4_MJJ1002_B04	MJJ1002	Wild DNA	bohaiensis (0.831)	0.831
D3_MJJ1004_D03	MJJ1004	Wild DNA	yanglinensis (0.828)	0.828
E6_MT1501_E06	MT1501	Wild DNA	armeniacus (0.763)	0.763
D1_MT0113	MT0113	Wild DNA	armeniacus (0.809)	0.809
C2_MT0113	MT0113	Wild DNA	armeniacus (0.801)	0.801
D1_MJJ1004_D01	MJJ1004	Wild DNA	albidoflavus (0.867)	0.867
D8_MJJJ1004_D07	MJJJ1004	Wild DNA	flavovirens (0.874)	0.874
G1_MJJ0117_G01	MJJ0117	Wild DNA	exfoliatus (0.856), albidoflavus (0.854)	0.856
D6_MJJ1004_D05	MJJ1004	Wild DNA	chrestomyceticus (0.828), platensis (0.822), iconiensis (0.818)	0.828
A6_MJJ1002_A06	MJJ1002	Wild DNA	cavourensis (0.866), flavovirens (0.865), anulatus (0.861), pratensis (0.861)	0.866
B11_MJJ1002_B10	MJJ1002	Wild DNA	anulatus (0.87)	0.87
F4_MT1501_F02	MT1501	Wild DNA	iconiensis (0.851)	0.851
A4_MJJ1002_A04	MJJ1002	Wild DNA	albidoflavus (0.843)	0.843
D7_MJJ1004_D06	MJJ1004	Wild DNA	sannurensis (0.874)	0.874
H12_MJJ0117_H08	MJJ0117	Wild DNA	qinglanensis (0.798)	0.798
H7_MJJ0117_H05	MJJ0117	Wild DNA	monomycini (0.761)	0.761
H3_MJJ0117_H02	MJJ0117	Wild DNA	griseus (0.868), fungicidicus (0.868), krainskii (0.868), saprophyticus (0.868)	0.868
A8_MJJ1002_A08	MJJ1002	Wild DNA	sannurensis (0.876)	0.876
A12_MN2615_A07	MN2615	Wild DNA	albiaxialis (0.836)	0.836
F4_MJJ0102_F01	MJJ0102	Wild DNA	sannurensis (0.854)	0.854
C6_MT0113_C04	MT0113	Wild DNA	qinglanensis (0.775)	0.775

E12_MJJ0102_E09	MJJ0102	Wild DNA	sannurensis (0.858)	0.858
E7_MJJ0102_E04	MJJ0102	Wild DNA	smyrnaeus (0.78), qinglanensis (0.775), cacaoi (0.773), xinjiangensis (0.771), nanshensis (0.77	0.78
E8_MJJ0102_E05	MJJ0102	Wild DNA	lienomycini (0.849), aurantiogriseus (0.849), collinus (0.849), mutabilis (0.847)	0.849
G4_MT3509_G01	MT3509	Wild DNA	albiaxialis (0.865)	0.865
H7_MT3509_H04	MT3509	Wild DNA	spiroverticillatus (0.843), cinnamonensis (0.836), mauvecolor (0.836), polyantibioticus (0.836)	0.843
A4_MN2615_A01	MN2615	Wild DNA	qinglanensis (0.794), nodosus (0.789)	0.794
B4_MN2615_B01	MN2615	Wild DNA	sp. (0.807)	0.807
E5_MJJ0102_E03	MJJ0102	Wild DNA	albidoflavus (0.858)	0.858
F10_MJJ0102_F06	MJJ0102	Wild DNA	albidoflavus (0.871)	0.871
D5_MT0113_D03	MT0113	Wild DNA	qinglanensis (0.831), panacagri (0.826)	0.831
G9_MT3509_G06	MT3509	Wild DNA	albiaxialis (0.853)	0.853
F12_MJJ0102_F08	MJJ0102	Wild DNA	albidoflavus (0.869)	0.869
D4_MT0113_D02	MT0113	Wild DNA	glycovorans (0.787)	0.787
H6_MT3509_H03	MT3509	Wild DNA	albiaxialis (0.854)	0.854
C4_MT0113_C02	MT0113	Wild DNA	aureoverticillatus (0.849), nobilis (0.849), fumigatiscleroticus (0.849), spiralis (0.847), platensi	0.849
A9_MN2615_A04	MN2615	Wild DNA	albiaxialis (0.839)	0.839
G10_MT3509_G07	MT3509	Wild DNA	albiaxialis (0.875)	0.875
G7_MT3509_G04	MT3509	Wild DNA	finlayi (0.887)	0.887
A5_MN2615_A02	MN2615	Wild DNA	qinglanensis (0.778), nanshensis (0.768)	0.778
D6_MT0113_D04	MT0113	Wild DNA	guanduensis (0.766), paucisporeus (0.761), sporocinereus (0.754), demainii (0.752), endus (0.7	0.766
H11_MT3509_H07	MT3509	Wild DNA	albiaxialis (0.915)	0.915
D3_MT0113_D01	MT0113	Wild DNA	thermoviolaceus (0.848), thermocyaneomaculatus (0.848), thermophilus (0.831), glaucus (0.812	0.848
B10_MN2615_B05	MN2615	Wild DNA	sannurensis (0.805), bohaiensis (0.782), sodiiphilus (0.802), reptomyces glauciniger (0.789)	0.805
E9_MJJ0102_E06	MJJ0102	Wild DNA	olivaceus (0.86), litmocidini (0.86), carnosus (0.858), pactum (0.858)	0.86
G12_MT3509_G08	MT3509	Wild DNA	albiaxialis (0.852)	0.852
C7_MT0113_C05	MT0113	Wild DNA	armeniacus (0.786), xinghaiensis (0.779), chungwhensis (0.776), artemisiae (0.776)	0.786
H10_MT3509_H06	MT3509	Wild DNA	albiaxialis (0.829)	0.829
F7_MJJ0102_F03	MJJ0102	Wild DNA	intermedius (0.804)	0.804
C3_MT0113_C01	MT0113	Wild DNA	armeniacus (0.795), artemisiae (0.792), xinghaiensis (0.79), chungwhensis (0.787)	0.795
B8_MN2615_B04	MN2615	Wild DNA	qinglanensis (0.775)	0.775
D11_MT0113_D09	MT0113	Wild DNA	cocklensis (0.847), paucisporeus (0.84), mucoflavus (0.83), yeochonensis (0.828)	0.847
F8_MJJ0102_F04	MJJ0102	Wild DNA	albus subsp. coleimyceticus (0.786), platensis subsp. malvinus (0.786), sioyaensis (0.782), hygr	0.786
D10_MT0113_D08	MT0113	Wild DNA	nanshensis (0.846)	0.846
G8_MT3509_G05	MT3509	Wild DNA	albiaxialis (0.859)	0.859
A6_MN2615_A03	MN2615	Wild DNA	sparsus (0.747), qinglanensis (0.747)	0.747
H3_MT3509_H01	MT3509	Wild DNA	albiaxialis (0.928)	0.928
B11_MN2615_B06	MN2615	Wild DNA	albiaxialis (0.838)	0.838
A10_MN2615_A05	MN2615	Wild DNA	daliensis (0.737), qinglanensis (0.735), chrestomyceticus (0.734), sparsus (0.732), violens (0.73	0.737
B5_MN2615_B02	MN2615	Wild DNA	xinghaiensis (0.836), qinglanensis (0.836), paucisporeus (0.834)	0.836
H9_MT3509_H05	MT3509	Wild DNA	albiaxialis (0.831)	0.831
E11_MJJ0102_E08	MJJ0102	Wild DNA	armeniacus (0.759), qinglanensis (0.759)	0.759
E4_MJJ0102_E02	MJJ0102	Wild DNA	albidoflavus (0.776), somaliensis (0.773), albus (0.773)	0.776

D8_MT0113_D06	MT0113	Wild DNA	armeniacus (0.773), artemisiae (0.769), chungwhensis (0.763)	0.773
B6_MN2615_B03	MN2615	Wild DNA	daliensis (0.761), qinglanensis (0.758), chrestomyceticus (0.758), sparsus (0.756), albiaxialis (0	0.761
F9_MJJ0102_F05	MJJ0102	Wild DNA	armeniacus (0.765)	0.765
C8_MT0113_C06	MT0113	Wild DNA	drozdowiczii (0.792), xantholiticus (0.777), cremeus (0.773), purpureus (0.772)	0.792
H5_MT3509_H02	MT3509	Wild DNA	albidoflavus (0.867)	0.867
D7_MT0113_D05	MT0113	Wild DNA	armeniacus (0.795), xinghaiensis (0.788), chungwhensis (0.785), artemisiae (0.785)	0.795
C10_MT0113_C07	MT0113	Wild DNA	cinereospinus (0.845), coeruleofuscus (0.83), coerulescens (0.825), anandii (0.823), gougerotii	0.845
C5_MT0113_C03	MT0113	Wild DNA	qinglanensis (0.762)	0.762
E10_MJJ0102_E07	MJJ0102	Wild DNA	rubrus (0.783), chungwhensis (0.775), xinghaiensis (0.775), griseocarneus (0.773)	0.783
G6_MT3509_G03	MT3509	Wild DNA	albiaxialis (0.848)	0.848
F6_MJJ0102_F02	MJJ0102	Wild DNA	sannurensis (0.865)	0.865
E3_MJJ0102_E01	MJJ0102	Wild DNA	violascens (0.84), albidoflavus (0.838), somaliensis (0.838), exfoliatus (0.838)	0.84

Supplementary Table 4.4 Taxonomy of core *Streptomyces* ASVs represennative sequences

***Streptomyces* core ASV**	**ASV**	**Matched_Clone**	**Score**	**Taxonomy**
1	ASV_34	B5_MJJ1002_B05	97	albidoflavus (0.865)
2	ASV_191	B1_MN2615	89.806	sulphureus (0.785), nanshensis (0.785)
3	ASV_229	B4_MN2615_B01	94.581	sp. (0.807)
4	ASV_234	B1_MJJ1002_B01	96.517	carnosus (0.857), pactum (0.857), olivaceus (0.857), litmocidini (0.857)
