# Supplementary material for: Assessing similarities and disparities in the skin microbiota between wild and laboratory populations of house mice
Source: ISME J. 2020 Jun 9;14(10):2367–80. doi: 10.1038/s41396-020-0690-7 (PMC7490391; doi:10.1038/s41396-020-0690-7)
Supplement: Supplementary file 13 — Supplementary Table 5 [file 41396_2020_690_MOESM13_ESM.pdf]

Supplementary Table 5. Defined ASVs based on *tuf* gene sequencing. db: database

ASV ID	Minium score to db	Minimum db species	Maximum score to db	Maximum db species	distance to outgroup	Assigned taxonomy	Maximum db species taxonomy	Detected in Wild	Detectted in HL-Lab	Detected in MPI-Lab	Detected in C57BL/6J
ASV_1	85.751	HM352947.1	99.746	HM352959.1	83.715	Staphylococcus_equorum	Staphylococcus_equorum	Yes	No	No	No
ASV_10	86.189	HM352945.1	99.233	HM352959.1	84.399	Staphylococcus_equorum	Staphylococcus_equorum	Yes	No	No	No
ASV_1002	87.277	HM352947.1	99.237	HM352958.1	85.242	Staphylococcus_succinus	Staphylococcus_succinus	Yes	No	No	No
ASV_1003	87.5	HM352947.1	98.469	HM352950.1	85.714	Staphylococcus_xylosus	Staphylococcus_xylosus	Yes	No	No	No
ASV_1005	82.952	HM352965.1	86.514	HM352943.1	90.331	Staphylococcus sp.	Staphylococcus_hyicus	Yes	No	No	No
ASV_1009	86.514	HM352947.1	97.964	HM352950.1	84.987	Staphylococcus_xylosus	Staphylococcus_xylosus	Yes	No	No	No
ASV_1010	85.533	HM352945.1	98.477	HM352959.1	83.756	Staphylococcus_equorum	Staphylococcus_equorum	Yes	No	No	No
ASV_1011	86.768	HM352947.1	98.728	HM352950.1	85.242	Staphylococcus_xylosus	Staphylococcus_xylosus	Yes	No	No	No
ASV_1014	87.817	HM352960.1	98.477	HM352942.1	85.533	Staphylococcus_gallinarum	Staphylococcus_gallinarum	Yes	No	No	No
ASV_1015	87.31	HM352947.1	99.239	HM352958.1	85.279	Staphylococcus_succinus	Staphylococcus_succinus	No	No	No	No
ASV_1016	82.653	HM352965.1	87.5	HM352954.1	86.224	Staphylococcus sp.	Staphylococcus_arlettae	Yes	No	No	No
ASV_1017	82.443	HM352919.1	86.041	HM352941.1	84.01	Staphylococcus sp.	Staphylococcus_felis	Yes	No	No	No
ASV_1019	87.023	HM352947.1	97.455	HM352950.1	85.496	Staphylococcus_xylosus	Staphylococcus_xylosus	Yes	No	No	No
ASV_102	87.5	HM352947.1	98.469	HM352950.1	85.714	Staphylococcus_xylosus	Staphylococcus_xylosus	Yes	No	No	No
ASV_1020	82.443	HM352923.1	86.514	HM352941.1	82.697	Staphylococcus sp.	Staphylococcus_felis	Yes	No	No	No
ASV_1026	81.98	HM352919.1	85.787	HM352941.1	82.741	Staphylococcus sp.	Staphylococcus_felis	Yes	No	No	No
ASV_1028	82.487	HM352945.1	86.041	HM352958.1	85.787	Staphylococcus sp.	Staphylococcus_succinus	Yes	No	No	No
ASV_1029	87.023	HM352961.1	99.237	HM352950.1	84.987	Staphylococcus_xylosus	Staphylococcus_xylosus	Yes	No	No	No
ASV_103	87.212	HM352961.1	100	HM352950.1	85.422	Staphylococcus_xylosus	Staphylococcus_xylosus	No	No	Yes	No
ASV_1030	87.212	HM352961.1	100	HM352950.1	85.422	Staphylococcus_xylosus	Staphylococcus_xylosus	Yes	No	No	No
ASV_1034	82.741	HM352958.1	86.294	HM352961.1	84.772	Staphylococcus sp.	Staphylococcus_fleurettii	Yes	No	No	No
ASV_1039	81.425	HM352956.1	86.768	HM352947.1	86.26	Staphylococcus sp.	Staphylococcus_sciuri	No	No	No	No
ASV_1040	86.445	HM352947.1	96.419	HM352950.1	85.166	Staphylococcus sp.	Staphylococcus_xylosus	Yes	No	No	No
ASV_1042	86.26	HM352961.1	98.982	HM352950.1	84.478	Staphylococcus_xylosus	Staphylococcus_xylosus	Yes	No	No	No
ASV_1045	85.025	HM352947.1	98.985	HM352959.1	82.995	Staphylococcus_equorum	Staphylococcus_equorum	Yes	No	No	No
ASV_1049	88.295	HM352960.1	98.982	HM352942.1	86.005	Staphylococcus_gallinarum	Staphylococcus_gallinarum	Yes	No	No	No
ASV_105	86.514	HM352947.1	98.473	HM352950.1	84.733	Staphylococcus_xylosus	Staphylococcus_xylosus	Yes	No	No	No
ASV_1051	81.633	HM352956.1	86.99	HM352947.1	86.48	Staphylococcus sp.	Staphylococcus_sciuri	No	Yes	No	No
ASV_1053	85.533	HM352945.1	98.223	HM352959.1	83.756	Staphylococcus_equorum	Staphylococcus_equorum	Yes	No	No	No
ASV_1056	85.496	HM352947.1	99.491	HM352959.1	83.461	Staphylococcus_equorum	Staphylococcus_equorum	Yes	No	No	No
ASV_1057	86.99	HM352947.1	97.959	HM352950.1	85.204	Staphylococcus_xylosus	Staphylococcus_xylosus	Yes	No	No	No
ASV_1059	82.741	HM352919.1	87.056	HM352954.1	86.548	Staphylococcus sp.	Staphylococcus_arlettae	Yes	No	No	No
ASV_106	87.563	HM352960.1	98.985	HM352942.1	85.279	Staphylococcus_gallinarum	Staphylococcus_gallinarum	Yes	No	Yes	No
ASV_1067	85.025	HM352940.1	98.477	HM352959.1	82.995	Staphylococcus_equorum	Staphylococcus_equorum	Yes	No	No	No
ASV_1069	82.653	HM352923.1	86.735	HM352941.1	82.908	Staphylococcus sp.	Staphylococcus_felis	Yes	No	No	No
ASV_107	87.31	HM352965.1	97.208	HM352919.1	87.056	Staphylococcus_aureus	Staphylococcus_aureus	No	No	No	No
ASV_1070	87.468	HM352947.1	99.488	HM352958.1	85.422	Staphylococcus_succinus	Staphylococcus_succinus	Yes	No	No	No
ASV_1071	82.864	HM352944.1	86.445	HM352941.1	86.957	Staphylococcus sp.	Staphylococcus_felis	Yes	No	No	No
ASV_1073	85.025	HM352965.1	93.655	HM352960.1	87.056	Staphylococcus sp.	Staphylococcus_vitulinus	Yes	No	No	No
ASV_108	85.496	HM352960.1	96.438	HM352950.1	84.733	Staphylococcus sp.	Staphylococcus_xylosus	Yes	No	No	No
ASV_1080	82.908	HM352919.1	86.514	HM352941.1	84.478	Staphylococcus sp.	Staphylococcus_felis	No	Yes	No	No
ASV_1083	87.786	HM352960.1	95.929	HM352938.1	84.478	Staphylococcus sp.	Staphylococcus_cohnii	Yes	No	No	No
ASV_1084	83.12	HM352965.1	88.235	HM352929.1	88.235	Staphylococcus sp.	Staphylococcus_pasteuri	Yes	No	No	No
ASV_1085	85.934	HM352947.1	100	HM352959.1	83.887	Staphylococcus_equorum	Staphylococcus_equorum	Yes	No	No	No
ASV_1087	88.747	HM352945.1	100	HM352954.1	86.957	Staphylococcus_arlettae	Staphylococcus_arlettae	Yes	No	No	No
ASV_1088	87.786	HM352947.1	99.746	HM352958.1	85.751	Staphylococcus_succinus	Staphylococcus_succinus	Yes	No	No	No
ASV_109	85.787	HM352961.1	98.477	HM352950.1	84.01	Staphylococcus_xylosus	Staphylococcus_xylosus	Yes	Yes	Yes	No
ASV_1090	86.548	HM352947.1	96.954	HM352950.1	85.025	Staphylococcus sp.	Staphylococcus_xylosus	Yes	No	No	No
ASV_1094	85.025	HM352959.1	98.985	HM352947.1	89.34	Staphylococcus_sciuri	Staphylococcus_sciuri	Yes	No	No	No
ASV_1097	86.548	HM352961.1	98.731	HM352950.1	84.518	Staphylococcus_xylosus	Staphylococcus_xylosus	Yes	No	No	No
ASV_1099	86.99	HM352961.1	99.235	HM352950.1	85.459	Staphylococcus_xylosus	Staphylococcus_xylosus	Yes	No	No	No
ASV_11	87.31	HM352947.1	99.239	HM352958.1	85.279	Staphylococcus_succinus	Staphylococcus_succinus	Yes	No	Yes	No
ASV_110	88.295	HM352960.1	99.746	HM352942.1	86.005	Staphylococcus_gallinarum	Staphylococcus_gallinarum	Yes	No	No	No
ASV_1101	86.445	HM352947.1	96.931	HM352950.1	85.678	Staphylococcus sp.	Staphylococcus_xylosus	Yes	No	No	No
ASV_1104	80.964	HM352926.1	87.056	HM352936.1	86.294	Staphylococcus sp.	Staphylococcus_schleiferi	Yes	No	No	No
ASV_1106	82.487	HM352919.1	86.802	HM352954.1	86.294	Staphylococcus sp.	Staphylococcus_arlettae	No	No	Yes	No
ASV_111	85.279	HM352945.1	98.477	HM352959.1	83.503	Staphylococcus_equorum	Staphylococcus_equorum	Yes	No	Yes	No
ASV_1113	81.472	HM352961.1	93.655	HM352950.1	79.695	Staphylococcus sp.	Staphylococcus_xylosus	Yes	No	No	No
ASV_1116	86.957	HM352947.1	98.977	HM352950.1	85.422	Staphylococcus_xylosus	Staphylococcus_xylosus	Yes	No	No	No
ASV_1117	86.957	HM352947.1	99.488	HM352950.1	85.166	Staphylococcus_xylosus	Staphylococcus_xylosus	Yes	No	No	No
ASV_112	86.802	HM352947.1	97.716	HM352950.1	85.025	Staphylococcus_xylosus	Staphylococcus_xylosus	Yes	No	No	No
ASV_1123	82.653	HM352926.1	85.969	HM352951.1	85.969	Staphylococcus sp.	Staphylococcus_kloosii	Yes	No	No	No
ASV_1125	85.751	HM352940.1	99.491	HM352959.1	83.715	Staphylococcus_equorum	Staphylococcus_equorum	Yes	No	No	No
ASV_1128	82.398	HM352923.1	86.735	HM352949.1	83.673	Staphylococcus sp.	Staphylococcus_simulans	Yes	No	No	No

ASV_1130	86.701	HM352965.1	97.442	HM352923.1	86.957	Staphylococcus_haemolyticus	Staphylococcus_haemolyticus	Yes	No	No	No
ASV_1131	87.724	HM352960.1	99.233	HM352935.1	85.678	Staphylococcus_saprophyticus	Staphylococcus_saprophyticus	Yes	No	No	No
ASV_1132	86.514	HM352959.1	98.728	HM352944.1	88.804	Staphylococcus_lentus	Staphylococcus_lentus	Yes	No	No	No
ASV_1140	85.533	HM352947.1	95.939	HM352950.1	84.772	Staphylococcus sp.	Staphylococcus_xylosus	Yes	No	No	No
ASV_1142	83.249	HM352957.1	86.294	HM352920.1	83.756	Staphylococcus sp.	Staphylococcus_capitis	Yes	No	No	No
ASV_1144	85.025	HM352947.1	98.985	HM352959.1	82.995	Staphylococcus_equorum	Staphylococcus_equorum	Yes	No	No	No
ASV_1145	81.586	HM352956.1	86.957	HM352947.1	86.445	Staphylococcus sp.	Staphylococcus_sciuri	Yes	No	No	No
ASV_1146	86.224	HM352945.1	98.98	HM352959.1	84.439	Staphylococcus_equorum	Staphylococcus_equorum	Yes	No	No	No
ASV_1147	86.041	HM352947.1	94.924	HM352950.1	84.264	Staphylococcus sp.	Staphylococcus_xylosus	Yes	No	No	No
ASV_1149	85.533	HM352945.1	98.223	HM352959.1	84.264	Staphylococcus_equorum	Staphylococcus_equorum	Yes	No	No	No
ASV_1154	81.218	HM352956.1	87.056	HM352947.1	86.548	Staphylococcus sp.	Staphylococcus_sciuri	Yes	No	No	No
ASV_1155	86.548	HM352947.1	97.462	HM352950.1	84.772	Staphylococcus_xylosus	Staphylococcus_xylosus	Yes	No	No	No
ASV_116	86.768	HM352947.1	98.728	HM352950.1	85.242	Staphylococcus_xylosus	Staphylococcus_xylosus	Yes	Yes	No	No
ASV_1160	81.98	HM352955.1	86.294	HM352942.1	85.279	Staphylococcus sp.	Staphylococcus_gallinarum	Yes	No	No	No
ASV_1161	86.224	HM352945.1	99.235	HM352959.1	84.439	Staphylococcus_equorum	Staphylococcus_equorum	Yes	No	No	No
ASV_1165	82.697	HM352945.1	86.768	HM352953.1	86.005	Staphylococcus sp.	Staphylococcus_carnosus	Yes	No	No	No
ASV_1166	87.31	HM352947.1	99.239	HM352958.1	85.279	Staphylococcus_succinus	Staphylococcus_succinus	Yes	No	No	No
ASV_1167	87.786	HM352945.1	95.674	HM352938.1	84.733	Staphylococcus sp.	Staphylococcus_cohnii	Yes	No	No	No
ASV_1169	82.864	HM352919.1	86.99	HM352949.1	84.949	Staphylococcus sp.	Staphylococcus_simulans	No	No	No	No
ASV_117	87.056	HM352965.1	97.208	HM352922.1	86.548	Staphylococcus_epidermidis	Staphylococcus_epidermidis	Yes	Yes	No	No
ASV_1173	85.025	HM352959.1	98.985	HM352947.1	89.34	Staphylococcus_sciuri	Staphylococcus_sciuri	Yes	No	No	No
ASV_1174	86.294	HM352961.1	98.477	HM352950.1	84.772	Staphylococcus_xylosus	Staphylococcus_xylosus	Yes	No	No	No
ASV_1176	87.023	HM352947.1	98.473	HM352950.1	85.242	Staphylococcus_xylosus	Staphylococcus_xylosus	Yes	No	No	No
ASV_1178	86.445	HM352961.1	99.233	HM352950.1	84.655	Staphylococcus_xylosus	Staphylococcus_xylosus	Yes	No	No	No
ASV_118	86.768	HM352961.1	98.982	HM352950.1	85.242	Staphylococcus_xylosus	Staphylococcus_xylosus	Yes	No	No	No
ASV_1180	85.934	HM352945.1	99.233	HM352959.1	84.143	Staphylococcus_equorum	Staphylococcus_equorum	Yes	No	No	No
ASV_1182	87.786	HM352947.1	99.746	HM352958.1	85.751	Staphylococcus_succinus	Staphylococcus_succinus	Yes	No	No	No
ASV_1183	82.653	HM352919.1	86.48	HM352941.1	83.418	Staphylococcus sp.	Staphylococcus_felis	Yes	No	No	No
ASV_1185	81.934	HM352950.1	86.005	HM352947.1	88.295	Staphylococcus sp.	Staphylococcus_sciuri	Yes	No	No	No
ASV_1187	82.697	HM352919.1	86.294	HM352941.1	84.264	Staphylococcus sp.	Staphylococcus_felis	Yes	No	No	No
ASV_1188	86.802	HM352947.1	97.716	HM352950.1	85.025	Staphylococcus_xylosus	Staphylococcus_xylosus	Yes	No	No	No
ASV_1189	83.418	HM352926.1	89.031	HM352952.1	86.99	Staphylococcus sp.	Staphylococcus_chromogenes	Yes	No	No	No
ASV_119	85.025	HM352959.1	98.985	HM352947.1	89.34	Staphylococcus_sciuri	Staphylococcus_sciuri	Yes	No	No	No
ASV_1192	85.934	HM352959.1	100	HM352947.1	89.77	Staphylococcus_sciuri	Staphylococcus_sciuri	Yes	No	No	No
ASV_1196	81.586	HM352944.1	85.934	HM352949.1	82.609	Staphylococcus sp.	Staphylococcus_simulans	Yes	No	No	No
ASV_1197	87.023	HM352961.1	99.237	HM352950.1	84.987	Staphylococcus_xylosus	Staphylococcus_xylosus	Yes	No	No	No
ASV_12	85.279	HM352947.1	99.239	HM352959.1	83.249	Staphylococcus_equorum	Staphylococcus_equorum	Yes	No	Yes	No
ASV_1208	83.929	HM352965.1	88.01	HM352928.1	86.48	Staphylococcus sp.	Staphylococcus_caprae	Yes	No	No	No
ASV_121	85.533	HM352945.1	98.477	HM352959.1	83.756	Staphylococcus_equorum	Staphylococcus_equorum	Yes	No	No	No
ASV_1212	82.487	HM352944.1	87.817	HM352952.1	85.279	Staphylococcus sp.	Staphylococcus_chromogenes	Yes	No	No	No
ASV_1217	81.074	HM352926.1	86.189	HM352949.1	84.91	Staphylococcus sp.	Staphylococcus_simulans	Yes	No	No	No
ASV_1219	83.163	HM352965.1	87.245	HM352954.1	85.204	Staphylococcus sp.	Staphylococcus_arlettae	Yes	No	No	No
ASV_122	87.212	HM352947.1	98.21	HM352950.1	85.422	Staphylococcus_xylosus	Staphylococcus_xylosus	Yes	No	No	No
ASV_1225	86.224	HM352947.1	99.49	HM352959.1	84.184	Staphylococcus_equorum	Staphylococcus_equorum	Yes	No	No	No
ASV_1227	85.533	HM352940.1	97.97	HM352959.1	83.503	Staphylococcus_equorum	Staphylococcus_equorum	Yes	No	No	No
ASV_1229	86.005	HM352947.1	96.438	HM352950.1	85.242	Staphylococcus sp.	Staphylococcus_xylosus	Yes	No	No	No
ASV_1230	87.212	HM352961.1	99.488	HM352950.1	85.166	Staphylococcus_xylosus	Staphylococcus_xylosus	Yes	No	No	No
ASV_1233	83.632	HM352947.1	96.419	HM352959.1	81.586	Staphylococcus sp.	Staphylococcus_equorum	Yes	No	No	No
ASV_1234	83.461	HM352945.1	86.768	HM352963.1	86.768	Staphylococcus sp.	Staphylococcus_pettenkoferi	Yes	No	No	No
ASV_1236	83.163	HM352919.1	86.768	HM352941.1	84.733	Staphylococcus sp.	Staphylococcus_felis	Yes	No	No	No
ASV_1237	81.679	HM352965.1	87.786	HM352961.1	87.277	Staphylococcus sp.	Staphylococcus_fleurettii	Yes	No	No	No
ASV_1239	86.041	HM352959.1	97.716	HM352944.1	88.325	Staphylococcus_lentus	Staphylococcus_lentus	Yes	No	No	No
ASV_124	82.952	HM352919.1	86.548	HM352941.1	84.518	Staphylococcus sp.	Staphylococcus_felis	No	Yes	No	No
ASV_1243	86.548	HM352961.1	98.731	HM352950.1	84.518	Staphylococcus_xylosus	Staphylococcus_xylosus	Yes	No	No	No
ASV_125	86.041	HM352955.1	98.731	HM352944.1	88.325	Staphylococcus_lentus	Staphylococcus_lentus	Yes	No	No	No
ASV_1250	87.056	HM352960.1	98.477	HM352935.1	85.025	Staphylococcus_saprophyticus	Staphylococcus_saprophyticus	Yes	No	No	No
ASV_1252	87.468	HM352965.1	97.698	HM352922.1	86.957	Staphylococcus_epidermidis	Staphylococcus_epidermidis	Yes	No	No	No
ASV_1256	86.735	HM352959.1	98.469	HM352944.1	89.031	Staphylococcus_lentus	Staphylococcus_lentus	Yes	No	No	No
ASV_1257	85.025	HM352959.1	98.985	HM352947.1	89.34	Staphylococcus_sciuri	Staphylococcus_sciuri	Yes	No	No	No
ASV_1259	86.768	HM352947.1	98.728	HM352950.1	85.242	Staphylococcus_xylosus	Staphylococcus_xylosus	Yes	No	No	No
ASV_126	82.995	HM352957.1	86.041	HM352924.1	85.279	Staphylococcus sp.	Staphylococcus_hominis	Yes	No	No	No
ASV_1260	85.279	HM352959.1	99.239	HM352947.1	89.086	Staphylococcus_sciuri	Staphylococcus_sciuri	Yes	No	No	No
ASV_1267	82.995	HM352940.1	86.294	HM352954.1	84.518	Staphylococcus sp.	Staphylococcus_arlettae	Yes	No	No	No
ASV_1268	82.741	HM352965.1	88.325	HM352947.1	88.325	Staphylococcus sp.	Staphylococcus_sciuri	Yes	No	No	No
ASV_127	86.548	HM352961.1	98.731	HM352950.1	84.518	Staphylococcus_xylosus	Staphylococcus_xylosus	Yes	No	No	No
ASV_1270	83.461	HM352965.1	87.532	HM352928.1	86.26	Staphylococcus sp.	Staphylococcus_caprae	Yes	No	No	No
ASV_1271	82.443	HM352919.1	86.041	HM352941.1	84.01	Staphylococcus sp.	Staphylococcus_felis	Yes	No	No	No

ASV_1276	82.697	HM352919.1	86.294	HM352941.1	84.264	Staphylococcus sp.	Staphylococcus_felis	Yes	No	No	No
ASV_1278	85.787	HM352947.1	94.924	HM352959.1	84.01	Staphylococcus sp.	Staphylococcus_equorum	Yes	No	No	No
ASV_1286	86.548	HM352947.1	97.462	HM352950.1	84.772	Staphylococcus_xylosus	Staphylococcus_xylosus	Yes	No	No	No
ASV_1290	81.282	HM352919.1	86.189	HM352949.1	85.166	Staphylococcus sp.	Staphylococcus_simulans	Yes	No	No	No
ASV_1291	86.768	HM352961.1	98.982	HM352950.1	85.242	Staphylococcus_xylosus	Staphylococcus_xylosus	Yes	No	No	No
ASV_1292	86.802	HM352947.1	98.731	HM352958.1	84.772	Staphylococcus_succinus	Staphylococcus_succinus	Yes	No	No	No
ASV_1295	82.952	HM352965.1	87.023	HM352954.1	84.478	Staphylococcus sp.	Staphylococcus_arlettae	Yes	No	No	No
ASV_1298	80.457	HM352959.1	86.802	HM352957.1	87.056	Staphylococcus sp.	Staphylococcus_muscae	Yes	No	No	No
ASV_1299	87.212	HM352961.1	99.488	HM352950.1	85.166	Staphylococcus_xylosus	Staphylococcus_xylosus	Yes	No	No	No
ASV_13	87.31	HM352960.1	95.431	HM352938.1	84.01	Staphylococcus sp.	Staphylococcus_cohnii	Yes	No	Yes	No
ASV_130	87.31	HM352960.1	95.431	HM352938.1	84.01	Staphylococcus sp.	Staphylococcus_cohnii	Yes	No	No	No
ASV_1300	82.353	HM352955.1	87.724	HM352962.1	88.235	Staphylococcus sp.	Staphylococcus_pseudintermedius	Yes	No	No	No
ASV_1301	86.294	HM352961.1	98.477	HM352950.1	84.772	Staphylococcus_xylosus	Staphylococcus_xylosus	Yes	No	No	No
ASV_1304	85.714	HM352947.1	99.745	HM352959.1	83.673	Staphylococcus_equorum	Staphylococcus_equorum	Yes	No	No	No
ASV_1311	82.234	HM352926.1	87.056	HM352951.1	87.056	Staphylococcus sp.	Staphylococcus_kloosii	Yes	No	No	No
ASV_1312	81.472	HM352965.1	86.768	HM352920.1	86.041	Staphylococcus sp.	Staphylococcus_capitis	Yes	No	No	No
ASV_1315	83.12	HM352945.1	86.701	HM352958.1	86.445	Staphylococcus sp.	Staphylococcus_succinus	Yes	No	No	No
ASV_1318	82.443	HM352919.1	86.041	HM352941.1	84.01	Staphylococcus sp.	Staphylococcus_felis	Yes	No	No	No
ASV_1319	86.294	HM352947.1	98.223	HM352950.1	84.772	Staphylococcus_xylosus	Staphylococcus_xylosus	Yes	No	No	No
ASV_1323	82.908	HM352919.1	86.514	HM352941.1	84.478	Staphylococcus sp.	Staphylococcus_felis	Yes	No	No	No
ASV_1326	83.715	HM352944.1	87.277	HM352941.1	87.532	Staphylococcus sp.	Staphylococcus_felis	No	Yes	No	No
ASV_133	85.751	HM352947.1	99.491	HM352959.1	83.715	Staphylococcus_equorum	Staphylococcus_equorum	Yes	No	No	No
ASV_1331	82.741	HM352923.1	86.802	HM352941.1	82.995	Staphylococcus sp.	Staphylococcus_felis	Yes	No	No	No
ASV_1333	87.212	HM352947.1	98.21	HM352950.1	85.422	Staphylococcus_xylosus	Staphylococcus_xylosus	Yes	No	No	No
ASV_1337	82.995	HM352919.1	86.802	HM352961.1	87.056	Staphylococcus sp.	Staphylococcus_fleurettii	Yes	No	No	No
ASV_1338	80.867	HM352923.1	86.224	HM352949.1	82.908	Staphylococcus sp.	Staphylococcus_simulans	Yes	No	No	No
ASV_1340	81.934	HM352947.1	95.929	HM352959.1	79.898	Staphylococcus sp.	Staphylococcus_equorum	Yes	No	No	No
ASV_1341	82.995	HM352928.1	86.548	HM352947.1	85.533	Staphylococcus sp.	Staphylococcus_sciuri	No	No	No	No
ASV_1345	82.353	HM352923.1	86.957	HM352949.1	83.887	Staphylococcus sp.	Staphylococcus_simulans	Yes	No	No	No
ASV_1347	85.496	HM352947.1	99.491	HM352959.1	83.461	Staphylococcus_equorum	Staphylococcus_equorum	Yes	No	No	No
ASV_1348	87.245	HM352947.1	97.704	HM352950.1	85.714	Staphylococcus_xylosus	Staphylococcus_xylosus	Yes	No	No	No
ASV_1349	86.802	HM352944.1	98.731	HM352955.1	87.056	Staphylococcus_piscifermentans	Staphylococcus_piscifermentans	Yes	No	No	No
ASV_135	86.548	HM352947.1	97.462	HM352950.1	84.772	Staphylococcus_xylosus	Staphylococcus_xylosus	Yes	No	Yes	No
ASV_1350	82.487	HM352945.1	86.294	HM352953.1	85.279	Staphylococcus sp.	Staphylococcus_carnosus	Yes	No	No	No
ASV_1351	81.934	HM352950.1	86.26	HM352961.1	89.567	Staphylococcus sp.	Staphylococcus_fleurettii	Yes	No	No	No
ASV_1355	85.787	HM352959.1	98.985	HM352944.1	88.071	Staphylococcus_lentus	Staphylococcus_lentus	Yes	No	No	No
ASV_1366	81.472	HM352923.1	85.787	HM352941.1	82.234	Staphylococcus sp.	Staphylococcus_felis	Yes	No	No	No
ASV_137	85.969	HM352947.1	100	HM352959.1	83.929	Staphylococcus_equorum	Staphylococcus_equorum	Yes	No	No	No
ASV_1370	80.916	HM352919.1	85.787	HM352949.1	84.518	Staphylococcus sp.	Staphylococcus_simulans	Yes	No	No	No
ASV_1374	87.755	HM352947.1	99.745	HM352958.1	85.714	Staphylococcus_succinus	Staphylococcus_succinus	Yes	No	No	No
ASV_1380	84.478	HM352945.1	90.585	HM352959.1	83.969	Staphylococcus sp.	Staphylococcus_equorum	Yes	No	No	No
ASV_1382	85.533	HM352947.1	95.939	HM352950.1	84.772	Staphylococcus sp.	Staphylococcus_xylosus	Yes	No	No	No
ASV_1383	86.041	HM352947.1	97.97	HM352950.1	84.518	Staphylococcus_xylosus	Staphylococcus_xylosus	Yes	No	No	No
ASV_1384	80.964	HM352956.1	86.294	HM352947.1	85.787	Staphylococcus sp.	Staphylococcus_sciuri	No	No	No	No
ASV_139	86.99	HM352959.1	98.724	HM352944.1	89.286	Staphylococcus_lentus	Staphylococcus_lentus	Yes	No	No	No
ASV_1394	81.218	HM352956.1	87.056	HM352947.1	86.548	Staphylococcus sp.	Staphylococcus_sciuri	Yes	No	No	No
ASV_1395	82.952	HM352947.1	93.384	HM352950.1	82.188	Staphylococcus sp.	Staphylococcus_xylosus	Yes	No	No	No
ASV_1396	87.277	HM352965.1	97.71	HM352924.1	87.277	Staphylococcus_hominis	Staphylococcus_hominis	No	No	Yes	No
ASV_1399	86.768	HM352961.1	98.982	HM352950.1	85.242	Staphylococcus_xylosus	Staphylococcus_xylosus	Yes	No	No	No
ASV_14	87.023	HM352961.1	99.237	HM352950.1	84.987	Staphylococcus_xylosus	Staphylococcus_xylosus	Yes	No	Yes	No
ASV_1400	86.26	HM352961.1	98.982	HM352950.1	84.478	Staphylococcus_xylosus	Staphylococcus_xylosus	Yes	No	No	No
ASV_1404	82.741	HM352947.1	96.701	HM352959.1	80.711	Staphylococcus sp.	Staphylococcus_equorum	Yes	No	No	No
ASV_1408	85.751	HM352965.1	99.491	HM352962.1	86.768	taphylococcus_pseudintermediu	Staphylococcus_pseudintermedius	Yes	No	No	No
ASV_141	85.969	HM352947.1	100	HM352959.1	83.929	Staphylococcus_equorum	Staphylococcus_equorum	Yes	No	No	No
ASV_1411	82.697	HM352919.1	86.294	HM352941.1	84.264	Staphylococcus sp.	Staphylococcus_felis	Yes	No	No	No
ASV_1412	82.995	HM352945.1	86.294	HM352963.1	86.294	Staphylococcus sp.	Staphylococcus_pettenkoferi	Yes	No	No	No
ASV_1416	86.802	HM352947.1	98.731	HM352958.1	84.772	Staphylococcus_succinus	Staphylococcus_succinus	No	No	Yes	No
ASV_1418	82.864	HM352919.1	86.99	HM352949.1	84.949	Staphylococcus sp.	Staphylococcus_simulans	No	No	No	No
ASV_142	86.548	HM352947.1	96.954	HM352950.1	85.025	Staphylococcus sp.	Staphylococcus_xylosus	Yes	No	No	No
ASV_1422	82.487	HM352965.1	87.563	HM352963.1	88.071	Staphylococcus sp.	Staphylococcus_pettenkoferi	Yes	No	No	No
ASV_1425	82.143	HM352923.1	86.48	HM352941.1	82.908	Staphylococcus sp.	Staphylococcus_felis	Yes	No	No	No
ASV_1429	81.934	HM352965.1	87.277	HM352949.1	88.55	Staphylococcus sp.	Staphylococcus_simulans	Yes	No	No	No
ASV_143	82.697	HM352919.1	86.294	HM352941.1	84.264	Staphylococcus sp.	Staphylococcus_felis	Yes	No	Yes	No
ASV_1436	81.122	HM352919.1	86.005	HM352949.1	84.987	Staphylococcus sp.	Staphylococcus_simulans	Yes	No	No	No
ASV_1437	82.952	HM352956.1	88.55	HM352961.1	89.059	Staphylococcus sp.	Staphylococcus_fleurettii	Yes	No	No	No
ASV_144	88.776	HM352961.1	96.939	HM352951.1	86.735	Staphylococcus sp.	Staphylococcus_kloosii	Yes	No	No	No
ASV_1440	84.733	HM352926.1	93.13	HM352959.1	86.005	Staphylococcus sp.	Staphylococcus_equorum	Yes	No	No	No

ASV_1441	82.908	HM352919.1	86.514	HM352941.1	84.478	Staphylococcus sp.	Staphylococcus_felis	Yes	No	No	No
ASV_1443	80.916	HM352919.1	85.787	HM352949.1	84.518	Staphylococcus sp.	Staphylococcus_simulans	Yes	No	No	No
ASV_1444	83.503	HM352965.1	87.056	HM352957.1	87.563	Staphylococcus sp.	Staphylococcus_muscae	No	No	Yes	No
ASV_145	80.964	HM352956.1	86.294	HM352947.1	85.787	Staphylococcus sp.	Staphylococcus_sciuri	Yes	No	Yes	No
ASV_1451	82.487	HM352947.1	96.447	HM352959.1	80.457	Staphylococcus sp.	Staphylococcus_equorum	Yes	No	No	No
ASV_1452	82.697	HM352919.1	86.294	HM352941.1	84.264	Staphylococcus sp.	Staphylococcus_felis	Yes	No	No	No
ASV_1456	85.279	HM352945.1	97.97	HM352959.1	83.503	Staphylococcus_equorum	Staphylococcus_equorum	Yes	No	No	No
ASV_1457	86.26	HM352961.1	98.982	HM352950.1	84.478	Staphylococcus_xylosus	Staphylococcus_xylosus	Yes	No	No	No
ASV_1458	82.952	HM352945.1	86.514	HM352958.1	86.26	Staphylococcus sp.	Staphylococcus_succinus	Yes	No	No	No
ASV_146	87.817	HM352945.1	98.985	HM352954.1	86.041	Staphylococcus_arlettae	Staphylococcus_arlettae	Yes	No	Yes	No
ASV_1469	82.864	HM352919.1	86.99	HM352949.1	84.949	Staphylococcus sp.	Staphylococcus_simulans	Yes	No	No	No
ASV_147	86.445	HM352947.1	96.931	HM352950.1	85.678	Staphylococcus sp.	Staphylococcus_xylosus	Yes	No	No	No
ASV_1471	86.041	HM352959.1	99.239	HM352960.1	88.832	Staphylococcus_vitulinus	Staphylococcus_vitulinus	Yes	No	No	No
ASV_1473	82.443	HM352919.1	86.041	HM352941.1	84.01	Staphylococcus sp.	Staphylococcus_felis	Yes	No	No	No
ASV_148	82.188	HM352919.1	86.294	HM352949.1	84.264	Staphylococcus sp.	Staphylococcus_simulans	Yes	Yes	No	No
ASV_1483	82.697	HM352947.1	93.13	HM352950.1	81.934	Staphylococcus sp.	Staphylococcus_xylosus	Yes	No	No	No
ASV_149	86.735	HM352959.1	98.98	HM352944.1	89.031	Staphylococcus_lentus	Staphylococcus_lentus	Yes	No	No	No
ASV_1491	87.31	HM352960.1	95.939	HM352938.1	84.01	Staphylococcus sp.	Staphylococcus_cohnii	Yes	No	No	No
ASV_1492	85.025	HM352960.1	89.594	HM352950.1	84.01	Staphylococcus sp.	Staphylococcus_xylosus	Yes	No	No	No
ASV_1493	87.277	HM352947.1	98.219	HM352950.1	85.496	Staphylococcus_xylosus	Staphylococcus_xylosus	Yes	No	No	No
ASV_1497	80.457	HM352965.1	86.041	HM352923.1	86.802	Staphylococcus sp.	Staphylococcus_haemolyticus	Yes	No	No	No
ASV_15	85.751	HM352945.1	98.982	HM352959.1	83.969	Staphylococcus_equorum	Staphylococcus_equorum	Yes	No	No	No
ASV_150	86.957	HM352947.1	98.977	HM352950.1	85.422	Staphylococcus_xylosus	Staphylococcus_xylosus	Yes	No	No	No
ASV_1501	87.31	HM352960.1	95.431	HM352938.1	84.01	Staphylococcus sp.	Staphylococcus_cohnii	No	No	Yes	No
ASV_1512	86.802	HM352965.1	97.208	HM352927.1	87.31	Staphylococcus_warneri	Staphylococcus_warneri	Yes	No	No	No
ASV_1514	82.741	HM352956.1	87.056	HM352953.1	84.518	Staphylococcus sp.	Staphylococcus_carnosus	Yes	No	No	No
ASV_1515	86.294	HM352961.1	97.462	HM352950.1	84.772	Staphylococcus_xylosus	Staphylococcus_xylosus	Yes	No	No	No
ASV_1521	85.787	HM352961.1	98.477	HM352950.1	84.01	Staphylococcus_xylosus	Staphylococcus_xylosus	Yes	No	No	No
ASV_1522	87.31	HM352965.1	97.208	HM352919.1	87.056	Staphylococcus_aureus	Staphylococcus_aureus	Yes	No	No	No
ASV_1523	87.468	HM352947.1	99.488	HM352958.1	85.422	Staphylococcus_succinus	Staphylococcus_succinus	No	No	Yes	No
ASV_1530	87.31	HM352960.1	95.431	HM352938.1	84.01	Staphylococcus sp.	Staphylococcus_cohnii	Yes	No	No	No
ASV_1531	85.533	HM352945.1	98.731	HM352959.1	83.756	Staphylococcus_equorum	Staphylococcus_equorum	Yes	No	No	No
ASV_1532	85.279	HM352947.1	99.239	HM352959.1	83.249	Staphylococcus_equorum	Staphylococcus_equorum	Yes	No	No	No
ASV_1533	86.802	HM352960.1	94.416	HM352938.1	83.503	Staphylococcus sp.	Staphylococcus_cohnii	Yes	No	No	No
ASV_1535	87.056	HM352960.1	95.178	HM352938.1	83.756	Staphylococcus sp.	Staphylococcus_cohnii	Yes	No	No	No
ASV_1538	86.768	HM352961.1	98.982	HM352950.1	85.242	Staphylococcus_xylosus	Staphylococcus_xylosus	Yes	No	No	No
ASV_1539	87.056	HM352960.1	95.178	HM352938.1	83.756	Staphylococcus sp.	Staphylococcus_cohnii	Yes	No	No	No
ASV_154	82.188	HM352956.1	87.532	HM352947.1	87.023	Staphylococcus sp.	Staphylococcus_sciuri	Yes	No	No	No
ASV_1542	85.969	HM352945.1	99.745	HM352959.1	84.184	Staphylococcus_equorum	Staphylococcus_equorum	Yes	No	No	No
ASV_1543	82.908	HM352961.1	95.663	HM352950.1	81.122	Staphylococcus sp.	Staphylococcus_xylosus	Yes	No	No	No
ASV_1544	87.31	HM352960.1	95.431	HM352938.1	84.01	Staphylococcus sp.	Staphylococcus_cohnii	Yes	No	No	No
ASV_1547	85.279	HM352945.1	98.477	HM352959.1	83.503	Staphylococcus_equorum	Staphylococcus_equorum	Yes	No	No	No
ASV_1548	87.31	HM352960.1	95.431	HM352938.1	84.01	Staphylococcus sp.	Staphylococcus_cohnii	No	No	Yes	No
ASV_1549	88.041	HM352947.1	98.982	HM352958.1	86.005	Staphylococcus_succinus	Staphylococcus_succinus	No	No	Yes	No
ASV_155	88.55	HM352961.1	96.692	HM352951.1	86.514	Staphylococcus sp.	Staphylococcus_kloosii	Yes	No	No	No
ASV_1550	86.26	HM352947.1	96.692	HM352950.1	85.496	Staphylococcus sp.	Staphylococcus_xylosus	No	No	No	No
ASV_1551	86.548	HM352947.1	97.97	HM352950.1	84.772	Staphylococcus_xylosus	Staphylococcus_xylosus	Yes	No	No	No
ASV_1552	85.533	HM352959.1	98.223	HM352944.1	87.817	Staphylococcus_lentus	Staphylococcus_lentus	Yes	No	No	No
ASV_1553	85.751	HM352947.1	99.491	HM352959.1	83.715	Staphylococcus_equorum	Staphylococcus_equorum	Yes	No	No	No
ASV_1554	86.224	HM352947.1	99.745	HM352959.1	84.184	Staphylococcus_equorum	Staphylococcus_equorum	No	No	No	No
ASV_1555	87.056	HM352960.1	95.178	HM352938.1	83.756	Staphylococcus sp.	Staphylococcus_cohnii	No	No	Yes	No
ASV_1556	87.31	HM352960.1	95.431	HM352938.1	84.01	Staphylococcus sp.	Staphylococcus_cohnii	No	No	No	No
ASV_1557	85.751	HM352947.1	97.71	HM352950.1	84.987	Staphylococcus_xylosus	Staphylococcus_xylosus	Yes	No	No	No
ASV_1558	86.041	HM352959.1	98.223	HM352944.1	88.325	Staphylococcus_lentus	Staphylococcus_lentus	Yes	No	No	No
ASV_1559	86.548	HM352947.1	97.462	HM352950.1	84.772	Staphylococcus_xylosus	Staphylococcus_xylosus	Yes	No	No	No
ASV_157	86.957	HM352961.1	99.233	HM352950.1	84.91	Staphylococcus_xylosus	Staphylococcus_xylosus	Yes	No	No	No
ASV_158	86.802	HM352947.1	98.731	HM352958.1	84.772	Staphylococcus_succinus	Staphylococcus_succinus	Yes	No	No	No
ASV_159	80.711	HM352945.1	86.294	HM352949.1	85.533	Staphylococcus sp.	Staphylococcus_simulans	Yes	No	No	No
ASV_16	85.787	HM352961.1	98.477	HM352950.1	84.01	Staphylococcus_xylosus	Staphylococcus_xylosus	Yes	No	No	No
ASV_160	87.532	HM352947.1	99.237	HM352958.1	85.496	Staphylococcus_succinus	Staphylococcus_succinus	Yes	No	No	No
ASV_161	86.294	HM352947.1	98.223	HM352950.1	84.518	Staphylococcus_xylosus	Staphylococcus_xylosus	Yes	No	No	No
ASV_162	86.48	HM352947.1	96.939	HM352950.1	85.714	Staphylococcus sp.	Staphylococcus_xylosus	Yes	No	No	No
ASV_163	87.98	HM352947.1	100	HM352958.1	85.934	Staphylococcus_succinus	Staphylococcus_succinus	Yes	No	No	No
ASV_164	87.468	HM352947.1	98.465	HM352950.1	85.678	Staphylococcus_xylosus	Staphylococcus_xylosus	Yes	No	No	No
ASV_165	85.533	HM352945.1	98.477	HM352959.1	83.756	Staphylococcus_equorum	Staphylococcus_equorum	Yes	No	Yes	No
ASV_166	87.056	HM352947.1	98.223	HM352938.1	84.264	Staphylococcus_cohnii	Staphylococcus_cohnii	Yes	No	Yes	No
ASV_168	85.533	HM352947.1	98.985	HM352959.1	83.503	Staphylococcus_equorum	Staphylococcus_equorum	Yes	No	No	No

ASV_17	85.787	HM352947.1	96.193	HM352950.1	85.025	Staphylococcus sp.	Staphylococcus_xylosus	Yes	No	Yes	No
ASV_171	86.802	HM352947.1	98.731	HM352958.1	84.772	Staphylococcus_succinus	Staphylococcus_succinus	Yes	No	No	No
ASV_172	85.025	HM352945.1	98.731	HM352959.1	82.995	Staphylococcus_equorum	Staphylococcus_equorum	Yes	No	No	No
ASV_177	85.496	HM352960.1	96.438	HM352950.1	84.733	Staphylococcus sp.	Staphylococcus_xylosus	Yes	No	No	No
ASV_178	82.864	HM352919.1	86.99	HM352949.1	84.949	Staphylococcus sp.	Staphylococcus_simulans	No	Yes	No	No
ASV_179	85.934	HM352947.1	100	HM352959.1	83.887	Staphylococcus_equorum	Staphylococcus_equorum	Yes	No	No	No
ASV_18	85.751	HM352940.1	99.491	HM352959.1	83.715	Staphylococcus_equorum	Staphylococcus_equorum	Yes	No	No	No
ASV_182	86.041	HM352959.1	98.223	HM352944.1	88.325	Staphylococcus_lentus	Staphylococcus_lentus	Yes	No	No	No
ASV_183	85.969	HM352940.1	99.745	HM352959.1	83.929	Staphylococcus_equorum	Staphylococcus_equorum	Yes	No	No	No
ASV_184	86.445	HM352961.1	99.233	HM352950.1	84.655	Staphylococcus_xylosus	Staphylococcus_xylosus	Yes	No	No	No
ASV_185	82.741	HM352926.1	88.325	HM352936.1	87.056	Staphylococcus sp.	Staphylococcus_schleiferi	Yes	No	No	No
ASV_186	85.279	HM352947.1	99.239	HM352959.1	83.249	Staphylococcus_equorum	Staphylococcus_equorum	Yes	No	No	No
ASV_187	83.163	HM352919.1	86.768	HM352941.1	84.733	Staphylococcus sp.	Staphylococcus_felis	Yes	No	Yes	No
ASV_188	87.056	HM352960.1	98.477	HM352935.1	85.025	Staphylococcus_saprophyticus	Staphylococcus_saprophyticus	Yes	No	Yes	No
ASV_189	85.714	HM352947.1	99.745	HM352959.1	83.673	Staphylococcus_equorum	Staphylococcus_equorum	Yes	No	No	No
ASV_19	85.279	HM352959.1	99.239	HM352947.1	89.086	Staphylococcus_sciuri	Staphylococcus_sciuri	Yes	Yes	Yes	No
ASV_191	81.934	HM352950.1	86.005	HM352929.1	89.059	Staphylococcus sp.	Staphylococcus_pasteuri	Yes	No	No	No
ASV_192	86.48	HM352961.1	99.235	HM352950.1	84.694	Staphylococcus_xylosus	Staphylococcus_xylosus	Yes	No	No	No
ASV_193	86.99	HM352947.1	98.98	HM352950.1	85.459	Staphylococcus_xylosus	Staphylococcus_xylosus	Yes	No	No	No
ASV_197	81.122	HM352926.1	86.224	HM352949.1	84.949	Staphylococcus sp.	Staphylococcus_simulans	Yes	No	No	No
ASV_198	87.31	HM352960.1	95.431	HM352938.1	84.01	Staphylococcus sp.	Staphylococcus_cohnii	Yes	No	No	No
ASV_199	86.957	HM352961.1	99.233	HM352950.1	85.422	Staphylococcus_xylosus	Staphylococcus_xylosus	Yes	No	No	No
ASV_2	85.279	HM352947.1	99.239	HM352959.1	83.249	Staphylococcus_equorum	Staphylococcus_equorum	Yes	No	Yes	No
ASV_20	85.496	HM352947.1	99.491	HM352959.1	83.715	Staphylococcus_equorum	Staphylococcus_equorum	Yes	No	No	No
ASV_200	85.279	HM352947.1	98.985	HM352959.1	83.249	Staphylococcus_equorum	Staphylococcus_equorum	Yes	No	No	No
ASV_202	86.548	HM352961.1	98.731	HM352950.1	84.518	Staphylococcus_xylosus	Staphylococcus_xylosus	Yes	No	No	No
ASV_204	88.041	HM352947.1	99.491	HM352958.1	86.005	Staphylococcus_succinus	Staphylococcus_succinus	Yes	No	No	No
ASV_205	87.5	HM352947.1	99.49	HM352958.1	85.459	Staphylococcus_succinus	Staphylococcus_succinus	Yes	No	No	No
ASV_207	86.768	HM352959.1	98.473	HM352944.1	89.059	Staphylococcus_lentus	Staphylococcus_lentus	Yes	No	No	No
ASV_208	81.726	HM352956.1	87.056	HM352947.1	86.548	Staphylococcus sp.	Staphylococcus_sciuri	Yes	No	No	No
ASV_21	87.468	HM352965.1	97.954	HM352924.1	87.468	Staphylococcus_hominis	Staphylococcus_hominis	No	No	Yes	No
ASV_211	87.98	HM352947.1	100	HM352958.1	85.934	Staphylococcus_succinus	Staphylococcus_succinus	Yes	No	No	No
ASV_213	88.295	HM352945.1	99.491	HM352954.1	86.514	Staphylococcus_arlettae	Staphylococcus_arlettae	Yes	No	No	No
ASV_214	88.041	HM352960.1	96.183	HM352938.1	84.733	Staphylococcus sp.	Staphylococcus_cohnii	Yes	No	No	No
ASV_216	86.802	HM352947.1	97.716	HM352950.1	85.025	Staphylococcus_xylosus	Staphylococcus_xylosus	Yes	No	No	No
ASV_217	88.071	HM352960.1	98.731	HM352942.1	85.787	Staphylococcus_gallinarum	Staphylococcus_gallinarum	Yes	No	No	No
ASV_219	85.025	HM352947.1	98.985	HM352959.1	82.995	Staphylococcus_equorum	Staphylococcus_equorum	Yes	No	Yes	No
ASV_22	82.443	HM352919.1	86.041	HM352941.1	84.01	Staphylococcus sp.	Staphylococcus_felis	Yes	Yes	No	No
ASV_220	86.514	HM352955.1	99.237	HM352944.1	88.804	Staphylococcus_lentus	Staphylococcus_lentus	Yes	No	No	No
ASV_221	82.995	HM352957.1	86.041	HM352924.1	85.279	Staphylococcus sp.	Staphylococcus_hominis	Yes	No	No	No
ASV_223	87.31	HM352945.1	95.178	HM352938.1	84.264	Staphylococcus sp.	Staphylococcus_cohnii	Yes	No	No	No
ASV_224	85.533	HM352945.1	98.223	HM352959.1	83.756	Staphylococcus_equorum	Staphylococcus_equorum	Yes	No	No	No
ASV_226	85.533	HM352945.1	98.223	HM352959.1	83.756	Staphylococcus_equorum	Staphylococcus_equorum	Yes	No	No	No
ASV_227	86.548	HM352961.1	98.731	HM352950.1	84.518	Staphylococcus_xylosus	Staphylococcus_xylosus	Yes	No	No	No
ASV_228	87.31	HM352947.1	99.239	HM352958.1	85.279	Staphylococcus_succinus	Staphylococcus_succinus	Yes	No	Yes	No
ASV_229	86.041	HM352959.1	97.716	HM352944.1	88.325	Staphylococcus_lentus	Staphylococcus_lentus	Yes	No	No	No
ASV_23	85.279	HM352947.1	99.239	HM352959.1	83.249	Staphylococcus_equorum	Staphylococcus_equorum	Yes	No	No	No
ASV_230	85.496	HM352947.1	99.491	HM352959.1	83.461	Staphylococcus_equorum	Staphylococcus_equorum	Yes	No	No	No
ASV_232	85.279	HM352940.1	98.985	HM352959.1	83.249	Staphylococcus_equorum	Staphylococcus_equorum	Yes	No	No	No
ASV_233	86.041	HM352947.1	97.97	HM352950.1	84.264	Staphylococcus_xylosus	Staphylococcus_xylosus	Yes	No	No	No
ASV_234	81.98	HM352923.1	86.041	HM352941.1	82.234	Staphylococcus sp.	Staphylococcus_felis	Yes	No	Yes	No
ASV_235	81.425	HM352956.1	86.768	HM352947.1	86.26	Staphylococcus sp.	Staphylococcus_sciuri	No	Yes	No	No
ASV_237	87.563	HM352947.1	98.985	HM352958.1	85.533	Staphylococcus_succinus	Staphylococcus_succinus	Yes	No	No	No
ASV_238	86.48	HM352961.1	99.235	HM352950.1	84.694	Staphylococcus_xylosus	Staphylococcus_xylosus	Yes	No	No	No
ASV_239	85.787	HM352959.1	98.985	HM352944.1	88.071	Staphylococcus_lentus	Staphylococcus_lentus	Yes	No	No	No
ASV_240	87.563	HM352960.1	95.685	HM352938.1	84.264	Staphylococcus sp.	Staphylococcus_cohnii	Yes	No	Yes	No
ASV_242	81.98	HM352923.1	87.563	HM352951.1	82.741	Staphylococcus sp.	Staphylococcus_kloosii	Yes	No	Yes	No
ASV_244	86.26	HM352947.1	96.183	HM352950.1	84.987	Staphylococcus sp.	Staphylococcus_xylosus	Yes	No	No	No
ASV_245	85.279	HM352959.1	99.239	HM352947.1	89.086	Staphylococcus_sciuri	Staphylococcus_sciuri	Yes	No	No	No
ASV_247	87.056	HM352965.1	97.208	HM352920.1	87.563	Staphylococcus_capitis	Staphylococcus_capitis	No	No	Yes	No
ASV_248	86.445	HM352947.1	96.931	HM352950.1	85.678	Staphylococcus sp.	Staphylococcus_xylosus	Yes	No	Yes	No
ASV_25	86.802	HM352947.1	98.731	HM352958.1	84.772	Staphylococcus_succinus	Staphylococcus_succinus	Yes	No	No	No
ASV_251	85.787	HM352961.1	98.477	HM352950.1	84.01	Staphylococcus_xylosus	Staphylococcus_xylosus	Yes	No	No	No
ASV_253	85.025	HM352947.1	98.985	HM352959.1	82.995	Staphylococcus_equorum	Staphylococcus_equorum	Yes	No	No	No
ASV_254	88.01	HM352947.1	100	HM352958.1	85.969	Staphylococcus_succinus	Staphylococcus_succinus	Yes	No	No	No
ASV_257	87.532	HM352944.1	99.237	HM352938.1	84.478	Staphylococcus_cohnii	Staphylococcus_cohnii	Yes	No	No	No
ASV_258	82.487	HM352926.1	87.563	HM352947.1	88.832	Staphylococcus sp.	Staphylococcus_sciuri	Yes	No	No	No

ASV_259	85.533	HM352961.1	98.223	HM352950.1	84.264	Staphylococcus_xylosus	Staphylococcus_xylosus	Yes	No	Yes	No
ASV_26	87.31	HM352947.1	98.477	HM352938.1	84.518	Staphylococcus_cohnii	Staphylococcus_cohnii	Yes	No	Yes	No
ASV_260	85.787	HM352947.1	96.193	HM352950.1	85.025	Staphylococcus sp.	Staphylococcus_xylosus	Yes	No	No	No
ASV_262	87.786	HM352960.1	95.929	HM352938.1	84.478	Staphylococcus sp.	Staphylococcus_cohnii	Yes	No	No	No
ASV_264	82.443	HM352919.1	86.041	HM352941.1	84.01	Staphylococcus sp.	Staphylococcus_felis	Yes	No	No	No
ASV_265	86.041	HM352959.1	98.223	HM352944.1	88.325	Staphylococcus_lentus	Staphylococcus_lentus	Yes	No	Yes	No
ASV_266	82.653	HM352919.1	86.768	HM352949.1	84.733	Staphylococcus sp.	Staphylococcus_simulans	Yes	No	No	No
ASV_268	88.01	HM352947.1	100	HM352958.1	85.969	Staphylococcus_succinus	Staphylococcus_succinus	Yes	No	No	No
ASV_27	85.934	HM352959.1	100	HM352947.1	89.77	Staphylococcus_sciuri	Staphylococcus_sciuri	Yes	No	Yes	No
ASV_270	82.188	HM352956.1	87.532	HM352947.1	87.023	Staphylococcus sp.	Staphylococcus_sciuri	Yes	No	No	No
ASV_274	86.514	HM352961.1	98.728	HM352950.1	84.733	Staphylococcus_xylosus	Staphylococcus_xylosus	Yes	No	No	No
ASV_275	86.26	HM352965.1	97.455	HM352923.1	86.514	Staphylococcus_haemolyticus	Staphylococcus_haemolyticus	Yes	No	No	No
ASV_277	85.751	HM352947.1	99.746	HM352959.1	83.715	Staphylococcus_equorum	Staphylococcus_equorum	Yes	No	No	No
ASV_278	85.242	HM352947.1	99.237	HM352959.1	83.461	Staphylococcus_equorum	Staphylococcus_equorum	Yes	No	No	No
ASV_279	82.864	HM352945.1	86.445	HM352958.1	86.189	Staphylococcus sp.	Staphylococcus_succinus	Yes	No	No	No
ASV_28	87.468	HM352965.1	97.954	HM352927.1	87.98	Staphylococcus_warneri	Staphylococcus_warneri	No	No	Yes	No
ASV_281	85.025	HM352947.1	98.985	HM352959.1	82.995	Staphylococcus_equorum	Staphylococcus_equorum	Yes	No	No	No
ASV_282	82.443	HM352965.1	86.26	HM352961.1	86.26	Staphylococcus sp.	Staphylococcus_fleurettii	Yes	No	No	No
ASV_286	86.768	HM352947.1	97.71	HM352950.1	84.987	Staphylococcus_xylosus	Staphylococcus_xylosus	Yes	No	No	No
ASV_29	86.548	HM352947.1	97.462	HM352950.1	84.772	Staphylococcus_xylosus	Staphylococcus_xylosus	Yes	Yes	No	No
ASV_292	85.678	HM352960.1	96.675	HM352950.1	84.91	Staphylococcus sp.	Staphylococcus_xylosus	Yes	No	No	No
ASV_294	85.025	HM352947.1	98.985	HM352959.1	82.995	Staphylococcus_equorum	Staphylococcus_equorum	Yes	No	No	No
ASV_298	87.245	HM352961.1	99.49	HM352950.1	85.204	Staphylococcus_xylosus	Staphylococcus_xylosus	Yes	No	No	No
ASV_3	87.31	HM352960.1	95.431	HM352938.1	84.01	Staphylococcus sp.	Staphylococcus_cohnii	Yes	No	Yes	No
ASV_30	87.212	HM352961.1	99.488	HM352950.1	85.166	Staphylococcus_xylosus	Staphylococcus_xylosus	Yes	Yes	No	No
ASV_300	82.443	HM352955.1	86.768	HM352942.1	85.751	Staphylococcus sp.	Staphylococcus_gallinarum	Yes	No	No	No
ASV_302	82.741	HM352956.1	86.548	HM352954.1	87.31	Staphylococcus sp.	Staphylococcus_arlettae	Yes	No	Yes	No
ASV_303	87.212	HM352947.1	98.21	HM352950.1	85.422	Staphylococcus_xylosus	Staphylococcus_xylosus	Yes	No	No	No
ASV_304	86.041	HM352955.1	98.731	HM352944.1	88.325	Staphylococcus_lentus	Staphylococcus_lentus	Yes	No	Yes	No
ASV_309	85.279	HM352945.1	98.477	HM352959.1	84.01	Staphylococcus_equorum	Staphylococcus_equorum	Yes	No	No	No
ASV_31	87.786	HM352947.1	99.746	HM352958.1	85.751	Staphylococcus_succinus	Staphylococcus_succinus	Yes	No	No	No
ASV_310	82.741	HM352957.1	85.787	HM352924.1	85.025	Staphylococcus sp.	Staphylococcus_hominis	Yes	No	No	No
ASV_311	86.548	HM352961.1	98.731	HM352950.1	84.518	Staphylococcus_xylosus	Staphylococcus_xylosus	Yes	No	No	No
ASV_313	83.206	HM352923.1	87.277	HM352941.1	83.461	Staphylococcus sp.	Staphylococcus_felis	Yes	No	No	No
ASV_314	86.802	HM352947.1	98.731	HM352958.1	84.772	Staphylococcus_succinus	Staphylococcus_succinus	Yes	No	No	No
ASV_315	86.294	HM352947.1	97.208	HM352950.1	84.518	Staphylococcus_xylosus	Staphylococcus_xylosus	Yes	No	Yes	No
ASV_316	87.98	HM352960.1	96.164	HM352938.1	84.655	Staphylococcus sp.	Staphylococcus_cohnii	Yes	No	Yes	No
ASV_317	83.12	HM352919.1	86.735	HM352941.1	84.694	Staphylococcus sp.	Staphylococcus_felis	No	Yes	No	No
ASV_318	82.952	HM352926.1	88.041	HM352947.1	89.313	Staphylococcus sp.	Staphylococcus_sciuri	Yes	No	No	No
ASV_319	85.279	HM352947.1	99.239	HM352959.1	83.249	Staphylococcus_equorum	Staphylococcus_equorum	Yes	No	No	No
ASV_32	81.586	HM352956.1	86.957	HM352947.1	86.445	Staphylococcus sp.	Staphylococcus_sciuri	Yes	No	Yes	No
ASV_321	87.532	HM352960.1	98.982	HM352935.1	85.496	Staphylococcus_saprophyticus	Staphylococcus_saprophyticus	Yes	No	No	No
ASV_322	85.496	HM352947.1	99.491	HM352959.1	83.461	Staphylococcus_equorum	Staphylococcus_equorum	Yes	No	No	No
ASV_325	81.472	HM352926.1	87.056	HM352936.1	86.548	Staphylococcus sp.	Staphylococcus_schleiferi	Yes	No	Yes	No
ASV_327	86.99	HM352959.1	98.724	HM352944.1	89.286	Staphylococcus_lentus	Staphylococcus_lentus	Yes	No	No	No
ASV_328	86.294	HM352961.1	98.477	HM352950.1	84.772	Staphylococcus_xylosus	Staphylococcus_xylosus	No	No	No	Yes
ASV_329	85.751	HM352947.1	99.237	HM352959.1	83.715	Staphylococcus_equorum	Staphylococcus_equorum	Yes	No	No	No
ASV_331	82.234	HM352956.1	87.056	HM352953.1	85.533	Staphylococcus sp.	Staphylococcus_carnosus	Yes	No	Yes	No
ASV_333	88.265	HM352960.1	99.745	HM352942.1	85.969	Staphylococcus_gallinarum	Staphylococcus_gallinarum	Yes	No	No	No
ASV_334	86.041	HM352955.1	98.731	HM352944.1	88.325	Staphylococcus_lentus	Staphylococcus_lentus	Yes	No	No	No
ASV_336	82.097	HM352958.1	87.468	HM352963.1	89.258	Staphylococcus sp.	Staphylococcus_pettenkoferi	Yes	No	No	No
ASV_338	80.964	HM352956.1	86.294	HM352947.1	85.787	Staphylococcus sp.	Staphylococcus_sciuri	Yes	No	No	No
ASV_340	87.245	HM352961.1	99.49	HM352950.1	85.204	Staphylococcus_xylosus	Staphylococcus_xylosus	Yes	No	No	No
ASV_342	83.461	HM352956.1	87.277	HM352954.1	88.041	Staphylococcus sp.	Staphylococcus_arlettae	Yes	No	No	No
ASV_343	88.491	HM352960.1	100	HM352942.1	86.189	Staphylococcus_gallinarum	Staphylococcus_gallinarum	Yes	No	No	No
ASV_345	85.533	HM352947.1	95.939	HM352950.1	84.772	Staphylococcus sp.	Staphylococcus_xylosus	Yes	No	No	No
ASV_347	86.548	HM352961.1	98.731	HM352950.1	84.518	Staphylococcus_xylosus	Staphylococcus_xylosus	Yes	No	No	No
ASV_348	89.796	HM352944.1	99.49	HM352951.1	86.735	Staphylococcus_kloosii	Staphylococcus_kloosii	Yes	No	No	No
ASV_353	82.443	HM352919.1	86.041	HM352941.1	84.01	Staphylococcus sp.	Staphylococcus_felis	Yes	No	No	No
ASV_354	85.787	HM352959.1	98.985	HM352944.1	88.071	Staphylococcus_lentus	Staphylococcus_lentus	Yes	No	Yes	No
ASV_355	87.563	HM352960.1	98.985	HM352942.1	85.279	Staphylococcus_gallinarum	Staphylococcus_gallinarum	Yes	No	No	No
ASV_356	85.787	HM352961.1	98.477	HM352950.1	84.01	Staphylococcus_xylosus	Staphylococcus_xylosus	Yes	No	No	No
ASV_357	86.294	HM352961.1	98.477	HM352950.1	84.772	Staphylococcus_xylosus	Staphylococcus_xylosus	Yes	No	No	No
ASV_358	85.279	HM352947.1	99.239	HM352959.1	83.249	Staphylococcus_equorum	Staphylococcus_equorum	Yes	No	No	No
ASV_359	82.653	HM352919.1	86.768	HM352949.1	84.733	Staphylococcus sp.	Staphylococcus_simulans	No	Yes	No	No
ASV_360	82.443	HM352927.1	86.514	HM352961.1	86.768	Staphylococcus sp.	Staphylococcus_fleurettii	Yes	No	No	No
ASV_361	85.279	HM352959.1	99.239	HM352947.1	89.086	Staphylococcus_sciuri	Staphylococcus_sciuri	Yes	No	Yes	No

ASV_363	85.533	HM352945.1	98.223	HM352959.1	83.756	Staphylococcus_equorum	Staphylococcus_equorum	Yes	No	No	No
ASV_365	85.533	HM352947.1	98.985	HM352959.1	83.503	Staphylococcus_equorum	Staphylococcus_equorum	Yes	No	No	No
ASV_366	88.071	HM352961.1	96.193	HM352951.1	86.041	Staphylococcus sp.	Staphylococcus_kloosii	Yes	No	Yes	No
ASV_367	86.802	HM352965.1	97.208	HM352924.1	86.802	Staphylococcus_hominis	Staphylococcus_hominis	No	Yes	No	No
ASV_369	86.294	HM352947.1	98.223	HM352950.1	84.772	Staphylococcus_xylosus	Staphylococcus_xylosus	Yes	No	No	No
ASV_37	86.802	HM352947.1	98.731	HM352958.1	84.772	Staphylococcus_succinus	Staphylococcus_succinus	Yes	No	Yes	No
ASV_370	82.443	HM352919.1	86.041	HM352941.1	84.01	Staphylococcus sp.	Staphylococcus_felis	Yes	No	No	No
ASV_372	86.294	HM352947.1	98.223	HM352950.1	84.518	Staphylococcus_xylosus	Staphylococcus_xylosus	Yes	No	No	No
ASV_374	81.472	HM352965.1	86.768	HM352920.1	86.041	Staphylococcus sp.	Staphylococcus_capitis	Yes	No	No	No
ASV_375	85.751	HM352947.1	99.746	HM352959.1	83.715	Staphylococcus_equorum	Staphylococcus_equorum	Yes	No	No	No
ASV_376	86.041	HM352965.1	96.701	HM352923.1	86.294	Staphylococcus sp.	Staphylococcus_haemolyticus	Yes	No	No	No
ASV_377	85.025	HM352959.1	98.985	HM352947.1	89.34	Staphylococcus_sciuri	Staphylococcus_sciuri	Yes	No	No	No
ASV_38	86.294	HM352947.1	98.223	HM352950.1	84.772	Staphylococcus_xylosus	Staphylococcus_xylosus	Yes	No	No	No
ASV_381	81.726	HM352965.1	87.31	HM352960.1	87.563	Staphylococcus sp.	Staphylococcus_vitulinus	Yes	No	Yes	No
ASV_382	81.218	HM352956.1	87.056	HM352947.1	86.548	Staphylococcus sp.	Staphylococcus_sciuri	Yes	No	No	No
ASV_386	85.787	HM352965.1	96.954	HM352923.1	86.041	Staphylococcus sp.	Staphylococcus_haemolyticus	Yes	No	No	No
ASV_390	87.056	HM352965.1	97.208	HM352920.1	87.563	Staphylococcus_capitis	Staphylococcus_capitis	Yes	No	No	No
ASV_391	88.747	HM352961.1	96.931	HM352951.1	86.701	Staphylococcus sp.	Staphylococcus_kloosii	Yes	No	No	No
ASV_393	87.563	HM352947.1	98.985	HM352958.1	85.533	Staphylococcus_succinus	Staphylococcus_succinus	Yes	No	No	No
ASV_396	85.279	HM352945.1	98.477	HM352959.1	83.503	Staphylococcus_equorum	Staphylococcus_equorum	Yes	No	No	No
ASV_399	86.445	HM352961.1	99.233	HM352950.1	84.655	Staphylococcus_xylosus	Staphylococcus_xylosus	Yes	No	No	No
ASV_4	86.548	HM352961.1	98.731	HM352950.1	84.518	Staphylococcus_xylosus	Staphylococcus_xylosus	Yes	No	No	No
ASV_40	87.563	HM352960.1	95.685	HM352938.1	84.264	Staphylococcus sp.	Staphylococcus_cohnii	Yes	No	No	No
ASV_400	86.99	HM352947.1	97.959	HM352950.1	85.204	Staphylococcus_xylosus	Staphylococcus_xylosus	Yes	No	No	No
ASV_401	86.294	HM352961.1	98.477	HM352950.1	84.264	Staphylococcus_xylosus	Staphylococcus_xylosus	Yes	No	No	No
ASV_402	85.496	HM352945.1	98.728	HM352959.1	84.224	Staphylococcus_equorum	Staphylococcus_equorum	Yes	No	No	No
ASV_403	83.376	HM352956.1	87.212	HM352954.1	87.98	Staphylococcus sp.	Staphylococcus_arlettae	Yes	No	No	No
ASV_404	86.802	HM352965.1	96.701	HM352927.1	87.056	Staphylococcus sp.	Staphylococcus_warneri	No	Yes	No	No
ASV_405	85.533	HM352923.1	97.716	HM352959.1	82.487	Staphylococcus_equorum	Staphylococcus_equorum	Yes	No	No	No
ASV_406	88.52	HM352960.1	100	HM352942.1	86.224	Staphylococcus_gallinarum	Staphylococcus_gallinarum	Yes	No	No	No
ASV_407	85.025	HM352947.1	98.985	HM352959.1	83.249	Staphylococcus_equorum	Staphylococcus_equorum	Yes	No	No	No
ASV_41	87.31	HM352947.1	99.239	HM352958.1	85.279	Staphylococcus_succinus	Staphylococcus_succinus	Yes	No	No	No
ASV_410	88.041	HM352960.1	99.491	HM352942.1	85.751	Staphylococcus_gallinarum	Staphylococcus_gallinarum	Yes	No	No	No
ASV_411	86.768	HM352947.1	99.237	HM352950.1	84.987	Staphylococcus_xylosus	Staphylococcus_xylosus	Yes	No	No	No
ASV_412	81.218	HM352938.1	85.787	HM352947.1	87.563	Staphylococcus sp.	Staphylococcus_sciuri	Yes	No	No	No
ASV_413	87.31	HM352947.1	99.239	HM352958.1	85.279	Staphylococcus_succinus	Staphylococcus_succinus	Yes	No	No	No
ASV_414	87.056	HM352965.1	97.208	HM352922.1	86.548	Staphylococcus_epidermidis	Staphylococcus_epidermidis	No	Yes	No	No
ASV_415	86.802	HM352965.1	96.954	HM352922.1	86.294	Staphylococcus sp.	Staphylococcus_epidermidis	No	Yes	No	No
ASV_416	88.747	HM352961.1	96.931	HM352951.1	86.701	Staphylococcus sp.	Staphylococcus_kloosii	Yes	No	No	No
ASV_418	85.279	HM352945.1	98.985	HM352959.1	83.503	Staphylococcus_equorum	Staphylococcus_equorum	Yes	No	No	No
ASV_42	86.189	HM352945.1	99.233	HM352959.1	84.399	Staphylococcus_equorum	Staphylococcus_equorum	Yes	No	No	No
ASV_420	87.31	HM352960.1	95.431	HM352938.1	84.01	Staphylococcus sp.	Staphylococcus_cohnii	Yes	No	No	No
ASV_421	86.514	HM352965.1	97.201	HM352923.1	86.768	Staphylococcus sp.	Staphylococcus_haemolyticus	Yes	No	Yes	No
ASV_422	86.26	HM352947.1	96.692	HM352950.1	85.496	Staphylococcus sp.	Staphylococcus_xylosus	Yes	No	No	No
ASV_423	87.817	HM352945.1	98.985	HM352954.1	86.041	Staphylococcus_arlettae	Staphylococcus_arlettae	Yes	No	No	No
ASV_424	85.242	HM352947.1	99.237	HM352959.1	83.461	Staphylococcus_equorum	Staphylococcus_equorum	Yes	No	No	No
ASV_426	87.277	HM352965.1	97.455	HM352922.1	86.768	Staphylococcus_epidermidis	Staphylococcus_epidermidis	No	No	Yes	No
ASV_427	86.701	HM352947.1	98.721	HM352950.1	84.91	Staphylococcus_xylosus	Staphylococcus_xylosus	Yes	No	No	No
ASV_429	86.802	HM352947.1	97.716	HM352950.1	85.025	Staphylococcus_xylosus	Staphylococcus_xylosus	Yes	No	No	No
ASV_43	85.279	HM352940.1	98.985	HM352959.1	83.249	Staphylococcus_equorum	Staphylococcus_equorum	Yes	No	Yes	No
ASV_431	85.279	HM352959.1	99.239	HM352947.1	89.086	Staphylococcus_sciuri	Staphylococcus_sciuri	Yes	No	No	No
ASV_434	86.99	HM352961.1	99.235	HM352950.1	85.459	Staphylococcus_xylosus	Staphylococcus_xylosus	Yes	No	No	No
ASV_435	82.864	HM352955.1	86.701	HM352942.1	85.678	Staphylococcus sp.	Staphylococcus_gallinarum	Yes	No	No	No
ASV_436	86.224	HM352947.1	96.684	HM352950.1	85.459	Staphylococcus sp.	Staphylococcus_xylosus	Yes	No	No	No
ASV_437	85.496	HM352947.1	99.491	HM352959.1	83.461	Staphylococcus_equorum	Staphylococcus_equorum	Yes	No	No	No
ASV_438	86.005	HM352945.1	98.728	HM352959.1	84.224	Staphylococcus_equorum	Staphylococcus_equorum	Yes	No	No	No
ASV_440	80.916	HM352926.1	86.005	HM352949.1	84.733	Staphylococcus sp.	Staphylococcus_simulans	Yes	No	No	No
ASV_441	87.245	HM352947.1	98.214	HM352950.1	85.459	Staphylococcus_xylosus	Staphylococcus_xylosus	Yes	No	No	No
ASV_442	85.751	HM352940.1	99.491	HM352959.1	83.715	Staphylococcus_equorum	Staphylococcus_equorum	Yes	No	No	No
ASV_444	85.934	HM352947.1	99.744	HM352959.1	83.887	Staphylococcus_equorum	Staphylococcus_equorum	Yes	No	No	No
ASV_446	88.325	HM352961.1	96.447	HM352951.1	86.294	Staphylococcus sp.	Staphylococcus_kloosii	Yes	No	No	No
ASV_447	85.787	HM352947.1	96.193	HM352950.1	85.025	Staphylococcus sp.	Staphylococcus_xylosus	Yes	No	No	No
ASV_448	82.952	HM352945.1	86.514	HM352958.1	86.26	Staphylococcus sp.	Staphylococcus_succinus	Yes	No	No	No
ASV_451	86.445	HM352947.1	96.931	HM352950.1	85.678	Staphylococcus sp.	Staphylococcus_xylosus	Yes	No	No	No
ASV_452	87.532	HM352960.1	95.674	HM352938.1	84.224	Staphylococcus sp.	Staphylococcus_cohnii	Yes	No	No	No
ASV_453	85.533	HM352945.1	98.223	HM352959.1	84.264	Staphylococcus_equorum	Staphylococcus_equorum	Yes	No	No	No
ASV_454	86.548	HM352961.1	98.731	HM352950.1	84.518	Staphylococcus_xylosus	Staphylococcus_xylosus	Yes	No	No	No

ASV_455	85.787	HM352947.1	96.193	HM352950.1	85.025	Staphylococcus sp.	Staphylococcus_xylosus	Yes	No	No	No
ASV_456	81.726	HM352956.1	87.056	HM352947.1	86.548	Staphylococcus sp.	Staphylococcus_sciuri	Yes	No	No	No
ASV_457	85.969	HM352945.1	99.235	HM352959.1	84.184	Staphylococcus_equorum	Staphylococcus_equorum	Yes	No	No	No
ASV_458	83.206	HM352926.1	86.768	HM352961.1	87.532	Staphylococcus sp.	Staphylococcus_fleurettii	Yes	No	No	No
ASV_459	87.31	HM352961.1	96.701	HM352938.1	84.772	Staphylococcus sp.	Staphylococcus_cohnii	Yes	No	No	No
ASV_46	85.279	HM352940.1	98.985	HM352959.1	83.249	Staphylococcus_equorum	Staphylococcus_equorum	Yes	No	Yes	No
ASV_460	86.514	HM352947.1	98.473	HM352950.1	85.496	Staphylococcus_xylosus	Staphylococcus_xylosus	Yes	No	No	No
ASV_462	82.995	HM352945.1	86.294	HM352963.1	86.294	Staphylococcus sp.	Staphylococcus_pettenkoferi	Yes	No	No	No
ASV_463	85.279	HM352947.1	98.985	HM352959.1	83.249	Staphylococcus_equorum	Staphylococcus_equorum	Yes	No	No	No
ASV_466	87.5	HM352947.1	99.49	HM352958.1	85.459	Staphylococcus_succinus	Staphylococcus_succinus	Yes	No	No	No
ASV_467	87.056	HM352947.1	98.223	HM352938.1	84.772	Staphylococcus_cohnii	Staphylococcus_cohnii	Yes	No	No	No
ASV_47	85.279	HM352945.1	98.477	HM352959.1	83.503	Staphylococcus_equorum	Staphylococcus_equorum	Yes	No	No	No
ASV_471	81.633	HM352956.1	86.99	HM352947.1	86.48	Staphylococcus sp.	Staphylococcus_sciuri	Yes	No	No	No
ASV_472	88.01	HM352960.1	96.173	HM352938.1	84.694	Staphylococcus sp.	Staphylococcus_cohnii	Yes	No	No	No
ASV_475	85.025	HM352945.1	98.731	HM352959.1	83.756	Staphylococcus_equorum	Staphylococcus_equorum	Yes	No	No	No
ASV_476	87.31	HM352947.1	99.239	HM352958.1	85.279	Staphylococcus_succinus	Staphylococcus_succinus	Yes	No	No	No
ASV_478	87.468	HM352947.1	99.488	HM352958.1	85.422	Staphylococcus_succinus	Staphylococcus_succinus	Yes	No	No	No
ASV_479	86.48	HM352947.1	96.939	HM352950.1	85.714	Staphylococcus sp.	Staphylococcus_xylosus	Yes	No	No	No
ASV_481	83.461	HM352945.1	86.768	HM352963.1	86.768	Staphylococcus sp.	Staphylococcus_pettenkoferi	Yes	No	No	No
ASV_482	85.934	HM352940.1	99.744	HM352959.1	83.887	Staphylococcus_equorum	Staphylococcus_equorum	Yes	No	No	No
ASV_483	87.31	HM352960.1	95.431	HM352938.1	84.01	Staphylococcus sp.	Staphylococcus_cohnii	Yes	No	No	No
ASV_485	87.31	HM352947.1	98.477	HM352938.1	84.518	Staphylococcus_cohnii	Staphylococcus_cohnii	Yes	No	No	No
ASV_486	85.787	HM352961.1	98.477	HM352950.1	84.01	Staphylococcus_xylosus	Staphylococcus_xylosus	Yes	No	No	No
ASV_487	86.445	HM352947.1	96.419	HM352950.1	85.166	Staphylococcus sp.	Staphylococcus_xylosus	Yes	No	No	No
ASV_488	86.768	HM352961.1	98.982	HM352950.1	84.733	Staphylococcus_xylosus	Staphylococcus_xylosus	Yes	No	No	No
ASV_489	86.294	HM352947.1	98.731	HM352950.1	84.518	Staphylococcus_xylosus	Staphylococcus_xylosus	Yes	No	No	No
ASV_49	85.969	HM352947.1	100	HM352959.1	83.929	Staphylococcus_equorum	Staphylococcus_equorum	Yes	No	No	No
ASV_491	85.787	HM352947.1	95.685	HM352950.1	84.518	Staphylococcus sp.	Staphylococcus_xylosus	Yes	No	No	No
ASV_494	85.496	HM352947.1	99.491	HM352959.1	83.461	Staphylococcus_equorum	Staphylococcus_equorum	Yes	No	No	No
ASV_499	86.48	HM352947.1	96.939	HM352950.1	85.714	Staphylococcus sp.	Staphylococcus_xylosus	Yes	No	No	No
ASV_5	86.26	HM352947.1	96.692	HM352950.1	85.496	Staphylococcus sp.	Staphylococcus_xylosus	Yes	No	No	No
ASV_500	85.678	HM352947.1	99.744	HM352959.1	83.632	Staphylococcus_equorum	Staphylococcus_equorum	Yes	No	No	No
ASV_502	82.741	HM352965.1	86.294	HM352954.1	84.518	Staphylococcus sp.	Staphylococcus_arlettae	Yes	No	No	No
ASV_505	82.188	HM352919.1	86.294	HM352949.1	84.264	Staphylococcus sp.	Staphylococcus_simulans	No	Yes	No	No
ASV_507	81.679	HM352965.1	86.48	HM352920.1	85.496	Staphylococcus sp.	Staphylococcus_capitis	Yes	No	No	No
ASV_509	85.279	HM352947.1	99.239	HM352959.1	83.249	Staphylococcus_equorum	Staphylococcus_equorum	Yes	No	No	No
ASV_51	85.787	HM352961.1	98.477	HM352950.1	84.01	Staphylococcus_xylosus	Staphylococcus_xylosus	Yes	No	No	No
ASV_510	85.496	HM352945.1	98.728	HM352959.1	83.715	Staphylococcus_equorum	Staphylococcus_equorum	Yes	No	No	No
ASV_511	85.025	HM352960.1	95.939	HM352950.1	84.264	Staphylococcus sp.	Staphylococcus_xylosus	Yes	No	No	No
ASV_514	87.532	HM352945.1	95.42	HM352938.1	84.478	Staphylococcus sp.	Staphylococcus_cohnii	Yes	No	No	No
ASV_515	80.916	HM352926.1	86.005	HM352949.1	84.733	Staphylococcus sp.	Staphylococcus_simulans	Yes	No	No	No
ASV_52	86.48	HM352961.1	99.235	HM352950.1	84.694	Staphylococcus_xylosus	Staphylococcus_xylosus	Yes	No	No	No
ASV_521	88.52	HM352960.1	100	HM352942.1	86.224	Staphylococcus_gallinarum	Staphylococcus_gallinarum	Yes	No	No	No
ASV_522	87.563	HM352960.1	98.985	HM352942.1	85.279	Staphylococcus_gallinarum	Staphylococcus_gallinarum	Yes	No	No	No
ASV_524	87.212	HM352947.1	98.721	HM352950.1	85.422	Staphylococcus_xylosus	Staphylococcus_xylosus	Yes	No	No	No
ASV_526	80.964	HM352956.1	86.294	HM352947.1	85.787	Staphylococcus sp.	Staphylococcus_sciuri	No	Yes	No	No
ASV_527	81.726	HM352919.1	85.787	HM352941.1	83.249	Staphylococcus sp.	Staphylococcus_felis	No	No	Yes	No
ASV_529	86.99	HM352959.1	98.724	HM352944.1	89.031	Staphylococcus_lentus	Staphylococcus_lentus	Yes	No	No	No
ASV_53	87.277	HM352947.1	99.237	HM352958.1	85.242	Staphylococcus_succinus	Staphylococcus_succinus	Yes	Yes	No	No
ASV_531	87.056	HM352959.1	99.239	HM352963.1	88.071	Staphylococcus_pettenkoferi	Staphylococcus_pettenkoferi	Yes	No	Yes	No
ASV_532	85.279	HM352945.1	98.477	HM352959.1	83.503	Staphylococcus_equorum	Staphylococcus_equorum	Yes	No	No	No
ASV_533	82.188	HM352923.1	86.005	HM352941.1	82.952	Staphylococcus sp.	Staphylococcus_felis	Yes	No	No	No
ASV_534	86.005	HM352961.1	98.728	HM352950.1	84.224	Staphylococcus_xylosus	Staphylococcus_xylosus	Yes	No	No	No
ASV_536	88.776	HM352960.1	99.745	HM352942.1	85.969	Staphylococcus_gallinarum	Staphylococcus_gallinarum	Yes	No	No	No
ASV_537	85.533	HM352947.1	96.447	HM352950.1	85.025	Staphylococcus sp.	Staphylococcus_xylosus	Yes	No	No	No
ASV_538	86.735	HM352947.1	98.214	HM352950.1	85.204	Staphylococcus_xylosus	Staphylococcus_xylosus	Yes	No	No	No
ASV_539	86.294	HM352959.1	97.97	HM352944.1	88.579	Staphylococcus_lentus	Staphylococcus_lentus	Yes	No	No	No
ASV_540	82.741	HM352919.1	87.056	HM352954.1	86.548	Staphylococcus sp.	Staphylococcus_arlettae	Yes	No	No	No
ASV_542	85.533	HM352945.1	98.223	HM352959.1	84.264	Staphylococcus_equorum	Staphylococcus_equorum	Yes	No	No	No
ASV_543	87.056	HM352965.1	97.208	HM352920.1	87.563	Staphylococcus_capitis	Staphylococcus_capitis	Yes	No	No	No
ASV_545	86.548	HM352961.1	98.731	HM352950.1	84.518	Staphylococcus_xylosus	Staphylococcus_xylosus	Yes	No	No	No
ASV_548	82.188	HM352919.1	86.26	HM352949.1	83.461	Staphylococcus sp.	Staphylococcus_simulans	Yes	No	No	No
ASV_550	85.751	HM352947.1	99.746	HM352959.1	83.715	Staphylococcus_equorum	Staphylococcus_equorum	Yes	No	No	No
ASV_551	82.443	HM352919.1	86.041	HM352941.1	84.01	Staphylococcus sp.	Staphylococcus_felis	Yes	Yes	No	No
ASV_553	83.418	HM352956.1	87.245	HM352954.1	88.01	Staphylococcus sp.	Staphylococcus_arlettae	Yes	No	No	No
ASV_554	86.041	HM352955.1	98.731	HM352944.1	88.325	Staphylococcus_lentus	Staphylococcus_lentus	Yes	No	No	No
ASV_555	85.969	HM352947.1	100	HM352959.1	83.929	Staphylococcus_equorum	Staphylococcus_equorum	Yes	No	No	No

ASV_557	81.425	HM352923.1	86.005	HM352949.1	83.461	Staphylococcus sp.	Staphylococcus_simulans	Yes	No	No	No
ASV_558	83.503	HM352965.1	88.071	HM352936.1	85.787	Staphylococcus sp.	Staphylococcus_schleiferi	Yes	No	No	No
ASV_560	86.041	HM352955.1	98.731	HM352944.1	88.325	Staphylococcus_lentus	Staphylococcus_lentus	Yes	No	No	No
ASV_561	87.212	HM352961.1	99.488	HM352950.1	85.166	Staphylococcus_xylosus	Staphylococcus_xylosus	No	No	Yes	No
ASV_567	81.726	HM352926.1	87.563	HM352936.1	87.056	Staphylococcus sp.	Staphylococcus_schleiferi	Yes	No	No	No
ASV_568	86.548	HM352947.1	97.97	HM352950.1	84.772	Staphylococcus_xylosus	Staphylococcus_xylosus	Yes	No	No	No
ASV_57	86.957	HM352947.1	98.977	HM352950.1	85.422	Staphylococcus_xylosus	Staphylococcus_xylosus	Yes	No	No	No
ASV_572	85.787	HM352961.1	98.477	HM352950.1	84.01	Staphylococcus_xylosus	Staphylococcus_xylosus	Yes	No	No	No
ASV_573	85.279	HM352947.1	97.97	HM352959.1	82.741	Staphylococcus_equorum	Staphylococcus_equorum	Yes	No	No	No
ASV_578	85.934	HM352945.1	99.233	HM352959.1	84.143	Staphylococcus_equorum	Staphylococcus_equorum	Yes	No	No	No
ASV_579	88.776	HM352960.1	99.49	HM352942.1	86.48	Staphylococcus_gallinarum	Staphylococcus_gallinarum	Yes	No	No	No
ASV_581	87.786	HM352947.1	99.746	HM352958.1	85.751	Staphylococcus_succinus	Staphylococcus_succinus	Yes	No	No	No
ASV_582	86.041	HM352947.1	95.431	HM352959.1	84.264	Staphylococcus sp.	Staphylococcus_equorum	Yes	No	No	No
ASV_583	85.496	HM352947.1	99.491	HM352959.1	83.715	Staphylococcus_equorum	Staphylococcus_equorum	Yes	No	No	No
ASV_585	87.786	HM352960.1	95.929	HM352938.1	84.478	Staphylococcus sp.	Staphylococcus_cohnii	Yes	No	No	No
ASV_586	86.735	HM352955.1	99.49	HM352944.1	89.031	Staphylococcus_lentus	Staphylococcus_lentus	Yes	No	No	No
ASV_587	85.496	HM352945.1	98.728	HM352959.1	83.715	Staphylococcus_equorum	Staphylococcus_equorum	Yes	No	No	No
ASV_588	87.023	HM352947.1	97.964	HM352950.1	85.242	Staphylococcus_xylosus	Staphylococcus_xylosus	Yes	No	No	No
ASV_589	85.279	HM352959.1	99.239	HM352947.1	89.086	Staphylococcus_sciuri	Staphylococcus_sciuri	Yes	No	No	No
ASV_59	87.817	HM352945.1	98.985	HM352954.1	86.041	Staphylococcus_arlettae	Staphylococcus_arlettae	Yes	No	No	No
ASV_592	86.294	HM352947.1	98.223	HM352950.1	84.772	Staphylococcus_xylosus	Staphylococcus_xylosus	Yes	No	No	No
ASV_593	86.802	HM352965.1	96.954	HM352922.1	86.294	Staphylococcus sp.	Staphylococcus_epidermidis	Yes	No	No	No
ASV_594	86.445	HM352947.1	96.931	HM352950.1	85.678	Staphylococcus sp.	Staphylococcus_xylosus	Yes	No	No	No
ASV_595	85.025	HM352940.1	98.731	HM352959.1	82.995	Staphylococcus_equorum	Staphylococcus_equorum	Yes	No	No	No
ASV_597	89.77	HM352944.1	99.488	HM352951.1	86.701	Staphylococcus_kloosii	Staphylococcus_kloosii	Yes	No	No	No
ASV_598	87.056	HM352965.1	97.208	HM352922.1	86.548	Staphylococcus_epidermidis	Staphylococcus_epidermidis	Yes	No	No	No
ASV_6	87.786	HM352960.1	95.929	HM352938.1	84.478	Staphylococcus sp.	Staphylococcus_cohnii	Yes	Yes	No	No
ASV_60	87.468	HM352965.1	97.954	HM352924.1	87.468	Staphylococcus_hominis	Staphylococcus_hominis	No	No	Yes	No
ASV_600	81.472	HM352923.1	86.041	HM352936.1	83.249	Staphylococcus sp.	Staphylococcus_schleiferi	Yes	No	No	No
ASV_601	81.934	HM352926.1	87.532	HM352936.1	87.023	Staphylococcus sp.	Staphylococcus_schleiferi	Yes	No	No	No
ASV_604	88.491	HM352945.1	99.744	HM352954.1	86.701	Staphylococcus_arlettae	Staphylococcus_arlettae	Yes	No	No	No
ASV_605	87.5	HM352947.1	98.469	HM352950.1	85.714	Staphylococcus_xylosus	Staphylococcus_xylosus	Yes	No	No	No
ASV_607	86.802	HM352959.1	98.985	HM352963.1	87.817	Staphylococcus_pettenkoferi	Staphylococcus_pettenkoferi	Yes	No	No	No
ASV_608	85.496	HM352947.1	99.491	HM352959.1	83.461	Staphylococcus_equorum	Staphylococcus_equorum	Yes	No	No	No
ASV_617	83.673	HM352945.1	86.99	HM352963.1	86.99	Staphylococcus sp.	Staphylococcus_pettenkoferi	Yes	No	No	No
ASV_618	83.969	HM352965.1	88.804	HM352936.1	86.005	Staphylococcus sp.	Staphylococcus_schleiferi	Yes	No	No	No
ASV_62	86.294	HM352947.1	97.208	HM352950.1	84.518	Staphylococcus_xylosus	Staphylococcus_xylosus	Yes	No	No	No
ASV_620	81.98	HM352927.1	86.041	HM352961.1	86.294	Staphylococcus sp.	Staphylococcus_fleurettii	Yes	No	Yes	No
ASV_622	81.934	HM352919.1	86.005	HM352949.1	83.206	Staphylococcus sp.	Staphylococcus_simulans	Yes	No	No	No
ASV_624	86.294	HM352961.1	98.477	HM352950.1	84.772	Staphylococcus_xylosus	Staphylococcus_xylosus	Yes	No	No	No
ASV_625	86.48	HM352959.1	99.745	HM352944.1	88.776	Staphylococcus_lentus	Staphylococcus_lentus	Yes	No	No	No
ASV_626	81.218	HM352944.1	85.787	HM352941.1	81.726	Staphylococcus sp.	Staphylococcus_felis	Yes	No	No	No
ASV_627	86.802	HM352947.1	97.716	HM352950.1	85.025	Staphylococcus_xylosus	Staphylococcus_xylosus	Yes	No	No	No
ASV_63	85.279	HM352947.1	99.239	HM352959.1	83.249	Staphylococcus_equorum	Staphylococcus_equorum	Yes	No	No	No
ASV_630	87.532	HM352965.1	97.71	HM352922.1	87.023	Staphylococcus_epidermidis	Staphylococcus_epidermidis	No	No	Yes	No
ASV_632	87.532	HM352947.1	98.728	HM352938.1	84.733	Staphylococcus_cohnii	Staphylococcus_cohnii	Yes	No	No	No
ASV_637	85.787	HM352961.1	98.477	HM352950.1	84.01	Staphylococcus_xylosus	Staphylococcus_xylosus	Yes	No	No	No
ASV_638	82.995	HM352944.1	88.325	HM352953.1	85.533	Staphylococcus sp.	Staphylococcus_carnosus	Yes	No	No	No
ASV_639	81.33	HM352919.1	86.224	HM352949.1	85.204	Staphylococcus sp.	Staphylococcus_simulans	Yes	No	No	No
ASV_640	81.679	HM352944.1	86.26	HM352941.1	82.188	Staphylococcus sp.	Staphylococcus_felis	Yes	No	No	No
ASV_642	81.726	HM352956.1	87.056	HM352947.1	86.548	Staphylococcus sp.	Staphylococcus_sciuri	Yes	No	No	No
ASV_643	86.26	HM352959.1	99.491	HM352944.1	88.55	Staphylococcus_lentus	Staphylococcus_lentus	Yes	No	No	No
ASV_644	84.439	HM352950.1	87.755	HM352922.1	87.245	Staphylococcus sp.	Staphylococcus_epidermidis	No	Yes	No	No
ASV_645	85.025	HM352947.1	98.985	HM352959.1	82.995	Staphylococcus_equorum	Staphylococcus_equorum	Yes	No	No	No
ASV_647	85.533	HM352961.1	98.223	HM352950.1	83.756	Staphylococcus_xylosus	Staphylococcus_xylosus	Yes	No	No	No
ASV_649	87.277	HM352965.1	97.71	HM352927.1	87.786	Staphylococcus_warneri	Staphylococcus_warneri	No	No	Yes	No
ASV_650	85.934	HM352947.1	100	HM352959.1	83.887	Staphylococcus_equorum	Staphylococcus_equorum	Yes	No	No	No
ASV_652	82.234	HM352945.1	86.294	HM352958.1	85.533	Staphylococcus sp.	Staphylococcus_succinus	Yes	No	No	No
ASV_654	81.633	HM352956.1	86.99	HM352947.1	86.48	Staphylococcus sp.	Staphylococcus_sciuri	Yes	No	No	No
ASV_656	80.457	HM352945.1	86.041	HM352949.1	85.279	Staphylococcus sp.	Staphylococcus_simulans	Yes	No	No	No
ASV_659	81.934	HM352956.1	87.277	HM352947.1	86.768	Staphylococcus sp.	Staphylococcus_sciuri	No	No	Yes	No
ASV_66	88.01	HM352960.1	96.173	HM352938.1	84.694	Staphylococcus sp.	Staphylococcus_cohnii	Yes	No	No	No
ASV_660	85.787	HM352965.1	96.954	HM352923.1	86.041	Staphylococcus sp.	Staphylococcus_haemolyticus	Yes	No	No	No
ASV_663	86.802	HM352947.1	98.731	HM352958.1	84.772	Staphylococcus_succinus	Staphylococcus_succinus	Yes	No	No	No
ASV_664	87.98	HM352960.1	96.164	HM352938.1	84.655	Staphylococcus sp.	Staphylococcus_cohnii	Yes	No	No	No
ASV_665	86.294	HM352947.1	98.223	HM352950.1	84.518	Staphylococcus_xylosus	Staphylococcus_xylosus	Yes	No	No	No
ASV_668	83.249	HM352957.1	86.294	HM352920.1	83.756	Staphylococcus sp.	Staphylococcus_capitis	No	No	Yes	No

ASV_67	85.934	HM352959.1	100	HM352947.1	89.77	Staphylococcus_sciuri	Staphylococcus_sciuri	No	No	Yes	No
ASV_671	82.143	HM352923.1	86.48	HM352941.1	82.908	Staphylococcus sp.	Staphylococcus_felis	Yes	No	No	No
ASV_672	86.294	HM352944.1	97.97	HM352949.1	87.056	Staphylococcus_simulans	Staphylococcus_simulans	Yes	No	Yes	No
ASV_675	83.12	HM352919.1	86.735	HM352941.1	84.694	Staphylococcus sp.	Staphylococcus_felis	Yes	No	No	No
ASV_677	88.041	HM352947.1	99.237	HM352958.1	86.005	Staphylococcus_succinus	Staphylococcus_succinus	Yes	No	No	No
ASV_679	86.26	HM352947.1	96.692	HM352950.1	85.496	Staphylococcus sp.	Staphylococcus_xylosus	Yes	No	No	No
ASV_680	85.496	HM352945.1	98.728	HM352959.1	83.715	Staphylococcus_equorum	Staphylococcus_equorum	Yes	No	No	No
ASV_681	85.533	HM352945.1	98.223	HM352959.1	83.756	Staphylococcus_equorum	Staphylococcus_equorum	Yes	No	No	No
ASV_682	86.041	HM352959.1	98.223	HM352944.1	88.325	Staphylococcus_lentus	Staphylococcus_lentus	Yes	No	No	No
ASV_684	82.188	HM352923.1	86.514	HM352949.1	83.461	Staphylococcus sp.	Staphylococcus_simulans	Yes	No	No	No
ASV_685	81.472	HM352926.1	87.31	HM352936.1	86.802	Staphylococcus sp.	Staphylococcus_schleiferi	Yes	No	No	No
ASV_686	83.756	HM352950.1	87.056	HM352922.1	86.548	Staphylococcus sp.	Staphylococcus_epidermidis	Yes	No	No	No
ASV_687	85.787	HM352947.1	96.193	HM352950.1	85.025	Staphylococcus sp.	Staphylococcus_xylosus	Yes	No	No	No
ASV_688	86.189	HM352947.1	96.675	HM352950.1	85.422	Staphylococcus sp.	Staphylococcus_xylosus	Yes	No	No	No
ASV_69	85.751	HM352959.1	99.746	HM352947.1	89.567	Staphylococcus_sciuri	Staphylococcus_sciuri	Yes	No	No	No
ASV_690	85.969	HM352959.1	100	HM352947.1	89.796	Staphylococcus_sciuri	Staphylococcus_sciuri	Yes	No	No	No
ASV_692	81.472	HM352923.1	85.787	HM352941.1	82.234	Staphylococcus sp.	Staphylococcus_felis	Yes	No	No	No
ASV_693	85.934	HM352940.1	99.744	HM352959.1	83.887	Staphylococcus_equorum	Staphylococcus_equorum	Yes	No	No	No
ASV_694	85.025	HM352947.1	98.985	HM352959.1	83.249	Staphylococcus_equorum	Staphylococcus_equorum	Yes	No	No	No
ASV_695	87.056	HM352947.1	97.462	HM352950.1	84.772	Staphylococcus_xylosus	Staphylococcus_xylosus	Yes	No	No	No
ASV_696	86.041	HM352959.1	98.223	HM352944.1	88.325	Staphylococcus_lentus	Staphylococcus_lentus	Yes	No	No	No
ASV_697	86.294	HM352947.1	98.223	HM352950.1	84.772	Staphylococcus_xylosus	Staphylococcus_xylosus	Yes	No	No	No
ASV_699	85.025	HM352945.1	98.731	HM352959.1	83.756	Staphylococcus_equorum	Staphylococcus_equorum	Yes	No	No	No
ASV_7	86.26	HM352961.1	98.982	HM352950.1	84.478	Staphylococcus_xylosus	Staphylococcus_xylosus	Yes	No	No	No
ASV_70	86.802	HM352947.1	98.731	HM352958.1	84.772	Staphylococcus_succinus	Staphylococcus_succinus	Yes	No	Yes	No
ASV_700	82.188	HM352919.1	86.294	HM352949.1	84.264	Staphylococcus sp.	Staphylococcus_simulans	Yes	No	No	No
ASV_702	80.964	HM352965.1	87.056	HM352961.1	87.056	Staphylococcus sp.	Staphylococcus_fleurettii	Yes	No	No	No
ASV_704	87.31	HM352945.1	95.178	HM352938.1	84.264	Staphylococcus sp.	Staphylococcus_cohnii	Yes	No	Yes	No
ASV_706	82.741	HM352923.1	86.802	HM352941.1	82.995	Staphylococcus sp.	Staphylococcus_felis	Yes	No	No	No
ASV_708	87.056	HM352960.1	95.178	HM352938.1	83.756	Staphylococcus sp.	Staphylococcus_cohnii	Yes	No	No	No
ASV_71	86.514	HM352959.1	98.728	HM352944.1	88.804	Staphylococcus_lentus	Staphylococcus_lentus	Yes	No	No	No
ASV_713	85.934	HM352959.1	100	HM352947.1	89.77	Staphylococcus_sciuri	Staphylococcus_sciuri	Yes	No	No	No
ASV_714	83.756	HM352944.1	87.056	HM352953.1	83.756	Staphylococcus sp.	Staphylococcus_carnosus	Yes	No	No	No
ASV_715	86.005	HM352940.1	98.728	HM352959.1	83.969	Staphylococcus_equorum	Staphylococcus_equorum	Yes	No	No	No
ASV_718	85.279	HM352947.1	98.985	HM352959.1	82.995	Staphylococcus_equorum	Staphylococcus_equorum	Yes	No	No	No
ASV_719	85.279	HM352940.1	98.985	HM352959.1	83.249	Staphylococcus_equorum	Staphylococcus_equorum	Yes	No	No	No
ASV_72	85.533	HM352945.1	98.477	HM352959.1	83.756	Staphylococcus_equorum	Staphylococcus_equorum	Yes	No	No	No
ASV_722	80.916	HM352919.1	85.787	HM352949.1	84.518	Staphylococcus sp.	Staphylococcus_simulans	Yes	No	No	No
ASV_723	80.964	HM352965.1	87.056	HM352961.1	87.056	Staphylococcus sp.	Staphylococcus_fleurettii	Yes	No	No	No
ASV_724	85.787	HM352961.1	98.477	HM352950.1	84.01	Staphylococcus_xylosus	Staphylococcus_xylosus	Yes	No	No	No
ASV_725	82.741	HM352926.1	86.294	HM352961.1	87.056	Staphylococcus sp.	Staphylococcus_fleurettii	No	No	Yes	No
ASV_726	87.31	HM352960.1	95.431	HM352938.1	84.01	Staphylococcus sp.	Staphylococcus_cohnii	Yes	No	No	No
ASV_727	87.563	HM352947.1	98.731	HM352958.1	85.533	Staphylococcus_succinus	Staphylococcus_succinus	Yes	No	No	No
ASV_728	85.934	HM352959.1	100	HM352947.1	89.77	Staphylococcus_sciuri	Staphylococcus_sciuri	No	No	Yes	No
ASV_729	81.633	HM352965.1	87.755	HM352947.1	89.031	Staphylococcus sp.	Staphylococcus_sciuri	Yes	No	No	No
ASV_73	87.817	HM352960.1	99.239	HM352942.1	85.533	Staphylococcus_gallinarum	Staphylococcus_gallinarum	Yes	No	No	No
ASV_730	81.888	HM352955.1	86.48	HM352933.1	86.224	Staphylococcus sp.	Staphylococcus_intermedius	Yes	No	No	No
ASV_732	87.212	HM352961.1	99.488	HM352950.1	85.166	Staphylococcus_xylosus	Staphylococcus_xylosus	Yes	No	No	No
ASV_734	88.776	HM352960.1	99.745	HM352942.1	85.969	Staphylococcus_gallinarum	Staphylococcus_gallinarum	Yes	No	No	No
ASV_737	86.294	HM352947.1	98.731	HM352950.1	84.518	Staphylococcus_xylosus	Staphylococcus_xylosus	Yes	No	No	No
ASV_739	81.679	HM352923.1	86.005	HM352941.1	82.443	Staphylococcus sp.	Staphylococcus_felis	Yes	No	No	No
ASV_74	85.279	HM352940.1	98.985	HM352959.1	83.249	Staphylococcus_equorum	Staphylococcus_equorum	Yes	No	Yes	No
ASV_740	85.787	HM352923.1	96.701	HM352959.1	82.995	Staphylococcus sp.	Staphylococcus_equorum	Yes	No	No	No
ASV_741	85.787	HM352947.1	96.193	HM352950.1	85.025	Staphylococcus sp.	Staphylococcus_xylosus	Yes	No	No	No
ASV_742	83.503	HM352965.1	88.071	HM352936.1	85.787	Staphylococcus sp.	Staphylococcus_schleiferi	No	No	Yes	No
ASV_744	81.218	HM352926.1	87.056	HM352936.1	86.548	Staphylococcus sp.	Staphylococcus_schleiferi	Yes	No	No	No
ASV_745	81.633	HM352923.1	85.969	HM352941.1	82.398	Staphylococcus sp.	Staphylococcus_felis	Yes	No	No	No
ASV_746	86.548	HM352947.1	97.462	HM352950.1	84.264	Staphylococcus_xylosus	Staphylococcus_xylosus	Yes	No	No	No
ASV_747	85.279	HM352940.1	98.985	HM352959.1	83.249	Staphylococcus_equorum	Staphylococcus_equorum	Yes	No	No	No
ASV_748	82.653	HM352965.1	87.755	HM352960.1	85.204	Staphylococcus sp.	Staphylococcus_vitulinus	Yes	No	No	No
ASV_749	83.206	HM352965.1	87.532	HM352961.1	88.041	Staphylococcus sp.	Staphylococcus_fleurettii	Yes	No	No	No
ASV_75	87.468	HM352965.1	97.954	HM352927.1	87.98	Staphylococcus_warneri	Staphylococcus_warneri	Yes	No	Yes	No
ASV_750	81.934	HM352923.1	86.26	HM352941.1	82.697	Staphylococcus sp.	Staphylococcus_felis	Yes	No	No	No
ASV_751	82.741	HM352945.1	86.294	HM352953.1	86.041	Staphylococcus sp.	Staphylococcus_carnosus	Yes	No	No	No
ASV_753	83.249	HM352944.1	86.802	HM352941.1	87.056	Staphylococcus sp.	Staphylococcus_felis	No	No	No	No
ASV_754	85.787	HM352959.1	98.985	HM352944.1	88.071	Staphylococcus_lentus	Staphylococcus_lentus	Yes	No	No	No
ASV_759	84.224	HM352950.1	87.532	HM352922.1	87.023	Staphylococcus sp.	Staphylococcus_epidermidis	Yes	No	No	No

ASV_76	86.005	HM352945.1	98.982	HM352959.1	84.224	Staphylococcus_equorum	Staphylococcus_equorum	Yes	No	No	No
ASV_760	83.206	HM352923.1	87.277	HM352941.1	83.461	Staphylococcus sp.	Staphylococcus_felis	Yes	No	No	No
ASV_763	81.378	HM352919.1	86.26	HM352949.1	84.987	Staphylococcus sp.	Staphylococcus_simulans	Yes	No	No	No
ASV_765	86.26	HM352961.1	98.728	HM352950.1	84.478	Staphylococcus_xylosus	Staphylococcus_xylosus	Yes	No	No	No
ASV_766	82.653	HM352919.1	86.768	HM352949.1	84.733	Staphylococcus sp.	Staphylococcus_simulans	No	Yes	No	No
ASV_769	85.025	HM352947.1	98.985	HM352959.1	83.249	Staphylococcus_equorum	Staphylococcus_equorum	Yes	No	No	No
ASV_77	87.31	HM352947.1	99.239	HM352958.1	85.279	Staphylococcus_succinus	Staphylococcus_succinus	Yes	No	No	No
ASV_770	85.279	HM352947.1	96.954	HM352959.1	83.503	Staphylococcus sp.	Staphylococcus_equorum	Yes	No	No	No
ASV_772	82.234	HM352945.1	86.294	HM352958.1	85.533	Staphylococcus sp.	Staphylococcus_succinus	Yes	No	No	No
ASV_773	83.632	HM352957.1	86.701	HM352924.1	85.934	Staphylococcus sp.	Staphylococcus_hominis	Yes	No	No	No
ASV_774	82.487	HM352940.1	85.787	HM352956.1	87.817	Staphylococcus sp.	Staphylococcus_auricularis	Yes	No	No	No
ASV_777	85.934	HM352947.1	100	HM352959.1	83.887	Staphylococcus_equorum	Staphylococcus_equorum	Yes	No	No	No
ASV_778	87.5	HM352947.1	99.49	HM352958.1	85.459	Staphylococcus_succinus	Staphylococcus_succinus	Yes	No	No	No
ASV_78	87.31	HM352947.1	99.239	HM352958.1	85.279	Staphylococcus_succinus	Staphylococcus_succinus	Yes	No	Yes	No
ASV_784	86.548	HM352947.1	97.462	HM352950.1	84.772	Staphylococcus_xylosus	Staphylococcus_xylosus	Yes	No	No	No
ASV_787	83.206	HM352919.1	87.532	HM352954.1	87.023	Staphylococcus sp.	Staphylococcus_arlettae	Yes	No	No	No
ASV_79	83.756	HM352950.1	87.056	HM352922.1	86.548	Staphylococcus sp.	Staphylococcus_epidermidis	No	No	No	No
ASV_790	85.025	HM352947.1	98.985	HM352959.1	83.249	Staphylococcus_equorum	Staphylococcus_equorum	Yes	No	No	No
ASV_792	81.218	HM352944.1	85.787	HM352941.1	81.726	Staphylococcus sp.	Staphylococcus_felis	Yes	No	No	No
ASV_794	86.005	HM352945.1	98.728	HM352959.1	84.733	Staphylococcus_equorum	Staphylococcus_equorum	Yes	No	No	No
ASV_795	86.294	HM352961.1	95.685	HM352950.1	83.503	Staphylococcus sp.	Staphylococcus_xylosus	Yes	No	No	No
ASV_796	80.964	HM352965.1	87.563	HM352961.1	87.31	Staphylococcus sp.	Staphylococcus_fleurettii	Yes	No	No	No
ASV_798	86.548	HM352959.1	97.716	HM352944.1	88.325	Staphylococcus_lentus	Staphylococcus_lentus	Yes	No	No	No
ASV_799	82.398	HM352923.1	86.99	HM352951.1	83.673	Staphylococcus sp.	Staphylococcus_kloosii	Yes	No	No	No
ASV_8	87.532	HM352960.1	95.674	HM352938.1	84.224	Staphylococcus sp.	Staphylococcus_cohnii	Yes	No	No	No
ASV_80	87.245	HM352947.1	98.214	HM352950.1	85.459	Staphylococcus_xylosus	Staphylococcus_xylosus	Yes	No	No	No
ASV_803	83.249	HM352944.1	86.802	HM352941.1	87.056	Staphylococcus sp.	Staphylococcus_felis	No	No	Yes	No
ASV_807	88.491	HM352945.1	99.744	HM352954.1	86.701	Staphylococcus_arlettae	Staphylococcus_arlettae	Yes	No	No	No
ASV_808	81.472	HM352926.1	87.31	HM352936.1	86.802	Staphylococcus sp.	Staphylococcus_schleiferi	Yes	No	No	No
ASV_809	84.399	HM352950.1	87.724	HM352922.1	87.212	Staphylococcus sp.	Staphylococcus_epidermidis	Yes	No	No	No
ASV_810	82.443	HM352919.1	86.041	HM352941.1	84.01	Staphylococcus sp.	Staphylococcus_felis	Yes	No	No	No
ASV_811	83.12	HM352919.1	86.735	HM352941.1	84.694	Staphylococcus sp.	Staphylococcus_felis	No	Yes	No	No
ASV_815	86.957	HM352947.1	98.977	HM352950.1	85.422	Staphylococcus_xylosus	Staphylococcus_xylosus	Yes	No	No	No
ASV_819	85.714	HM352959.1	99.745	HM352947.1	90.051	Staphylococcus_sciuri	Staphylococcus_sciuri	Yes	No	No	No
ASV_823	83.12	HM352919.1	86.735	HM352941.1	84.694	Staphylococcus sp.	Staphylococcus_felis	No	Yes	No	No
ASV_824	86.445	HM352959.1	99.744	HM352944.1	88.747	Staphylococcus_lentus	Staphylococcus_lentus	Yes	No	No	No
ASV_829	80.964	HM352965.1	87.056	HM352961.1	87.056	Staphylococcus sp.	Staphylococcus_fleurettii	Yes	No	No	No
ASV_83	87.817	HM352960.1	99.239	HM352942.1	85.533	Staphylococcus_gallinarum	Staphylococcus_gallinarum	Yes	No	Yes	No
ASV_831	87.31	HM352960.1	95.431	HM352938.1	84.01	Staphylococcus sp.	Staphylococcus_cohnii	Yes	No	No	No
ASV_832	85.025	HM352959.1	98.985	HM352947.1	88.832	Staphylococcus_sciuri	Staphylococcus_sciuri	No	No	Yes	No
ASV_834	86.548	HM352959.1	97.716	HM352944.1	88.325	Staphylococcus_lentus	Staphylococcus_lentus	Yes	No	No	No
ASV_837	85.934	HM352945.1	99.233	HM352959.1	84.143	Staphylococcus_equorum	Staphylococcus_equorum	Yes	No	No	No
ASV_838	85.969	HM352940.1	99.745	HM352959.1	83.929	Staphylococcus_equorum	Staphylococcus_equorum	Yes	No	No	No
ASV_839	83.503	HM352926.1	87.563	HM352952.1	85.533	Staphylococcus sp.	Staphylococcus_chromogenes	Yes	No	No	No
ASV_840	86.548	HM352947.1	97.462	HM352950.1	84.772	Staphylococcus_xylosus	Staphylococcus_xylosus	Yes	No	No	No
ASV_842	85.787	HM352947.1	95.685	HM352950.1	84.518	Staphylococcus sp.	Staphylococcus_xylosus	No	No	Yes	No
ASV_845	85.279	HM352947.1	99.239	HM352959.1	83.249	Staphylococcus_equorum	Staphylococcus_equorum	Yes	No	No	No
ASV_846	85.969	HM352940.1	99.745	HM352959.1	83.929	Staphylococcus_equorum	Staphylococcus_equorum	Yes	No	No	No
ASV_847	81.472	HM352956.1	85.787	HM352952.1	83.249	Staphylococcus sp.	Staphylococcus_chromogenes	No	No	Yes	No
ASV_849	85.678	HM352959.1	99.744	HM352947.1	90.026	Staphylococcus_sciuri	Staphylococcus_sciuri	Yes	No	No	No
ASV_85	81.586	HM352956.1	86.957	HM352947.1	86.445	Staphylococcus sp.	Staphylococcus_sciuri	No	No	Yes	No
ASV_850	85.496	HM352947.1	99.491	HM352959.1	83.715	Staphylococcus_equorum	Staphylococcus_equorum	Yes	No	No	No
ASV_852	85.279	HM352947.1	98.985	HM352959.1	82.995	Staphylococcus_equorum	Staphylococcus_equorum	Yes	No	No	No
ASV_855	83.969	HM352965.1	88.55	HM352936.1	86.26	Staphylococcus sp.	Staphylococcus_schleiferi	Yes	No	No	No
ASV_859	85.279	HM352940.1	98.985	HM352959.1	83.249	Staphylococcus_equorum	Staphylococcus_equorum	Yes	No	No	No
ASV_86	87.31	HM352965.1	97.208	HM352919.1	87.056	Staphylococcus_aureus	Staphylococcus_aureus	No	No	No	No
ASV_862	86.548	HM352961.1	98.731	HM352950.1	84.518	Staphylococcus_xylosus	Staphylococcus_xylosus	Yes	No	No	No
ASV_863	87.468	HM352947.1	99.488	HM352958.1	85.422	Staphylococcus_succinus	Staphylococcus_succinus	Yes	No	No	No
ASV_864	85.787	HM352947.1	96.193	HM352950.1	85.025	Staphylococcus sp.	Staphylococcus_xylosus	Yes	No	No	No
ASV_865	87.98	HM352947.1	100	HM352958.1	85.934	Staphylococcus_succinus	Staphylococcus_succinus	Yes	No	No	No
ASV_868	86.294	HM352961.1	98.477	HM352950.1	84.772	Staphylococcus_xylosus	Staphylococcus_xylosus	Yes	No	No	No
ASV_869	86.802	HM352947.1	95.431	HM352950.1	85.025	Staphylococcus sp.	Staphylococcus_xylosus	Yes	No	No	No
ASV_871	81.425	HM352926.1	87.532	HM352936.1	86.768	Staphylococcus sp.	Staphylococcus_schleiferi	Yes	No	No	No
ASV_872	82.188	HM352927.1	86.26	HM352961.1	86.514	Staphylococcus sp.	Staphylococcus_fleurettii	Yes	No	No	No
ASV_879	88.55	HM352945.1	99.746	HM352954.1	86.768	Staphylococcus_arlettae	Staphylococcus_arlettae	Yes	No	No	No
ASV_88	86.005	HM352961.1	98.728	HM352950.1	84.733	Staphylococcus_xylosus	Staphylococcus_xylosus	Yes	Yes	No	No
ASV_880	82.995	HM352919.1	86.041	HM352955.1	83.249	Staphylococcus sp.	Staphylococcus_piscifermentans	Yes	No	No	No

ASV_882	85.751	HM352945.1	98.982	HM352959.1	83.969	Staphylococcus_equorum	Staphylococcus_equorum	Yes	No	No	No
ASV_883	86.48	HM352959.1	98.214	HM352944.1	88.776	Staphylococcus_lentus	Staphylococcus_lentus	Yes	No	No	No
ASV_885	87.98	HM352947.1	100	HM352958.1	85.934	Staphylococcus_succinus	Staphylococcus_succinus	Yes	No	No	No
ASV_886	82.995	HM352926.1	86.294	HM352951.1	85.787	Staphylococcus sp.	Staphylococcus_kloosii	Yes	No	No	No
ASV_887	86.548	HM352961.1	96.193	HM352950.1	83.756	Staphylococcus sp.	Staphylococcus_xylosus	Yes	No	No	No
ASV_888	82.995	HM352956.1	86.041	HM352920.1	86.294	Staphylococcus sp.	Staphylococcus_capitis	Yes	No	No	No
ASV_891	86.548	HM352947.1	97.462	HM352950.1	84.772	Staphylococcus_xylosus	Staphylococcus_xylosus	Yes	No	No	No
ASV_896	87.056	HM352965.1	97.208	HM352922.1	86.548	Staphylococcus_epidermidis	Staphylococcus_epidermidis	Yes	No	No	No
ASV_9	85.787	HM352947.1	96.193	HM352950.1	85.025	Staphylococcus sp.	Staphylococcus_xylosus	Yes	No	No	No
ASV_90	83.756	HM352950.1	87.056	HM352922.1	86.548	Staphylococcus sp.	Staphylococcus_epidermidis	No	No	No	No
ASV_900	85.533	HM352945.1	98.223	HM352959.1	84.264	Staphylococcus_equorum	Staphylococcus_equorum	Yes	No	No	No
ASV_901	88.01	HM352960.1	96.173	HM352938.1	84.694	Staphylococcus sp.	Staphylococcus_cohnii	Yes	No	No	No
ASV_902	88.01	HM352947.1	100	HM352958.1	85.969	Staphylococcus_succinus	Staphylococcus_succinus	Yes	No	No	No
ASV_904	82.908	HM352919.1	86.514	HM352941.1	84.478	Staphylococcus sp.	Staphylococcus_felis	No	Yes	No	No
ASV_905	81.98	HM352923.1	86.041	HM352941.1	82.234	Staphylococcus sp.	Staphylococcus_felis	Yes	No	No	No
ASV_908	87.724	HM352965.1	97.954	HM352922.1	87.212	Staphylococcus_epidermidis	Staphylococcus_epidermidis	Yes	No	No	No
ASV_909	83.418	HM352927.1	86.48	HM352961.1	87.755	Staphylococcus sp.	Staphylococcus_fleurettii	Yes	No	No	No
ASV_910	86.548	HM352959.1	97.716	HM352944.1	88.325	Staphylococcus_lentus	Staphylococcus_lentus	Yes	No	No	No
ASV_912	85.751	HM352945.1	98.982	HM352959.1	84.478	Staphylococcus_equorum	Staphylococcus_equorum	Yes	No	No	No
ASV_919	86.294	HM352961.1	98.477	HM352950.1	84.772	Staphylococcus_xylosus	Staphylococcus_xylosus	Yes	No	No	No
ASV_92	87.023	HM352947.1	97.964	HM352950.1	85.242	Staphylococcus_xylosus	Staphylococcus_xylosus	Yes	No	No	No
ASV_924	87.98	HM352960.1	96.164	HM352938.1	84.655	Staphylococcus sp.	Staphylococcus_cohnii	Yes	No	No	No
ASV_926	86.189	HM352945.1	98.977	HM352959.1	84.399	Staphylococcus_equorum	Staphylococcus_equorum	Yes	No	No	No
ASV_93	82.487	HM352945.1	86.041	HM352958.1	85.787	Staphylococcus sp.	Staphylococcus_succinus	Yes	No	Yes	No
ASV_931	88.747	HM352961.1	96.931	HM352951.1	86.701	Staphylococcus sp.	Staphylococcus_kloosii	Yes	No	No	No
ASV_934	80.916	HM352926.1	86.005	HM352949.1	84.733	Staphylococcus sp.	Staphylococcus_simulans	Yes	No	No	No
ASV_935	83.715	HM352965.1	88.295	HM352954.1	85.751	Staphylococcus sp.	Staphylococcus_arlettae	Yes	No	No	No
ASV_936	85.279	HM352947.1	96.701	HM352959.1	83.503	Staphylococcus sp.	Staphylococcus_equorum	Yes	No	No	No
ASV_94	86.48	HM352947.1	96.939	HM352950.1	85.714	Staphylococcus sp.	Staphylococcus_xylosus	Yes	No	No	No
ASV_940	83.418	HM352919.1	87.023	HM352941.1	84.987	Staphylococcus sp.	Staphylococcus_felis	No	No	No	No
ASV_941	80.711	HM352945.1	86.294	HM352949.1	85.533	Staphylococcus sp.	Staphylococcus_simulans	Yes	No	No	No
ASV_942	82.188	HM352965.1	87.786	HM352960.1	88.041	Staphylococcus sp.	Staphylococcus_vitulinus	Yes	No	No	No
ASV_944	87.245	HM352961.1	99.49	HM352950.1	85.204	Staphylococcus_xylosus	Staphylococcus_xylosus	Yes	No	No	No
ASV_947	85.533	HM352940.1	98.985	HM352959.1	83.503	Staphylococcus_equorum	Staphylococcus_equorum	Yes	No	No	No
ASV_948	87.5	HM352965.1	97.959	HM352927.1	88.01	Staphylococcus_warneri	Staphylococcus_warneri	Yes	No	No	No
ASV_949	86.294	HM352947.1	95.431	HM352950.1	83.756	Staphylococcus sp.	Staphylococcus_xylosus	Yes	No	No	No
ASV_95	87.786	HM352947.1	98.982	HM352938.1	84.987	Staphylococcus_cohnii	Staphylococcus_cohnii	Yes	No	No	No
ASV_953	86.548	HM352959.1	97.716	HM352944.1	88.325	Staphylococcus_lentus	Staphylococcus_lentus	Yes	No	No	No
ASV_954	83.206	HM352956.1	87.023	HM352954.1	87.786	Staphylococcus sp.	Staphylococcus_arlettae	Yes	No	No	No
ASV_96	87.277	HM352947.1	98.219	HM352950.1	85.496	Staphylococcus_xylosus	Staphylococcus_xylosus	Yes	No	No	No
ASV_960	86.294	HM352947.1	97.208	HM352950.1	84.518	Staphylococcus_xylosus	Staphylococcus_xylosus	Yes	No	No	No
ASV_961	85.459	HM352965.1	89.796	HM352920.1	89.541	Staphylococcus sp.	Staphylococcus_capitis	Yes	No	No	No
ASV_962	86.224	HM352945.1	99.235	HM352959.1	84.439	Staphylococcus_equorum	Staphylococcus_equorum	Yes	No	No	No
ASV_963	80.711	HM352956.1	85.787	HM352961.1	86.041	Staphylococcus sp.	Staphylococcus_fleurettii	Yes	No	No	No
ASV_97	87.245	HM352961.1	99.49	HM352950.1	85.204	Staphylococcus_xylosus	Staphylococcus_xylosus	Yes	No	No	No
ASV_971	82.487	HM352965.1	86.548	HM352954.1	84.518	Staphylococcus sp.	Staphylococcus_arlettae	Yes	No	No	No
ASV_972	80.916	HM352919.1	85.787	HM352949.1	84.518	Staphylococcus sp.	Staphylococcus_simulans	Yes	No	No	No
ASV_973	81.98	HM352955.1	86.294	HM352942.1	85.279	Staphylococcus sp.	Staphylococcus_gallinarum	Yes	No	No	No
ASV_975	86.735	HM352947.1	98.724	HM352950.1	84.949	Staphylococcus_xylosus	Staphylococcus_xylosus	Yes	No	No	No
ASV_978	87.212	HM352947.1	98.721	HM352950.1	85.422	Staphylococcus_xylosus	Staphylococcus_xylosus	Yes	No	No	No
ASV_979	85.678	HM352947.1	99.744	HM352959.1	83.632	Staphylococcus_equorum	Staphylococcus_equorum	Yes	No	No	No
ASV_98	86.041	HM352959.1	98.223	HM352944.1	88.325	Staphylococcus_lentus	Staphylococcus_lentus	Yes	No	No	No
ASV_980	86.041	HM352959.1	97.716	HM352944.1	88.325	Staphylococcus_lentus	Staphylococcus_lentus	Yes	No	No	No
ASV_983	81.633	HM352919.1	86.514	HM352949.1	84.733	Staphylococcus sp.	Staphylococcus_simulans	Yes	No	No	No
ASV_987	82.353	HM352956.1	87.724	HM352947.1	87.212	Staphylococcus sp.	Staphylococcus_sciuri	Yes	No	No	No
ASV_988	83.632	HM352945.1	96.419	HM352959.1	81.586	Staphylococcus sp.	Staphylococcus_equorum	Yes	No	No	No
ASV_99	85.025	HM352947.1	98.985	HM352959.1	82.995	Staphylococcus_equorum	Staphylococcus_equorum	Yes	No	No	No
ASV_991	86.26	HM352965.1	99.237	HM352957.1	88.295	Staphylococcus_muscae	Staphylococcus_muscae	Yes	No	No	No
ASV_994	86.041	HM352947.1	97.462	HM352950.1	84.518	Staphylococcus_xylosus	Staphylococcus_xylosus	Yes	No	No	No
ASV_995	85.279	HM352940.1	98.985	HM352959.1	83.249	Staphylococcus_equorum	Staphylococcus_equorum	Yes	No	No	No
