# Supplementary material for: Assessing similarities and disparities in the skin microbiota between wild and laboratory populations of house mice
Source: ISME J. 2020 Jun 9;14(10):2367–80. doi: 10.1038/s41396-020-0690-7 (PMC7490391; doi:10.1038/s41396-020-0690-7)
Supplement: Supplementary file 14 — Supplementary Table 6 [file 41396_2020_690_MOESM14_ESM.pdf]

**Supplementary Table 6** Effect of host features and sampling location on major phyla and genera abundance, and diversity measures based on ASVs distribution in the standing communities (DNA) in wild mice (n=115). Significant p values (p ≤ 0.05) are indicated in bold. Un: unclassified

| | | Variance Components | | | % Total Variance | | | Fixed structure | | |
|---|---|---|---|---|---|---|---|---|---|---|
| **Model** | **Response variable** | **Population** | **Haplogroup** | **Residuals** | **Population** | **Haplogroup** | **Residuals** | **Terms** | **$R^2m$** | **Significance** |
| **Abundances of major phyla and genera** | Firmicutes | 0 | 0 | 534281 | 0 | 0 | 100 | Farm | 30.6 | 0.34 |
| | Proteobacteria | 0 | 4.0329 | 123.408 | 0 | 3.16 | 96.83 | Farm | 28.56 | 0.24 |
| | Actinobacteria | 0 | 0 | 99.067 | 0 | 0 | 100 | Farm | 35.1 | 0.23 |
| | Bacteroidetes | 7.2032 | 2.0066 | 44.4982 | 13.41 | 3.73 | 82.85 | Farm | 77.33 | **8.05E-10** |
| | *Staphylococcus* | 0 | 0 | 141.82 | 0 | 0 | 100 | Farm | 30.77 | 0.32 |
| | Un_Muribaculaceae | 10.22 | 0 | 28.566 | 26.34 | 0 | 73.65 | Farm | 81.04 | **5.60E-11** |
| | *Streptomyces* | 0 | 2.9504 | 32.291 | 0 | 8.37 | 91.62 | Farm | 47.43 | **0.006** |
| | *Saccharopolyspora* | 15.86 | 0 | 46.07 | 25.6 | 0 | 74.4 | Farm | 35.38 | **0.051** |
| | Un_Actinobacteria | 2.46 | 0.27 | 34.74 | 6.58 | 0.72 | 92.69 | Farm | 35.97 | 0.12 |
| **Alpha diversity indices** | Shannon | 0 | 0.053 | 0.447 | 0 | 10.73 | 89.26 | Fram | 19.64 | 0.47 |
| | Chao1 | 121.29 | 66.767 | 2267.76 | 4.93 | 2.71 | 92.34 | Fram<br>Sex | 27.7 | 0.32<br>**0.056** |
| | PD | 0.2897 | 0 | 11.59 | 2.43 | 0 | 97.56 | Farm | 23.82 | 0.5 |
| **Beta diversity indices** | Bray-Curtis PC1 | 0.00094 | 0 | 0.011 | 7.5 | 0 | 92.49 | Farm | 84.2 | **1.97E-07** |
| | Bray-Curtis PC2 | 0 | 0 | 0.127 | 0 | 0 | 100 | Farm<br>Weight | 54.52 | **0.032**<br>**0.0512** |
| | Bray-Curtis PC3 | 0.0011 | 0.00033 | 0.009 | 10.68 | 3.17 | 86.13 | Farm | 44.73 | **0.001** |
| | Jaccard PC1 | 0.00015 | 0.0001 | 0.0014 | 9.42 | 6.36 | 84.21 | Farm | 90.8 | **3.30E-17** |
| | Jaccard PC2 | 0.00033 | 2.86E-05 | 0.0015 | 17.17 | 1.48 | 81.34 | Farm | 88.35 | **2.00E-16** |
| | Jaccard PC3 | 0.0006 | 4.4E-05 | 0.00322 | 15.62 | 1.16 | 83.21 | Farm | 51.59 | **0.0034** |
| | Unweighted Unifrac PC1 | 0 | 0 | 0.0047 | 0 | 0 | 100 | Farm | 74.34 | **0.005** |
| | Unweighted Unifrac PC2 | 0 | 0 | 0.0035 | 0 | 0 | 100 | Farm | 86.72 | **0.0005** |
| | Unweighted Unifrac PC3 | 0 | 9E-05 | 0.0048 | 0 | 1.84 | 98.15 | Farm | 27.15 | 0.36 |
| | Weighted Unifrac PC1 | 0 | 0 | 0.009 | 0 | 0 | 100 | Farm | 55.9 | **0.049** |
| | Weighted Unifrac PC2 | 0 | 0 | 0.00519 | 0 | 0 | 100 | Farm | 57.76 | **0.027** |
| | Weighted Unifrac PC3 | 3.78E-05 | 0.00013 | 0.004 | 0.86 | 3.13 | 95.99 | Farm | 34.54 | 0.077 |
