# Supplementary material for: Assessing similarities and disparities in the skin microbiota between wild and laboratory populations of house mice
Source: ISME J. 2020 Jun 9;14(10):2367–80. doi: 10.1038/s41396-020-0690-7 (PMC7490391; doi:10.1038/s41396-020-0690-7)
Supplement: Supplementary file 15 — Supplementary Table 7 [file 41396_2020_690_MOESM15_ESM.pdf]

Supplementary Table 7 Effect of host features and sampling location on major phyla and genera abundance, and diversity measures based on ASVs species distribution in the active communities (RNA) in wild mice (n=115). Significant p values (p ≤ 0.05) are indicated in bold. Un: unclassified. BMI: Body Mass Index

		Variance Components			% Total Variance			Fixed Structure		
**Model**	**Response variable**	**Population**	**Haplogroup**	**Residuals**	**Population**	**Haplogroup**	**Residuals**	**Terms**	**R^2m**	**Significance**
**Abundances of major phyla and genera**	Firmicutes	0	14909	352024	0	4.06	95.93	Farm<br>BMI	53.55	**0.005**<br>**0.008**
	Proteobacteria	9.93	0	77.56	11.34	0	88.65	Farm	22.66	0.61
	Actinobacteria	1.046	0	75.224	1.37	0	98.62	Farm<br>BMI	54.76	**0.01**<br>**0.06**
	Bacteroidetes	1.68	0	38.43	4.2	0	95.79	Farm<br>Weight	41.35	0.06<br>0.06
	Staphylococcus	0	10.903	95.05	0	10.29	89.7	Farm<br>Weight	39.38	**0.04**<br>**0.006**
	Pseudomonas	0	0	50.34	0	0	100	Farm	25.8	0.4
	Saccharopolyspora	0	0	47.43	0	0	100	Farm	49.62	**0.06**
	Streptomyces	3.2	0	47.22	6.36	0	93.63	Farm	29.96	0.15
	Unclassified_Chloroplast	0	3.92	75.37	0	4.95	95.045	Farm	20.03	0.57
**Alpha diversity**	Shannon	0.02	0	0.335	6.26	0	93.73	Farm	51.04	**0.0027**
	Chao1	5861.85	679.19	33459.74	14.65	1.69	83.64	Farm	44.94	**0.00056**
	PD	6.663	0.273	48.269	12.07	0.494	87.43	Farm	58.03	**2.64E-05**
**Beta diversity**	Bray-Curtis PC1	0	0.0002	0.137	0	1.42	98.57	Farm<br>Weight<br>BMI	57.07	**0.0051**<br>**0.047**<br>**0.024**
	Bray-Curtis PC2	0	0.0003	0.0159	0	1.86		Farm<br>Weight	47.92	**0.045**<br>**0.0002**
	Bray-Curtis PC3	0	0.00094	0.0133	0	6.63	93.36	Farm	39.67	**0.032**
	Jaccard PC1	0	5.54E-05	0.0041	0	1.31	98.68	Farm	74.66	**0.00064**
	Jaccard PC2	0	0	0.00517	0	0	100	Farm	63.29	**0.013**
	Jaccard PC3	0.00055	0.00062	0.0068	6.94	7.77	85.27	farm	44.99	**0.0039**
	Unweighted Unifrac PC1	0	0	0.00644	0	0	100	Farm	78.35	**0.0022**
	Unweighted Unifrac PC2	0.00091	0.001	0.0097	7.4	13.14	79.44	Farm	46.44	**0.0009**
	Unweighted Unifrac PC3	0.00016	0.00028	0.0054	2.81	4.81	92.36	Farm<br>BMI	46.24	**0.004**<br>**0.059**
	Weighted Unifrac PC1	0	0.00054	0.0061	0	8.26	91.73	Farm	41.93	**0.027**
	Weighted Unifrac PC2	0	0.0002	0.0034	0	5.6	94.39	Farm	61.75	**0.00074**
	Weighted Unifrac PC3	0.00015	5.02E-05	0.0026	5.17	1.73	93.08	Farm	36.89	**0.029**
