# Supplementary material for: Assessing similarities and disparities in the skin microbiota between wild and laboratory populations of house mice
Source: ISME J. 2020 Jun 9;14(10):2367–80. doi: 10.1038/s41396-020-0690-7 (PMC7490391; doi:10.1038/s41396-020-0690-7)
Supplement: Supplementary file 16 — Supplementary Table 8 [file 41396_2020_690_MOESM16_ESM.pdf]

Supplementary Table 8 Distribution of sampling locations around Espelette in the southwest of France (n=34).

Sampling location	Longitude	Latitude
JJM01	43.327778	-1.476222
JJM02	43.318543	-1.490102
JJM04	43.306704	-1.536797
JJM05	43.298985	-1.54735
JJM06	43.304951	-1.551148
JJM07	43.318737	-1.52807
JJM08	43.337143	-1.495154
JJM09	43.316138	-1.560482
JJM10	43.295746	-1.507033
JJM12	43.265708	-1.362697
JJM13	43.261364	-1.378737
MJJ01	43.343913	-1.551134
MJJ03	43.367031	-1.595092
MJJ06	43.375537	-1.563524
MJJ07	43.376255	-1.582535
MJJ09	43.394596	-1.532853
MJJ10	43.393006	-1.534956
MJJ11	43.421932	-1.542395
MN02	43.362902	-1.450289
MN03	43.365562	-1.447467
MN12	43.389935	-1.492119
MN24	43.328051	-1.328891
MN26	43.343003	-1.290997
MN29	43.329674	-1.320866
MN32	43.335121	-1.311468
MN41	43.322619	-1.292134

MT01	43.35525	-1.447263
MT13	43.388949	-1.335757
MT14	43.376863	-1.325136
MT15	43.353908	-1.452392
MT17	43.383507	-1.346379
MT21	43.376832	-1.33241
MT26	43.368674	-1.342967
MT35	43.380325	-1.366892
