# Supplementary material for: Assessing similarities and disparities in the skin microbiota between wild and laboratory populations of house mice
Source: ISME J. 2020 Jun 9;14(10):2367–80. doi: 10.1038/s41396-020-0690-7 (PMC7490391; doi:10.1038/s41396-020-0690-7)
Supplement: Supplementary file 17 — Supplementary Table 9 [file 41396_2020_690_MOESM17_ESM.pdf]

Supplementary Table 9 Neutral microsatellite loci in wild-caught mice (n=203)

Mouse_ID	Chr01_25	Chr01_25	Chr02_01	Chr02_01	Chr03_21	Chr03_21	Chr03_24	Chr03_24	Chr04_31	Chr04_31	Chr05_15	Chr05_15	Chr05_45	Chr05_45	Chr07_38	Chr07_38	Chr08_11	Chr08_11	Chr09_20	Chr09_20	Chr11_64	Chr11_64	Chr12_05	Chr12_05	Chr13_22	Chr13_22	Chr14_16	Chr14_16	Chr16_21	Chr16_21	Chr17_09	Chr17_09	Chr18_08	Chr18_08	Chr19_08	Chr19_08
JJM0101	14	19	20	26	14	21	26	30	14	18	21	22	9	20	13	17	18	18	21	22	22	23	20	21	22	23	18	19	22	24	13	19	17	20	17	19
JJM0102	19	20	20	22	14	21	22	28	14	18	20	21	18	21	13	17	16	18	21	23	22	23	18	20	23	25	18	19	23	24	8	8	16	20	18	23
JJM0202	19	19	17	27	14	14	26	30	18	19	20	23	18	20	9	9	18	19	21	23	20	23	20	21	23	24	9	**18**	**22**	23	13	14	10	17	22	24
JJM0203A	19	19	17	17	14	21	28	30	14	14	21	22	9	20	18	21	18	19	17	17	20	22	19	21	18	18	15	**15**	**22**	22	8	8	22	22	20	27
JJM0203B	19	19	17	19	15	20	30	31	14	17	21	21	9	21	9	19	18	19	16	23	15	20	19	20	21	22	14	18	22	23	13	19	20	20	16	19
JJM0204	19	19	19	19	20	21	28	28	13	14	21	22	19	20	17	18	18	20	25	25	20	22	22	25	22	22	14	15	22	23	8	15	17	22	15	16
JJM0206	18	20	17	18	13	14	30	30	14	14	22	22	19	20	9	18	18	18	21	23	17	23	20	26	19	23	18	18	22	23	8	13	10	19	22	25
JJM0207	19	19	21	27	14	20	?	?	14	19	20	22	?	?	9	20	18	18	23	23	18	20	20	21	23	24	14	18	?	?	8	13	10	22	22	25
JJM0208	19	19	21	27	14	20	30	31	14	14	19	23	9	20	18	20	18	19	22	23	18	23	20	21	18	19	9	14	23	24	8	14	19	22	25	27
JJM0209	19	19	21	22	19	20	26	31	14	14	20	23	9	20	9	20	18	18	22	23	18	22	20	20	19	23	14	14	22	23	8	8	20	22	22	27
JJM0210	19	19	22	27	14	20	30	31	14	19	19	23	20	22	9	18	18	18	22	23	20	22	20	21	18	23	14	18	22	23	8	13	10	20	22	27
JJM0401	16	23	21	23	19	20	28	29	19	19	22	22	17	17	14	21	18	18	16	18	23	23	21	24	22	23	20	25	22	22	15	16	20	25	20	22
JJM0402	17	22	21	23	14	17	20	26	18	20	11	22	19	20	14	15	16	18	22	23	22	22	20	21	22	23	22	22	21	23	13	13	23	24	22	24
JJM0501	19	20	12	12	17	18	25	31	13	15	21	22	17	20	16	17	18	18	16	21	22	23	21	24	18	21	18	22	21	23	8	8	16	22	21	22
JJM0502	?	?	9	9	16	16	28	28	11	13	19	20	18	18	15	15	14	14	?	?	19	20	19	22	15	18	?	?	18	22	?	?	?	?	20	20
JJM0503	17	19	12	22	14	18	25	25	13	15	20	22	17	17	17	18	18	18	16	16	17	23	21	23	18	18	19	22	17	21	8	8	15	16	16	22
JJM0504	17	19	22	22	14	18	25	25	13	15	20	21	17	20	17	18	18	18	16	25	22	23	23	23	18	21	19	22	17	21	8	21	15	15	16	22
JJM0601	19	20	17	17	18	21	25	30	14	21	21	22	19	20	17	18	18	18	16	16	20	22	20	21	18	24	19	19	21	23	8	21	14	15	22	23
JJM0602	17	19	12	25	20	21	17	25	14	18	22	25	17	20	13	13	18	23	16	23	20	22	19	21	18	21	15	15	21	22	8	8	17	22	22	22
JJM0603	19	20	17	25	20	21	21	30	19	21	22	22	17	20	13	18	18	18	16	24	22	23	20	21	18	22	19	19	21	21	8	8	14	15	23	24
JJM0604	19	19	17	25	15	20	21	25	13	19	21	22	17	22	13	13	18	18	23	24	22	23	21	21	18	22	19	19	21	22	8	15	14	22	16	24
JJM0701	19	19	17	20	15	21	25	28	14	18	20	22	17	18	9	13	19	20	16	23	22	23	19	22	19	25	18	18	22	23	8	8	15	17	16	22
JJM0702	17	19	19	20	14	22	15	18	14	14	12	19	17	17	13	21	18	18	16	17	22	22	20	21	21	23	18	22	22	23	8	14	20	23	23	24
JJM0801	17	19	17	22	20	21	28	30	13	15	12	20	18	22	13	21	18	18	?	?	20	21	21	25	23	24	?	?	21	23	?	?	?	?	23	23
JJM0802	19	19	17	20	14	21	23	28	13	18	19	23	17	20	17	21	18	19	22	23	21	22	21	21	18	18	18	22	18	23	13	13	17	19	11	24
JJM0901	16	17	23	25	14	20	28	28	18	20	17	23	17	19	19	20	15	18	18	23	22	23	20	24	22	23	20	20	22	23	8	16	21	25	20	24
JJM0902	16	19	22	23	20	21	28	28	15	18	12	23	17	17	17	20	18	18	23	23	23	23	22	24	19	22	14	20	20	23	13	16	18	25	16	20
JJM0903	17	20	12	25	20	21	17	30	14	20	20	21	17	22	14	17	18	25	23	23	23	23	20	22	18	25	19	22	21	22	8	13	22	22	20	22
JJM0905	16	20	19	23	14	21	28	28	19	20	11	23	17	19	17	20	15	18	23	23	22	23	22	24	18	22	19	20	20	23	8	13	18	25	20	22
JJM0906	16	20	19	23	20	21	17	28	15	18	16	17	19	20	13	20	15	18	23	23	22	23	22	24	21	22	19	20	23	24	16	21	18	21	16	24
JJM0908	17	21	12	25	14	21	28	28	18	19	17	20	17	19	17	20	15	18	23	26	22	23	20	20	19	22	14	20	22	24	8	13	18	25	16	20
JJM0909	19	24	20	27	19	20	23	25	17	19	20	21	21	24	20	21	18	20	18	22	22	23	20	22	21	22	18	24	22	23	8	16	14	20	20	21
JJM0910	19	19	17	27	19	20	25	25	19	20	21	23	17	21	21	21	18	20	18	28	16	23	20	20	22	22	24	24	22	23	15	16	14	20	20	25
JJM0911	24	24	20	20	19	21	23	26	15	17	20	20	23	24	20	21	16	18	22	22	22	24	21	22	21	22	18	23	23	24	8	15	15	20	16	21
JJM0912	19	24	17	20	19	20	23	25	17	20	20	21	21	24	21	21	18	18	18	22	16	22	20	22	22	22	23	24	23	24	8	16	15	20	16	25
JJM1001	19	20	18	18	18	22	28	30	13	18	21	22	18	20	13	13	18	26	24	24	22	23	24	25	22	23	18	18	23	24	8	15	18	24	16	27
JJM1002	19	20	13	18	19	20	16	17	18	18	21	22	9	20	13	21	18	18	24	25	20	23	19	21	21	22	14	18	23	24	8	14	17	20	16	17
JJM1201	19	19	19	24	14	14	21	29	14	14	17	21	20	21	9	13	18	18	23	23	17	24	21	22	22	25	19	25	21	22	8	19	16	18	22	23
JJM1202	19	19	24	25	20	20	21	21	15	15	21	22	17	20	9	13	18	23	23	23	17	22	20	20	21	22	19	24	16	27	16	17	19	19	23	23
JJM1203	17	19	25	25	14	18	18	21	15	15	21	22	16	25	9	13	18	23	23	26	22	22	19	20	22	22	15	25	16	21	8	8	18	18	20	23
JJM1204	19	19	22	22	19	20	21	29	13	20	21	21	20	22	9	9	23	23	23	23	20	20	19	20	23	25	25	25	16	20	8	13	14	17	20	23
JJM1301	17	17	19	25	14	20	28	28	14	16	20	20	22	23	13	13	18	18	26	26	22	22	22	22	25	25	16	19	23	23	8	8	15	15	16	18
MJJ0101	18	25	17	22	14	21	30	31	13	14	22	23	16	22	22	22	18	18	20	23	23	23	19	21	18	22	10	18	16	22	14	21	18	18	16	25
MJJ0102	18	18	24	24	14	14	28	31	13	14	22	22	20	22	21	22	18	18	23	24	20	20	20	21	18	22	15	21	21	22	13	14	17	17	24	25
MJJ0103	18	25	21	24	14	15	25	31	14	14	22	23	20	21	14	17	18	18	20	23	20	23	19	21	18	22	10	21	16	22	8	21	17	22	25	26
MJJ0104	18	19	21	24	14	22	17	31	13	14	22	22	20	21	21	22	16	18	23	23	20	23	19	21	18	22	10	21	16	22	8	14	17	22	22	25
MJJ0105	18	19	23	24	14	15	25	28	14	14	20	22	18	20	14	22	18	18	22	24	20	23	19	21	19	21	18	19	22	23	8	21	16	17	18	22
MJJ0106	19	25	23	23	14	15	25	31	14	14	22	22	20	22	22	22	18	18	23	24	20	23	19	21	18	22	18	21	16	22	21	21	17	22	26	26
MJJ0107	18	18	24	24	14	15	25	31	13	14	20	22	18	20	14	22	18	18	20	22	20	23	20	21	21	22	21	21	21	22	20	21	17	24	25	26
MJJ0108	18	18	24	24	15	21	25	30	13	14	20	22	16	18	13	14	18	18	22	22	20	23	19	20	21	22	19	21	21	23	19	21	16	24	18	26
MJJ0109	18	18	23	24	14	21	28	30	13	14	20	22	18	20	14	14	18	18	22	24	23	23	19	20	18	22	21	21	22	23	19	21	16	17	22	26
MJJ0111	18	18	23	24	14	21	28	30	14	14	20	22	18	21	13	14	18	18	22	24	20	23	20	21	18	22	21	21	22	23	8	19	16	17	18	26
MJJ0112	18	25	22	24	14	15	25	28	13	14	20	20	16	22	19	22	18	18	20	22	20	23	20	21	18	21	21	21	22	23	8	18	16	16	16	26
MJJ0113	18	18	21	24	20	21	30	30	13	13	22	23	18	22	14	17	18	18	20	22	20	20	19	21	19	21	15	19	21	22	19	21	16	22	18	26
MJJ0114	?	?	23	24	14	21	30	31	13	14	20	23	16	20	14	22	16	18	20	22	20	23	19	20	21	22	15	21	21	22	14	21	17	24	25	26
MJJ0115	18	18	23	24	14	14	31	31	14	14	22	23	20	22	22	22	18	18	20	24	20	20	19	21	19	22	15	21	21	22	14	21	17	22	23	25
MJJ0116	18	25	24	24	15	15	25	25	13	14	19	20	22	22	14	19	18	18	22	22	20	23	20	20	18	22	19	21	22	23	8	19	16	24	18	26
MJJ0117	18	19	21	24	14	21	30	31	13	14	22	23	20	22	17	22	18	18	20	20	20	20	20	21	19	19	15	18	21	22	20	21	22	22	23	26
MJJ0301	19	19	17	19	18	23	31	31	13	13	22	22	17	20	13	17	18	18	23	24	22	23	16	24	18	23	10	10	21	22	8	8	10	17	16	21
MJJ0601	19	20	25	25	20	21	29	34	17	18	21	23	19	20	12	13	18	18	26	27	21	21	18	20	23	23	19	19	21	22	8	8	18	18	21	22
MJJ0602	19	20	18	18	20	21	18	34	14	17	21	24	19	20	13	17	18	18	26	26	21	21	20	22	23	23	19	20	16	22	8	14	18	18	24	25
MJJ0603	19	20	25	25	19	20	29	29	17	17	23	23	19	20	13	17	18	18	26	27	21	21	18	20	22	23	19	20	21	22	8	21	18	18	21	22
MJJ0604	19	19	18	18	19	20	18	29	14	17	20	23	19	21	17	17	18	18	26	26	21	21	19	20	21	22	19	20	22	22	8	14	18	18	24	25
MJJ0605	20	20	18	18	20	20	18	18	14	14	21	21	19	19	13	17	18	18	26	26	21	21	18	22	23	23	19	20	16	22	8	14	18	18	24	25
MJJ0606	19	20	17	18	19	20	29	29	14	17	21	24	19	20	13	13	19	19	23	26	21	21	21	22	21	22	19	19	21	22	8	14	18	18	23	25
MJJ0607	19	20	25	25	20	20	29	29	17	17	21	23	19	20	13	17	18	18	26	27	21	21	17	18	22	23	19	20	21	22	8	21	18	18	22	24
MJJ0608	19	19	17	18	20	20	29	29	17	17	21	24	19	20	13	17	18	18	26	26	21	21	18	22	22	23	19	20	21	22	14	21	18	18	23	25
MJJ0609	20	21	18	18	20	20	18	29	14	14	21	24	21	21	13	17	18	18	23	23	21	21	20	22	23	24	20	20	16	22	8	21	18	18	23	25
MJJ0610	19	19	17	25	20	20	29	29	17	17	23	24	19	20	16	17	18	19	26	26	21	21	17	18	22	23	19	19	22	23	14	21	18	18	22	24
MJJ0611	20	20	17	25	20	20	29	29	17	17	23	23	19	20	16	17	18	18	23	23	21	21	19	20	22	23	19	20	22	22	8	8	18	18	21	22
MJJ0701	?	?	19	21	21	22	25	28	14	14	?	?	18	19	17	22	?	?	?	?	21	23	?	?	?	?	?	?	22	22	?	?	?	?	18	18
MJJ0702	19	19	19	21	19	21	25	30	14	14	21	21	21	22	20	22	19	19	16	17	23	23	19	20	21	22	10	18	22	24	13	19	17	22	24	26
MJJ0703	19	19	21	25	17	22	25	28	13	14	15	20	18	18	16	17	19	19	22	22	23	23	18	19	21	22	10	17	22	22	18	19	18	18	18	26
MJJ0901	18	20	13	21	20	21	28	28	13	14	21	22	21	21	13	17	18	18	16	23	22	24	16	19	20	22	14	20	16	20	8	8	26	26	24	24
MJJ0902	19	20	13	21	20	23	19	28	13	13	21	22	15	21	13	17	18	18	23	23	24	24	16	25	14	20	14	14	20	22	8	19	16	26	22	22
MJJ1001	19	20	19	21	20	21	28	30	13	13	21	21	15	16	13	13	18	18	23	25	22	22	19	19	20	22	19	20	16	22	14	14	14	26	22	24
MJJ1002	20	21	13	21	20	21	19	28	13	13	21	22	15	21	13	13	18	18	23	23	22	24	16	19	14	20	18	20	19	20	8	8	26	26	23	24
MJJ1003	20	20	13	22	21	23	28	28	14	17	21	22	15	19	12	13	18	18	23	23	22	23	19	19	20	25	14	20	20	23	8	8	17	26	18	24
MJJ1004	18	20	13	19	21	23	?	?	13	14	21	21	?	?	14	14	18	18	23	23	22	22	16	16	14	18	18	20	?	?	8	14	16	23	22	24
MJJ1005	18	20	13	13	20	23	19	28	13	14	21	21	21	21	13	13	18	18	23	23	23	24	19	25	14	14	18	22	20	20	8	19	16	23	24	24

MJJ1101	17	19	18	19	20	20	25	28	13	18	21	21	16	20	14	17	18	24	16	23	22	22	19	22	22	25	18	18	21	23	13	13	17	23	16	21
MN0201	19	22	19	19	14	15	19	23	13	16	21	21	18	18	21	21	18	18	24	24	23	23	19	22	23	23	18	19	16	23	8	21	18	18	18	23
MN0202	19	23	18	24	18	20	25	25	14	16	22	25	9	20	13	17	18	18	23	23	18	20	22	24	19	24	18	18	21	23	8	13	17	17	11	27
MN0203	17	19	17	20	14	22	19	28	17	22	21	25	19	21	13	21	18	18	24	28	20	23	19	24	24	25	16	18	20	21	8	13	15	20	15	18
MN0204	16	23	19	24	18	19	19	28	13	22	11	21	15	21	18	23	18	18	23	24	20	22	19	21	19	19	16	18	20	22	8	21	18	18	23	24
MN0205	19	23	17	17	14	19	28	28	14	22	21	25	15	21	18	21	18	18	24	28	23	23	19	22	19	25	16	18	21	27	8	21	18	20	18	24
MN0206	17	19	20	24	18	18	-3	25	14	16	21	22	17	17	17	21	16	18	23	23	18	20	19	24	19	25	18	18	17	21	8	21	20	20	15	22
MN0207	16	20	19	20	14	22	25	28	14	22	21	22	20	21	21	21	18	18	23	24	20	22	19	24	19	25	16	16	20	23	19	21	18	20	15	23
MN0301	19	19	19	22	14	15	28	28	14	22	10	11	14	15	14	23	18	19	24	24	22	23	21	22	18	22	18	19	22	23	8	21	18	26	17	24
MN0302	19	22	19	19	15	21	19	19	22	22	21	21	18	21	17	23	18	19	24	24	23	23	22	25	18	22	18	19	22	22	21	21	17	18	22	22
MN0303	19	19	19	22	15	21	20	28	22	22	11	21	15	18	14	23	18	19	23	24	23	23	22	25	18	22	18	19	23	24	21	21	18	18	22	24
MN0304	19	19	17	22	15	21	20	28	14	22	11	11	15	15	14	23	18	18	23	24	22	23	22	25	18	18	18	19	24	27	8	21	18	26	17	22
MN0305	22	23	19	19	15	18	19	19	22	22	11	21	16	16	17	23	19	19	24	24	23	23	19	20	22	22	18	19	22	22	8	21	18	18	11	22
MN0306	20	22	19	19	15	21	19	19	22	22	11	21	15	18	14	23	19	19	24	24	23	23	20	25	22	22	18	18	21	22	8	21	17	18	22	22
MN0307	19	22	19	22	15	21	19	20	22	22	11	22	15	18	14	23	18	19	24	24	22	23	20	22	21	22	18	18	22	23	8	21	17	18	21	22
MN0308	19	20	19	22	15	18	19	28	16	22	11	22	15	18	14	17	18	18	24	24	23	23	21	22	18	22	18	19	22	24	21	21	17	18	17	24
MN0309	19	19	19	22	15	21	20	28	16	22	11	21	19	19	13	14	18	18	23	24	22	23	21	22	18	18	18	19	23	24	8	21	18	18	17	22
MN0310	19	20	19	19	15	21	19	28	22	22	21	22	15	21	17	23	18	18	24	24	23	23	20	22	18	22	18	19	22	24	8	21	18	18	22	24
MN0311	19	22	19	22	15	21	20	28	22	22	21	21	16	16	13	14	18	19	24	24	23	23	20	22	22	22	18	18	22	22	8	21	18	18	21	22
MN0312	19	19	19	22	18	21	19	20	16	22	11	21	15	18	13	14	18	19	24	24	22	23	22	25	18	18	18	18	23	24	8	21	18	26	17	24
MN1202	19	19	13	19	14	23	28	30	13	17	19	21	17	21	13	21	19	30	23	23	22	22	20	20	21	21	19	21	21	22	8	8	17	23	23	24
MN2401	19	20	24	25	20	21	25	28	13	13	20	21	19	20	17	21	18	24	23	23	22	25	20	20	19	25	18	19	23	23	8	8	18	20	18	22
MN2402	18	19	25	25	15	18	18	28	13	14	21	23	19	20	18	21	18	18	23	26	22	22	19	20	23	23	19	24	21	23	8	13	14	17	16	22
MN2601	16	19	22	27	20	22	25	26	14	21	21	21	20	22	13	21	18	18	23	24	17	17	16	20	22	23	19	23	21	27	8	13	14	17	18	22
MN2602	19	19	12	25	20	22	26	28	21	21	21	21	18	20	13	22	18	18	16	23	22	25	16	20	15	20	23	27	21	27	8	19	14	18	18	22
MN2603	16	19	22	25	20	22	25	28	14	21	21	21	20	22	21	22	18	18	16	24	17	22	16	16	15	22	18	23	23	27	13	19	14	14	16	22
MN2605	16	25	12	27	22	23	25	25	14	21	15	25	18	18	12	13	18	18	23	23	23	25	20	23	20	22	18	27	21	23	8	8	18	18	18	22
MN2606	16	19	?	?	?	?	25	26	21	21	21	25	18	20	13	13	18	18	23	23	17	25	20	20	20	23	19	27	16	21	8	8	17	18	18	18
MN2608	19	25	?	?	?	?	25	26	21	21	15	21	20	20	9	13	18	18	23	23	23	25	20	23	22	23	11	27	16	24	8	19	17	18	18	22
MN2609	19	19	?	?	?	?	25	28	14	21	15	25	18	20	12	13	18	18	23	23	17	25	20	23	22	23	18	19	16	24	8	19	17	18	18	22
MN2610	19	19	?	?	?	?	25	26	13	14	21	25	18	22	13	22	18	18	23	24	17	23	16	23	15	23	23	27	21	23	8	13	14	18	18	22
MN2611	19	19	?	?	?	?	25	26	13	21	21	25	18	20	12	13	18	18	23	23	17	25	20	23	21	23	20	27	16	21	8	19	18	22	18	27
MN2613	19	19	?	?	?	?	25	28	14	21	21	25	18	20	12	13	18	20	23	23	23	25	20	23	20	22	11	19	21	23	8	8	17	18	18	22
MN2614	16	19	?	?	?	?	25	26	21	21	21	25	18	20	9	13	18	18	23	23	17	25	20	20	20	25	18	27	16	24	8	8	18	18	18	22
MN2616	16	19	12	18	20	22	25	28	14	21	21	21	18	20	13	22	18	19	23	23	17	17	19	20	15	23	19	19	15	16	8	17	16	18	18	27
MN2901	19	21	24	25	19	19	23	23	14	17	19	21	18	19	13	21	19	20	22	23	22	22	19	23	18	21	9	24	16	21	8	13	18	20	18	21
MN2902	19	21	20	25	19	21	18	23	22	22	21	21	18	20	13	22	18	20	18	23	22	23	20	22	23	23	19	19	23	24	13	19	16	18	24	25
MN3201	19	25	20	22	20	21	18	28	13	20	22	22	17	18	9	21	16	18	18	23	22	23	19	20	22	23	19	19	21	23	8	13	18	22	22	27
MN3202	19	25	22	25	14	14	31	31	14	22	22	23	20	22	20	21	19	20	23	23	22	22	19	20	21	22	26	26	23	23	8	8	18	18	20	22
MN3203	19	21	21	22	14	22	26	28	20	22	21	21	18	20	13	21	18	19	18	27	23	25	19	19	22	22	16	23	21	22	8	19	18	27	24	27
MN3204	20	21	20	20	19	20	31	31	13	14	21	22	20	22	9	13	18	18	18	23	22	22	20	24	23	23	11	26	23	24	8	8	17	17	16	25
MN3205	21	25	25	25	21	23	25	28	22	22	22	22	20	20	17	23	18	20	20	23	23	23	20	20	22	23	11	19	22	24	8	8	16	22	11	22
MN3206	25	25	20	20	20	21	26	30	13	20	22	23	17	18	9	21	18	20	18	20	22	23	19	20	22	23	11	26	23	24	8	13	22	27	26	27
MN3207	19	25	20	22	14	21	18	30	13	22	22	23	16	18	20	21	18	18	18	23	22	25	19	20	23	23	19	19	21	23	8	8	22	27	22	27
MN3208	21	21	20	25	20	21	26	28	13	21	22	22	20	22	21	21	18	18	20	20	22	23	19	20	22	23	19	19	23	24	8	8	16	16	26	27
MN3209	21	25	25	25	20	21	26	28	20	21	22	22	19	20	21	21	18	18	20	20	22	22	19	20	22	23	19	26	22	24	8	8	16	27	16	27
MN3210	25	25	20	20	20	23	25	26	20	21	22	22	18	22	13	21	18	20	20	20	22	23	19	20	22	23	19	26	23	24	8	13	22	27	16	27
MN3211	25	25	20	20	20	21	26	30	20	20	22	22	18	22	20	21	18	18	18	20	22	22	19	20	23	23	19	26	22	24	8	13	22	27	16	22
MN3212	21	25	25	25	20	21	26	28	20	21	22	22	19	20	21	21	18	18	20	23	23	23	19	20	23	23	11	19	23	24	8	8	16	27	26	27
MN3214	21	25	20	25	20	21	18	28	21	21	22	22	18	20	13	21	18	20	18	20	22	23	19	20	22	23	19	26	23	24	8	13	16	27	16	27
MN3215	25	25	20	22	14	23	26	30	21	22	22	23	16	18	9	13	18	18	18	20	22	25	20	20	22	23	19	26	23	24	8	13	22	27	16	22
MN4101	20	20	25	25	20	21	18	28	13	17	21	25	9	18	12	13	20	24	24	27	22	22	20	22	18	25	19	25	16	23	8	19	17	22	16	22
MN4102	19	19	20	25	20	21	20	25	13	13	21	22	19	20	9	14	19	25	22	24	22	23	22	22	22	25	18	26	21	23	8	18	14	18	20	23
MN4103	19	19	12	25	18	20	26	26	13	19	21	23	19	20	13	17	18	20	22	27	22	23	16	22	22	23	19	23	23	25	18	19	18	18	14	22
MN4104	19	21	25	25	15	22	28	29	13	22	21	21	9	9	14	21	19	19	20	23	23	25	19	24	23	26	18	18	22	23	8	16	14	14	22	23
MN4105	19	19	24	25	18	20	26	26	14	19	21	23	18	20	9	17	18	20	22	27	22	22	16	22	22	23	19	26	21	23	8	19	18	22	14	22
MN4106	20	21	25	25	20	22	18	28	13	13	21	25	9	20	17	21	18	19	20	25	23	25	19	24	21	26	18	19	16	23	16	21	14	17	22	23
MN4107	19	19	12	25	20	22	26	31	14	19	21	23	18	20	13	13	18	20	22	29	22	23	19	22	22	23	11	26	23	25	8	17	18	27	14	22
MT0101	23	23	17	17	15	19	25	30	13	13	21	25	16	17	9	18	18	19	16	16	18	23	20	25	22	23	19	19	21	23	8	19	17	20	17	17
MT0102	19	23	17	17	19	20	25	30	13	15	19	21	17	20	9	13	18	19	16	24	23	24	16	20	22	23	18	19	21	23	8	19	17	20	17	24
MT0103	23	23	17	25	15	19	25	30	13	14	19	25	17	20	13	18	18	19	16	21	18	23	20	25	22	23	16	19	21	22	8	19	18	20	16	17
MT0104	14	23	13	25	19	21	25	25	14	15	19	21	16	17	9	18	18	19	21	23	20	23	20	24	22	23	19	23	21	23	8	19	18	18	16	24
MT0105	23	23	17	25	15	19	30	30	14	15	21	25	19	20	9	18	18	19	21	24	18	23	16	20	22	23	16	19	22	23	8	8	17	20	16	24
MT0106	19	23	17	17	19	19	25	30	13	14	19	21	17	20	13	13	19	19	16	16	18	24	19	20	23	23	19	19	21	23	8	19	18	18	16	24
MT0107	19	23	17	25	18	19	25	30	13	15	19	25	17	20	9	18	19	19	16	21	23	24	16	20	22	23	19	19	21	21	8	19	18	20	17	24
MT0108	14	23	23	25	15	19	19	25	13	15	15	19	17	20	9	13	18	19	16	23	20	20	20	24	23	23	18	19	21	23	8	8	16	17	15	24
MT0109	23	23	22	23	14	15	19	25	14	22	11	25	18	20	9	21	18	25	16	23	20	23	20	25	22	23	16	19	20	22	8	19	18	18	14	15
MT0110	23	23	20	23	15	22	19	19	13	14	11	21	18	21	12	13	18	19	16	23	20	23	20	22	22	23	16	20	19	21	8	19	15	18	18	26
MT0111	23	23	17	23	15	22	19	25	14	15	20	21	17	21	9	21	18	19	23	24	20	23	19	20	22	23	18	23	21	22	19	19	16	16	24	26
MT0112	23	23	23	25	15	22	19	19	13	22	15	25	19	21	12	13	18	19	16	24	20	23	19	20	22	23	16	18	20	22	8	8	16	20	15	18
MT0113	18	19	19	22	15	18	25	26	13	13	11	22	17	18	18	18	18	18	16	28	23	23	19	20	22	25	18	18	20	22	8	21	15	24	11	25
MT0114	16	23	20	22	15	22	19	19	13	14	11	15	17	19	12	13	18	19	21	24	22	23	19	20	22	23	16	20	20	22	8	19	15	18	15	18
MT0115	23	23	20	23	14	15	25	28	13	14	15	25	20	21	13	21	18	19	21	23	23	23	19	20	19	22	16	18	21	22	19	19	16	20	15	18
MT0116	18	23	22	23	15	18	19	26	13	22	11	11	18	20	13	18	18	19	16	23	20	23	19	20	22	23	18	18	20	22	19	21	16	24	11	18
MT1301	17	17	18	20	14	21	18	26	13	15	17	21	21	22	12	13	18	18	16	16	24	24	21	26	20	23	23	23	21	24	8	19	14	18	22	23
MT1302	17	19	20	20	14	21	18	28	13	16	17	21	20	22	13	17	16	18	16	23	24	24	19	21	20	21	19	23	22	23	8	13	18	18	22	23
MT1303	17	19	18	20	14	14	18	23	15	16	17	21	17	22	13	13	18	18	23	23	22	24	21	22	20	21	19	26	22	24	13	19	18	18	22	23
MT1304	17	19	18	25	21	21	26	31	15	15	17	21	17	22	12	13	18	18	16	23	22	24	19	26	21	23	19	23	22	24	13	19	14	18	23	24
MT1305	17	19	18	20	14	21	18	31	13	15	21	21	17	22	12	13	18	18	16	23	24	24	21	26	20	21	19	19	22	23	13	19	14	18	22	23
MT1306	19	19	18	20	14	21	23	31	15	15	17	21	17	17	12	13	18	18	16	23	22	24	20	22	20	21	19	26	22	24	19	19	18	18	22	24
MT1307	19	19	18	20	14	21	23	28	15	16	17	21	16	22	13	13	18	18	16	23	24	24	21	22	20	20	19	19	22	24	8	19	18	18	23	23
MT1308	17	19	18	20	21	21	28	31	15	16	21	21	17	22	13	17	18	18	16	23	24	24	21	21	21	21	19	19	21	23	13	19	18	18	22	24
MT1401	19	19	18	19	15	15	19	25	13	22	21	21	17	23	13	15	18	18	23	23	22	26	19	20	24	24	9	18	21	22	8	13	19	19	11	17

**MT1402**	19	19	19	23	15	21	28	30	22	22	17	21	18	23	13	15	18	18	23	23	18	26	19	20	24	24	9	19	21	22	8	13	10	10	11	18
**MT1501**	18	19	19	24	17	17	19	20	14	18	10	11	21	24	14	21	18	18	28	28	20	23	20	25	18	18	18	19	19	21	8	21	17	17	23	25
**MT1502**	16	19	20	24	15	17	19	20	14	18	11	21	18	24	17	21	18	18	28	28	20	23	20	25	17	18	16	18	20	21	8	21	18	18	22	26
**MT1503**	17	19	19	22	17	21	22	23	18	18	11	20	16	21	14	17	18	18	23	28	20	23	20	25	18	22	16	18	20	21	21	21	15	24	22	23
**MT1701**	19	19	19	19	14	23	26	26	13	14	17	23	17	22	13	15	18	18	22	23	22	26	20	21	21	21	15	16	22	24	14	14	24	24	22	24
**MT1702**	19	20	22	24	15	23	23	29	13	13	21	25	17	20	13	14	19	20	22	23	18	23	16	20	23	25	15	18	22	25	15	19	22	23	15	18
**MT1704**	19	22	20	22	23	23	29	29	13	13	17	21	21	22	13	14	15	18	23	25	17	24	20	21	21	25	9	26	21	23	13	15	16	17	16	24
**MT1705**	19	19	22	22	15	17	25	25	13	20	21	21	20	22	9	18	15	19	22	23	23	24	20	21	19	20	15	23	22	23	8	19	17	23	15	15
**MT1706**	19	19	19	22	15	17	25	30	13	17	21	21	20	25	15	18	15	18	22	23	22	24	20	20	20	21	15	18	22	22	15	19	22	23	15	22
**MT1707**	19	20	18	25	20	20	21	30	13	21	20	21	20	22	18	20	18	19	24	26	22	23	20	21	18	21	18	19	19	23	8	13	18	19	24	26
**MT2101**	19	20	12	18	15	21	21	30	19	19	20	20	20	20	17	18	23	23	23	23	24	24	19	20	22	22	11	16	21	22	8	8	18	18	25	26
**MT2102**	19	20	12	18	15	21	21	30	19	19	20	20	19	20	17	18	23	23	23	23	18	24	19	20	22	22	11	16	21	22	8	19	16	18	25	26
**MT2103**	20	20	12	18	15	21	21	30	14	19	20	20	19	20	18	20	20	23	23	28	18	24	20	20	18	22	11	11	21	22	8	19	16	18	25	26
**MT2104**	19	20	12	19	20	21	30	30	13	19	20	21	20	20	17	18	18	23	23	23	18	24	20	20	22	25	11	24	22	22	8	19	18	18	18	26
**MT2105**	20	20	12	12	21	21	30	30	14	19	20	20	20	20	15	18	20	23	23	28	24	24	20	20	20	20	11	11	22	22	8	19	18	18	26	26
**MT2106**	19	20	12	18	20	21	25	30	13	14	20	20	18	20	15	17	18	20	23	28	24	24	19	20	20	22	11	16	20	21	8	19	16	18	26	26
**MT2107**	20	20	18	18	21	22	25	30	13	14	20	20	20	20	18	18	23	23	23	28	18	24	20	20	22	25	11	16	22	22	13	19	18	18	26	26
**MT2108**	20	20	12	12	21	21	25	30	13	19	20	20	20	20	18	18	23	23	23	28	18	24	20	20	22	22	11	16	22	22	19	19	18	18	25	26
**MT2109**	19	21	19	19	15	23	30	30	13	15	21	21	22	26	15	17	18	18	23	28	22	23	20	23	25	25	16	19	22	22	13	19	16	18	16	18
**MT2110**	20	20	12	19	21	22	30	30	18	19	17	17	19	20	17	18	18	23	23	23	17	24	20	23	18	18	9	16	21	21	8	19	18	18	18	26
**MT2112**	14	19	12	12	21	22	23	30	19	19	20	20	20	20	18	18	20	23	23	23	18	24	19	20	22	25	16	16	21	22	8	19	18	18	18	26
**MT2113**	20	20	12	12	21	21	30	30	19	19	17	20	20	20	18	18	18	23	23	28	18	24	19	20	20	22	11	11	21	22	13	19	18	18	18	26
**MT2114**	19	20	12	12	19	19	30	30	14	14	17	20	18	20	15	18	18	20	23	23	17	24	19	20	20	22	11	16	20	21	8	19	10	18	18	26
**MT2115**	20	20	12	18	21	22	30	30	13	14	20	20	20	20	17	23	18	23	23	23	21	21	19	20	20	22	16	16	22	22	13	17	16	18	25	26
**MT2116**	20	20	12	12	21	22	30	30	14	19	17	20	20	20	17	18	20	23	22	23	18	24	19	20	20	22	11	16	20	22	8	8	16	18	16	21
**MT2117**	19	19	12	18	15	22	21	30	18	19	20	20	19	20	17	18	18	20	23	23	17	24	19	20	22	22	9	11	21	22	8	19	18	18	18	26
**MT2118**	?	?	12	12	21	22	30	30	18	19	20	20	19	20	16	18	18	23	?	?	24	24	20	23	22	22	?	?	21	22	?	?	?	?	21	26
**MT2120**	19	20	12	18	20	21	25	30	19	19	20	20	20	20	18	18	20	23	23	28	18	24	19	20	20	22	11	16	22	22	8	19	18	18	26	26
**MT2121**	19	20	12	18	21	22	30	30	19	19	17	20	19	20	17	18	22	23	23	28	17	24	20	23	20	22	11	16	21	22	13	19	18	18	25	26
**MT2601**	19	19	12	19	14	15	19	26	13	13	21	21	9	19	13	14	18	18	16	28	17	23	20	21	22	22	16	18	21	26	8	8	19	22	16	17
**MT2603**	18	19	19	19	15	20	28	30	13	13	21	22	17	19	17	18	18	23	26	26	23	24	22	23	21	22	9	18	16	24	8	19	22	22	20	21
**MT3501**	17	19	18	20	?	?	25	31	20	20	19	21	22	23	8	9	16	18	22	23	24	25	20	21	17	18	18	19	21	22	8	8	17	17	15	16
**MT3502**	19	19	27	27	?	?	29	31	14	14	20	20	17	23	9	20	15	19	22	23	22	24	16	20	19	25	16	23	21	22	8	14	18	18	18	26
**MT3503**	?	?	22	27	14	15	25	29	14	17	20	21	22	23	20	23	15	19	22	23	22	24	19	20	19	25	16	16	22	26	8	19	18	18	16	26
**MT3506**	17	19	20	27	15	21	25	31	17	20	19	21	17	22	15	17	18	18	23	23	22	25	20	21	18	19	19	23	22	22	8	14	17	17	18	26
**MT3507**	19	19	?	?	19	21	29	31	14	14	20	21	17	23	8	9	18	19	16	22	23	24	16	20	23	25	14	23	22	22	8	14	18	18	18	26
**MT3508**	17	19	18	18	20	21	25	25	13	14	20	21	9	23	9	18	18	18	22	23	22	22	19	21	21	25	16	18	22	23	8	19	18	18	17	18
**MT3509**	19	19	19	22	19	20	25	29	13	14	21	21	9	25	21	23	15	19	16	25	22	23	16	20	25	25	18	19	20	22	8	8	18	26	18	20
**MT3511**	?	?	24	27	19	21	29	31	17	18	19	20	19	23	15	18	15	19	23	23	22	23	16	21	18	24	18	18	22	24	8	8	17	22	15	18
**MT3513**	19	19	18	18	20	21	25	29	13	14	20	21	9	23	8	9	15	18	23	23	22	23	19	21	21	25	18	19	20	23	8	8	18	18	17	18
