# Supplementary material for: Assessing similarities and disparities in the skin microbiota between wild and laboratory populations of house mice
Source: ISME J. 2020 Jun 9;14(10):2367–80. doi: 10.1038/s41396-020-0690-7 (PMC7490391; doi:10.1038/s41396-020-0690-7)
Supplement: Supplementary file 18 — Supplementary Table 10 [file 41396_2020_690_MOESM18_ESM.pdf]

Supplementary Table 10. List of primers used for the *tuf* gene library.

First Step PCR primers

Sequence name	Sequence
1-F	ACACTCTTTCCCTACACGACGCTCTTCCGATCTGCCAGTTGAGGACGTATTCT
2-F	ACACTCTTTCCCTACACGACGCTCTTCCGATCTTGCCAGTTGAGGACGTATTCT
3-F	ACACTCTTTCCCTACACGACGCTCTTCCGATCTGTGCCAGTTGAGGACGTATTCT
4-F	ACACTCTTTCCCTACACGACGCTCTTCCGATCTCGAGCCAGTTGAGGACGTATTCT
5-F	ACACTCTTTCCCTACACGACGCTCTTCCGATCTATGAGCCAGTTGAGGACGTATTCT
6-F	ACACTCTTTCCCTACACGACGCTCTTCCGATCTTGCGAGCCAGTTGAGGACGTATTCT
7-F	ACACTCTTTCCCTACACGACGCTCTTCCGATCTGAGTGGCCAGTTGAGGACGTATTCT
1-R	GTGACTGGAGTTCAGACGTGTGCTCTTCCGATCTCCATTTCAGTACCTTCTGGTAA
2-R	GTGACTGGAGTTCAGACGTGTGCTCTTCCGATCTACCATTTCAGTACCTTCTGGTAA
3-R	GTGACTGGAGTTCAGACGTGTGCTCTTCCGATCTTCCCATTTCAGTACCTTCTGGTAA
4-R	GTGACTGGAGTTCAGACGTGTGCTCTTCCGATCTCTACCATTTCAGTACCTTCTGGTAA
5-R	GTGACTGGAGTTCAGACGTGTGCTCTTCCGATCTGATACCATTTCAGTACCTTCTGGTAA
6-R	GTGACTGGAGTTCAGACGTGTGCTCTTCCGATCTACTCACCATTTCAGTACCTTCTGGTAA
7-R	GTGACTGGAGTTCAGACGTGTGCTCTTCCGATCTTTCTCTCCATTTCAGTACCTTCTGGTAA
8-R	GTGACTGGAGTTCAGACGTGTGCTCTTCCGATCTCACTTCTCCATTTCAGTACCTTCTGGTAA

Second Step PCR primers

Sequence name	Sequence
F-1	AATGATACGGCGACCACCGAGATCTACAC AACCGCAT ACACTCTTTCCCTACACG
F-2	AATGATACGGCGACCACCGAGATCTACAC AAGGCCTT ACACTCTTTCCCTACACG
F-3	AATGATACGGCGACCACCGAGATCTACAC AGAGTGTG ACACTCTTTCCCTACACG
F-4	AATGATACGGCGACCACCGAGATCTACAC CACAAGTC ACACTCTTTCCCTACACG
F-5	AATGATACGGCGACCACCGAGATCTACAC CGTTCCTA ACACTCTTTCCCTACACG
F-6	AATGATACGGCGACCACCGAGATCTACAC GCTTGGAT ACACTCTTTCCCTACACG
F-7	AATGATACGGCGACCACCGAGATCTACAC GTCAACAC ACACTCTTTCCCTACACG
F-8	AATGATACGGCGACCACCGAGATCTACAC GTCACTGA ACACTCTTTCCCTACACG
F-9	AATGATACGGCGACCACCGAGATCTACAC TCTCGTCA ACACTCTTTCCCTACACG
F-10	AATGATACGGCGACCACCGAGATCTACAC TTGGTACG ACACTCTTTCCCTACACG
F-11	AATGATACGGCGACCACCGAGATCTACAC CGTTGGAT ACACTCTTTCCCTACACG
F-12	AATGATACGGCGACCACCGAGATCTACAC CGTTAAGC ACACTCTTTCCCTACACG
F-13	AATGATACGGCGACCACCGAGATCTACAC ACAGCTCA ACACTCTTTCCCTACACG
F-14	AATGATACGGCGACCACCGAGATCTACAC GACAAGTG ACACTCTTTCCCTACACG
F-15	AATGATACGGCGACCACCGAGATCTACAC GCATTAGC ACACTCTTTCCCTACACG
R-A	CAAGCAGAAGACGGCATACGAGAT AACCGGAA GTGACTGGAGTTCAGACG
R-B	CAAGCAGAAGACGGCATACGAGAT AGAGTGAC GTGACTGGAGTTCAGACG
R-C	CAAGCAGAAGACGGCATACGAGAT CAACTGGT GTGACTGGAGTTCAGACG

R-D	CAAGCAGAAGACGGCATACGAGAT CGTTCGTT GTGACTGGAGTTCAGACG
R-E	CAAGCAGAAGACGGCATACGAGAT CTGTTCAC GTGACTGGAGTTCAGACG
R-F	CAAGCAGAAGACGGCATACGAGAT GCTTGCAA GTGACTGGAGTTCAGACG
R-G	CAAGCAGAAGACGGCATACGAGAT GTCAACTG GTGACTGGAGTTCAGACG
R-H	CAAGCAGAAGACGGCATACGAGAT TCCTCATG GTGACTGGAGTTCAGACG
R-I	CAAGCAGAAGACGGCATACGAGAT TCGACTAG GTGACTGGAGTTCAGACG
R-J	CAAGCAGAAGACGGCATACGAGAT TTGCAAGC GTGACTGGAGTTCAGACG
R-K	CAAGCAGAAGACGGCATACGAGAT AGAGGTGT GTGACTGGAGTTCAGACG
R-L	CAAGCAGAAGACGGCATACGAGAT GCTACGAT GTGACTGGAGTTCAGACG
R-M	CAAGCAGAAGACGGCATACGAGAT GTCAAGAG GTGACTGGAGTTCAGACG
R-N	CAAGCAGAAGACGGCATACGAGAT ATGGTAGG GTGACTGGAGTTCAGACG
R-O	CAAGCAGAAGACGGCATACGAGAT GACTTCAG GTGACTGGAGTTCAGACG
